# Induction of the filamentous prophage, Pf4, is conditionally regulated by PA3572 (difP) in Pseudomonas aeruginosa biofilms

**DOI:** 10.1099/mic.0.001770

**Published:** 2026-09-09

**Authors:** Sanaya Z. Patell, Martin Welch

**Affiliations:** 1 Department of Biochemistry, University of Cambridge, Tennis Court Road, CB2 1QW, Cambridge, UK

**Keywords:** biofilm, cell envelope stress, CpxP, Pf4, prophage, *Pseudomonas aeruginosa*

## Abstract

*Pseudomonas aeruginosa* is an opportunistic Gram-negative pathogen, often associated with airway infections in the lungs of people with cystic fibrosis (CF). Biofilms of many *P. aeruginosa* strains, such as PAO1, produce copious quantities of filamentous Pf4 phage. High titres of Pf4 phage are associated with a decline in lung function in people living with CF. We previously showed that genes encoding Pf4 and also an unlinked hypothetical protein, PA3572, form part of the core biofilm-associated transcriptome in PAO1. Here, using reporter gene assays, we confirm that PA3572 (which we designate *difP* – depresses induction of filamentous Pf4 prophage) is strongly induced in biofilms of PAO1. Transcriptomic analysis of a *difP* deletion mutant revealed elevated expression of several Pf4 ORFs, as well as elevated expression of the cell envelope stress-associated protein, CpxP. These modulations were confirmed by quantitative reverse transcription-PCR, indicating that DifP functions to depress Pf4 gene expression. Consistent with this, Pf4 phage titres were elevated in a Δ*difP* mutant, whereas over-expression of *difP* depressed Pf4 titres and Pf4 gene expression. This effect of *difP* was abolished in a *cpxP* mutant, indicating that DifP-dependent regulation of Pf4 production is likely linked with cell envelope stress sensing. Taken together and by contrast with most other regulators of Pf4 identified to date (which promote Pf4 production in biofilms), our data indicate that DifP plays a role in restraining Pf4 production in PAO1 biofilms.

## Introduction

*Pseudomonas aeruginosa* is an opportunistic human pathogen, infamous for its ability to acquire resistance to antibiotics and to form biofilms on biotic and abiotic surfaces [1]. Around 20% of all *P. aeruginosa* strains – including many clinical strains – carry chromosomally integrated Pf4-like filamentous prophage [2]. Indeed, the most widely used laboratory strain of *P. aeruginosa* (PAO1) encodes a Pf4 prophage at loci PA0715–PA0729 [3]. Removal of Pf4 prophage from the *P. aeruginosa* chromosome leads to a diminution in virulence [3] and the released Pf4 phage have been shown to form liquid crystal-like structures that protect the nearby bacterial cells from antibiotic action [4].

Pf4 is induced to enter the ‘lytic cycle’ in the presence of certain antibiotics and genotoxic agents and during the biofilm mode of growth. However, the resulting filamentous Pf4 phage do not necessarily always lyse the host cells. Instead, the phage are extruded across the cell envelope, effectively converting the host cell into a phage-producing factory [2]. Phage production by these cells is increased by a mechanism involving a Pf4-encoded protein, termed PfsE. PfsE acts in a twofold manner. First it binds to the major pilin protein (PilC), which controls assembly and disassembly of the type IV pilus at the *P. aeruginosa* cell surface. Since type 4 pili are the main receptor for Pf4, this PfsE–PilC interaction blocks superinfection of the cells by already-released phage [5]. Second, PfsE binds to and inhibits PqsA, which is involved in the synthesis of the quorum-sensing molecule PQS. Via mechanism(s) not yet understood, this inhibition of PqsA appears to increase the replication efficiency of Pf4 [6]. Given this elegant rewiring of host cell physiology, it is not surprising that Pf4 phage can accumulate to high titres in scenarios where biofilms thrive, such as in the chronic airway infections associated with cystic fibrosis (CF). Indeed, in that disease scenario, the presence of high filamentous phage titres is associated with a long-term decline in lung function [7].

Bacterial host cells harbouring prophage are usually resistant to superinfection by the free (lytic) forms of those phage. As noted above, in the case of Pf4, phage exclusion by PfsE-dependent masking of the Pf4 receptor (type IV pili) contributes towards this. Other factors that have been implicated in the prevention of superinfection include the Pf4 encoded repressor (Pf4r [8]) and also an excisionase (XisF4) which regulates prophage excision and also up-regulates expression of the prophage replication initiator gene, PA0727 [9]. The expression of *xisF4* is normally repressed by a pair of host-derived xenogenic silencer proteins (MvaU and MvaT), although this repression is relieved during growth as biofilms [10]. Interestingly, it has been recently reported that superinfective Pf4 phage themselves manifest a curious genome reduction in which a phage-encoded reverse transcriptase and a nearby non-coding RNA (*phrD*) cooperate to generate a truncated version of Pf4. This truncated Pf4 carries all the genes necessary for Pf4 infection and replication (including XisF4) but lacks Pf4r, leading to a rapid increase in Pf4 production by biofilm cells [11].

A meta-analysis of different studies comparing the transcriptome of PAO1 cultures grown as biofilms or as planktonic cultures revealed that the ‘core’ biofilm-associated transcriptome is remarkably small [12]. The growth medium, planktonic comparator growth phase (exponential, stationary) and specific biofilm model employed had a far greater impact on the transcriptome than the growth mode per se. However, one of the core transcripts that was up-regulated across multiple studies was encoded by an uncharacterized locus, PA3572. In this short communication, we show that PA3572 – which we now designate as DifP (depresses induction of filamentous Pf4 prophage) – plays a regulatory role by suppressing induction of Pf4 in PAO1 biofilms.

## Methods

Details describing strain construction, quantitative reverse transcription PCR (Q-RT-PCR), primers, β-galactosidase assays, cloning, etc. are described in the Methods S1 section.

## Results and discussion

**Fig. 1.**
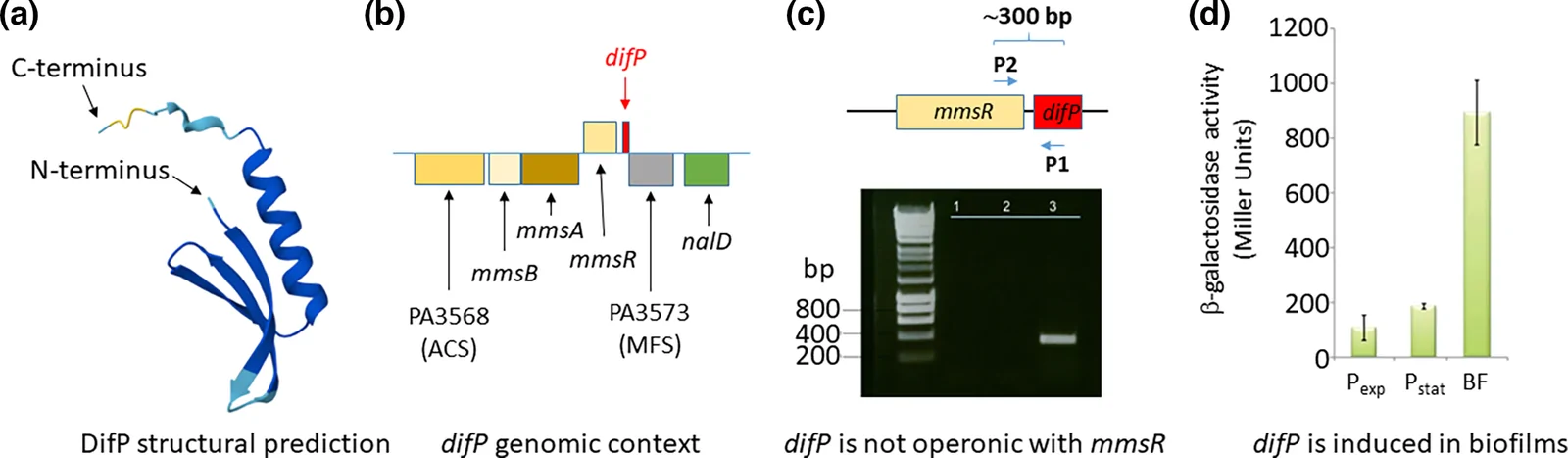
Predicted structure of the DifP protein, its genetic context and biofilm-associated expression profile. (**a**) Structure of the protein encoded by *difP* (PA3572), based on AlphaFold3 prediction. (**b**) Genetic context of *difP* (indicated in red). (**c**) *MmsR* and *difP* are encoded on different transcripts. Primers P1 (5′-ATGTTCATGGCGATTCATCC-3′) and P2 (5′-GTACGCCTGCCAGTTGCT-3′) which point towards one another and anneal to the 5′ end of the *difP* ORF and the 3′ end of the *mmsR* ORF, respectively, were used in PCR reactions to amplify the ca. 300 bp region in between. Lane 1; cDNA (+RT) from total mRNA extracted from PAO1 biofilm cells, lane 2; as in lane 1 but no RT reaction carried out prior to PCR, lane 3; PAO1 gDNA. (**d**) DifP expression is elevated in 2-day-old continuous flow tube biofilms of PAO1. Expression levels were assessed from the β-galactosidase activity associated with the *lacZ*-based *difP* transcriptional reporter (see text for details). Comparators were planktonic cells grown in the same medium (alanine-glycerol-salts-yeast extract medium, AGSY [17]) harvested during the exponential or stationary phase of growth, as indicated. Data represent the mean±sd for three biological replicates of each sample.

PA3572 encodes a 58aa-long protein that is predicted by AlphaFold3 to adopt a twisted three-stranded β-sheet (residues Met1-Phe27) overlain by a single α-helix (Glu30-Arg45) (Fig. 1a). The ORF encoding the protein is located adjacent to a transcriptional regulator, *mmsR* (Fig. 1b). However, Q-RT-PCR revealed that PA3572 is not operonic with *mmsR* (Fig. 1c).

The protein encoded by PA3572 is conserved in *P. aeruginosa* and related pseudomonads (Fig. S1). Alignment of the PA3572 protein sequence with the non-redundant NCBI database confirmed that most of the amino acids in PA3572 are conserved, with Trp16 and Cys19 almost completely conserved, perhaps indicative of functional importance (Fig. S2). However, we also noted that the genetic context of PA3572 is different in *P. aeruginosa* compared with other pseudomonads (Fig. S3). In *P. aeruginosa* strains, PA3572 is located between the *mmsR-mmsAB*/ACS gene cluster and a divergently transcribed predicted major facilitator family (MFS) transporter, PA3573. By contrast, in other pseudomonads, the PA3572 homologue is located between the MFS and a Zn-dependent dehydrogenase (homologous to PA3567 in PAO1), and the *mmsR-mmsAB*/ACS gene cluster (Fig. S3) is absent. The *mmsR-mmsAB*/ACS ORFs appear to have inserted between PA3572 and the Zn-dependent dehydrogenase in *P. aeruginosa*.

To confirm the induction of PA3572 expression during biofilm growth, the region 300 bp upstream of the PA3572 start codon was cloned into mini-CTX-*lacZ* [13] and the resulting construct was integrated at a neutral site in the PAO1 chromosome. Following excision of the plasmid backbone, the reporter construct was grown in shake flasks (i.e. as planktonic culture) or as continuous-flow tube biofilms (Fig. S4) [14]. The biofilm cells were harvested by extrusion of the attached biomass into buffer, followed by vigorous resuspension. Following determination of cell density (OD_600_) in each sample, the β-galactosidase activity was measured. PA3572 was induced ∼5-fold (*cf.* the planktonic cultures) in the biofilm cultures (Fig. 1d).

**Fig. 2.**
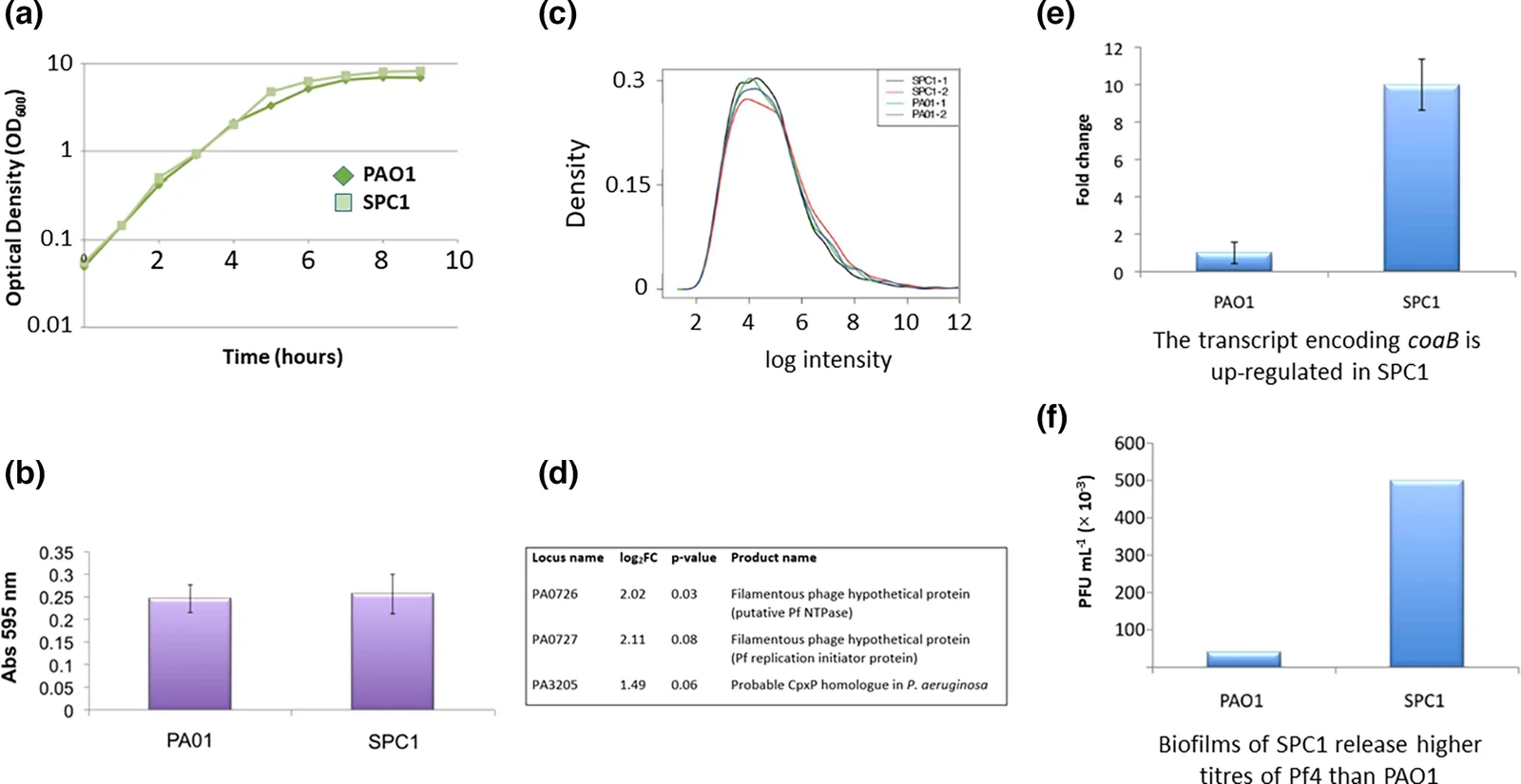
DifP suppresses Pf4 gene expression in PAO1. (**a**) The *difP* mutant (Δ*difP*::Gm; SPC1) and its WT progenitor (PAO1) show no significant differences in planktonic growth in AGSY medium. Data represent the mean±sd of three biological replicates. (**b**) SPC1 and PAO1 have similar cell attachment phenotypes. Cell attachment to the polystyrene wells of microtitre plates was assayed using crystal violet staining (*N*=8 replicates ±sd). (**c**) Density plots for Robust Micro-array Average-normalized data from the Affymetrix microarrays. Data are shown for *N*=2 microarrays for each strain. RNA was harvested for analysis from 3 day continuous flow biofilms of PAO1 and SPC1. (**d**) A list of the three modulated transcripts discussed in the body text associated with the *difP* mutation, along with their false discovery rate-adjusted log_2_ fold changes and *P*-values. (**e**) Expression of the Pf4 gene, *coaB*, is up-regulated in the *difP* mutant, SPC1. Q-RT-PCR was carried out on cDNA obtained from 3-day biofilms of each strain. The relative expression level of *coaB* in PAO1 was set as 1. The transcript of the conserved segment of the *P. aeruginosa* 16S rRNA genes was used as an internal standard. The data represent the mean±sd for three biological replicates. (**f**) Effluents from 3 day PAO1 or SPC1 biofilms (as indicated) were serially diluted and mixed with a soft agar overlay of PAO1 to enumerate Pf4 virion counts.

We next used pEX18T-mediated allele exchange to generate a mutant (SPC1) in which the PA3572 ORF was deleted and replaced with a gentamicin resistance cassette. The mutant showed normal growth (*cf.* its PAO1 progenitor) in liquid culture (Fig. 2a) and manifested no defect in cell attachment (as measured using the crystal violet assay [15], Fig. 2b).

To gain insight into the likely function of PA3572, we next carried out an Affymetrix microarray-based transcriptomic comparison of SPC1 *cf.* PAO1 biofilm cells. Following Robust Micro-array Average (RMA) normalization (Fig. 2c), just one transcript (from the Pf4-encoded ORF, PA0726) displayed significant (*P*<0.05) modulation (Fig. 2d). Two other transcripts (PA0727 and PA3205, encoding another Pf4 ORF and a homologue of the *Escherichia coli* cell envelope stress-responsive protein, CpxP, respectively) displayed modulations with *P*=0.08 and *P*=0.06, respectively (Fig. 2d). The relatively high *P*-values of these ‘hits’ are likely because only *N*=2 biological replicates were examined for each strain. However, as outlined below, these hits (and others) were followed up with independent phenotypic and Q-RT-PCR-based validation. The PA0726, PA0727 and PA3205 transcripts were up-regulated in the SPC1 *cf.* PAO1. Only one transcript (encoding PA3572 itself) was significantly down-regulated in SPC1. A complete list of the detected transcripts and their modulations is shown in Table S1. These data indicated that PA3572 normally depresses expression of the Pf4 ORFs. We, therefore, set out to investigate this possibility further.

**Fig. 3.**
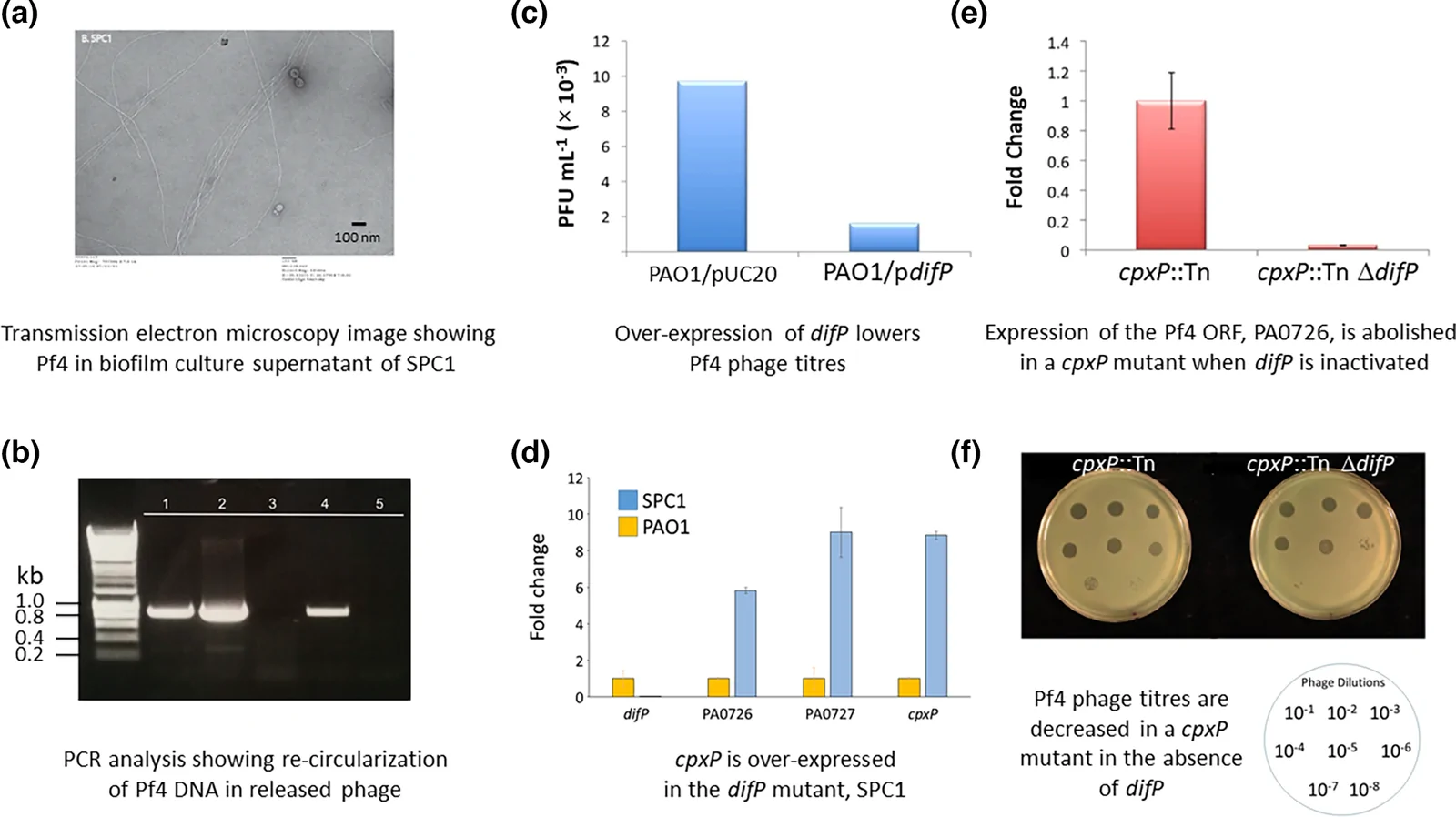
DifP-dependent suppression of Pf4 phage production is abolished in the absence of *cpxP*. (**a**) Transmission electron micrograph of an SPC1 biofilm effluent sample, showing an abundance of interacting filamentous phage particles typical of Pf4. Bar=100 nm. (**b**) The Pf4 DNA in biofilm effluents is circular (indicative of the presence of infective phage particles), whereas Pf4-encoding DNA in the PAO1 or SPC1 genome is linear. Primers were designed to read out of each end of the chromosomally-integrated Pf4 phage such that when the ends are attached to one another (as would be expected in a circularized phage genome) a PCR product of 839 bp is generated. Templates for the PCR reactions were: Lane 1; effluent from 3 day PAO1 biofilm, Lane 2; Effluent from 3 day SPC1 biofilm, Lane 3; PAO1 gDNA, Lane 4; Effluent from 3 day PAO1/pUCP20 biofilm, Lane 5; Effluent from 3 day PAO1/pUCP20(*difP*) biofilm. The pUCP20(*difP*) plasmid encodes the *difP* ORF and ca. 300 bp upstream DNA and so expresses *difP* from the endogenous *difP* promoter. (**c**) Over-expression of *difP* from pUCP20(*difP*) depresses Pf4 phage titres (measured as in Fig. 2F) in 3 day PAO1 biofilm effluents. P.f.u. data were measured using the standard plaque assay (see Supplementary Methods). (**d**) Q-RT-PCR was used to measure expression of the indicated ORFs in cDNA derived from the cells of 3 day biofilms of PAO1 or SPC1 (as indicated). Note the up-regulation of *cpxP* in the SPC1 biofilms. For each ORF, the mean expression level in PAO1 was set to 1. The transcript of a 16S rRNA gene was used as an internal standard. The data represent the mean±sd for three biological replicates. (**e**) Expression of the Pf4 ORF, PA0726, is abolished when *difP* is inactivated in a *cpxP* mutant. Q-RT-PCR was used to measure PA0726 transcript levels in RNA extracted from a *cpxP*::Tn mutant and from a *cpxP*::Tn Δ*difP*::Gm double mutant, as indicated. The transcript of a 16S rRNA gene was used as an internal standard. The data represent the mean±sd for two biological replicates. (**f**) Pf4 phage titres were measured (using the spot assay method outlined in the Supplementary Methods section) in biofilm effluents of the *cpxP*::Tn mutant and the *cpxP*::Tn Δ*difP*::Gm double mutant.

PA0726 and PA0727 are part of separate operons in the Pf4 prophage, so we next used Q-RT-PCR to examine whether an ORF (PA0723; *coaB*) encoded by a different Pf4 operon was also up-regulated in SPC1 biofilms. *CoaB* was indeed up-regulated in SPC1 *cf.* PAO1 (Fig. 2e). Collectively, these data indicated that biofilms of SPC1 release higher titres of Pf4 than the PAO1 progenitor (see also Fig. 3d). Direct titration of phage in the PAO1 and SPC1 biofilm supernatants confirmed this (Fig. 2f).

Transmission electron microscopy of SPC1 biofilm supernatants confirmed the presence of copious quantities of filamentous phage (Fig. 3a). PCR analyses indicated that these were indeed Pf4 phage containing circularized DNA (no circularization was seen when PAO1 gDNA was used as a template (Fig. 3b)). Over-expression of PA3572 from pUCP20 led to a substantial lowering of Pf4 titres (Fig. 3c). Based on these data, we hereafter designate PA3572 as *difP*.

Given its modulation in SPC1, we next examined whether the *cpxP* homologue, PA3205, might also play a role in modulating Pf4 production. The *cpx* system senses damage to the cell envelope. In *P. aeruginosa*, the *cpx* system comprises a sensor kinase (CpxS, PA3206), a cognate response regulator (CpxR, PA3204) and CpxP, which shuts down the *cpx* response once the stress has been resolved [16]. *CpxP* expression was confirmed to be elevated in SPC1 biofilms using Q-RT-PCR (Fig. 3d) – presumably, as a consequence of the increased cell envelope stress associated with the accompanying elevation of Pf4 production. Expression of the Pf4 ORF PA0726 (Fig. 3e) and Pf4 phage release (Fig. 3f) was essentially normal in a *cpxP*::Tn mutant. However, and unlike the situation in the PAO1 progenitor where inactivation of *difP* led to enhanced Pf4 gene expression and phage release (Fig. 2), inactivation of *difP* in the *cpxP*::Tn mutant led to depression of PA0726 expression (Fig. 3e) and ca. 20-fold lower Pf4 release (9×10^10^ p.f.u. released ml^−1^ *cpxP*::Tn biofilm effluent *cf.* 4×10^9^ p.f.u. released ml^−1^ in the *cpxP*::Tn Δ*difP* mutant biofilm effluent Fig. 3f).

## Concluding comments

Taken together, our data indicate that *difP* (PA3572) functions to depress Pf4 production in *P. aeruginosa*. The *difP* gene is widely conserved in pseudomonads and is flanked in most species by an MFS family transporter on one side and a Zn^2+^-dependent dehydrogenase on the other. However, in *P. aeruginosa*, the *mmsR-mmsAB*/ACS cluster of genes has inserted between *difP* and the Zn^2+^-dependent dehydrogenase. The *mmsAB*/ACS genes encode enzymes involved in branched-chain amino acid catabolism and are not found in non-*aeruginosa* pseudomonads. We note that insertion of the *mmsR-mmsAB*/ACS cluster of genes does not appear to affect the biofilm-dependent expression of *difP*.

Deletion of *difP* enhanced Pf4 production, whereas over-expression of the ORF suppressed Pf4 production. However, this suppressive effect of DifP was abolished in a *cpxP* mutant. These data suggest a relationship between cell envelope stress sensing through the *cpx* system and DifP function. In this regard, it is worth noting that most of the Pf4 regulatory factors identified to date promote Pf4 gene expression during biofilm growth, whereas DifP does the opposite. Since unfettered Pf4 production – especially given the propensity of this phage for superinfection and cell lysis – is likely detrimental to the wider PAO1 population, having such an active restraining mechanism in place makes good biological sense. Similarly, given that excessive Pf4 production is likely to damage cell envelope integrity, it is also logical that this restraining mechanism should be linked with the detection of cell envelope stress.

Given that PA3572 belongs to an entirely uncharacterized family of proteins, in the absence of biochemical data it is difficult to speculate on the mechanism by which it suppresses Pf4 gene expression. However, given its small size and chromodomain-like structure (three antiparallel β strands overlain by a C-terminal α helix), one possibility is that – like other chromodomain proteins – DifP mediates its effects via protein-protein interactions (e.g. with the H-NS-like proteins MvaT and MvaU, which are known to impinge on Pf4 gene expression). Nevertheless, we also note that chromodomain-containing proteins are also known to bind directly to DNA and RNA, so this too remains a formal possibility. Efforts to purify and characterize the binding partner(s) of DifP are currently underway.

## Supplementary material

10.1099/mic.0.001770 Supplementary Material 1.

## Abbreviations

aa: amino acid
AGSY: alanine-glycerol-salts-yeast extract medium
CF: cystic fibrosis
DifP: depresses induction of filamentous Pf4 prophage
MFS: major facilitator family
PFU: plaque forming unit
PilC: pilin protein
Q-RT-PCR: quantitative reverse transcription polymerase chain reaction
RMA: Robust Microarray Average
