# Supplementary material for: Induction of the filamentous prophage, Pf4, is conditionally regulated by PA3572 (difP) in Pseudomonas aeruginosa biofilms
Source: Microbiology (Reading). 2026 Sep 9;172(9):001770. doi: 10.1099/mic.0.001770 (PMC13583186; doi:10.1099/mic.0.001770)
Supplement: Supplementary file 1 [file mic-172-01770-s001.pdf]

Supplementary Data

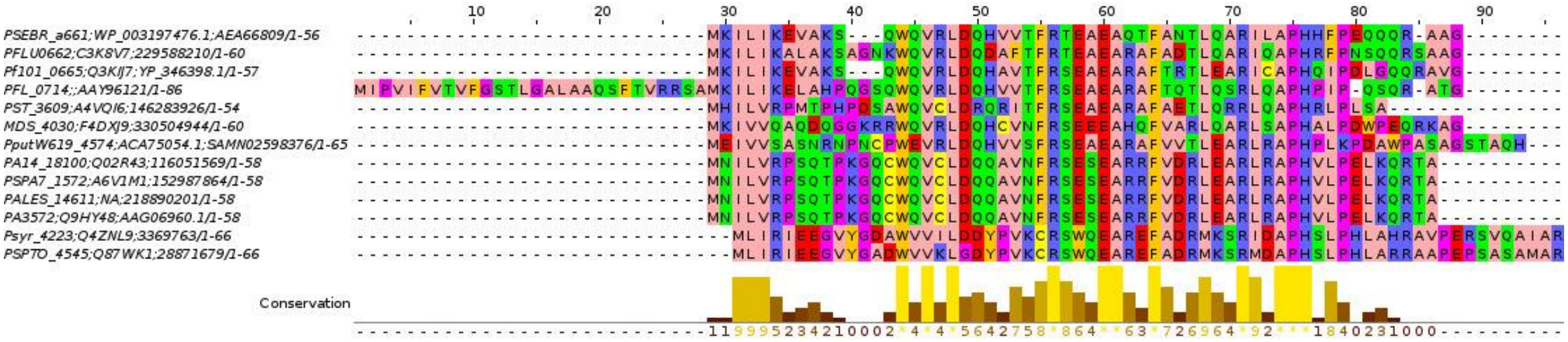

Figure S1. Amino acid sequence conservation of PA3572 in different species of Pseudomonas. Absolutely conserved residues are shown as asterisks in the conservation score, and the rest are scored numerically (e.g. 9 indicates high residue conservation). Note, the extended N-terminus of the predicted protein (PFL_0714) in *Pseudomonas protegens* strain Pf5 is likely due to an auto-annotation error, since the probable correct start codon (beginning with sequence MKIL…) is preceeded by a near perfect Shine-Dalgarno sequence (AGGAGGTX_5ATG…). Key to species: PSEBR: *Pseudomonas brassicacearum* subsp. *brassicacearum* NFM421, PFLU: *Pseudomonas fluorescens* SBW25, Pfl01: *Pseudomonas fluorescens* Pf0-1, PFL: *Pseudomonas protegens* Pf5, PST: *Pseudomonas stutzeri* A1501, MDS: *Pseudomonas mendocina* NK-01, Pputw619: *Pseudomonas putida* W619, PA14: *Pseudomonas aeruginosa* PA14 (clinical isolate), PSPA7: *Pseudomonas aeruginosa* PA7 (taxonomic outlier), PALES: *Pseudomonas aeruginosa* (Liverpool epidemic strain), PA3572: *Pseudomonas aeruginosa* PAO1 (this study), Psyr: *Pseudomonas syringae* pathovar *syringae* B728a, PSPTO: *Pseudomonas syringae* pathovar *tomato* D3000.

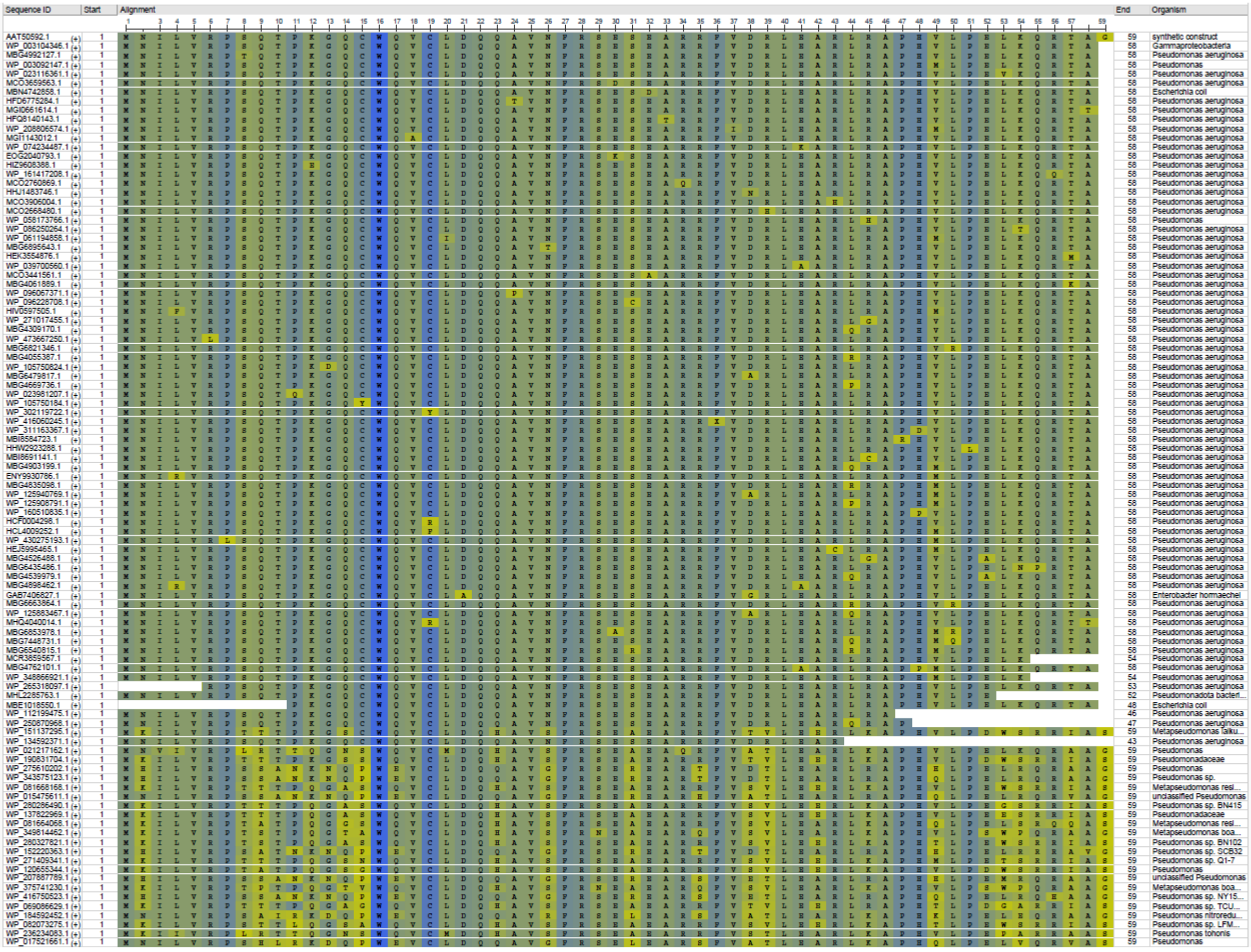

Figure S2. Output from the <u>CO</u>nstraint-<u>B</u>ased multiple sequence <u>AL</u>ignment <u>T</u>ool (COBALT) showing the PA3572 amino acid sequence aligned with 100 top hits from the non-redundant protein sequences database hosted at NCBI. The colouring is based on the BLOSUM45 matrix. Note the absolute conservation of Trp16 and the very high level of conservation of Cys19 (PA3572 numbering), possibly indicating that these residues are of functional importance.

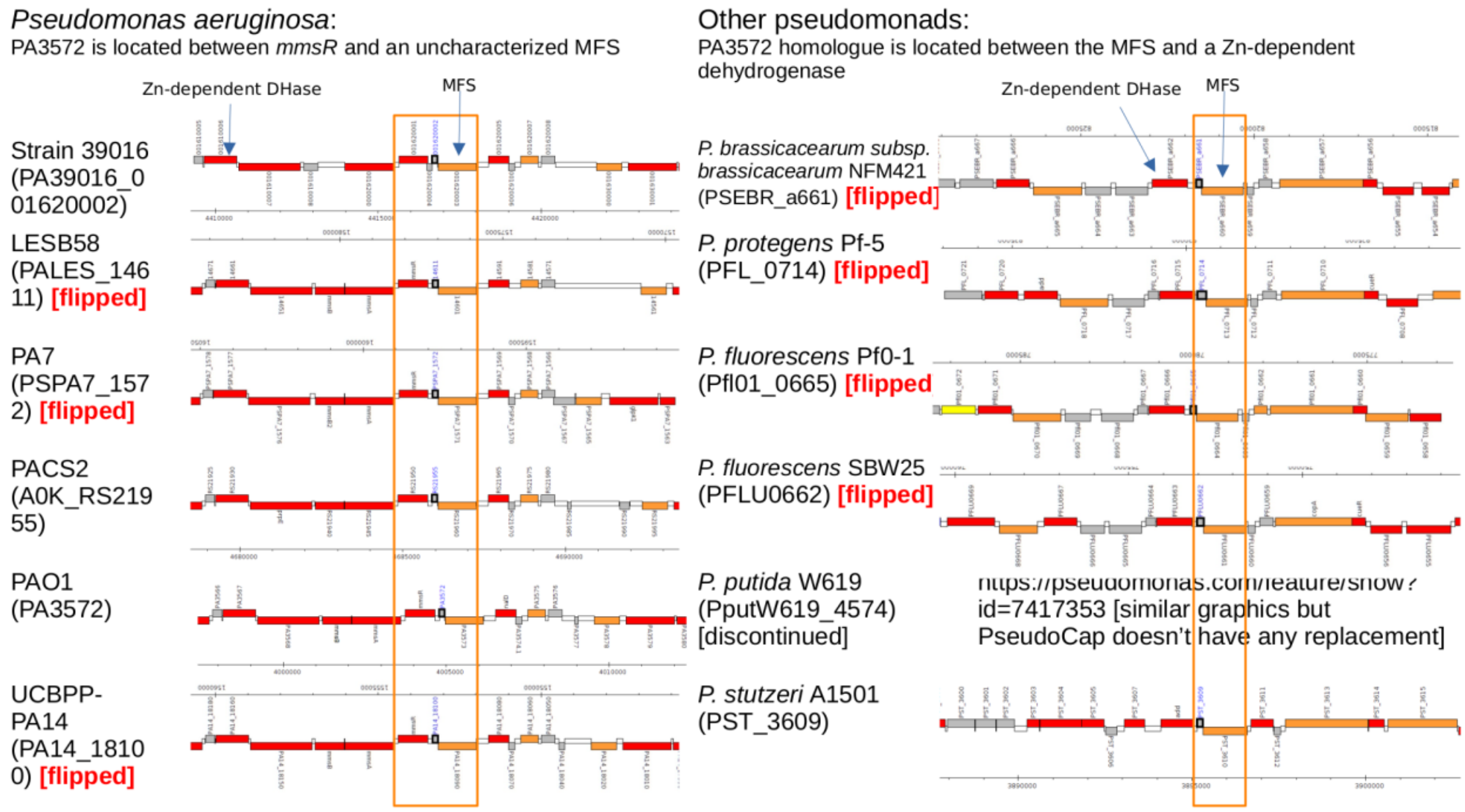

Figure S3. The *mmsR-mmsAB*/ACS gene cluster appears to have inserted between PA3572 and a Zn-dependent dehydrogenase (corresponding to PA3567 in *P. aeruginosa* strain PAO1) in *Pseudomonas aeruginosa*. Other pseudomonads do not encode the *mmsR-mmsAB*/ACS gene cluster adjacent to the PA3572 homologue (or elsewhere in the chromosome).

Supplementary Methods

Planktonic cultures were inoculated to an OD_{600} = 0.05 and grown in 75 mL AGSY (alanine-glycerol-salts-yeast extract) medium in 500 mL baffled flasks. When required, the medium was supplemented with antibiotics. Typically, flasks were incubated at 37°C with vigorous aeration (shaking at 300 rpm). For planktonic microaerophilic growth, non-baffled flasks contained 75 mL AGSY overlayed with 50 mL sterile mineral oil. Cultures were inoculated to an OD_{600} = 0.05 and incubated at 37°C with gentle shaking (50 rpm).

The continuous-flow biofilms were set-up based on the protocol described in Mikkelsen *et al.* (1) with minor modifications. **Figure S4** represents a schematic drawing of the biofilm set-up. Silicon tubing (Versilic, Fischer Scientific) was used throughout except for the section inside the peristaltic pump, where stronger marprene tubing (Watson Marlow) was used. All fittings were purchased from the Cole-Parmer Instrumental Company. The system was assembled as shown in **Figure S4**. The free ends of the tubing were enveloped in cotton wool and covered in aluminium foil before being placed in a sealed autoclave bag. The contents were then sterilized by autoclaving. The AGSY medium (minus salts) was made in 5-10 L bottles fitted with silicone tubing and foam bungs at the mouth. The tops of the bottles were covered with aluminium foil and autoclaved. During assembly of the system, the fittings were soaked in ethanol to prevent contamination. Salts were added to the medium under

sterile conditions immediately prior to use (to prevent precipitation). After assembly, the tubes and pump were filled with medium and the flow rate was adjusted to 50 mL h^{-1}. Prior to inoculation, the 3 mm tubes upstream of the 6 mm tubes were clamped off to prevent any backflow of the inoculum. Each tube was inoculated with 4×10^8 colony forming units. Cells were allowed to attach for 1 h before the flow of media was restarted. The flow rate of the medium was monitored daily by measuring the volume of effluent collected for each channel and minor adjustments were made as necessary. In order to avoid sedimentation of the cells, the 6 mm tubes in which the biofilms were allowed to form were rested vertically. When samples were harvested, the tube upstream of the biofilm was clamped, and the attached biomass on the inside of the 6 mm tube was extruded into a suitable receptacle on ice. Samples for RNA extraction were squeezed directly into RNAlater on ice. Effluents were collected daily for Pf4 phage plaque assays.

1, Mikkelsen H, Duck Z, Lilley KS, Welch M. Interrelationships between colonies, biofilms, and planktonic cells of Pseudomonas aeruginosa. J Bacteriol. 2007 Mar;189(6):2411-6. doi: 10.1128/JB.01687-06. Epub 2007 Jan 12. PMID: 17220232; PMCID: PMC1899361.

Pf4 plaque assays. PAO1 prophages were isolated from the run-off effluent collected from continuous-flow biofilms. The samples were sedimented (10,000 × *g*, 20°C, 10 min) to pellet any planktonic cells and the supernatant was filter sterilized using a 0.45 µm filter (Millipore). Phage content was measured in the samples using one of two approaches. In the “standard plaque assay”, serial dilutions (100 µL volume) of the sample were mixed with 3 mL of soft LB-agar (0.3%, held at 50°C, just above the gelling temperature) containing 50 µL overnight culture of PAO1. The mixture was then promptly poured as an overlay onto 25 mL solidified 1.5% LB-agar. The resulting plates were then incubated overnight and plaques were scored the following day. In the second “spot assay” approach, serial dilutions (10 µL volume) of the filtered supernatant were spotted onto top lawns of LB-agar seeded with 100 µL overnight culture of PAO1. Plates were incubated overnight at 37°C and discrete clearings on the bacterial lawn were counted as one plaque.

Assay for the replicative (circularized) Pf4 genome. The replicative form of Pf4 was assessed by PCR using primers (shown 5’→3’) AGCAGCGCGATGAAGCAAT and TAGAGGCCATTTGTGACTGGA. These primers yield an 839 bp product if the phage genome is a closed circle of DNA, but yield no product from the linear form of the phage genome (i.e., when the phage DNA is chromosomally integrated into the host genome).

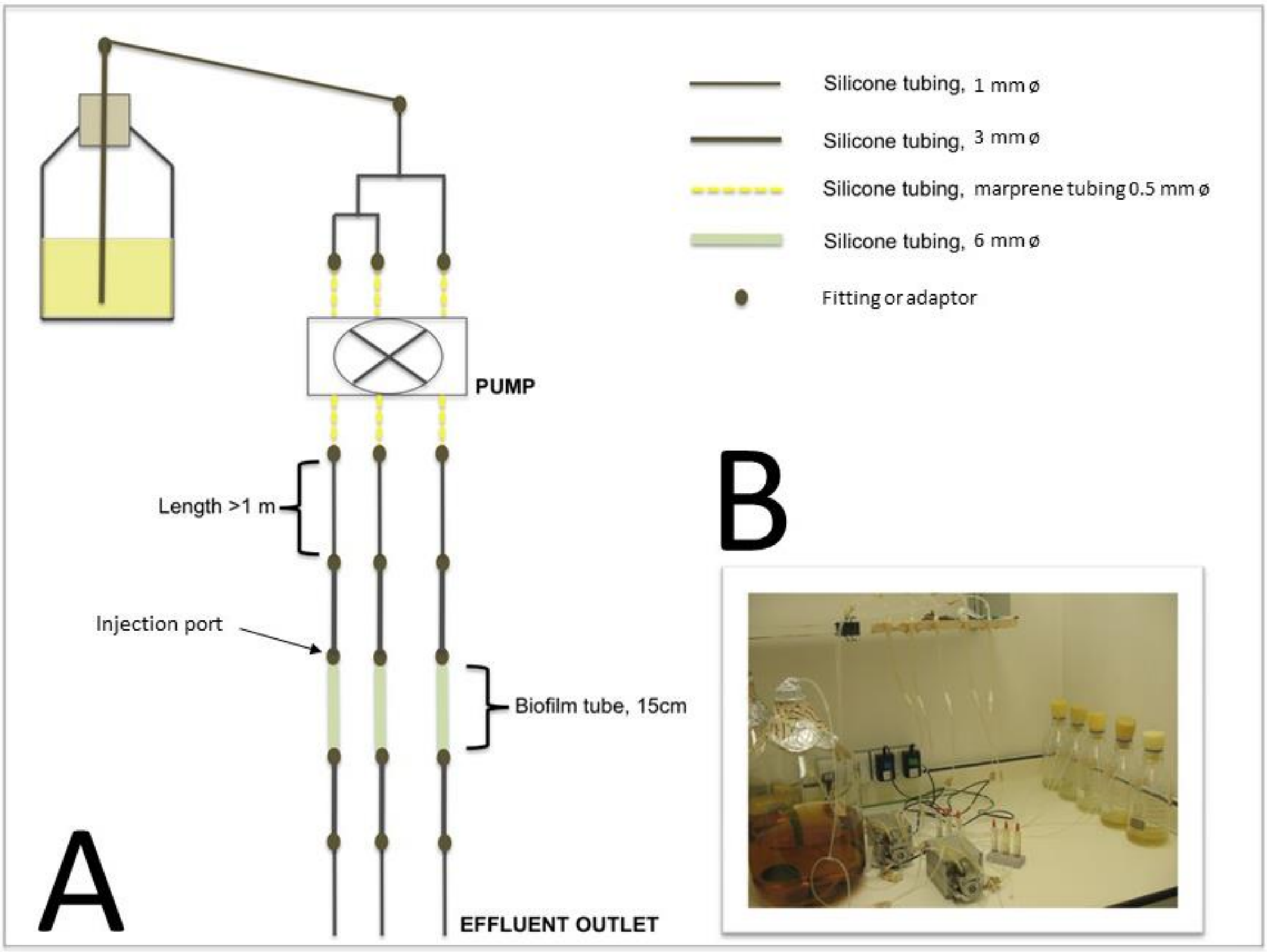

Figure S4 – Schematic of the continuous flow biofilm setup. (A) The media bottle was sealed with a foam bung and aluminium foil, and sterilized separately from the tubing. Salts were added to the media in a laminar flow hood just before use. Media was pumped through the system at a constant rate of 50 mL h^{-1} using a sealed-loop peristaltic pump. Inoculation was carried out using a sterile syringe inserted at the indicated injection port. (B) Picture showing the continuous-flow biofilm system setup *in situ* (housed in a warm room at 37°C).

The *difP-lacZ* chromosomal promotor-*lacZ* fusion strain was constructed using the mini-CTX-*lacZ* system (Hoang *et al*, 2). The upstream region (275 bp) of *difP* was PCR amplified using primers (shown 5'→3') TTTTGGATCCGGACAGCAGCGACCAGAG and TTTTGAATTCGAGGATGTTCATGGCGATTC, which incorporated EcoRI and BamHI restriction sites at the ends of the amplicon. Following digestion with these enzymes, the promoter fragment was cloned into similarly digested mini-CTX-*lacZ*. The construct was then introduced into *Escherichia coli* β2163 by transformation. Finally, the construct was transferred by conjugal biparental mating into PAO1 and SPC1 (the Δ*difP*::Gm mutant). Mini-CTX-*lacZ* harbors the ΦCTX integrase, allowing integration of the entire construct at the *attB* site on the PAO1 chromosome. The mini-CTX plasmid backbone was then excised using the Flp-FRT recombinase

(Hoang *et al*, 3) to generate a markerless chromosomal *difP* promoter-*lacZ* fusion in a wild type (PAO1) and Δ*difP*::Gm mutant background. The presence of a *difP* promoter-*lacZ* fusion integrated at the *attB* site in each strain was confirmed by PCR using primers CGAGTGGTTTAAGGCAACGGTCTTGA and AGTTCGGCCTGGTGGAACAACTCG.

2, Hoang TT, Karkhoff-Schweizer RR, Kutchma AJ, Schweizer HP. A broad-host-range Flp-FRT recombination system for site-specific excision of chromosomally-located DNA sequences: application for isolation of unmarked Pseudomonas aeruginosa mutants. Gene. 1998 May 28;212(1):77-86. doi: 10.1016/s0378-1119(98)00130-9. PMID: 9661666.

3, Hoang TT, Kutchma AJ, Becher A, Schweizer HP. Integration-proficient plasmids for Pseudomonas aeruginosa: site-specific integration and use for engineering of reporter and expression strains. Plasmid. 2000 Jan;43(1):59-72. doi: 10.1006/plas.1999.1441. PMID: 10610820.

β-galactosidase activity measurement. Cells were pelleted from 1 mL of culture harvested from exponential (4 h) or stationary phase (9 h) planktonic growth, or from 3-day continuous flow biofilms. The cell pellets were resuspended in 1 mL Z buffer (60 mM Na_2HPO_4, 40 mM NaH_2PO_4, 10 mM KCl, 2 mM $MgSO_4$, 0.3% v/v β-mercaptoethanol) and 50 µL of toluene was added. The samples were then vortexed vigorously for 30 s. The resulting mix was chilled on ice for 20 min and 700 µL of the cell lysate was removed from under the toluene layer. Aliquots (50 µL) of this toluenised cell extract were further diluted in 450 µL Z buffer and equilibrated at 37°C for 2-3 min. Subsequently, 100 µL pre-heated ONPG (*O*-nitrophenyl β-D-galactoside; 4 mg mL^{-1}) was added to the mixture and the rate of colour development was monitored at 420 nm. The data are expressed in Miller units: A_{420} min^{-1} per OD_{600} of culture sample.

Quantitative reverse transcription PCR (qRT-PCR) was used to measure mRNA levels, and hence changes in expression. Oligonucleotides (all listed 5'→3') used as primers for quantitative PCR were: *difP* (AAACCCCGAAAGGCCAAT and GCCGTTCGCTGTTTCAGTT), *cpxP* (TGAAGGAACTGGACCTGAC and TCCTTCTGGGTCTTCTCG), PA0726 (CAACTGCTGTTTCCCAAAT and CGCGGATGTAGGAGATGT), and PA0727 (AGAACGCTGAAATGGGTCT and TGGTTTCGCAGTGACTGA). Total RNA was collected as described below for the microarray analyses. The cDNAs were synthesized from total RNA by reverse transcription using random hexamer priming. Mixtures lacking reverse transcriptase (RT) served as a control for DNA contamination. A 5 µL aliquot of template cDNA was used in each 25 µL PCR reaction. The PCR reaction mixtures also contained 1 × SYBR Green PCR Master Mix (Applied Biosystems), and 10 pmol of the indicated primers. PCR amplification was done using an ABI PRISM 7300 real-time PCR system. Amplification was accomplished using 40 cycles of 95°C (15 s) and 60°C (1 min). Fluorescence

data were processed using SDS software (ABI) to produce threshold cycle values for each sample. Relative gene expression determined against a 16S rRNA control, with the mRNA/16S rRNA ratio arbitrarily set as 1 in the wild type (PAO1) samples. All measurements were made in triplicate.

Construction of SPC1 (Δ*difP*::Gm). The *difP* ORF and ca. 500 bp flanking DNA was PCR amplified from PAO1 gDNA template using primers (listed 5'→3') TTTTTTGAATTCGCTACCGCTTCCAGCACTT and TTTTTTGGATCCGTTCGCCCTACGTGTTCATC. After digestion with BamHI and EcoRI, the fragment was sub-cloned into pUCP20. Next, the resulting plasmid was used as a template for PCR using primers TTTTTCTCGAGATTCATCCATCGGGGCTG and TTTTTACTAGTAGACGGCGATCACCAGAGT, which read out from the ends of the cloned *difP* ORF, amplifying the whole plasmid minus *difP* (but including the *difP* flanking DNA). The resulting 4.9 kb frgament was digested with SpeI and XhoI. In parallel, the Gm^R cassette from plasposon pTnMod-O-Gm was excised and ligated to the 4.9 kb fragment. This yielded a construct in which the *difP* ORF was precisely deleted and replaced with a Gm^R cassette. Next, primers TTTTTTGAATTCGCTACCGCTTCCAGCACTT and TTTTTTGGATCCGTTCGCCCTACGTGTTCATC were used to amplify the gentamicin cassette and ca. 500 bp flanking regions of *difP*. The fragment was then digested with BamHI and EcoRI and ligated to similarly digested pEX18Tc. The pEX18Tc-derived marker exchange construct (pEX18Tc (Δ*difP*::Gm)) was maintained in the *E. coli* helper strain, β2163. The pEX18Tc (Δ*difP*::Gm) was introduced into PAO1 following bi-parental conjugation with the β2163/pEX18Tc (Δ*difP*::Gm). The mating patch was resuspended in a aliquot of LB, which was plated on to gentamicin (50 $\mu g\ mL^{-1}$) to select for co-integrants. The next day, a single co-integrant colony was used to inoculate 5 mL LB, and incubated overnight. Aliquots of the overnight culture were then plated on 10% sucrose-LBA containing gentamicin. A selection of Gm^R colonies were confirmed by PCR amplification across the junctions flanking the Δ*difP*::Gm insert and subsequent DNA sequencing of the products. Where indicated, the Δ*difP*::Gm mutation was also generated into the *cpxP*::Tn mutant, phoAwp08q1G01. This mutant contains an IS*phoA*/hah insertion in the forward orientation at position 391 (out of 441) in the *cpxP* ORF (PA3205). The Tn insertion in this mutant was confirmed using primers (5'→ 3') TACTACAGCCACTGAGCGGG and ATCCAGGTGTCCTGGTTCAG. In the absence of a Tn insertion, the amplicon from these primers is 804 bp in length, and this was seen when PAO1 gDNA was used as a template, but not when gDNA from the Δ*difP*::Gm *cpxP*::Tn mutant or the *cpxP*::Tn mutant was used as a template.

Over-expression of *difP*. The *difP* ORF was constitutively expressed from pUCP20 using the *lac* promoter on the plasmid to drive gene expression. The *difP* ORF along with 76 bp upstream DNA (inlcuding the native ribosome binding site) and 94 bp of downstream DNA was PCR-amplified using primers (listed 5'→3') TTTTTTGAATTCCTGATCCCGGTCAGTGTTTT and

TTTTTTGGATCCCCCATGACTCTGGTGATCG. The PCR product was digested with EcoRI and BamHI, and ligated to similarly digested pUCP20 to yield p*difP*.

Attachment assays. These were carried out in polystyrene flat-bottomed 96-well plates (Nunc). Eight technical and three biological replicates of each strain were performed. AGSY or LB media was inoculated to an OD_{600} = 1 from an overnight culture. Aliquots (200 µL) were dispensed into each well. The plates were incubated (without their lids) with good aeration in a sealed/sterile humidified chamber at 37°C for 18 h. Following this, the liquid culture was gently aspirated and the wells were washed with 200 µL of sterile water. The attached cells were stained with 250 µL of 0.1% (w/v) crystal violet and left for 1 h. Excess stain was removed by washing the wells (3 ×) with water. Finally, the stain attached to the biomass was leached with 250 µL of 50% w/v ethanol for 1 h. The released stain was quantified by measuring A_{595} in a Biorad plate reader.

Electron microscopy. The phage lysate was prepared by collecting the effluent from 3-day-old biofilms. The cells in the sample were pelleted by centrifugation and the supernatant was filter sterilized using 0.22 µm filters (Millipore). An aliquot (4 mL) of the supernatant was sedimented in a Beckman MLS-50 (TL-100) benchtop ultracentrifuge at 50,000 × *g*, for 1 h at 4°C. The resulting pellet was resuspended in 4 mL of 50 mM Tris-HCl, 8 mM $MgSO_4$, 0.2 M NaCl and 0.1 g gelatine (pH 7.4). The resuspended lysate was sedimented a further two times as before. The resulting gelatinous pellet was resuspended in 50 µL of the same buffer ready for electron microscopy. This was carried out at the Multi-Imaging Centre (University of Cambridge) using an FEI Philips CM100 transmission electron microscope. Carbon-coated Formvar discharged copper grids (400 mesh) were placed in five microliters of phage lysate for 45 s then washed three times in 5 µL water. Phage samples were subsequently stained with 1% uranyl acetate for 45 s before microscopy. The grids were then blotted onto filter paper to remove excess liquid and allowed to air-dry.

Microarray analyses. Cells were grown in AGSY medium at 37°C as continuous-flow biofilms for 3 days or as planktonic cultures (harvested during stationary phase (ca. 9 h growth)). The planktonic cultures (0.5 mL) were injected directly into ice-cold RNAlater whereas the biofilm cells were squeezed out of the tubes directly into RNAlater and stored at 4°C overnight to ensure complete penetration. After this, both sample types were stored at -80°C. Prior to extraction, a suitable volume of the culture/RNAlater mixture was sedimented (5 min, 4°C, 13,000 rpm in an Eppendorf centrifuge) and the pellet was resuspended in 1 mg mL^{-1} lysozyme solution. After 13 min at room temperature, the RNA was extracted using an RNEasy Mini Purification Kit (Qiagen). RNA was eluted in 55 µL RNase free water and stored at -80°C. For RNA extractions of planktonic stationary cultures, five separate extractions were performed for each biological replicate, and the extracted RNA was pooled and precipitated. Precipitation was accomplished by adding 1/10 volume sodium acetate (3 M) followed

by 2.5 volumes of 100% ethanol. The samples were then left overnight at -80°C. The precipitates were then pelleted (5 min, 15,000 x *g*, 4°C), dried, and resuspended in RNase free water. Where necessary, additional DNaseI treatment was required to eliminate DNA contamination in samples grown to a high optical density. This was done by adding 80 µL of DNaseI working solution directly to the RNA samples and incubating for 30 min at room temperature. The RNA was re-purified using the RNA cleanup protocol (Qiagen). After purification, RNA concentration was determined using a Nanodrop ND-1000 Spectrophotometer (Labtech International) and RNA quality was checked on a 1.2% (w/v) agarose gel, where bands of 23S and 16S RNA were clearly visible. Final concentrations of RNA were between 0.9 and 2 mg mL^{-1}. The procedure above was also followed for the RNA samples used in the qRT-PCR analysis.

cDNA synthesis was carried out in two steps. First, random hexamer primers (Invitrogen) were annealed to the RNA. Reaction volumes were 30 µL and contained 0.33 µg μL^{-1} total RNA and 25 ng μL^{-1} random hexamers. Mixtures were incubated at 98°C for 3 min, followed by 10 min at 70°C and 10 min at 25°C. This was followed by the cDNA synthesis reaction. Reaction mixtures (60 µL) contained 1 × reaction buffer (Thermo), 10 mM DTT, 0.5 mM dNTP mixture, 30 U SUPERase-In, and 1500 U Superscript II. Reactions were incubated at 25°C for 10 min, 37°C for 60 min, 55°C 180 min, then 70°C for 10 min. RNA was removed by adding 20 µL 1 M NaOH to each sample and incubating at 65°C for 30 min. After cooling to 4°C, the samples were neutralized by addition of 20 µL 1 M HCl. cDNA was purified using the MinElute PCR purification kit (Qiagen), following the manufacturer's instructions. Final cDNA concentrations were in the range of 100 and 130 µg mL^{-1}. The cDNA concentration was measured using a Nanodrop ND-1000 Spectrophotometer (Labtech International). The A_{260}/A_{280} ratio was close to 1.8. The cDNA was then fragmented using 0.2 U $(\mu g\ cDNA)^{-1}$ RQ1 RNase-free DNase (Promega) for 10 min, followed by heat inactivation of the DNase at 98°C (10 min). The fragmentated cDNA was stored until needed at -20°C.

Sample labelling for microarray. Reaction mixtures (70 µL) for microarray analysis were prepared using the GeneChip DNA labeling reagent (Affymetrix). Briefly, the fragmented cDNA (50 µL) was mixed with 14 µL 5 × reaction buffer, 3 µL terminal deoxynucleotidyl transferase, and 2 µL GeneChip labelling reagent (Affymetrix). The samples were incubated for 1 h, and the reaction was stopped by adding 0.5 M EDTA (2 µL).

Hybridization. Affymetrix arrays were equilibrated at room temperature for 15 min. The arrays were then mixed with hybridization cocktail (0.5 – 7.0 µg fragmented/labelled cDNA, 50 pM control oligonucleotide, 1 × hydridization buffer (Affymetrix), 7.8% DMSO, and water to a final volume iof 130 µL) and the samples were incubated, rotating at 60 rpm, in a hybridization oven at 50°C for 18 h. Washing and staining was performed on an Affymetrix Fluidics Station 450 using the manufacturer's

recommended buffers/developing reagents. The arrays were scanned using a GeneChip Scanner 3000 (Affymetrix). The data were processed using the open source bioinformatics software, R 2.12.1 (r-project.org) and quality checks, normalization and statistical analyses were carried out using the BioConductor packages affyPLM, GCRMA and LIMMA (bioconductor.org).

Table S1

Microarray data comparing the transcriptome of SPC1 (the Δ*difP*::Gm mutant) with that of the PAO1 progenitor.

**ProbeID**	**intensity PA3572_ 1**	**intensity PA3572_2**	**intensity PA01_1**	**intensity PA01_2**	**FDR PA3572-PA01 log_2FC**	**FDR PA3572-PA01 adj.p value**
PA0726_at	10.00	10.13	7.87	8.21	2.02	0.03
PA3572_at	2.42	2.35	5.59	6.44	-3.63	0.03
PA3205_at	7.95	7.73	6.31	6.39	1.49	0.06
PA0727_at	9.35	9.90	7.66	7.38	2.11	0.08
PA2782_at	4.29	4.26	5.32	5.42	-1.10	0.13
PA0971_tolA_at	6.87	6.81	5.93	5.94	0.90	0.22
PA2783_at	4.63	4.81	5.76	6.09	-1.21	0.22
PA5528_at	5.46	5.57	4.52	4.61	0.95	0.22
PA0621_at	7.03	7.06	6.17	6.24	0.84	0.25
PA0723_coaB_at	11.80	12.25	10.77	10.80	1.24	0.25
PA0852_cpbD_at	5.45	5.43	6.24	6.42	-0.89	0.25
PA1930_at	3.53	3.53	4.33	4.53	-0.90	0.25
PA0807_at	6.47	6.35	5.58	5.32	0.96	0.27
PA4141_at	6.48	6.90	7.76	7.93	-1.15	0.27
PA3328_at	4.10	4.18	4.92	4.89	-0.77	0.31
PA0620_at	5.27	5.24	4.52	4.55	0.72	0.38
PA0635_at	5.33	5.18	4.41	4.55	0.78	0.38
PA0725_at	5.31	5.97	4.20	4.44	1.32	0.38
PA0728_at	6.72	7.39	5.83	5.37	1.45	0.38
PA1341_at	4.46	4.45	5.09	5.23	-0.71	0.38
PA1905_s_at	5.16	5.29	5.90	6.10	-0.78	0.38
PA2024_at	5.16	5.42	6.13	6.11	-0.83	0.38
PA2618_at	3.80	3.58	4.48	4.46	-0.78	0.38
PA2656_at	5.30	5.17	4.56	4.52	0.69	0.38
PA5130_at	6.49	6.41	5.48	5.77	0.83	0.38
PA0122_at	4.24	4.47	5.03	5.12	-0.72	0.46
PA0622_at	7.50	7.93	6.66	6.89	0.94	0.46
PA0625_at	5.78	5.74	5.17	4.99	0.68	0.46
PA0724_at	6.50	7.21	5.44	5.74	1.27	0.46
PA1901_s_at	7.60	7.90	8.49	8.48	-0.74	0.46
PA2331_at	4.61	4.59	5.38	5.17	-0.68	0.46

PA2755_eco_at	5.18	5.20	4.62	4.51	0.62	0.46
PA3478_rhlB_at	2.96	3.23	3.78	4.02	-0.81	0.46
PA0510_at	6.34	6.22	6.89	6.86	-0.60	0.48
PA0628_at	5.90	6.20	5.35	5.37	0.69	0.48
PA1151_imm2_at	6.30	5.70	4.95	4.98	1.04	0.48
PA1903_s_at	4.94	4.91	5.45	5.61	-0.60	0.48
PA3479_rhlA_at	4.85	4.88	5.78	5.43	-0.74	0.48
PA3723_at	5.76	6.32	6.96	7.29	-1.08	0.48
PA3841_exoS_at	7.15	7.59	8.18	8.20	-0.82	0.48
PA4525_pilA_at	5.50	6.32	7.12	7.29	-1.29	0.48
PA4607_at	6.85	6.93	7.48	7.47	-0.58	0.48
PA4765_omlA_at	7.97	7.48	6.56	6.89	1.00	0.48
PA4921_at	5.03	5.28	4.53	4.33	0.73	0.48
Pae_5SrRNA_s_at	6.77	7.10	7.67	8.13	-0.96	0.48
PA3756_at	6.09	6.10	5.48	5.57	0.57	0.48
PA4211_g_at	5.79	6.24	6.77	7.10	-0.92	0.48
PA4739_at	4.87	4.90	4.34	4.00	0.71	0.48
PA0743_at	3.54	3.63	4.21	4.10	-0.57	0.49
PA1342_at	6.51	6.84	7.32	7.47	-0.72	0.49
PA1871_lasA_at	4.83	4.62	5.29	5.56	-0.70	0.49
PA2128_at	4.97	4.97	5.52	5.51	-0.54	0.49
PA4154_at	4.58	4.92	4.09	3.78	0.81	0.49
PA4480_mreC_at	7.04	7.57	6.45	6.40	0.88	0.49
PA0617_at	5.71	5.67	5.15	5.18	0.52	0.49
PA0623_at	7.80	8.19	7.22	7.35	0.71	0.49
PA0636_at	6.79	7.23	6.30	6.14	0.79	0.49
PA0638_at	4.30	4.68	3.78	3.78	0.71	0.49
PA1337_ansB_at	5.58	5.29	6.08	6.02	-0.62	0.49
PA2366_at	3.57	3.80	4.47	4.21	-0.66	0.49
PA3186_oprB_s_at	4.41	4.48	5.01	4.94	-0.53	0.49
PA3330_at	4.33	4.40	4.86	5.06	-0.59	0.49
PA3331_at	5.20	5.30	5.75	6.08	-0.66	0.49
PA3418_ldh_at	8.27	8.27	8.75	8.88	-0.54	0.49
PA3552_at	3.49	3.44	2.98	2.73	0.61	0.49
PA3569_mmsB_at	4.02	4.89	3.30	3.17	1.22	0.49
PA3570_mmsA_at	5.25	5.50	4.83	4.65	0.63	0.49
PA3719_at	6.28	6.47	5.84	5.77	0.57	0.49
PA4236_katA_at	5.40	5.68	6.13	6.46	-0.75	0.49
PA4763_recN_at	5.17	5.72	4.56	4.65	0.84	0.49
PA5200_ompR_at	4.34	4.89	3.69	3.83	0.85	0.49
PA5276_lppL_i_at	7.15	6.57	5.60	6.03	1.05	0.49

PA5436_at	5.83	5.88	5.33	5.36	0.51	0.49
PA4217_at	5.65	6.10	6.55	6.87	-0.84	0.49
PA0459_at	5.47	5.74	6.18	6.23	-0.59	0.49
PA0907_at	3.67	3.59	3.16	2.85	0.63	0.49
PA0922_at	6.89	7.32	6.42	6.38	0.71	0.49
PA2023_galU_at	5.17	5.54	4.49	4.75	0.74	0.49
PA2195_hcnC_at	5.52	5.48	5.95	6.23	-0.59	0.49
PA2247_bkdA1_at	6.72	6.72	7.26	7.19	-0.50	0.49
PA3790_oprC_at	4.60	5.13	5.64	6.29	-1.09	0.49
PA4315_mvaT_at	9.85	9.78	9.30	9.33	0.50	0.49
PA4642_at	5.22	4.87	4.12	4.46	0.75	0.49
PA5030_at	5.17	5.29	4.78	4.58	0.55	0.52
PA2114_at	2.67	2.66	3.20	3.11	-0.49	0.53
PA3517_at	3.09	2.86	3.47	3.68	-0.60	0.53
PA2985_at	4.00	3.91	3.51	3.22	0.59	0.53
PA0069_at	3.67	3.44	3.06	2.97	0.54	0.55
PA0999_fabH1_at	5.48	5.90	6.28	6.59	-0.75	0.55
PA2193_hcnA_at	3.73	3.68	4.13	4.27	-0.50	0.55
PA2253_ansA_at	3.39	3.27	2.86	2.82	0.49	0.55
PA3417_at	6.01	6.21	6.60	6.69	-0.53	0.55
PA4556_pilE_at	4.37	4.49	4.86	5.05	-0.53	0.55
PA0400_at	3.36	3.66	4.03	4.19	-0.59	0.56
PA0643_at	5.56	5.23	4.65	4.88	0.63	0.56
PA1812_mltD_at	5.84	6.08	5.48	5.25	0.59	0.56
PA4443_cysD_at	3.23	3.17	3.61	3.88	-0.55	0.56
PA0291_oprE_at	4.86	5.07	5.41	5.55	-0.52	0.57
PA0611_prtR_at	6.18	6.23	5.72	5.77	0.46	0.57
PA0641_at	5.69	5.44	5.07	5.01	0.53	0.57
PA0647_at	5.21	5.11	4.71	4.66	0.47	0.57
PA1340_at	3.19	3.48	4.14	3.82	-0.64	0.57
PA1789_at	6.81	7.31	7.68	7.93	-0.75	0.57
PA2619_infA_at	6.05	5.80	5.45	5.30	0.55	0.57
PA2646_nuoK_at	5.89	6.30	6.70	6.75	-0.63	0.57
PA3332_at	5.23	5.23	5.63	5.92	-0.55	0.57
PA3496_at	6.37	6.46	6.01	5.74	0.54	0.57
PA3568_at	3.71	4.25	3.33	3.05	0.79	0.57
PA3990_at	4.48	4.33	3.98	3.83	0.50	0.57
PA4042_xseB_at	3.57	3.42	2.64	3.05	0.65	0.57
PA4922_azu_at	8.99	9.04	9.46	9.48	-0.45	0.57
PA5173_arcC_at	7.07	7.64	8.03	8.29	-0.80	0.57
PA5562_spoOJ_at	5.36	5.74	4.86	5.00	0.61	0.57
PA3724_lasB_at	3.82	3.66	4.20	4.23	-0.47	0.57
PA0572_at	3.75	3.59	4.09	4.45	-0.60	0.58

PA1377_at	4.57	4.67	4.22	4.07	0.47	0.58
PA5203_gshA_at	3.97	4.15	3.35	3.64	0.57	0.58
PA3533_at	5.51	5.65	4.88	5.18	0.55	0.58
PA0619_at	6.89	7.10	6.34	6.57	0.54	0.59
PA2194_hcnB_at	7.54	7.35	7.87	7.98	-0.48	0.59
PA2966_acpP_at	7.20	7.27	6.83	6.74	0.45	0.59
PA3413_at	7.89	8.21	7.52	7.49	0.54	0.59
PA3827_at	3.02	3.15	2.55	2.68	0.47	0.59
PA3284_at	3.80	4.36	3.45	2.99	0.86	0.60
PA3621_fdxA_at	3.73	3.90	3.34	3.35	0.46	0.60
PA1557_at	6.10	6.29	6.75	6.62	-0.49	0.61
PA0722_at	12.06	12.21	11.70	11.69	0.44	0.62
PA0872_phhA_at	5.81	6.56	6.99	7.11	-0.86	0.62
PA1476_ccmB_at	4.23	4.09	3.72	3.71	0.44	0.62
PA2642_nuoG_at	5.03	5.11	5.47	5.54	-0.44	0.62
PA2781_at	5.54	6.12	6.61	6.48	-0.72	0.62
PA3488_at	4.00	3.80	4.30	4.48	-0.49	0.62
PA4134_i_at	5.75	6.03	6.48	6.34	-0.52	0.62
PA4535_at	4.26	4.38	3.87	3.90	0.44	0.62
PA4587_ccpR_at	6.59	7.19	7.55	7.71	-0.74	0.62
PA4620_at	3.35	3.28	2.91	2.84	0.44	0.62
PA5317_at	3.54	3.44	3.92	3.91	-0.43	0.62
PA4352_at	6.20	6.82	7.20	7.33	-0.75	0.62
PA4336_at	6.05	6.34	6.64	6.81	-0.53	0.63
PA1479_ccmE_at	6.15	6.48	5.75	5.83	0.53	0.63
PA3007_lexA_at	6.71	7.53	6.06	6.31	0.93	0.63
PA4896_at	3.42	3.56	3.02	3.08	0.44	0.63
PA5170_arcD_at	7.61	8.17	8.53	8.64	-0.70	0.63
PA0461_at	4.12	4.06	3.62	3.73	0.41	0.63
PA0522_r_at	7.10	7.34	6.80	6.74	0.45	0.63
PA0639_at	4.10	4.66	3.70	3.75	0.65	0.63
PA0645_at	3.73	3.71	3.25	3.38	0.41	0.63
PA0721_r_at	11.69	11.73	11.35	11.22	0.42	0.63
PA0932_cysM_at	5.25	5.45	4.76	4.97	0.49	0.63
PA1096_at	5.87	6.42	5.58	5.29	0.71	0.63
PA1559_at	4.21	3.82	3.39	3.51	0.56	0.63
PA1588_sucC_at	6.51	7.01	7.52	7.31	-0.66	0.63
PA1675_at	4.10	4.37	3.73	3.81	0.46	0.63
PA1676_at	4.74	5.09	4.48	4.21	0.57	0.63
PA1902_s_at	5.08	4.87	5.39	5.48	-0.46	0.63
PA2483_at	4.78	5.17	4.49	4.37	0.54	0.63
PA2501_at	7.50	8.13	8.43	8.68	-0.74	0.63
PA2582_at	5.50	5.93	5.05	5.19	0.60	0.63
PA2644_nuoI_at	5.03	5.26	5.53	5.73	-0.48	0.63

PA2735_at	4.75	4.73	4.23	4.38	0.43	0.63
PA2757_at	3.98	4.41	3.67	3.50	0.61	0.63
PA2778_at	4.45	4.46	4.10	4.01	0.40	0.63
PA2937_at	5.24	5.14	5.81	5.54	-0.48	0.63
PA3211_at	4.47	4.15	3.66	3.89	0.53	0.63
PA3218_at	3.41	3.33	2.93	3.01	0.40	0.63
PA3787_at	3.33	2.93	2.51	2.64	0.56	0.63
PA4131_at	6.83	7.00	7.29	7.44	-0.45	0.63
PA4132_at	4.37	4.37	4.76	4.77	-0.40	0.63
PA4451_at	4.52	4.67	3.89	4.22	0.53	0.63
PA4478_at	4.34	4.61	4.05	3.94	0.48	0.63
PA4487_at	5.43	5.30	5.83	5.76	-0.43	0.63
PA4723_dksA_at	7.54	7.63	7.24	7.04	0.44	0.63
PA4853_fis_at	6.94	6.54	6.15	6.24	0.54	0.63
PA4915_at	3.68	3.51	4.02	4.04	-0.43	0.63
PA4960_at	3.24	3.61	2.93	2.87	0.52	0.63
PA5423_at	5.68	6.05	5.40	5.24	0.54	0.63
PA5460_at	2.80	2.77	3.13	3.30	-0.43	0.63
PA5464_at	3.03	3.10	2.64	2.69	0.40	0.63
PA5550_at	3.95	4.06	3.64	3.52	0.42	0.63
PA2649_nuoN_at	2.77	2.87	3.32	3.16	-0.42	0.64
PA3459_at	3.47	3.54	2.83	3.18	0.50	0.64
PA0463_creB_at	4.56	4.48	4.03	4.18	0.42	0.64
PA1306_at	3.69	3.68	3.34	3.26	0.39	0.64
PA1667_at	3.62	3.97	4.27	4.33	-0.50	0.64
PA2830_htpX_at	4.19	4.61	3.88	3.84	0.54	0.64
PA4061_at	6.95	7.05	6.65	6.52	0.41	0.64
PA5171_arcA_at	8.57	9.29	9.62	9.78	-0.77	0.64
PA5351_at	6.89	5.29	4.70	4.46	1.52	0.64
PA5427_adhA_at	8.54	8.95	9.29	9.28	-0.54	0.64
PA0923_dinP_at	5.23	5.27	4.92	4.53	0.52	0.64
PA1001_phnA_at	4.25	4.38	4.69	4.73	-0.40	0.64
PA4731_panD_at	5.56	4.43	3.85	3.96	1.09	0.64
PA2641_nuoF_at	4.10	4.16	4.47	4.59	-0.40	0.65
PA3440_at	5.75	6.01	5.02	5.50	0.62	0.65
PA4041_at	3.24	3.22	3.56	3.67	-0.39	0.65
PA4552_pilW_at	4.58	5.02	5.43	5.30	-0.56	0.65
PA0783_putP_at	4.22	4.73	4.98	5.25	-0.64	0.65
PA1160_at	3.83	3.84	3.46	3.46	0.37	0.65
PA1200_at	4.26	4.36	3.81	3.98	0.41	0.65
PA3190_at	5.34	5.21	5.67	5.66	-0.39	0.65
PA4133_at	6.75	7.21	7.45	7.70	-0.60	0.65
PA4477_cafA_at	4.49	4.58	4.15	4.16	0.38	0.65
PA1587_lpdG_at	5.80	6.03	6.50	6.27	-0.47	0.66

PA0909_i_at	5.15	4.92	4.52	4.66	0.44	0.66
PA3201_at	4.55	5.04	4.19	4.26	0.57	0.66
PA3764_at	4.16	4.04	3.72	3.71	0.39	0.66
PA5172_arcB_at	8.68	9.23	9.46	9.77	-0.66	0.67
PA1194_at	3.79	3.88	3.41	3.50	0.38	0.67
PA3565_at	4.56	5.35	4.24	4.08	0.79	0.67
PA3616_at	4.62	5.24	4.30	4.25	0.65	0.67
PA0102_at	6.16	6.30	5.78	5.89	0.39	0.68
PA0612_i_at	4.32	5.72	3.61	3.88	1.27	0.68
PA0629_at	4.68	4.74	4.34	4.35	0.37	0.68
PA1401_at	3.81	3.37	4.04	4.23	-0.55	0.68
PA1475_ccmA_at	4.95	5.45	4.60	4.67	0.57	0.68
PA1793_ppiB_at	7.64	7.57	6.85	7.30	0.53	0.68
PA2323_at	5.17	5.42	5.72	5.71	-0.42	0.68
PA2329_at	3.70	3.36	4.17	3.92	-0.52	0.68
PA2799_at	3.56	3.51	3.09	3.22	0.38	0.68
PA3327_at	4.76	4.69	5.09	5.11	-0.37	0.68
PA4328_at	4.66	5.13	5.36	5.63	-0.60	0.68
PA4920_nadE_at	6.12	6.36	5.84	5.80	0.42	0.68
PA5182_at	6.21	6.88	5.96	5.71	0.71	0.68
PA0052_at	4.93	4.49	5.20	5.27	-0.52	0.68
PA1666_at	4.93	4.61	5.17	5.28	-0.46	0.69
PA5475_at	5.94	6.11	6.50	6.36	-0.40	0.69
PA2850_ohr_at	3.93	3.89	4.20	4.63	-0.51	0.69
PA0436_at	4.81	4.47	3.83	4.27	0.59	0.69
PA1174_napA_at	4.23	4.54	4.83	4.82	-0.44	0.69
PA2270_at	5.37	5.57	5.15	4.78	0.50	0.69
PA2504_at	3.58	3.65	3.97	3.98	-0.36	0.69
ig_1063544_1064555_at	4.53	4.60	4.20	4.21	0.36	0.70
PA1750_at	4.01	4.04	3.61	3.71	0.37	0.70
PA2288_r_at	6.85	7.40	6.62	6.41	0.60	0.70
PA0368_at	4.37	4.09	3.89	3.66	0.46	0.71
PA1555_at	8.76	8.98	9.24	9.28	-0.39	0.71
PA3187_at	2.92	2.92	3.33	3.23	-0.36	0.71
PA5040_pilQ_at	6.44	6.78	6.97	7.27	-0.51	0.71
PA5270_at	3.04	3.18	2.58	2.81	0.41	0.71
PA5314_at	3.16	3.34	2.79	2.92	0.39	0.71
PA5154_at	2.90	2.63	2.20	2.42	0.45	0.72
PA0631_at	5.49	5.97	5.11	5.28	0.54	0.72
PA0633_at	6.47	7.14	5.76	6.28	0.78	0.72
PA0668_tyrZ_at	4.72	4.49	4.25	4.15	0.40	0.72
PA0745_at	4.26	4.38	4.61	4.80	-0.38	0.72
PA0911_at	4.18	4.04	3.79	3.70	0.37	0.72
PA0985_at	5.12	4.72	4.35	4.52	0.49	0.72

PA1118_at	5.95	5.54	6.19	6.25	-0.48	0.72
PA1573_at	3.79	3.88	3.54	3.38	0.38	0.72
PA1888_at	3.86	3.98	4.33	4.25	-0.37	0.72
PA1904_s_at	3.79	4.52	4.77	4.96	-0.71	0.72
PA2381_at	6.40	6.92	7.11	7.38	-0.59	0.72
PA2621_at	6.15	6.48	6.69	6.85	-0.45	0.72
PA2643_nuoH_at	4.15	4.59	4.76	5.27	-0.64	0.72
PA2856_tesA_at	4.07	3.99	3.62	3.72	0.36	0.72
PA3006_at	6.57	6.98	5.92	6.40	0.62	0.72
PA3038_at	3.29	2.94	3.63	3.51	-0.46	0.72
PA3040_at	7.44	7.49	7.03	7.17	0.36	0.72
PA3293_at	2.72	2.79	3.14	3.07	-0.35	0.72
PA3662_at	6.96	6.99	6.53	6.69	0.36	0.72
PA4563_rpsT_at	6.30	6.88	5.89	6.07	0.60	0.72
PA5206_argE_at	3.75	3.73	3.46	3.25	0.39	0.72
PA5220_at	4.27	4.04	4.64	4.49	-0.41	0.72
PA0236_at	4.03	3.97	3.66	3.66	0.34	0.72
PA3617_recA_at	7.23	7.99	6.95	6.88	0.69	0.73
PA5021_at	3.87	3.67	3.45	3.33	0.39	0.73
PA0304_potI_at	3.97	3.70	4.31	4.18	-0.41	0.73
ig_3545880_3545073_at	6.14	6.64	5.94	5.79	0.53	0.73
PA0271_at	3.26	3.41	3.61	3.90	-0.42	0.73
PA0529_at	3.79	4.47	3.49	3.50	0.64	0.73
PA1068_at	5.25	5.74	5.01	4.96	0.51	0.73
PA1535_at	4.56	4.53	4.10	4.27	0.36	0.73
PA1650_at	2.96	2.68	2.34	2.48	0.41	0.73
PA2738_himA_at	9.73	9.63	9.32	9.36	0.35	0.73
PA3101_xcpT_at	4.42	4.34	4.70	4.74	-0.34	0.73
PA3785_at	5.51	5.81	6.01	6.15	-0.42	0.73
PA4703_at	3.45	3.21	3.63	4.06	-0.52	0.73
PA4996_rfaE_at	5.41	5.83	5.11	5.17	0.48	0.73
PA5152_at	4.48	4.42	3.79	4.19	0.46	0.73
PA4156_at	2.74	2.60	3.04	3.00	-0.35	0.74
PA0962_at	7.01	7.58	7.72	8.35	-0.74	0.75
PA4728_folK_at	4.51	4.45	4.18	4.10	0.34	0.75
PA1150_pys2_at	5.61	5.90	5.31	5.40	0.40	0.76
PA1241_at	3.51	3.64	3.22	3.24	0.34	0.76
PA5279_at	5.36	5.42	5.10	5.01	0.34	0.76
PA5523_at	3.75	3.94	3.44	3.53	0.36	0.76
ig_2905549_2906165_at	3.62	3.39	3.88	3.87	-0.37	0.77
PA0871_phhB_at	5.33	5.63	5.86	5.89	-0.40	0.77
PA1331_at	3.81	3.89	3.57	3.45	0.34	0.77
PA0121_at	5.28	5.36	5.05	4.89	0.35	0.77

PA0613_at	6.08	6.70	5.54	5.94	0.65	0.77
PA1244_at	5.07	5.60	5.81	5.92	-0.53	0.77
PA3204_at	4.37	4.63	4.15	4.09	0.38	0.77
PA3386_at	3.34	3.91	2.99	3.15	0.56	0.77
PA3387_rhlG_at	3.24	3.41	2.92	3.01	0.36	0.77
PA3812_iscA_at	6.85	7.47	6.59	6.58	0.58	0.77
PA4951_orn_at	3.94	4.08	3.71	3.62	0.34	0.77
PA4614_mscL_at	4.30	4.33	4.65	4.62	-0.32	0.77
PA5355_glcD_at	3.41	3.38	3.65	3.87	-0.36	0.77
PA0263_hcpC_s_ at	3.76	3.42	4.09	3.94	-0.43	0.78
PA1984_s_at	5.25	4.92	5.42	5.60	-0.43	0.78
PA4421_at	5.47	5.83	5.29	5.15	0.44	0.78
PA0389_at	3.23	3.27	2.97	2.88	0.33	0.78
PA1268_at	3.00	3.28	3.56	3.50	-0.39	0.78
PA1965_at	4.68	4.59	4.33	4.27	0.33	0.78
PA0074_ppkA_at	3.42	3.39	3.71	3.73	-0.32	0.78
PA0345_at	5.47	5.43	5.13	5.15	0.31	0.78
PA0646_at	5.06	4.85	4.58	4.63	0.35	0.78
PA0738_at	3.88	3.86	3.60	3.47	0.33	0.78
PA0937_at	5.10	4.95	4.68	4.70	0.33	0.78
PA0972_tolB_at	5.18	5.38	4.87	4.99	0.35	0.78
PA0986_i_at	3.03	2.98	3.33	3.32	-0.32	0.78
PA1105_fliJ_at	2.93	3.28	2.75	2.60	0.42	0.78
PA1178_oprH_at	3.70	4.51	3.20	3.55	0.73	0.78
PA1365_at	3.28	3.24	3.54	3.63	-0.32	0.78
PA1474_at	5.90	6.07	5.72	5.51	0.37	0.78
PA1608_at	3.10	3.50	2.72	2.94	0.46	0.78
PA1709_popD_at	6.17	6.69	6.94	6.93	-0.50	0.78
PA1746_at	6.61	7.20	7.35	7.62	-0.58	0.78
PA2075_at	3.68	3.49	3.32	3.05	0.40	0.78
PA2305_at	4.88	4.89	5.23	5.18	-0.32	0.78
PA2570_pa1L_at	3.17	3.09	3.69	3.37	-0.40	0.78
PA2583_at	4.49	4.20	4.02	3.91	0.39	0.78
PA2840_at	7.13	8.17	6.84	6.78	0.84	0.78
PA3622_rpoS_at	4.68	5.25	5.41	5.61	-0.55	0.78
PA3732_at	5.81	5.75	5.43	5.50	0.32	0.78
PA4296_at	4.38	4.46	4.70	4.79	-0.32	0.78
PA5027_at	4.69	4.71	5.07	4.98	-0.32	0.78
PA1007_at	3.50	3.55	3.28	3.09	0.34	0.79
PA3611_at	4.35	4.74	4.17	4.06	0.43	0.79
PA1685_masA_at	3.46	3.61	3.81	3.94	-0.34	0.79
PA4499_at	5.04	4.91	4.61	4.68	0.33	0.79
PA0044_exoT_at	5.83	6.66	6.84	7.03	-0.70	0.79
PA2008_fahA_at	3.18	3.20	3.50	3.49	-0.31	0.79

PA4129_at	5.06	5.17	5.42	5.45	-0.32	0.79
PA3081_at	3.89	4.05	4.45	4.23	-0.37	0.80
PA0525_at	5.07	5.47	4.84	4.84	0.42	0.80
PA1554_at	4.43	4.73	4.97	4.94	-0.37	0.80
PA3014_faoA_at	4.91	5.32	4.63	4.74	0.44	0.80
PA5544_at	4.00	3.73	4.33	4.17	-0.38	0.80
PA2983_at	4.48	4.68	5.00	4.87	-0.35	0.80
PA0626_at	3.80	3.61	3.32	3.41	0.34	0.80
PA2538_at	4.19	4.36	3.92	3.97	0.33	0.80
PA4113_at	3.79	3.35	4.00	4.03	-0.45	0.80
PA4948_at	4.73	5.17	4.53	4.48	0.45	0.80
ig_884799_884172_at	2.41	2.35	2.66	2.71	-0.31	0.80
PA0024_hemF_at	4.53	5.04	4.29	4.33	0.47	0.80
PA0129_gabP_at	2.92	2.74	3.07	3.35	-0.38	0.80
PA0512_at	3.53	3.57	3.78	4.05	-0.36	0.80
PA0537_at	4.27	4.71	3.94	4.12	0.46	0.80
PA0610_prtN_at	5.70	6.40	5.30	5.57	0.62	0.80
PA0624_at	6.38	6.40	5.81	6.18	0.40	0.80
PA0658_at	4.08	4.08	3.85	3.64	0.34	0.80
PA0854_fumC2_at	4.76	5.18	4.14	4.67	0.57	0.80
PA1493_cysP_at	3.39	3.81	3.92	4.23	-0.48	0.80
PA1589_sucD_at	6.88	7.11	7.28	7.46	-0.37	0.80
PA1848_at	3.43	3.63	3.15	3.23	0.34	0.80
PA2120_at	2.11	2.11	2.39	2.45	-0.30	0.80
PA2896_at	4.17	4.34	3.66	4.03	0.41	0.80
PA3010_at	4.62	4.91	5.15	5.11	-0.37	0.80
PA3069_at	3.91	4.24	3.55	3.77	0.42	0.80
PA3206_at	3.85	3.76	3.51	3.48	0.31	0.80
PA3251_at	2.78	2.96	2.55	2.54	0.33	0.80
PA3554_at	4.70	4.16	3.93	3.95	0.49	0.80
PA3819_at	5.55	5.87	5.29	5.38	0.38	0.80
PA4776_at	3.04	3.07	2.72	2.78	0.30	0.80
PA3152_hisH2_at	5.13	4.69	4.47	4.48	0.43	0.81
PA4555_pilY2_at	2.57	2.76	3.00	3.00	-0.33	0.81
PA0998_at	4.74	5.28	5.68	5.40	-0.53	0.81
PA2868_i_at	3.06	2.98	2.79	2.57	0.34	0.81
PA3126_ibpA_at	8.33	8.84	8.96	9.22	-0.50	0.81
PA3357_dsdA_at	3.19	3.85	3.03	2.88	0.56	0.81
PA4578_at	8.07	8.03	8.35	8.35	-0.30	0.81
PA4801_at	2.66	2.63	2.98	2.91	-0.30	0.81
PA4869_at	2.24	2.19	2.57	2.47	-0.31	0.81
PA5345_recG_at	3.60	3.63	3.34	3.29	0.30	0.81
PA4890_at	2.89	2.94	2.65	2.57	0.30	0.81

PA3639_accA_at	3.88	3.93	3.63	3.58	0.30	0.81
PA2572_at	3.27	3.79	4.06	3.95	-0.47	0.82
PA0362_fdx1_at	3.82	4.04	3.49	3.67	0.35	0.82
PA0649_trpG_at	4.23	4.71	3.97	4.07	0.45	0.82
PA1665_at	3.58	3.59	3.88	3.87	-0.29	0.82
PA2521_czcB_at	3.32	3.03	2.84	2.81	0.35	0.82
PA2648_nuoM_at	3.32	3.33	3.61	3.63	-0.29	0.82
PA3712_at	6.07	6.46	5.79	5.93	0.41	0.82
PA4527_pilC_at	5.68	5.63	5.28	5.42	0.31	0.82
PA4902_at	4.11	4.06	3.70	3.84	0.31	0.82
PA3814_iscS_at	6.19	7.27	5.88	5.97	0.80	0.83
PA5107_blc_at	4.54	5.14	4.37	4.29	0.51	0.83
PA2660_at	3.77	3.77	3.28	3.56	0.35	0.83
PA1175_napD_at	3.54	3.74	4.16	3.87	-0.38	0.83
PA3553_at	3.16	3.00	2.78	2.75	0.31	0.83
PA4078_at	2.94	2.48	3.32	3.04	-0.47	0.83
PA4476_at	3.69	4.26	3.52	3.46	0.49	0.83
PA4545_comL_at	5.76	5.58	5.22	5.42	0.35	0.83
PA4070_at	2.81	2.86	3.10	3.17	-0.30	0.83
PA4721_at	4.60	4.12	4.84	4.77	-0.44	0.83
PA0112_at	3.24	2.58	3.73	3.31	-0.61	0.84
PA1155_nrdB_at	6.96	7.66	7.75	8.05	-0.59	0.84
PA1301_at	3.53	3.54	3.31	3.16	0.31	0.84
PA1323_at	3.69	3.87	3.31	3.55	0.35	0.84
PA1869_at	3.68	3.73	3.90	4.23	-0.36	0.84
PA2320_gntR_at	4.74	4.78	4.50	4.44	0.29	0.84
PA4027_at	4.70	4.85	5.10	5.07	-0.31	0.84
PA4698_at	6.03	6.04	5.83	5.56	0.34	0.84
PA2527_at	3.28	3.19	2.99	2.89	0.30	0.84
PA3842_at	5.82	6.83	6.97	7.22	-0.77	0.84
PA5368_pstC_at	4.05	3.89	4.25	4.31	-0.31	0.84
PA2322_at	2.74	2.79	2.42	2.52	0.29	0.84
PA2647_nuoL_at	4.43	5.11	5.35	5.28	-0.55	0.84
PA3243_minC_at	3.28	3.24	2.79	3.05	0.34	0.84
PA4036_at	3.01	2.90	3.32	3.20	-0.30	0.84
PA4898_at	3.27	3.26	3.01	2.95	0.29	0.84
PA3239_at	3.88	4.23	3.40	3.80	0.46	0.84
ig_3526677_3527428_at	5.22	5.30	4.90	5.03	0.30	0.84
PA0953_at	4.38	3.92	4.58	4.57	-0.42	0.84
PA1495_at	2.77	2.72	3.10	2.98	-0.30	0.84
PA3472_at	2.70	3.30	2.43	2.57	0.50	0.84
PA3499_at	3.09	3.10	3.39	3.36	-0.28	0.84
PA0959_at	5.46	5.62	5.84	5.85	-0.30	0.84

PA0294_at	3.43	3.38	3.11	3.14	0.28	0.84
PA1837_at	3.27	2.99	3.51	3.44	-0.34	0.84
PA5247_at	3.66	3.43	3.84	3.89	-0.32	0.84
PA0289_at	3.38	3.39	3.61	3.74	-0.29	0.84
PA1020_at	3.13	2.64	2.37	2.52	0.44	0.84
PA1095_at	6.71	7.41	6.55	6.49	0.54	0.84
PA1112_at	4.68	4.70	4.46	4.32	0.30	0.84
PA2113_at	2.87	2.67	3.01	3.20	-0.34	0.84
PA2645_nuoJ_at	2.86	3.25	3.41	3.46	-0.38	0.84
PA2657_at	4.30	4.66	4.10	4.11	0.37	0.84
PA2855_at	4.07	4.34	3.94	3.77	0.35	0.84
PA2993_at	4.96	5.24	5.53	5.37	-0.35	0.84
PA3462_at	3.17	3.11	3.80	3.33	-0.42	0.84
PA3485_r_at	4.15	4.31	4.59	4.48	-0.31	0.84
PA4378_inaA_at	4.57	4.90	4.22	4.46	0.39	0.84
PA4479_mreD_at	5.75	6.56	5.63	5.46	0.61	0.84
PA4778_at	6.03	5.54	5.42	5.27	0.44	0.84
PA0039_at	5.01	5.09	4.78	4.75	0.28	0.85
PA0055_at	4.32	4.57	4.04	4.18	0.33	0.85
PA0451_at	3.21	3.24	3.56	3.45	-0.28	0.85
PA0630_at	5.13	5.22	4.89	4.90	0.28	0.85
PA0679_at	4.40	3.93	4.58	4.58	-0.41	0.85
PA1034_at	2.91	2.85	2.65	2.55	0.29	0.85
PA1041_at	5.17	5.39	5.57	5.60	-0.31	0.85
PA1299_at	4.71	4.33	4.18	4.10	0.38	0.85
PA2084_at	3.15	3.00	3.48	3.30	-0.32	0.85
PA2931_at	6.29	6.79	6.95	6.98	-0.43	0.85
PA3203_at	5.31	5.47	5.04	5.13	0.30	0.85
PA4067_oprG_at	8.94	9.62	9.69	9.99	-0.56	0.85
PA4309_pctA_at	3.94	4.75	3.70	3.78	0.60	0.85
PA4569_ispB_at	5.26	5.37	4.99	5.06	0.29	0.85
PA4704_at	5.26	5.59	5.76	5.80	-0.36	0.85
Pae_tRNA_Lys_f_at	2.87	2.88	2.68	2.41	0.33	0.85
PA2628_at	2.65	2.66	2.47	2.18	0.33	0.86
PA2760_at	4.00	4.22	4.31	4.73	-0.41	0.86
PA3877_narK1_at	3.72	4.10	3.53	3.55	0.37	0.86
PA5138_at	2.65	2.61	2.87	2.95	-0.28	0.86
PA3731_at	6.06	6.15	5.70	5.90	0.31	0.86
PA3706_at	3.28	3.28	3.49	3.65	-0.29	0.86
PA2104_at	3.89	4.24	3.80	3.57	0.38	0.87
PA3675_at	4.16	4.24	3.84	3.98	0.29	0.87
PA2915_at	3.20	3.16	3.41	3.52	-0.28	0.87
PA3439_folX_at	2.55	2.65	2.84	2.93	-0.28	0.87
Pae_tRNA_Ala_f_	3.44	3.58	3.30	3.08	0.32	0.87

at						
PA2365_at	3.75	3.58	3.97	3.94	-0.29	0.87
PA0506_at	5.94	6.70	5.74	5.78	0.56	0.87
PA0588_at	9.01	8.99	9.24	9.30	-0.27	0.87
PA0758_at	2.98	3.12	3.43	3.27	-0.30	0.87
PA1102_fliG_at	5.88	6.32	5.70	5.72	0.39	0.87
PA1362_i_at	2.34	2.47	2.61	2.83	-0.31	0.87
PA3372_at	3.21	3.18	3.45	3.48	-0.27	0.87
PA3575_at	3.78	3.86	3.52	3.57	0.28	0.87
PA3614_at	4.29	4.74	4.82	5.05	-0.42	0.87
PA3817_at	4.49	4.63	4.34	4.16	0.30	0.87
PA4210_s_at	3.45	3.18	3.66	3.60	-0.32	0.87
PA4520_at	3.58	3.86	3.41	3.38	0.32	0.87
PA4880_at	3.35	3.56	3.15	3.15	0.30	0.87
PA5108_at	4.11	4.68	3.73	4.07	0.50	0.87
PA5289_at	6.02	5.75	5.68	5.30	0.40	0.87
ig_1349416_1348401_at	3.69	3.77	3.91	4.16	-0.31	0.88
ig_2244492_2243603_at	3.18	3.18	2.92	2.91	0.27	0.88
PA0087_at	4.06	4.26	3.84	3.90	0.29	0.88
PA0167_at	3.01	2.77	2.57	2.62	0.29	0.88
PA0179_at	4.89	4.65	5.08	5.07	-0.30	0.88
PA0281_cysW_at	3.33	2.84	3.43	3.56	-0.41	0.88
PA0326_at	3.03	3.02	2.75	2.79	0.26	0.88
PA0336_at	5.98	6.41	5.50	5.95	0.47	0.88
PA0458_at	4.23	3.82	4.48	4.31	-0.37	0.88
PA0627_at	6.88	6.86	6.36	6.72	0.34	0.88
PA0657_at	3.88	3.99	4.16	4.25	-0.28	0.88
PA0671_at	4.15	4.26	3.98	3.87	0.28	0.88
PA0869_pbpG_at	4.87	5.00	4.73	4.57	0.28	0.88
PA0895_aruC_at	4.64	4.75	4.37	4.47	0.28	0.88
PA0913_mgtE_at	5.86	6.51	5.66	5.76	0.47	0.88
PA0915_at	2.94	2.98	2.74	2.65	0.26	0.88
PA1098_fleS_at	5.74	6.24	5.66	5.49	0.42	0.88
PA1249_aprA_at	3.88	3.60	3.98	4.17	-0.33	0.88
PA1344_at	3.84	3.63	4.05	4.00	-0.29	0.88
PA1368_at	3.09	2.92	3.50	3.20	-0.35	0.88
PA1461_at	3.27	3.54	3.14	3.03	0.32	0.88
PA1546_hemN_at	3.77	4.28	4.37	4.50	-0.42	0.88
PA1607_at	3.41	3.53	3.70	3.79	-0.27	0.88
PA1680_at	4.20	4.06	3.83	3.87	0.28	0.88
PA1708_popB_at	6.89	8.00	8.11	8.26	-0.74	0.88
PA1728_at	3.76	3.97	4.07	4.33	-0.33	0.88

PA1787_acnB_at	5.63	5.79	5.94	6.04	-0.28	0.88
PA1858_str_at	2.94	2.88	2.71	2.57	0.27	0.88
PA1971_braZ_at	3.44	3.49	3.91	3.64	-0.31	0.88
PA2092_at	2.71	2.45	2.90	2.87	-0.30	0.88
PA2173_at	2.59	2.53	2.98	2.74	-0.30	0.88
PA2196_at	3.46	3.56	3.71	3.89	-0.29	0.88
PA2232_at	3.29	3.34	3.53	3.63	-0.27	0.88
PA2286_at	3.51	3.42	3.82	3.68	-0.28	0.88
PA2476_dsbG_at	4.14	3.95	3.78	3.72	0.29	0.88
PA2753_at	5.54	6.38	6.55	6.54	-0.58	0.88
PA2795_at	2.85	2.91	3.15	3.13	-0.26	0.88
PA2816_i_at	2.87	2.88	2.65	2.59	0.26	0.88
PA2862_lipA_at	2.51	2.57	2.77	2.83	-0.26	0.88
PA2883_at	7.64	7.68	7.42	7.36	0.27	0.88
PA3008_at	3.88	4.97	3.64	3.72	0.75	0.88
PA3056_at	3.04	3.16	3.36	3.37	-0.26	0.88
PA3195_gapA_at	6.08	6.14	6.36	6.38	-0.26	0.88
PA3259_at	5.68	5.77	5.50	5.41	0.27	0.88
PA3300_fadD2_at	2.34	2.48	2.80	2.61	-0.29	0.88
PA3337_rfaD_at	5.27	5.27	5.57	5.51	-0.27	0.88
PA3353_at	6.29	6.61	6.10	6.16	0.33	0.88
PA3415_at	5.59	5.62	5.88	5.86	-0.27	0.88
PA3491_at	5.17	5.10	4.91	4.84	0.26	0.88
PA3608_potB_at	2.39	2.48	2.67	2.73	-0.27	0.88
PA3684_i_at	5.19	5.73	5.08	4.98	0.43	0.88
PA3693_at	3.52	3.05	3.67	3.70	-0.40	0.88
PA3943_at	3.30	3.34	3.54	3.63	-0.27	0.88
PA4011_at	3.91	3.87	3.66	3.58	0.27	0.88
PA4069_at	3.70	3.83	3.52	3.46	0.28	0.88
PA4222_at	4.62	4.31	4.80	4.80	-0.33	0.88
PA4266_fusA1_at	6.33	7.09	7.18	7.31	-0.54	0.88
PA4300_at	3.38	3.15	3.05	2.85	0.32	0.88
PA4554_pilY1_at	3.33	3.53	3.73	3.71	-0.29	0.88
PA4577_at	7.57	7.91	8.09	8.05	-0.33	0.88
PA4596_at	4.65	4.94	5.14	5.09	-0.32	0.88
PA4611_at	6.90	6.94	7.16	7.20	-0.26	0.88
PA4702_at	4.22	4.77	4.92	4.94	-0.44	0.88
PA4779_at	3.20	2.97	3.28	3.56	-0.34	0.88
PA4866_at	3.00	2.74	3.10	3.29	-0.32	0.88
PA4917_at	5.16	4.85	4.66	4.70	0.33	0.88
PA5033_at	2.93	2.90	3.28	3.12	-0.28	0.88
PA5146_at	4.35	4.54	4.20	4.09	0.30	0.88
PA5165_at	3.40	3.36	3.22	2.95	0.30	0.88
PA5168_at	3.36	3.11	3.47	3.64	-0.32	0.88

PA5380_at	5.09	4.95	4.77	4.73	0.27	0.88
PA5476_citA_at	3.48	3.28	3.15	3.01	0.30	0.88
PA5507_at	4.44	4.64	4.77	4.94	-0.31	0.88
Pae_tRNA_Ser_f_at	5.54	4.92	5.80	5.60	-0.47	0.88
ig_2558918_2557964_s_at	2.80	2.89	2.68	2.38	0.31	0.88
PA1556_at	8.69	8.85	8.98	9.14	-0.29	0.88
PA1585_sucA_at	4.75	5.23	5.29	5.50	-0.40	0.88
PA2300_chiC_at	3.73	3.60	3.85	4.08	-0.30	0.88
PA3443_at	3.40	3.34	3.01	3.18	0.28	0.88
PA4694_ilvC_at	5.06	5.34	5.46	5.55	-0.31	0.88
ig_517462_518083_at	3.29	3.59	3.71	3.80	-0.31	0.88
PA0176_at	4.70	4.33	4.95	4.78	-0.35	0.88
PA0480_at	3.65	3.39	3.81	3.82	-0.29	0.88
PA0917_kup_at	3.12	3.47	3.65	3.59	-0.33	0.88
PA0945_purM_at	5.19	5.35	5.04	4.96	0.27	0.88
PA0958_oprD_at	7.13	7.82	7.87	8.06	-0.49	0.88
PA1015_at	5.06	4.71	4.58	4.53	0.33	0.88
PA1173_napB_at	2.92	2.95	3.20	3.18	-0.25	0.88
PA1440_at	6.96	7.32	6.82	6.79	0.33	0.88
PA1657_at	4.51	4.32	4.71	4.67	-0.27	0.88
PA1658_at	2.63	2.67	2.89	2.91	-0.25	0.88
PA2277_arsR_at	4.33	4.31	4.02	4.11	0.26	0.88
PA3021_at	4.71	5.06	5.25	5.19	-0.33	0.88
PA3141_wbpM_g_at	3.79	4.23	3.71	3.55	0.38	0.88
PA3228_at	4.74	4.74	5.00	4.98	-0.25	0.88
PA3503_at	3.28	3.36	3.84	3.47	-0.34	0.88
PA3967_at	2.65	2.98	2.56	2.40	0.34	0.88
PA4370_at	5.11	5.14	5.35	5.41	-0.26	0.88
PA4727_pcnB_at	4.65	4.99	4.52	4.47	0.32	0.88
PA0918_at	7.42	7.94	8.04	8.12	-0.40	0.88
PA2066_at	4.39	4.52	4.69	4.74	-0.26	0.88
PA2200_at	3.56	3.69	3.89	3.88	-0.26	0.88
PA2550_at	2.74	2.64	2.92	2.97	-0.26	0.88
PA1995_i_at	4.12	4.30	4.40	4.61	-0.30	0.89
PA1553_at	4.00	4.28	4.47	4.41	-0.30	0.89
PA4622_at	3.95	3.61	4.29	3.99	-0.36	0.89
PA1113_at	2.86	2.71	3.07	3.03	-0.26	0.90
PA0388_at	6.51	6.81	6.88	7.08	-0.32	0.90
PA0554_at	4.56	4.76	4.42	4.34	0.28	0.90
PA1540_at	3.04	3.27	2.52	3.00	0.40	0.90
PA1766_at	4.39	4.40	4.70	4.60	-0.25	0.90

PA1915_at	3.66	3.57	3.79	4.01	-0.28	0.90
PA2920_at	3.99	4.24	4.33	4.51	-0.30	0.90
PA3238_at	3.35	3.50	3.06	3.23	0.28	0.90
PA3444_at	3.28	3.06	2.52	3.02	0.40	0.90
PA3758_at	2.87	3.03	2.72	2.66	0.27	0.90
PA4195_at	3.42	3.39	3.21	3.09	0.26	0.90
PA4354_at	2.73	2.90	2.49	2.60	0.27	0.90
Pae_orfM_at	2.95	2.55	3.09	3.09	-0.34	0.90
PA5437_at	3.86	3.76	3.97	4.23	-0.29	0.91
Pae_M98270cds1_at	2.42	2.25	2.60	2.60	-0.26	0.91
PA0632_at	5.03	4.77	4.59	4.63	0.29	0.91
PA1374_at	3.41	3.34	2.70	3.25	0.40	0.91
PA4553_pilX_at	4.01	4.00	4.30	4.21	-0.25	0.91
PA0070_at	6.75	7.28	7.40	7.41	-0.39	0.91
PA3963_at	3.08	2.83	2.66	2.68	0.28	0.91
PA4682_at	4.01	3.93	4.36	4.14	-0.28	0.91
PA5335_at	3.42	3.22	3.08	3.01	0.27	0.91
PA1659_at	3.23	3.04	3.62	3.30	-0.32	0.91
PA4049_at	3.05	3.10	3.24	3.47	-0.28	0.91
PA0637_at	4.64	4.59	4.15	4.47	0.31	0.91
PA2278_arsB_at	4.28	4.10	4.05	3.53	0.40	0.91
PA3333_fabH2_at	3.47	3.67	3.77	3.95	-0.29	0.91
PA3451_at	2.47	2.47	2.65	2.81	-0.26	0.91
PA0462_at	5.55	5.99	5.42	5.41	0.35	0.91
PA0505_at	4.99	5.53	4.82	4.91	0.39	0.91
PA1713_exsA_at	5.12	5.95	5.98	6.19	-0.55	0.91
PA3895_at	4.03	3.99	3.83	3.69	0.25	0.91
PA4899_at	3.58	3.48	3.78	3.77	-0.25	0.91
Pae_AF291700cds_at	3.09	2.59	3.35	3.13	-0.40	0.91
PA2955_at	2.59	2.73	2.45	2.35	0.26	0.91
PA4438_at	4.00	3.90	3.73	3.67	0.25	0.91
PA3881_at	4.56	4.90	4.41	4.43	0.31	0.91
PA1430_lasR_at	5.74	5.79	6.14	5.93	-0.27	0.91
PA2423_at	3.99	3.76	3.44	3.69	0.31	0.91
PA1195_at	3.69	4.24	3.60	3.54	0.40	0.91
PA1547_at	3.45	3.48	3.28	3.14	0.25	0.91
PA1806_fabI_at	4.24	4.04	4.43	4.39	-0.27	0.91
PA3143_at	3.08	3.08	3.36	3.29	-0.24	0.91
PA3529_at	6.82	7.26	7.34	7.44	-0.35	0.91
PA4147_acoR_at	4.16	4.28	4.48	4.45	-0.25	0.91
PA4180_at	4.07	3.99	4.31	4.24	-0.25	0.91
PA1372_at	2.61	2.44	2.25	2.28	0.26	0.92

PA2048_at	3.92	3.82	4.26	4.03	-0.28	0.92
PA2556_at	3.08	2.96	2.74	2.80	0.25	0.92
PA4700_mrcB_at	4.15	4.69	4.10	3.94	0.40	0.92
PA0159_at	3.30	3.07	3.54	3.39	-0.28	0.92
PA0214_at	2.87	2.70	2.60	2.43	0.27	0.92
PA0355_pfpI_at	3.11	3.11	2.88	2.86	0.24	0.92
PA0489_at	2.96	2.90	2.64	2.73	0.24	0.92
PA0900_at	4.99	5.32	4.62	4.98	0.36	0.92
PA1008_bcp_at	4.96	4.95	4.74	4.70	0.23	0.92
PA1077_flgB_at	3.49	3.24	2.77	3.22	0.37	0.92
PA1152_at	3.45	3.06	2.99	2.84	0.33	0.92
PA1216_at	2.38	2.31	2.60	2.56	-0.24	0.92
PA1432_lasI_at	6.34	6.28	6.54	6.56	-0.24	0.92
PA1605_at	4.40	4.51	4.26	4.16	0.25	0.92
PA1719_pscF_at	5.44	6.02	6.07	6.20	-0.40	0.92
PA1885_at	3.28	3.89	3.14	3.19	0.42	0.92
PA2001_atoB_at	3.80	3.80	4.10	3.99	-0.24	0.92
PA2006_at	2.68	2.77	3.17	2.86	-0.29	0.92
PA2089_at	2.66	2.65	2.93	2.86	-0.24	0.92
PA2330_at	3.38	3.33	3.68	3.53	-0.25	0.92
PA2651_at	4.30	3.93	3.79	3.82	0.32	0.92
PA2667_at	3.23	3.79	3.04	3.17	0.40	0.92
PA3343_at	3.70	3.38	3.32	3.12	0.31	0.92
PA3455_at	3.88	3.77	4.07	4.07	-0.24	0.92
PA3463_at	4.67	4.94	4.51	4.53	0.28	0.92
PA3747_at	3.70	4.10	3.61	3.53	0.33	0.92
PA3795_at	4.57	4.09	3.91	4.02	0.36	0.92
PA4020_mpl_at	4.19	4.65	4.20	3.82	0.41	0.92
PA4064_at	3.01	2.54	3.16	3.09	-0.35	0.92
PA4179_at	2.62	2.37	2.72	2.84	-0.28	0.92
PA4360_at	5.42	5.62	5.15	5.32	0.28	0.92
PA4405_at	2.99	2.84	2.56	2.74	0.27	0.92
PA4420_at	4.92	5.24	4.81	4.75	0.30	0.92
PA4526_pilB_at	5.20	5.25	5.45	5.47	-0.24	0.92
PA4787_at	4.35	4.50	4.24	4.08	0.26	0.92
PA4802_at	3.77	3.67	3.96	3.97	-0.24	0.92
PA4928_at	3.53	3.89	3.24	3.50	0.34	0.92
PA4953_motB_at	3.76	3.85	3.59	3.54	0.24	0.92
PA5133_at	5.41	6.00	5.27	5.33	0.40	0.92
PA5204_argA_at	3.73	3.57	3.87	3.95	-0.25	0.92
PA5399_at	4.23	4.05	4.48	4.33	-0.27	0.92
PA5505_at	3.46	3.65	3.77	3.86	-0.26	0.92
PA5517_at	3.33	3.42	3.10	3.16	0.24	0.92
PA4112_at	3.23	3.15	3.43	3.43	-0.24	0.92

PA1782_at	3.15	3.03	3.29	3.39	-0.25	0.93
PA2573_at	4.33	3.92	4.47	4.44	-0.33	0.93
PA3361_at	5.34	5.65	5.72	5.87	-0.30	0.93
PA0049_at	3.08	2.85	3.17	3.30	-0.27	0.93
PA1246_aprD_at	3.24	2.80	3.38	3.33	-0.34	0.93
PA2784_at	4.83	4.34	5.11	4.83	-0.39	0.93
PA5053_hslV_at	5.91	7.11	5.83	5.80	0.70	0.93
PA5415_glyA1_s_at	4.25	4.24	4.50	4.45	-0.23	0.93
ig_327284_326671_at	2.81	2.55	2.89	3.03	-0.28	0.93
PA0377_at	5.69	5.43	5.36	5.20	0.28	0.93
PA0590_apaH_at	5.34	5.76	5.10	5.31	0.34	0.93
PA1411_at	2.79	3.01	3.25	3.10	-0.27	0.93
PA4718_at	3.83	3.81	3.97	4.18	-0.26	0.93
PA5041_pilP_at	5.10	5.65	5.74	5.76	-0.38	0.93
PA5458_at	3.58	3.26	3.12	3.15	0.29	0.93
PA4941_hflC_at	5.99	6.18	5.81	5.85	0.25	0.93
AFFX-Bsubtilis_thrC_at	3.99	4.44	4.42	4.78	-0.38	0.93
ig_1947041_1948502_at	3.72	3.97	3.61	3.62	0.23	0.93
ig_1948502_1947041_at	2.45	2.39	2.66	2.61	-0.22	0.93
ig_2341640_2342493_at	2.58	2.65	2.84	2.84	-0.22	0.93
ig_2918966_2918211_at	3.33	3.36	3.17	3.08	0.22	0.93
ig_3112877_3112150_at	3.29	3.60	3.26	3.06	0.28	0.93
ig_4297249_4294604_at	2.67	2.59	2.86	2.79	-0.20	0.93
ig_4889111_4888194_at	2.85	3.13	2.67	2.78	0.27	0.93
ig_5130767_5131427_at	3.18	3.61	2.88	3.20	0.35	0.93
PA0027_at	4.27	3.93	4.31	4.48	-0.29	0.93
PA0041_at	3.15	3.02	3.29	3.33	-0.22	0.93
PA0050_r_at	6.82	6.56	6.99	6.88	-0.25	0.93
PA0067_prlC_at	6.71	6.91	6.54	6.63	0.23	0.93
PA0077_at	3.70	4.42	4.31	4.91	-0.55	0.93
PA0100_at	5.12	5.30	5.64	5.33	-0.28	0.93
PA0104_at	4.27	3.77	4.72	4.15	-0.42	0.93
PA0113_at	3.66	3.40	3.69	3.91	-0.27	0.93
PA0114_at	4.61	4.89	4.28	4.64	0.29	0.93
PA0175_at	3.63	3.43	3.77	3.77	-0.24	0.93
PA0177_at	2.18	2.16	2.42	2.35	-0.22	0.93

PA0200_i_at	6.45	6.67	6.87	6.77	-0.26	0.93
PA0222_at	2.78	2.63	3.18	2.80	-0.29	0.93
PA0247_pobA_at	2.74	2.59	2.87	2.95	-0.24	0.93
PA0261_at	3.94	3.74	4.12	4.02	-0.23	0.93
PA0284_at	4.80	4.30	4.84	4.96	-0.35	0.93
PA0288_speB1_at	3.03	2.64	3.13	3.12	-0.29	0.93
PA0322_at	3.20	2.88	3.27	3.36	-0.27	0.93
PA0347_glpQ_at	2.81	2.76	2.67	2.45	0.22	0.93
PA0351_at	4.64	4.53	4.42	4.31	0.22	0.93
PA0358_at	3.70	4.05	3.62	3.55	0.29	0.93
PA0365_at	5.41	5.07	5.04	4.92	0.26	0.93
PA0366_at	4.34	4.54	4.19	4.23	0.23	0.93
PA0370_at	4.08	4.55	4.55	4.72	-0.32	0.93
PA0376_rpoH_at	8.19	9.27	8.10	8.15	0.60	0.93
PA0386_at	2.75	2.98	2.61	2.65	0.24	0.93
PA0393_proC_at	4.33	4.38	4.16	4.12	0.21	0.93
PA0399_at	2.82	2.96	3.25	3.02	-0.25	0.93
PA0413_at	5.52	6.33	6.44	6.36	-0.47	0.93
PA0417_at	3.31	3.20	2.97	3.10	0.21	0.93
PA0423_at	3.31	3.56	3.06	3.28	0.27	0.93
PA0503_at	3.80	3.79	3.56	3.64	0.20	0.93
PA0511_nirJ_at	5.66	5.62	5.76	6.03	-0.26	0.93
PA0515_at	6.45	6.87	6.91	7.08	-0.33	0.93
PA0518_nirM_at	8.09	8.40	8.52	8.47	-0.25	0.93
PA0520_nirQ_at	5.78	6.03	5.52	5.73	0.28	0.93
PA0528_at	4.48	4.43	4.76	4.59	-0.22	0.93
PA0530_at	3.86	3.59	3.48	3.48	0.24	0.93
PA0578_at	4.30	4.74	4.34	4.07	0.31	0.93
PA0596_at	6.14	6.60	6.11	6.02	0.31	0.93
PA0602_at	3.49	3.07	3.79	3.43	-0.33	0.93
PA0614_at	5.36	5.64	5.34	5.16	0.25	0.93
PA0618_at	5.06	5.61	5.09	4.92	0.34	0.93
PA0634_at	3.59	3.62	3.13	3.51	0.28	0.93
PA0640_at	4.94	4.58	4.43	4.52	0.29	0.93
PA0642_at	3.04	2.93	2.76	2.81	0.20	0.93
PA0666_at	2.74	2.75	2.58	2.47	0.22	0.93
PA0667_at	4.25	4.69	3.78	4.37	0.40	0.93
PA0702_at	2.66	2.71	3.03	2.78	-0.23	0.93
PA0705_at	3.81	3.95	3.60	3.69	0.23	0.93
PA0720_at	12.15	12.48	11.84	12.17	0.31	0.93
PA0744_at	6.00	6.04	6.27	6.22	-0.22	0.93
PA0763_mucA_at	9.20	9.88	9.05	9.23	0.40	0.93
PA0766_mucD_at	6.06	6.67	5.95	6.07	0.36	0.93
PA0777_at	2.99	3.12	2.84	2.85	0.21	0.93

PA0788_at	2.94	3.11	3.18	3.40	-0.27	0.93
PA0805_at	6.11	7.25	6.00	6.11	0.62	0.93
PA0833_at	5.26	6.40	5.12	5.33	0.61	0.93
PA0838_at	3.13	3.48	3.46	3.80	-0.33	0.93
PA0862_at	4.57	4.45	4.31	4.26	0.23	0.93
PA0887_acsA_at	3.72	3.38	3.65	4.25	-0.40	0.93
PA0889_aotQ_at	3.38	3.63	3.69	3.87	-0.28	0.93
PA0891_at	5.97	6.06	5.83	5.77	0.21	0.93
PA0938_at	3.87	3.78	3.69	3.51	0.23	0.93
PA0970_tolR_at	5.23	5.19	5.06	4.90	0.23	0.93
PA0994_at	4.00	3.82	3.70	3.62	0.25	0.93
PA1049_pdxH_at	4.67	4.96	5.06	5.09	-0.26	0.93
PA1050_at	4.62	4.61	4.40	4.38	0.23	0.93
PA1052_at	2.89	2.83	3.05	3.07	-0.20	0.93
PA1064_at	4.30	4.44	4.08	4.23	0.22	0.93
PA1084_flgI_at	3.47	3.64	3.38	3.29	0.22	0.93
PA1101_fliF_at	4.69	5.48	4.65	4.64	0.44	0.93
PA1114_at	6.03	6.37	6.01	5.79	0.30	0.93
PA1133_at	3.45	3.09	3.51	3.58	-0.27	0.93
PA1158_at	3.19	3.14	3.04	2.81	0.24	0.93
PA1166_at	3.67	3.43	3.99	3.68	-0.28	0.93
PA1176_napF_at	2.46	2.70	2.85	2.82	-0.25	0.93
PA1177_napE_at	2.50	2.42	2.72	2.61	-0.21	0.93
PA1218_at	2.60	2.54	2.81	2.74	-0.20	0.93
PA1229_at	4.12	4.22	3.83	4.04	0.23	0.93
PA1252_at	3.73	3.54	3.45	3.34	0.24	0.93
PA1272_cobO_at	4.98	5.07	4.84	4.77	0.22	0.93
PA1414_at	10.63	10.85	10.95	10.99	-0.23	0.93
PA1420_at	2.42	2.47	2.61	2.74	-0.23	0.93
PA1434_at	3.43	3.30	3.12	3.15	0.23	0.93
PA1469_at	3.54	3.90	3.48	3.39	0.29	0.93
PA1471_at	4.11	5.10	4.01	4.09	0.55	0.93
PA1504_at	3.13	3.23	2.93	3.00	0.22	0.93
PA1505_moaA2_at	2.50	2.72	2.47	2.27	0.24	0.93
PA1516_at	3.52	3.53	3.90	3.64	-0.24	0.93
PA1526_at	4.63	4.62	4.34	4.48	0.21	0.93
PA1537_at	3.51	3.43	3.29	3.18	0.23	0.93
PA1566_at	2.40	2.23	2.65	2.45	-0.24	0.93
PA1584_sdhB_at	6.98	7.40	7.44	7.49	-0.28	0.93
PA1590_braB_at	4.09	4.11	4.41	4.26	-0.24	0.93
PA1619_at	3.05	3.01	2.86	2.75	0.23	0.93
PA1620_at	4.03	3.92	4.22	4.13	-0.21	0.93
PA1662_at	3.25	3.03	3.43	3.36	-0.25	0.93
PA1698_popN_at	5.08	5.40	5.60	5.43	-0.27	0.93

PA1744_at	3.66	3.43	3.73	3.86	-0.25	0.93
PA1759_at	5.35	5.25	5.11	5.00	0.24	0.93
PA1769_at	3.63	3.93	3.54	3.51	0.25	0.93
PA1770_ppsA_at	2.71	3.21	3.16	3.56	-0.40	0.93
PA1811_at	3.00	3.12	3.28	3.28	-0.21	0.93
PA1813_at	3.37	3.62	3.83	3.68	-0.26	0.93
PA1863_modA_at	3.57	4.32	3.46	3.54	0.45	0.93
PA1906_at	3.06	2.84	2.74	2.71	0.23	0.93
PA1913_at	3.09	2.89	3.15	3.28	-0.22	0.93
PA1917_at	2.56	2.39	2.79	2.64	-0.24	0.93
PA1919_at	3.15	3.04	3.39	3.27	-0.23	0.93
PA1998_at	3.46	3.30	3.08	3.22	0.23	0.93
PA2007_maiA_i_at	3.07	3.30	3.42	3.41	-0.23	0.93
PA2020_at	3.82	3.98	3.72	3.64	0.22	0.93
PA2028_at	3.16	2.98	3.17	3.57	-0.30	0.93
PA2036_at	3.22	3.04	3.40	3.29	-0.22	0.93
PA2053_cynT_at	2.98	2.89	3.14	3.14	-0.20	0.93
PA2070_at	3.34	3.01	3.46	3.41	-0.26	0.93
PA2098_at	3.88	3.48	3.97	3.97	-0.29	0.93
PA2101_at	3.53	3.58	3.81	3.75	-0.22	0.93
PA2115_at	2.96	3.40	3.56	3.41	-0.30	0.93
PA2117_at	3.51	3.47	3.63	3.75	-0.20	0.93
PA2140_at	3.52	3.47	3.19	3.37	0.21	0.93
PA2146_i_at	4.36	3.68	4.28	4.69	-0.46	0.93
PA2172_at	2.64	2.48	2.99	2.66	-0.27	0.93
PA2197_at	4.33	4.25	4.49	4.55	-0.23	0.93
PA2202_at	2.53	2.24	2.69	2.58	-0.25	0.93
PA2204_at	4.62	3.98	4.58	4.78	-0.38	0.93
PA2249_bkdB_at	4.74	4.48	4.92	4.83	-0.27	0.93
PA2254_pvcA_at	3.22	3.10	2.94	2.91	0.23	0.93
PA2275_at	3.48	3.59	3.19	3.39	0.24	0.93
PA2352_at	3.24	3.32	3.45	3.58	-0.24	0.93
PA2356_msuD_at	2.83	2.54	2.88	2.96	-0.24	0.93
PA2422_at	2.86	2.72	3.03	2.98	-0.21	0.93
PA2432_at	2.80	2.61	2.36	2.54	0.25	0.93
PA2434_at	3.20	3.20	3.06	2.84	0.25	0.93
PA2447_at	3.30	3.50	3.12	3.20	0.24	0.93
PA2482_at	4.35	5.13	4.16	4.35	0.48	0.93
PA2486_at	2.37	2.63	2.06	2.38	0.28	0.93
PA2517_xylY_at	3.30	3.22	3.51	3.44	-0.21	0.93
PA2519_xylS_at	2.68	2.47	2.27	2.39	0.24	0.93
PA2560_at	2.44	2.50	2.25	2.27	0.21	0.93
PA2561_at	3.78	4.01	3.56	3.72	0.26	0.93

PA2562_at	7.16	7.30	6.86	7.11	0.25	0.93
PA2565_at	4.11	4.09	4.43	4.26	-0.24	0.93
PA2594_at	3.96	3.94	4.20	4.13	-0.21	0.93
PA2623_icd_at	6.03	6.67	6.71	6.76	-0.38	0.93
PA2632_at	3.95	4.02	4.17	4.24	-0.22	0.93
PA2659_at	4.54	4.15	3.98	4.13	0.29	0.93
PA2664_fhp_at	3.57	3.85	3.53	3.31	0.29	0.93
PA2682_at	2.86	2.55	2.45	2.47	0.25	0.93
PA2715_at	2.22	2.15	2.45	2.36	-0.22	0.93
PA2733_at	5.07	4.83	4.64	4.76	0.25	0.93
PA2748_at	3.75	3.46	3.42	3.26	0.26	0.93
PA2762_at	4.16	3.87	3.79	3.77	0.24	0.93
PA2807_at	3.11	2.92	3.45	3.14	-0.28	0.93
PA2808_i_at	2.76	2.38	2.98	2.77	-0.30	0.93
PA2968_fabD_at	2.92	2.95	2.57	2.82	0.24	0.93
PA2977_murB_at	2.67	2.52	2.98	2.73	-0.26	0.93
PA2986_at	3.65	3.46	3.34	3.32	0.23	0.93
PA2996_nqrD_at	3.47	3.52	3.82	3.66	-0.24	0.93
PA2997_nqrC_at	3.07	3.09	3.48	3.19	-0.25	0.93
PA3016_at	3.48	3.53	3.62	3.83	-0.22	0.93
PA3026_at	4.08	3.83	4.17	4.21	-0.23	0.93
PA3031_at	5.18	5.69	5.06	5.14	0.34	0.93
PA3055_at	6.71	6.84	6.62	6.48	0.22	0.93
PA3077_at	3.47	3.52	3.72	3.68	-0.20	0.93
PA3100_xcpU_at	4.13	4.31	4.36	4.56	-0.24	0.93
PA3103_xcpR_at	4.51	4.37	4.91	4.57	-0.30	0.93
PA3118_leuB_at	3.79	4.14	3.80	3.57	0.28	0.93
PA3207_at	2.93	2.92	2.72	2.73	0.20	0.93
PA3209_at	3.46	3.32	3.29	2.95	0.27	0.93
PA3285_at	4.72	4.79	4.56	4.51	0.22	0.93
PA3288_at	3.61	3.62	3.82	3.82	-0.20	0.93
PA3309_at	8.74	9.08	9.34	9.07	-0.29	0.93
PA3335_at	4.15	3.77	4.23	4.31	-0.31	0.93
PA3382_phnE_at	2.99	2.83	3.08	3.18	-0.22	0.93
PA3389_at	3.98	3.77	3.52	3.72	0.25	0.93
PA3391_nosR_at	4.25	4.61	4.21	4.04	0.31	0.93
PA3397_fpr_at	4.17	4.15	4.38	4.36	-0.21	0.93
PA3414_at	4.38	4.56	3.97	4.39	0.29	0.93
PA3433_at	2.79	2.72	3.22	2.85	-0.28	0.93
PA3436_at	3.62	3.71	3.97	3.83	-0.24	0.93
PA3437_at	4.01	4.00	4.23	4.19	-0.20	0.93
PA3450_at	4.47	4.00	4.44	4.66	-0.31	0.93
PA3531_bfrB_at	4.71	5.13	4.67	4.54	0.31	0.93
PA3532_at	4.86	4.40	4.21	4.41	0.31	0.93

PA3534_at	3.49	3.54	3.15	3.37	0.25	0.93
PA3544_algE_at	2.81	2.60	2.45	2.48	0.24	0.93
PA3582_glpK_at	5.85	5.52	5.88	6.12	-0.31	0.93
PA3604_at	5.06	5.15	4.80	4.94	0.24	0.93
PA3615_at	5.11	5.21	5.30	5.48	-0.23	0.93
PA3625_surE_at	5.61	6.10	6.11	6.32	-0.36	0.93
PA3642_rnhB_at	3.99	4.48	3.85	4.00	0.31	0.93
PA3664_at	3.53	3.64	3.38	3.37	0.21	0.93
PA3689_at	3.32	3.43	3.12	3.22	0.21	0.93
PA3690_at	4.58	4.76	4.43	4.42	0.24	0.93
PA3692_at	5.97	6.45	5.75	5.98	0.34	0.93
PA3704_at	3.13	3.13	3.51	3.23	-0.24	0.93
PA3746_ffh_at	5.44	5.35	5.21	5.18	0.20	0.93
PA3755_at	3.79	3.47	3.36	3.37	0.27	0.93
PA3762_at	4.32	4.53	4.18	4.19	0.24	0.93
PA3784_at	2.74	2.80	3.24	2.88	-0.29	0.93
PA3791_at	4.61	4.45	4.74	4.76	-0.22	0.93
PA3815_at	8.09	9.24	7.97	8.19	0.59	0.93
PA3859_at	5.76	5.70	5.83	6.09	-0.23	0.93
PA3860_at	3.85	3.36	3.38	3.05	0.39	0.93
PA3862_at	3.68	4.67	3.63	3.57	0.57	0.93
PA3956_at	4.48	4.77	4.95	4.82	-0.26	0.93
PA3977_hemL_at	5.22	5.30	5.33	5.89	-0.35	0.93
PA3982_at	5.26	5.86	5.07	5.32	0.37	0.93
PA3996_lipA_at	3.75	4.02	3.72	3.54	0.26	0.93
PA4003_pbpA_at	3.59	3.28	3.12	3.24	0.26	0.93
PA4029_at	3.19	3.24	2.98	3.01	0.22	0.93
PA4053_ribE_at	4.96	5.13	4.75	4.85	0.25	0.93
PA4079_at	4.88	5.03	5.14	5.23	-0.23	0.93
PA4090_at	3.94	3.68	3.45	3.62	0.28	0.93
PA4101_at	2.98	2.80	2.69	2.64	0.22	0.93
PA4122_at	3.46	3.27	3.74	3.50	-0.26	0.93
PA4130_at	6.23	6.11	6.32	6.53	-0.26	0.93
PA4139_at	2.73	2.69	2.94	2.91	-0.21	0.93
PA4181_at	3.66	3.26	3.82	3.67	-0.29	0.93
PA4187_at	3.26	3.31	3.65	3.43	-0.25	0.93
PA4197_at	4.45	5.44	4.49	4.38	0.51	0.93
PA4281_sbcD_at	3.64	3.70	3.25	3.55	0.27	0.93
PA4283_recD_at	3.23	3.34	3.48	3.51	-0.21	0.93
PA4289_at	4.34	4.52	4.20	4.15	0.25	0.93
PA4290_at	2.62	2.56	2.74	2.90	-0.23	0.93
PA4320_at	3.27	3.08	2.97	2.93	0.23	0.93
PA4326_at	3.69	4.32	3.49	3.74	0.39	0.93
PA4330_at	3.10	2.89	3.28	3.18	-0.23	0.93

PA4344_at	3.30	3.27	3.88	3.37	-0.34	0.93
PA4377_at	4.94	5.43	4.85	4.91	0.30	0.93
PA4387_at	3.62	4.14	3.66	3.36	0.37	0.93
PA4389_at	4.37	4.32	4.22	3.98	0.24	0.93
PA4456_at	4.69	4.86	4.54	4.54	0.23	0.93
PA4457_at	4.59	5.06	4.60	4.35	0.34	0.93
PA4462_rpoN_at	3.33	3.41	3.19	3.08	0.23	0.93
PA4491_at	5.34	5.64	5.76	5.78	-0.28	0.93
PA4503_at	5.06	4.81	5.16	5.21	-0.25	0.93
PA4507_at	3.37	3.47	3.27	3.15	0.21	0.93
PA4524_nadC_at	3.53	3.12	3.07	3.03	0.28	0.93
PA4534_at	3.10	2.87	3.12	3.47	-0.31	0.93
PA4538_ndh_at	4.45	3.93	4.69	4.40	-0.35	0.93
PA4571_at	5.25	4.98	5.30	5.44	-0.26	0.93
PA4584_at	4.15	4.13	3.90	3.98	0.20	0.93
PA4597_oprJ_at	4.05	3.71	4.16	4.14	-0.27	0.93
PA4636_at	4.41	4.30	4.11	4.18	0.21	0.93
PA4644_at	3.03	2.83	3.12	3.18	-0.22	0.93
PA4716_at	3.44	3.45	3.50	4.05	-0.33	0.93
PA4740_pnp_at	2.66	3.36	3.20	3.75	-0.46	0.93
PA4755_greA_at	3.90	3.61	4.13	3.95	-0.28	0.93
PA4773_at	3.32	3.05	2.88	2.99	0.25	0.93
PA4784_at	3.61	3.48	3.38	3.29	0.21	0.93
PA4821_at	3.76	3.55	3.92	3.86	-0.24	0.93
PA4823_at	2.88	2.96	2.79	2.55	0.25	0.93
PA4840_at	4.28	4.22	4.10	3.93	0.23	0.93
PA4863_at	3.32	3.34	3.00	3.20	0.23	0.93
PA4867_ureB_at	2.15	2.17	2.53	2.31	-0.26	0.93
PA4908_at	4.75	4.43	4.81	4.90	-0.26	0.93
PA4916_at	3.63	3.52	3.39	3.35	0.21	0.93
PA4918_at	7.00	7.22	6.80	6.94	0.24	0.93
PA4943_at	6.22	6.95	6.16	6.13	0.44	0.93
PA4945_miaA_at	3.95	4.25	4.49	4.30	-0.29	0.93
PA4955_at	3.48	4.00	3.57	3.19	0.36	0.93
PA4974_at	3.69	4.02	3.59	3.61	0.26	0.93
PA4978_at	3.44	3.06	2.93	2.97	0.30	0.93
PA4988_waaA_at	4.66	4.37	4.19	4.29	0.28	0.93
PA5019_at	3.74	3.88	4.01	4.07	-0.23	0.93
PA5028_at	5.66	5.72	5.43	5.49	0.23	0.93
PA5050_priA_at	3.20	3.11	2.96	2.91	0.22	0.93
PA5054_hslU_at	6.22	6.69	5.96	6.26	0.34	0.93
PA5057_phaD_at	2.64	2.63	2.45	2.42	0.20	0.93
PA5067_hisE_at	5.09	5.92	5.08	4.95	0.49	0.93
PA5095_at	5.93	5.61	5.39	5.55	0.30	0.93

PA5100_hutU_at	3.68	3.91	4.09	4.01	-0.26	0.93
PA5101_at	3.70	3.58	3.79	4.03	-0.27	0.93
PA5124_ntrB_at	2.93	3.14	3.22	3.39	-0.27	0.93
PA5145_at	3.05	2.97	3.30	3.15	-0.21	0.93
PA5156_at	5.38	5.00	4.96	4.86	0.28	0.93
PA5157_at	3.35	4.07	3.31	3.28	0.41	0.93
PA5167_at	4.22	4.22	4.43	4.42	-0.20	0.93
PA5181_at	4.21	4.10	4.47	4.31	-0.23	0.93
PA5201_at	4.35	4.58	4.25	4.20	0.24	0.93
PA5213_gcvP1_at	4.76	4.43	4.79	5.00	-0.30	0.93
PA5214_gcvH1_at	4.59	4.73	4.76	5.18	-0.32	0.93
PA5218_at	3.40	3.32	3.59	3.57	-0.22	0.93
PA5224_pepP_at	4.33	4.28	4.07	4.10	0.22	0.93
PA5226_at	4.72	4.59	4.51	4.34	0.23	0.93
PA5229_at	5.57	6.02	6.06	6.12	-0.30	0.93
PA5254_at	3.35	3.14	3.04	2.99	0.23	0.93
PA5255_algQ_at	6.99	6.89	6.82	6.53	0.26	0.93
PA5268_corA_at	2.88	2.89	3.03	3.22	-0.24	0.93
PA5286_at	3.71	3.82	3.95	4.00	-0.21	0.93
PA5303_at	3.25	3.73	3.78	3.80	-0.30	0.93
PA5309_at	3.86	3.93	3.55	3.77	0.23	0.93
PA5376_at	2.70	2.63	2.92	2.87	-0.23	0.93
PA5389_at	2.93	2.92	3.16	3.14	-0.23	0.93
PA5390_at	3.55	3.08	2.88	3.11	0.32	0.93
PA5428_at	5.04	5.10	5.32	5.24	-0.21	0.93
PA5485_at	4.66	4.73	4.48	4.45	0.23	0.93
PA5499_np20_at	6.82	6.94	7.02	7.20	-0.23	0.93
PA5503_at	4.34	4.52	4.21	4.15	0.24	0.93
PA5522_at	3.77	4.53	3.63	3.81	0.43	0.93
PA5541_at	2.78	2.57	3.06	2.83	-0.27	0.93
PA5564_gidB_at	5.16	5.37	5.02	5.05	0.23	0.93
PA5568_at	7.58	7.97	7.42	7.57	0.28	0.93
Pae_AF035937cds4_at	2.27	2.01	2.36	2.38	-0.23	0.93
Pae_AF147795cds2_at	2.16	2.27	2.54	2.37	-0.24	0.93
Pae_AF191564cds4_at	3.14	3.02	3.57	3.20	-0.30	0.93
Pae_M14850cds_at	2.25	2.04	2.66	2.25	-0.31	0.93
Pae_M29695cds2_at	2.31	2.03	2.41	2.41	-0.24	0.93
Pae_orfJ_at	2.58	2.34	2.16	2.23	0.26	0.93
Pae_orfN_at	2.50	2.52	2.82	2.65	-0.22	0.93
Pae_tRNA_Val_f_	6.45	6.85	6.27	6.41	0.31	0.93

at						
PA4551_pilV_at	4.51	4.58	4.73	4.75	-0.20	0.94
PA1946_rbsB_at	5.23	5.66	5.17	5.17	0.28	0.94
PA3342_at	3.61	3.02	3.64	3.66	-0.33	0.94
PA4539_at	2.73	3.05	2.60	2.69	0.25	0.94
PA4574_at	4.19	4.18	4.05	3.91	0.20	0.94
PA0051_at	3.60	3.25	3.62	3.74	-0.26	0.94
PA0276_at	3.54	3.68	3.41	3.40	0.20	0.94
PA0359_at	4.98	5.41	5.52	5.42	-0.28	0.94
PA0502_at	3.63	4.00	4.11	4.04	-0.26	0.94
PA0713_at	7.54	7.81	7.88	7.94	-0.23	0.94
PA0736_at	3.84	3.44	3.10	3.52	0.33	0.94
PA0831_oruR_at	4.83	5.00	4.67	4.74	0.21	0.94
PA1389_at	3.12	2.83	3.15	3.29	-0.24	0.94
PA1706_pcrV_at	5.55	6.82	6.87	6.73	-0.62	0.94
PA1947_rbsA_at	3.41	3.80	3.41	3.24	0.28	0.94
PA2308_at	3.51	2.93	3.64	3.48	-0.34	0.94
PA2489_at	3.47	3.24	3.24	2.95	0.26	0.94
PA2848_at	3.20	3.06	3.01	2.79	0.23	0.94
PA2853_oprI_at	10.15	10.82	10.78	10.93	-0.37	0.94
PA2890_at	4.35	3.85	4.32	4.51	-0.31	0.94
PA3120_leuD_at	3.67	3.76	3.91	3.91	-0.20	0.94
PA3149_wbpH_at	5.92	5.42	5.44	5.28	0.31	0.94
PA3244_minD_at	6.20	6.70	6.11	6.18	0.30	0.94
PA3275_at	4.97	4.48	4.62	4.00	0.41	0.94
PA3369_at	3.22	3.62	3.14	3.16	0.27	0.94
PA3691_at	6.60	7.52	6.56	6.63	0.46	0.94
PA3911_at	6.95	7.05	6.93	6.52	0.27	0.94
PA4481_mreB_at	6.15	7.21	6.08	6.23	0.53	0.94
PA5195_at	3.59	3.80	3.93	3.90	-0.22	0.94
PA5250_at	3.49	3.33	3.20	3.21	0.21	0.94
PA5266_at	3.02	2.69	3.11	3.08	-0.24	0.94
PA5446_i_at	9.52	10.29	10.16	10.53	-0.44	0.94
PA0375_ftsX_at	5.16	5.39	4.86	5.17	0.26	0.94
PA3659_at	5.22	4.23	5.12	5.33	-0.50	0.94
AFFX-Bsubtilis_lys_at	2.09	2.31	2.48	2.37	-0.22	0.94
PA0367_at	2.28	2.66	2.20	2.22	0.26	0.94
PA2523_at	4.24	4.08	4.22	4.68	-0.29	0.94
PA4591_at	3.14	2.76	3.06	3.47	-0.31	0.94
Pae_AF147795cds8_at	2.40	2.30	2.54	2.56	-0.20	0.94
PA1036_at	3.31	3.83	3.93	3.83	-0.31	0.94
PA1103_at	4.53	5.15	4.39	4.59	0.35	0.94
PA1202_at	4.59	4.77	4.57	4.32	0.24	0.94

PA1437_at	4.71	4.69	4.52	4.50	0.19	0.94
PA1668_at	4.37	3.95	4.43	4.42	-0.27	0.94
PA2379_at	3.29	3.25	3.38	3.59	-0.21	0.94
PA2873_at	2.75	2.48	2.30	2.46	0.24	0.94
PA4039_at	2.88	2.66	2.56	2.55	0.21	0.94
PA4383_at	5.08	5.20	5.33	5.34	-0.20	0.94
PA4606_at	6.41	6.50	6.69	6.61	-0.20	0.94
PA4969_at	3.80	3.78	3.93	4.05	-0.20	0.94
PA5068_tatA_at	4.70	4.66	4.42	4.55	0.20	0.94
PA5160_at	3.00	2.69	3.02	3.16	-0.24	0.94
PA5164_rmlC_at	4.02	4.50	4.44	4.71	-0.31	0.94
PA5215_gcvT1_at	5.23	4.89	5.34	5.27	-0.25	0.94
PA1222_at	3.36	3.22	2.99	3.16	0.21	0.94
PA4877_at	4.58	4.45	4.27	4.35	0.20	0.94
ig_3527428_3526677_at	5.46	5.40	5.28	5.18	0.20	0.94
PA0890_aotM_at	4.07	4.23	4.04	3.81	0.23	0.94
PA1405_at	3.07	2.90	3.18	3.20	-0.20	0.94
PA1745_at	3.89	3.54	4.02	3.91	-0.25	0.94
PA2911_at	2.84	2.65	2.83	3.16	-0.26	0.94
PA3821_secD_at	4.78	5.02	5.05	5.20	-0.23	0.94
PA4738_at	5.06	4.85	4.53	4.87	0.26	0.94
PA4952_at	5.74	5.34	5.28	5.28	0.26	0.94
Pae_AF241171cds43_s_at	2.05	2.08	2.28	2.23	-0.19	0.94
PA1549_at	4.38	4.06	4.43	4.49	-0.24	0.94
PA2149_at	3.65	3.72	3.55	3.41	0.20	0.94
PA3672_at	4.52	4.50	4.31	4.34	0.19	0.94
PA3770_guaB_at	4.73	5.50	4.63	4.80	0.40	0.94
PA4581_rtcR_at	3.17	3.04	2.92	2.89	0.20	0.94
PA0382_micA_at	3.41	3.32	3.23	3.08	0.20	0.94
PA3786_at	4.21	4.08	3.99	3.90	0.20	0.94
Pae_tRNA_Gln_s_at	7.47	8.23	7.49	7.43	0.39	0.94
PA2875_at	4.83	4.96	4.78	4.57	0.22	0.95
PA1859_at	2.46	2.46	2.20	2.33	0.20	0.95
PA2191_exoY_at	4.88	5.36	5.35	5.47	-0.29	0.95
PA1580_gltA_at	5.01	5.52	5.61	5.52	-0.30	0.95
PA1775_at	4.63	5.32	4.61	4.61	0.36	0.95
PA2471_at	2.39	2.44	2.56	2.65	-0.19	0.95
PA3310_at	3.29	2.95	2.96	2.77	0.26	0.95
ig_1087843_1087095_at	3.86	3.74	4.13	3.90	-0.22	0.95
PA0607_rpe_at	3.81	3.60	3.85	4.00	-0.22	0.95
PA0700_at	4.62	4.19	4.63	4.73	-0.27	0.95

PA1634_kdpB_at	3.59	3.59	3.46	3.34	0.19	0.95
PA2124_at	3.71	3.40	3.81	3.76	-0.23	0.95
PA2143_at	3.23	2.71	2.67	2.67	0.30	0.95
PA2568_at	4.72	5.21	4.77	4.56	0.30	0.95
PA3811_hscB_at	3.89	4.56	3.79	3.95	0.36	0.95
PA4332_at	2.88	2.80	2.59	2.70	0.20	0.95
PA4505_at	4.38	4.24	4.70	4.40	-0.24	0.95
PA0526_at	5.67	5.98	5.58	5.61	0.23	0.95
PA0574_at	3.56	3.49	3.32	3.35	0.19	0.95
PA2311_r_at	4.72	4.41	4.72	4.91	-0.25	0.95
PA2939_at	2.87	2.74	3.05	2.95	-0.20	0.95
PA4317_at	4.99	5.41	5.42	5.51	-0.27	0.95
PA4857_at	2.89	2.61	2.52	2.54	0.22	0.95
PA5527_at	5.12	5.41	5.00	5.07	0.23	0.95
PA0942_at	6.35	5.97	6.45	6.38	-0.25	0.95
PA1223_at	3.58	3.45	3.38	3.23	0.21	0.95
PA3146_wbpK_at	4.71	4.71	4.58	4.46	0.19	0.95
PA1009_at	3.41	3.67	3.28	3.37	0.22	0.95
PA1093_at	6.64	7.10	6.60	6.60	0.27	0.95
PA5504_at	2.97	2.92	2.79	2.72	0.19	0.95
PA0025_aroE_at	2.85	2.96	2.98	3.31	-0.24	0.95
PA0128_at	5.00	5.17	4.98	4.75	0.22	0.95
PA0361_at	3.39	3.25	3.12	3.13	0.20	0.95
PA1926_at	3.07	2.98	3.10	3.40	-0.23	0.95
PA2163_at	3.01	2.70	2.99	3.21	-0.25	0.95
PA4448_hisD_at	3.07	3.07	3.39	3.17	-0.21	0.95
Pae_AF035937cds5_at	2.90	2.68	3.00	2.99	-0.21	0.95
PA1316_at	3.00	2.88	3.11	3.15	-0.19	0.96
PA1429_at	5.56	5.75	5.87	5.85	-0.20	0.96
PA3184_at	3.41	3.33	3.47	3.68	-0.21	0.96
ig_4888194_4889111_at	2.65	2.47	2.79	2.73	-0.20	0.96
PA0876_at	4.27	4.94	4.86	5.06	-0.36	0.96
PA1025_at	3.07	2.64	3.20	3.05	-0.27	0.96
PA1773_at	5.55	6.04	5.55	5.48	0.28	0.96
PA1997_at	3.70	3.81	4.06	3.87	-0.21	0.96
PA2027_at	3.31	3.04	3.32	3.50	-0.23	0.96
PA2029_i_at	3.23	2.93	3.30	3.31	-0.23	0.96
PA5106_at	4.94	4.99	5.11	5.19	-0.19	0.96
PA5290_at	3.45	3.62	3.74	3.72	-0.20	0.96
PA5482_at	3.58	3.99	4.02	4.06	-0.26	0.96
PA5512_at	3.20	3.25	3.04	3.05	0.18	0.96
PA0090_at	3.91	3.61	3.60	3.45	0.23	0.96
PA1720_pscG_at	4.81	5.03	5.06	5.21	-0.22	0.96

PA3257_prc_at	6.41	6.50	6.22	6.31	0.19	0.96
PA4385_groEL_at	8.50	9.01	8.94	9.17	-0.30	0.96
PA0716_at	2.33	2.06	2.03	1.92	0.22	0.96
PA0997_at	5.16	5.37	5.51	5.43	-0.21	0.96
PA1333_r_at	5.34	5.83	5.72	6.09	-0.32	0.96
PA1404_at	3.81	3.82	3.60	3.67	0.18	0.96
PA2290_gcd_at	4.81	4.78	4.99	4.96	-0.18	0.96
PA3720_at	2.92	3.18	2.72	2.92	0.23	0.96
PA3880_at	6.70	7.58	6.72	6.71	0.42	0.96
PA5127_at	2.53	2.49	2.28	2.37	0.19	0.96
PA5347_at	2.66	2.58	2.93	2.72	-0.21	0.96
PA3816_cysE_at	5.45	5.74	5.39	5.36	0.22	0.96
PA3306_at	5.33	5.98	5.02	5.50	0.39	0.96
PA5211_at	4.34	4.73	4.74	4.84	-0.25	0.96
PA4816_at	3.67	3.63	3.55	3.35	0.20	0.96
PA4430_at	4.35	5.07	5.05	5.10	-0.36	0.97
PA4522_ampD_at	3.88	3.34	3.42	3.17	0.31	0.97
PA4822_at	3.05	2.95	3.09	3.32	-0.21	0.97
ig_1064555_1063544_at	2.80	2.70	2.58	2.56	0.18	0.97
ig_1117390_1118158_at	4.19	3.91	3.87	3.80	0.21	0.97
ig_1996806_1997509_at	3.73	3.84	3.93	3.99	-0.17	0.97
ig_2068728_2069490_at	3.19	3.26	3.38	3.43	-0.18	0.97
ig_2240302_2239267_at	2.75	2.59	2.50	2.47	0.18	0.97
ig_4241341_4240578_at	4.07	3.80	3.86	3.54	0.24	0.97
ig_4956028_4956733_at	2.50	2.60	2.73	2.74	-0.18	0.97
ig_5541409_5542072_at	2.86	3.05	2.98	3.42	-0.25	0.97
PA0037_trpI_at	3.62	3.45	3.27	3.42	0.19	0.97
PA0038_at	6.71	7.22	6.67	6.75	0.25	0.97
PA0040_s_at	3.14	3.01	2.89	2.91	0.18	0.97
PA0071_at	3.63	3.74	3.85	3.86	-0.17	0.97
PA0107_at	3.18	2.85	3.09	3.45	-0.25	0.97
PA0135_at	2.56	2.51	2.36	2.38	0.17	0.97
PA0168_at	5.04	5.24	4.89	5.02	0.19	0.97
PA0201_at	4.58	4.28	4.04	4.32	0.25	0.97
PA0213_at	2.72	2.54	3.04	2.67	-0.23	0.97
PA0238_at	3.76	3.44	3.73	3.91	-0.22	0.97
PA0283_sbp_at	4.53	3.92	4.65	4.44	-0.32	0.97
PA0296_s_at	4.21	4.52	4.58	4.57	-0.21	0.97

PA0329_at	7.06	7.46	6.94	7.10	0.24	0.97
PA0331_ilvA1_at	3.67	3.82	3.65	3.46	0.19	0.97
PA0342_thyA_at	5.59	5.94	5.51	5.58	0.22	0.97
PA0422_at	2.96	3.14	2.74	2.95	0.21	0.97
PA0519_nirS_at	8.04	8.53	8.51	8.56	-0.25	0.97
PA0523_norC_at	6.59	7.37	6.62	6.66	0.34	0.97
PA0524_norB_at	4.65	5.90	4.57	4.88	0.55	0.97
PA0564_at	3.04	3.24	3.01	2.87	0.20	0.97
PA0567_i_at	3.12	3.22	3.01	2.97	0.17	0.97
PA0577_dnaG_at	4.42	5.27	4.31	4.59	0.40	0.97
PA0585_at	4.76	4.62	4.91	4.84	-0.19	0.97
PA0595_ostA_at	4.94	5.75	4.92	5.04	0.37	0.97
PA0604_at	4.27	4.16	4.51	4.30	-0.19	0.97
PA0608_at	5.34	5.13	5.41	5.43	-0.18	0.97
PA0696_at	2.65	2.46	2.78	2.72	-0.20	0.97
PA0730_at	3.29	3.38	3.63	3.41	-0.19	0.97
PA0772_recO_at	3.64	3.69	3.32	3.59	0.21	0.97
PA0829_at	3.57	2.78	2.77	2.84	0.37	0.97
PA0857_bolA_at	4.32	4.38	4.16	4.20	0.17	0.97
PA0873_phhR_at	3.54	3.49	3.55	3.91	-0.22	0.97
PA0888_aotJ_at	5.11	5.48	5.20	4.89	0.24	0.97
PA0902_at	3.16	3.20	2.97	3.04	0.18	0.97
PA0943_at	4.27	5.20	4.16	4.46	0.42	0.97
PA0949_wrbA_at	4.11	4.76	4.18	4.10	0.30	0.97
PA0965_ruvC_at	3.53	3.88	3.28	3.59	0.27	0.97
PA1032_at	4.01	3.85	4.15	4.08	-0.18	0.97
PA1053_at	7.24	7.63	7.57	7.78	-0.24	0.97
PA1060_at	3.54	3.47	3.26	3.40	0.18	0.97
PA1081_flgF_at	2.86	2.68	3.01	2.93	-0.20	0.97
PA1083_flgH_at	5.73	5.85	6.03	5.93	-0.19	0.97
PA1088_at	3.55	3.36	3.30	3.23	0.19	0.97
PA1100_fliE_at	5.48	6.16	5.48	5.54	0.32	0.97
PA1123_at	3.68	3.84	3.96	3.93	-0.19	0.97
PA1137_at	3.48	3.77	3.26	3.54	0.23	0.97
PA1148_toxA_at	3.74	3.76	3.67	3.49	0.17	0.97
PA1149_at	3.19	3.02	2.95	2.92	0.17	0.97
PA1167_at	4.03	3.73	3.66	3.68	0.21	0.97
PA1192_at	3.87	3.87	3.74	3.66	0.17	0.97
PA1199_at	3.75	4.05	3.45	3.83	0.26	0.97
PA1231_at	3.24	2.97	3.45	3.21	-0.23	0.97
PA1237_at	3.42	3.21	3.19	3.04	0.20	0.97
PA1245_at	4.57	4.51	4.98	4.59	-0.25	0.97
PA1254_at	2.87	2.71	2.92	3.06	-0.20	0.97
PA1259_at	3.25	2.97	3.34	3.31	-0.22	0.97

PA1262_at	3.57	3.25	3.09	3.25	0.23	0.97
PA1282_at	2.75	2.40	2.26	2.43	0.23	0.97
PA1293_at	4.90	4.62	4.51	4.60	0.21	0.97
PA1295_at	2.78	2.76	2.61	2.57	0.18	0.97
PA1312_at	2.94	2.73	2.99	3.08	-0.20	0.97
PA1327_at	4.18	4.07	4.40	4.25	-0.20	0.97
PA1330_at	3.42	3.50	3.66	3.62	-0.18	0.97
PA1338_ggt_at	3.45	3.36	3.55	3.60	-0.17	0.97
PA1343_at	3.39	3.26	3.18	3.10	0.19	0.97
PA1360_at	2.88	2.93	2.68	2.77	0.18	0.97
PA1394_at	2.52	2.67	2.37	2.46	0.18	0.97
PA1445_fliO_at	2.43	2.73	2.91	2.71	-0.23	0.97
PA1453_flhF_at	5.39	5.87	5.30	5.41	0.28	0.97
PA1466_at	2.66	2.63	2.80	2.84	-0.18	0.97
PA1477_ccmC_at	4.50	4.53	4.72	4.65	-0.18	0.97
PA1489_at	3.48	3.24	3.22	3.06	0.22	0.97
PA1529_lig_at	4.48	4.90	4.23	4.60	0.28	0.97
PA1531_at	2.39	2.41	2.54	2.60	-0.17	0.97
PA1532_dnaX_at	4.64	5.67	4.68	4.73	0.45	0.97
PA1539_at	4.09	4.07	4.62	4.07	-0.27	0.97
PA1545_at	5.04	5.81	5.18	4.99	0.34	0.97
PA1571_at	3.50	3.69	3.25	3.53	0.21	0.97
PA1583_sdhA_at	5.52	6.47	6.46	6.40	-0.44	0.97
PA1622_at	5.32	5.27	5.54	5.42	-0.18	0.97
PA1636_kdpD_at	3.83	3.50	3.77	4.03	-0.23	0.97
PA1649_at	2.62	2.65	2.54	2.34	0.20	0.97
PA1654_at	4.35	4.47	4.25	4.19	0.19	0.97
PA1669_at	3.53	3.36	3.67	3.59	-0.18	0.97
PA1699_at	4.31	5.10	5.10	5.03	-0.36	0.97
PA1778_cobA_at	3.90	3.41	3.98	3.85	-0.26	0.97
PA1784_at	3.15	3.00	3.26	3.27	-0.19	0.97
PA1786_at	3.26	3.05	3.30	3.38	-0.19	0.97
PA1831_at	3.34	3.23	3.09	3.11	0.19	0.97
PA1838_cysI_at	4.10	3.25	4.02	4.10	-0.38	0.97
PA1865_at	4.30	3.75	3.72	3.79	0.27	0.97
PA1875_at	3.41	3.37	3.23	3.20	0.17	0.97
PA1924_at	2.89	2.73	3.05	2.95	-0.19	0.97
PA1936_at	3.38	3.00	3.46	3.39	-0.24	0.97
PA1943_at	3.00	3.06	3.17	3.22	-0.17	0.97
PA1959_bacA_at	4.93	5.25	4.97	4.74	0.24	0.97
PA1996_ppiC1_at	3.06	2.93	2.85	2.77	0.18	0.97
PA2000_at	2.74	2.54	2.85	2.82	-0.19	0.97
PA2016_at	7.40	7.26	7.16	7.14	0.18	0.97
PA2019_at	4.57	5.34	4.67	4.54	0.35	0.97

PA2031_i_at	3.83	3.86	4.12	3.94	-0.18	0.97
PA2039_at	2.47	2.25	2.58	2.51	-0.19	0.97
PA2055_at	2.78	2.67	2.85	2.95	-0.17	0.97
PA2106_at	3.28	3.40	3.12	3.21	0.17	0.97
PA2109_at	2.92	2.97	3.13	3.10	-0.18	0.97
PA2142_at	3.27	3.12	3.50	3.30	-0.20	0.97
PA2151_at	2.80	2.60	2.91	2.86	-0.18	0.97
PA2234_at	2.45	2.46	2.73	2.54	-0.18	0.97
PA2326_at	3.19	3.19	3.14	2.81	0.22	0.97
PA2342_mtlD_at	3.12	2.58	3.28	3.02	-0.30	0.97
PA2374_at	2.70	2.33	2.72	2.79	-0.24	0.97
PA2413_at	3.39	3.18	3.22	2.93	0.21	0.97
PA2421_at	2.81	2.64	2.96	2.87	-0.19	0.97
PA2424_at	3.08	2.76	3.17	3.10	-0.21	0.97
PA2436_at	6.40	6.64	6.71	6.71	-0.19	0.97
PA2437_at	3.79	3.37	3.69	4.01	-0.27	0.97
PA2473_at	3.28	3.12	3.06	2.98	0.18	0.97
PA2488_at	4.73	4.63	4.50	4.52	0.17	0.97
PA2506_at	2.73	2.86	2.58	2.65	0.18	0.97
PA2540_at	3.41	3.82	3.33	3.44	0.23	0.97
PA2552_at	3.58	3.25	3.92	3.47	-0.28	0.97
PA2553_at	3.47	3.33	3.63	3.53	-0.19	0.97
PA2566_at	4.27	3.96	4.20	4.48	-0.23	0.97
PA2584_pgsA_at	5.51	5.69	5.40	5.41	0.19	0.97
PA2600_at	2.95	2.95	3.10	3.14	-0.17	0.97
PA2624_idh_at	5.32	5.58	5.66	5.63	-0.19	0.97
PA2640_nuoE_at	3.04	3.38	3.38	3.49	-0.23	0.97
PA2686_pfeR_at	2.10	2.05	2.25	2.26	-0.18	0.97
PA2689_at	3.54	3.27	3.20	3.20	0.20	0.97
PA2697_at	3.37	3.18	3.67	3.35	-0.24	0.97
PA2747_at	2.71	2.80	2.99	2.89	-0.19	0.97
PA2763_at	3.20	2.91	2.79	2.90	0.20	0.97
PA2776_at	4.71	5.13	4.80	4.48	0.28	0.97
PA2793_at	3.34	3.16	3.12	2.99	0.20	0.97
PA2797_at	3.51	3.51	3.23	3.41	0.19	0.97
PA2820_at	3.29	4.16	3.27	3.43	0.38	0.97
PA2847_at	3.81	3.53	3.56	3.28	0.25	0.97
PA2859_greB_at	2.93	2.93	3.17	3.05	-0.18	0.97
PA2885_at	2.52	2.64	2.44	2.33	0.19	0.97
PA2909_i_at	4.55	3.83	4.77	4.35	-0.38	0.97
PA2916_at	2.88	2.91	2.71	2.75	0.16	0.97
PA2919_at	2.66	2.51	2.74	2.77	-0.17	0.97
PA2958_at	3.08	3.25	2.96	2.99	0.19	0.97
PA2975_rluC_at	4.27	4.60	4.67	4.65	-0.22	0.97

PA2989_at	3.41	3.43	3.00	3.37	0.24	0.97
PA2999_nqrA_at	3.67	4.26	4.21	4.30	-0.29	0.97
PA3015_at	3.90	3.76	3.64	3.67	0.18	0.97
PA3023_at	3.50	3.64	3.70	3.82	-0.19	0.97
PA3159_wbpA_at	4.62	4.90	4.54	4.60	0.19	0.97
PA3160_wzz_at	3.90	4.13	3.83	3.80	0.20	0.97
PA3164_at	4.49	4.39	4.50	4.81	-0.21	0.97
PA3200_at	2.85	3.06	2.86	2.62	0.22	0.97
PA3208_at	3.12	3.04	2.94	2.87	0.17	0.97
PA3221_csaA_at	4.41	3.93	4.33	4.54	-0.26	0.97
PA3234_at	2.81	2.52	2.86	2.86	-0.20	0.97
PA3276_at	3.57	3.35	3.23	3.30	0.20	0.97
PA3283_at	2.97	3.13	2.94	2.79	0.19	0.97
PA3314_at	4.00	4.29	4.31	4.43	-0.22	0.97
PA3326_at	7.22	6.85	7.32	7.21	-0.23	0.97
PA3334_at	4.79	4.61	5.05	4.77	-0.21	0.97
PA3340_at	4.29	4.51	4.12	4.27	0.21	0.97
PA3348_at	5.80	6.10	6.06	6.30	-0.23	0.97
PA3362_at	4.67	4.56	4.94	4.70	-0.21	0.97
PA3385_at	8.07	8.83	8.03	8.14	0.36	0.97
PA3427_at	2.90	2.36	2.29	2.39	0.29	0.97
PA3540_algD_at	3.21	2.93	2.85	2.86	0.21	0.97
PA3558_at	3.47	2.90	2.93	2.88	0.28	0.97
PA3566_at	5.36	5.63	5.49	4.93	0.29	0.97
PA3583_glpR_at	4.70	4.79	4.54	4.60	0.17	0.97
PA3605_at	3.32	2.86	3.31	3.38	-0.26	0.97
PA3607_potA_at	3.89	3.78	3.62	3.69	0.18	0.97
PA3623_at	4.82	4.46	4.36	4.46	0.23	0.97
PA3627_at	3.21	3.01	2.81	3.01	0.20	0.97
PA3635_eno_at	5.16	5.36	5.12	5.04	0.18	0.97
PA3636_kdsA_at	3.62	4.06	3.98	4.19	-0.25	0.97
PA3654_pyrH_at	6.17	6.88	6.00	6.32	0.36	0.97
PA3657_map_at	5.52	6.14	5.55	5.51	0.30	0.97
PA3667_at	2.92	3.24	3.25	3.36	-0.22	0.97
PA3674_at	3.74	4.02	4.09	4.05	-0.19	0.97
PA3694_at	3.06	3.05	2.84	2.93	0.17	0.97
PA3727_at	4.05	4.07	3.73	4.00	0.20	0.97
PA3763_purL_at	5.17	5.42	5.05	5.12	0.21	0.97
PA3768_at	4.97	4.72	4.59	4.71	0.20	0.97
PA3779_at	3.82	3.72	3.47	3.68	0.20	0.97
PA3794_at	4.85	5.14	4.74	4.82	0.22	0.97
PA3801_at	5.24	5.68	5.73	5.70	-0.26	0.97
PA3822_at	4.93	5.44	5.52	5.38	-0.27	0.97
PA3847_at	4.05	4.16	3.83	4.00	0.19	0.97

PA3872_narI_at	7.19	7.48	7.16	7.10	0.21	0.97
PA3902_at	6.16	6.86	6.19	6.21	0.32	0.97
PA3912_at	4.62	4.54	4.66	4.93	-0.21	0.97
PA3937_at	2.93	2.82	2.82	2.44	0.24	0.97
PA3945_at	5.49	6.17	6.00	6.38	-0.35	0.97
PA3966_at	6.01	6.55	6.03	6.00	0.27	0.97
PA3973_at	5.17	4.96	4.94	4.78	0.21	0.97
PA3976_thiE_at	3.85	4.36	3.91	3.78	0.26	0.97
PA4001_mltB2_at	3.18	3.45	3.13	3.09	0.21	0.97
PA4002_rodA_at	3.60	3.70	3.54	3.41	0.18	0.97
PA4031_ppa_at	5.68	6.15	6.13	6.21	-0.25	0.97
PA4035_at	3.05	3.07	3.00	2.69	0.22	0.97
PA4058_at	3.46	3.65	3.45	3.22	0.22	0.97
PA4059_at	5.31	5.31	5.20	5.08	0.17	0.97
PA4080_at	2.98	2.79	3.07	3.06	-0.19	0.97
PA4104_at	3.71	3.30	3.70	3.79	-0.24	0.97
PA4115_at	3.72	4.58	3.74	3.81	0.37	0.97
PA4120_at	3.07	3.08	2.95	2.84	0.18	0.97
PA4186_at	2.89	2.89	3.06	3.06	-0.17	0.97
PA4194_at	3.64	3.52	3.83	3.71	-0.19	0.97
PA4268_rpsL_at	6.21	6.88	6.92	6.79	-0.31	0.97
PA4311_at	4.66	5.10	5.32	5.00	-0.28	0.97
PA4329_pykA_at	4.54	4.83	4.81	4.97	-0.20	0.97
PA4337_at	2.56	2.62	2.73	2.80	-0.17	0.97
PA4345_at	3.44	3.23	3.19	3.10	0.19	0.97
PA4349_at	4.78	4.87	5.06	4.96	-0.18	0.97
PA4364_at	4.01	3.84	4.14	4.09	-0.19	0.97
PA4422_at	5.30	5.10	5.00	5.02	0.19	0.97
PA4426_at	3.64	3.64	3.34	3.55	0.20	0.97
PA4519_at	3.17	3.55	3.12	3.13	0.23	0.97
PA4523_at	6.78	7.32	7.37	7.29	-0.28	0.97
PA4576_at	2.77	3.07	3.05	3.26	-0.24	0.97
PA4615_at	2.98	3.33	3.02	2.83	0.23	0.97
PA4641_at	5.33	5.20	5.37	5.52	-0.18	0.97
PA4668_at	4.36	4.47	4.34	4.11	0.19	0.97
PA4691_at	3.96	3.58	3.62	3.46	0.23	0.97
PA4693_pssA_at	4.25	4.32	4.13	4.08	0.18	0.97
PA4701_at	4.90	4.72	4.77	4.39	0.23	0.97
PA4707_at	3.81	3.67	3.61	3.47	0.20	0.97
PA4749_glmM_at	4.43	4.69	4.32	4.40	0.20	0.97
PA4751_ftsH_at	5.64	6.27	5.51	5.74	0.33	0.97
PA4781_at	4.21	4.17	4.40	4.33	-0.17	0.97
PA4791_at	4.93	4.84	4.62	4.80	0.18	0.97
PA4810_fdnI_at	3.25	3.47	3.45	3.69	-0.21	0.97

PA4831_at	3.89	3.71	3.67	3.51	0.21	0.97
PA4868_ureC_at	3.56	3.28	3.57	3.69	-0.21	0.97
PA4872_at	3.65	3.83	4.12	3.79	-0.21	0.97
PA4878_at	4.14	4.44	4.03	4.13	0.21	0.97
PA4879_at	4.52	4.46	4.18	4.43	0.19	0.97
PA4881_at	2.46	2.58	2.28	2.39	0.19	0.97
PA4892_ureF_at	3.97	3.62	3.50	3.62	0.24	0.97
PA4919_pncB1_at	5.17	5.47	5.00	5.18	0.23	0.97
PA4927_at	3.39	3.30	2.97	3.29	0.22	0.97
PA5015_aceE_at	7.55	7.97	7.93	8.06	-0.23	0.97
PA5022_at	3.64	3.66	3.55	3.36	0.20	0.97
PA5042_pilO_at	6.34	7.04	7.07	7.00	-0.34	0.97
PA5069_tatB_at	4.24	4.20	4.07	4.03	0.18	0.97
PA5089_at	3.12	2.70	2.76	2.55	0.25	0.97
PA5137_at	2.53	2.46	2.67	2.66	-0.17	0.97
PA5161_rmlB_at	4.24	4.72	4.17	4.29	0.25	0.97
PA5235_glpT_at	3.02	3.22	3.60	3.14	-0.25	0.97
PA5274_rnk_at	5.35	5.94	5.36	5.37	0.28	0.97
PA5281_at	2.58	2.43	2.69	2.67	-0.18	0.97
PA5329_at	3.61	3.58	3.42	3.43	0.17	0.97
PA5337_rpoZ_at	4.57	5.15	4.44	4.67	0.30	0.97
PA5348_at	5.66	5.64	5.67	6.09	-0.23	0.97
PA5381_at	3.04	2.90	2.84	2.75	0.18	0.97
PA5382_at	3.31	3.04	3.48	3.31	-0.22	0.97
PA5393_at	3.45	3.50	3.61	3.65	-0.16	0.97
PA5412_at	5.74	5.58	5.33	5.58	0.21	0.97
PA5413_ltaA_at	2.92	2.93	2.71	2.80	0.17	0.97
PA5445_at	3.99	3.57	3.94	4.15	-0.26	0.97
PA5473_at	4.11	4.38	3.83	4.18	0.24	0.97
PA5484_at	2.47	3.22	2.48	2.52	0.35	0.97
PA5490_cc4_at	4.91	6.09	5.92	6.05	-0.49	0.97
PA5508_at	3.17	3.07	2.86	3.01	0.18	0.97
PA5516_pdxY_at	3.62	3.27	3.37	2.99	0.26	0.97
PA5519_at	3.49	3.59	3.23	3.47	0.20	0.97
PA5551_at	2.96	2.77	2.68	2.68	0.18	0.97
Pae_AF241171cds19_at	2.76	2.55	2.78	2.97	-0.22	0.97
Pae_AF241171cds42_at	2.99	2.69	3.20	2.92	-0.22	0.97
Pae_orfF_at	2.69	2.37	2.83	2.66	-0.21	0.97
Pae_tRNA_Arg_s_at	3.07	3.46	3.14	2.90	0.24	0.97
PA4444_mltB1_at	4.48	4.20	4.15	4.14	0.19	0.97
PA1325_at	3.35	3.51	3.26	3.26	0.17	0.97

PA3649_at	4.13	4.18	3.99	4.00	0.16	0.97
PA0927_ldhA_at	2.72	2.73	2.88	2.89	-0.16	0.97
PA1168_at	3.00	2.73	3.18	2.97	-0.21	0.97
PA1635_kdpC_at	2.73	2.43	2.72	2.84	-0.20	0.97
PA2456_at	5.09	4.45	4.45	4.50	0.29	0.97
PA2953_at	3.96	4.23	3.79	4.00	0.21	0.97
PA3383_at	2.52	2.54	2.73	2.65	-0.16	0.97
PA3661_at	2.91	2.58	2.90	3.01	-0.21	0.97
PA4025_r_at	3.94	3.80	3.96	4.16	-0.19	0.97
PA1825_at	2.74	2.87	2.64	2.64	0.17	0.97
PA1300_at	3.10	3.15	2.98	2.95	0.16	0.97
PA2073_at	3.33	2.99	3.40	3.33	-0.21	0.97
PA3922_at	2.91	2.89	3.05	3.08	-0.16	0.97
PA0500_bioB_at	4.14	4.08	3.96	3.94	0.16	0.97
PA2502_at	4.07	3.76	4.12	4.11	-0.20	0.97
PA2759_at	2.52	2.54	2.65	2.73	-0.16	0.97
PA2792_at	2.95	2.66	2.64	2.58	0.20	0.97
PA3512_at	3.71	2.92	2.90	3.05	0.34	0.97
PA3632_at	4.86	4.86	5.00	5.03	-0.16	0.97
PA3876_narK2_at	4.95	5.04	4.78	4.88	0.17	0.97
PA0541_at	4.38	4.67	4.65	4.79	-0.20	0.97
PA3920_at	3.64	3.96	3.59	3.61	0.20	0.97
PA5554_atpD_at	5.71	5.87	5.89	6.05	-0.18	0.97
PA4397_panE_at	4.05	3.94	4.22	4.11	-0.17	0.97
PA0897_aruG_at	2.55	2.90	2.52	2.52	0.20	0.97
AFFX-Bsubtilis_dapB_at	6.41	7.00	6.95	7.01	-0.27	0.97
PA0036_trpB_at	3.83	4.30	3.73	3.91	0.24	0.97
PA0134_at	3.85	4.17	3.99	3.49	0.27	0.97
PA0293_at	2.50	2.38	2.33	2.21	0.17	0.97
PA0308_at	3.26	3.44	3.22	3.12	0.18	0.97
PA0491_at	3.90	4.00	4.04	4.21	-0.17	0.97
PA0709_at	3.10	3.11	3.29	3.23	-0.16	0.97
PA0804_at	4.03	3.84	4.09	4.13	-0.17	0.97
PA1047_at	3.40	3.60	3.04	3.49	0.24	0.97
PA1172_napC_at	5.70	5.23	5.65	5.76	-0.24	0.97
PA1464_at	6.76	7.23	6.72	6.79	0.24	0.97
PA1527_at	4.38	4.33	4.17	4.22	0.16	0.97
PA2136_at	2.37	2.33	2.21	2.18	0.16	0.97
PA2248_bkdA2_at	6.35	6.66	6.60	6.85	-0.22	0.97
PA2258_ptxR_at	3.74	3.50	3.55	3.26	0.21	0.97
PA2451_at	4.17	4.25	4.42	4.33	-0.16	0.97
PA2972_at	2.98	2.92	3.04	3.21	-0.17	0.97
PA3217_at	3.68	3.84	3.62	3.56	0.17	0.97

PA3247_at	3.88	4.07	3.65	3.91	0.20	0.97
PA3269_at	2.92	2.73	2.99	3.00	-0.17	0.97
PA3290_at	4.69	4.51	4.94	4.66	-0.20	0.97
PA3311_at	2.82	2.78	2.97	2.94	-0.16	0.97
PA3527_pyrC_at	3.23	3.14	3.08	2.96	0.17	0.97
PA3541_at	2.33	2.18	2.58	2.32	-0.19	0.97
PA3581_glpF_at	7.13	7.32	7.44	7.37	-0.18	0.97
PA3633_at	4.12	4.33	4.15	3.91	0.20	0.97
PA3750_at	3.03	2.72	2.76	2.57	0.21	0.97
PA3978_at	6.50	6.92	6.82	7.09	-0.24	0.97
PA4023_at	2.41	2.49	2.68	2.56	-0.17	0.97
PA4062_at	2.50	2.52	2.41	2.29	0.16	0.97
PA4238_rpoA_at	6.74	7.43	7.24	7.59	-0.33	0.97
PA4252_rplX_at	3.89	4.50	4.38	4.59	-0.29	0.97
PA4485_at	2.70	2.58	2.45	2.51	0.16	0.97
PA4785_at	5.38	5.36	5.23	5.20	0.16	0.97
PA4901_mdlC_at	2.68	2.66	2.58	2.42	0.17	0.97
PA5374_betI_at	4.61	4.92	4.47	4.65	0.21	0.97
PA5463_at	3.96	4.88	4.03	4.05	0.38	0.97
PA5509_at	4.78	4.96	4.98	5.12	-0.18	0.97
PA2719_at	2.99	2.89	3.14	3.07	-0.16	0.97
PA3076_at	3.59	3.58	3.90	3.63	-0.18	0.97
PA0034_at	2.68	2.61	2.44	2.53	0.16	0.97
PA0123_at	3.10	3.12	2.86	3.02	0.17	0.97
PA1986_pqqB_at	4.34	4.20	4.39	4.49	-0.17	0.97
PA4340_at	4.66	4.52	4.52	4.30	0.18	0.97
PA5129_grx_at	5.85	6.00	5.55	5.89	0.21	0.97
PA5372_betA_at	3.68	3.38	3.68	3.76	-0.19	0.97
PA1033_at	3.09	3.00	3.04	3.56	-0.25	0.97
PA3027_at	3.16	3.29	3.37	3.41	-0.16	0.97
PA4865_ureA_at	2.48	3.31	3.02	3.56	-0.40	0.97
PA5563_soj_at	4.71	5.45	4.42	4.99	0.38	0.97
PA0477_at	2.90	2.98	3.09	3.11	-0.16	0.97
PA1082_flgG_at	5.74	6.03	5.64	5.74	0.19	0.98
PA3166_pheA_at	4.08	3.77	3.87	3.53	0.23	0.98
PA1298_i_at	2.44	2.35	2.31	2.14	0.17	0.98
PA1339_at	4.92	4.69	5.03	4.94	-0.18	0.98
PA1565_at	3.40	3.25	3.16	3.17	0.16	0.98
PA1942_at	2.57	2.17	2.53	2.64	-0.22	0.98
PA2370_at	3.64	3.32	3.62	3.73	-0.20	0.98
PA3298_at	3.49	2.72	2.74	2.82	0.32	0.98
PA4149_at	2.65	2.68	2.91	2.75	-0.17	0.98
PA5024_at	3.46	3.24	3.61	3.46	-0.18	0.98
PA5282_at	3.99	3.59	3.88	4.17	-0.24	0.98

PA2336_at	3.52	3.05	3.49	3.54	-0.23	0.98
ig_5458499_5457716_at	3.16	2.70	3.15	3.16	-0.23	0.98
PA0759_at	3.04	2.98	2.79	2.91	0.16	0.98
PA0894_at	4.09	3.69	4.27	3.99	-0.23	0.98
PA2884_at	4.57	4.60	4.29	4.53	0.18	0.98
PA2925_hisM_at	2.86	2.61	3.00	2.85	-0.19	0.98
PA3380_at	2.75	2.48	2.51	2.33	0.19	0.98
PA3773_at	3.87	3.52	3.39	3.58	0.21	0.98
PA0310_at	3.99	4.11	3.92	3.85	0.16	0.98
PA1170_at	3.25	3.20	3.12	3.01	0.16	0.98
PA5288_glnK_at	7.06	7.24	7.30	7.33	-0.17	0.98
ig_2923366_2922568_at	2.77	2.91	2.47	2.81	0.20	0.98
PA0980_at	3.20	3.31	3.12	3.07	0.16	0.98
PA1226_at	3.29	2.96	2.88	2.99	0.20	0.98
PA1509_at	5.88	5.29	5.82	5.89	-0.27	0.98
PA1957_at	2.58	2.53	2.89	2.59	-0.19	0.98
PA2601_at	3.77	3.59	3.45	3.57	0.17	0.98
PA0079_at	3.83	4.00	4.07	4.10	-0.17	0.98
PA1026_at	2.81	2.69	3.07	2.80	-0.19	0.98
PA3406_hasD_at	2.55	2.49	2.64	2.72	-0.16	0.98
ig_785969_785174_at	4.05	5.26	4.16	4.22	0.46	0.98
PA0576_rpoD_at	5.79	6.72	5.70	6.02	0.39	0.98
PA0616_at	5.20	5.83	5.26	5.23	0.27	0.98
PA2396_at	3.41	3.24	3.16	3.17	0.16	0.98
PA3287_at	3.02	2.83	2.87	2.59	0.20	0.98
PA3408_hasR_at	3.14	3.04	3.26	3.23	-0.16	0.98
PA4742_truB_at	4.19	5.35	4.30	4.35	0.45	0.98
PA5500_znuC_at	3.57	4.03	3.63	3.51	0.23	0.98
Pae_AF241171cds11_at	2.66	2.52	2.83	2.68	-0.17	0.99
PA4148_at	3.37	3.17	3.10	3.10	0.17	0.99
PA0225_at	4.06	3.97	3.94	3.74	0.17	0.99
PA1465_at	2.65	2.90	2.53	2.65	0.18	0.99
PA2265_at	4.36	4.79	4.60	5.12	-0.29	0.99
PA2525_at	3.72	3.59	3.70	3.99	-0.19	0.99
ig_2342493_2341640_at	2.77	2.89	2.98	2.99	-0.16	0.99
PA0125_at	3.25	3.35	3.16	3.13	0.15	0.99
PA0136_at	3.94	3.76	3.79	3.54	0.19	0.99
PA0172_at	3.14	3.10	3.25	3.29	-0.15	0.99
PA0252_at	3.25	2.93	2.89	2.90	0.19	0.99
PA0549_at	3.81	3.85	3.66	3.70	0.15	0.99
PA0603_at	3.54	3.96	3.79	4.25	-0.27	0.99

PA0791_at	2.70	2.64	2.89	2.77	-0.16	0.99
PA0879_at	3.13	3.19	3.60	3.16	-0.22	0.99
PA1067_at	2.43	2.43	2.71	2.49	-0.17	0.99
PA1600_at	3.02	2.62	2.59	2.64	0.21	0.99
PA1694_pscQ_at	5.34	5.56	5.62	5.61	-0.17	0.99
PA1850_at	3.57	3.69	3.55	3.38	0.17	0.99
PA1935_at	4.09	3.70	3.82	3.51	0.23	0.99
PA1940_at	3.44	3.37	3.48	3.65	-0.16	0.99
PA2063_at	2.53	2.59	2.72	2.70	-0.15	0.99
PA2132_at	2.93	2.75	3.03	2.98	-0.16	0.99
PA2224_at	2.81	2.85	2.68	2.68	0.15	0.99
PA2299_at	3.13	3.23	3.20	3.55	-0.20	0.99
PA2507_catA_at	2.61	2.58	2.81	2.69	-0.16	0.99
PA2529_at	3.18	3.16	3.55	3.19	-0.20	0.99
PA2665_at	3.75	3.85	3.90	4.02	-0.16	0.99
PA2666_at	2.37	2.62	2.37	2.27	0.18	0.99
PA2677_at	3.23	3.02	2.92	2.99	0.17	0.99
PA2723_at	3.37	3.32	3.33	3.80	-0.22	0.99
PA2838_at	3.04	2.96	3.11	3.20	-0.16	0.99
PA3110_at	6.17	6.56	6.26	6.03	0.22	0.99
PA3297_at	3.48	3.38	3.27	3.28	0.15	0.99
PA3329_at	4.20	4.49	4.48	4.57	-0.18	0.99
PA3716_at	3.04	3.10	3.16	3.29	-0.16	0.99
PA3736_hom_at	5.57	5.88	5.78	6.09	-0.22	0.99
PA3940_at	5.28	5.49	5.18	5.25	0.17	0.99
PA4175_at	3.91	3.81	4.01	4.02	-0.15	0.99
PA4237_rplQ_at	5.75	6.30	6.24	6.31	-0.25	0.99
PA4394_at	3.48	3.26	3.22	3.19	0.17	0.99
PA4726_at	4.71	5.62	4.70	4.89	0.37	0.99
PA5307_at	3.71	3.68	3.58	3.51	0.15	0.99
PA5388_at	3.35	2.98	2.97	2.97	0.20	0.99
PA5429_aspA_at	5.93	6.57	5.94	6.02	0.27	0.99
Pae_orfK_at	2.45	2.29	2.49	2.59	-0.17	0.99
PA0540_at	2.66	2.78	2.44	2.65	0.17	0.99
PA0764_mucB_at	4.70	5.75	4.75	4.89	0.40	0.99
PA1586_sucB_at	6.67	6.83	6.89	6.93	-0.16	0.99
PA1805_ppiD_at	3.89	4.32	4.35	4.28	-0.21	0.99
PA0424_mexR_at	5.72	5.89	5.43	5.78	0.20	0.99
PA0334_at	4.91	4.57	5.14	4.80	-0.22	0.99
ig_65479_66303_at	2.19	2.30	2.44	2.37	-0.15	0.99
PA0383_at	3.14	2.98	2.94	2.86	0.16	0.99
PA0450_at	4.02	3.72	3.74	3.64	0.18	0.99
PA0546_metK_at	4.18	4.51	4.45	4.63	-0.20	0.99
PA0606_at	2.87	2.88	2.72	2.73	0.15	0.99

PA0659_at	6.12	6.19	6.28	6.32	-0.15	0.99
PA0823_at	2.51	2.77	3.07	2.66	-0.22	0.99
PA0933_ygcA_at	3.59	3.84	3.68	3.35	0.21	0.99
PA1091_at	4.30	3.99	3.94	3.98	0.18	0.99
PA2487_i_at	3.82	3.57	3.46	3.58	0.18	0.99
PA2706_at	5.30	4.98	5.34	5.31	-0.19	0.99
PA3109_at	4.14	4.42	4.11	4.09	0.18	0.99
PA3169_at	4.97	5.07	4.76	4.95	0.17	0.99
PA3384_phnC_at	2.44	2.48	2.66	2.57	-0.15	0.99
PA3613_at	7.48	7.48	7.69	7.57	-0.15	0.99
PA4815_at	2.89	2.89	3.20	2.93	-0.17	0.99
PA4818_at	2.80	2.61	3.15	2.71	-0.22	0.99
PA5210_at	3.93	3.95	4.11	4.07	-0.15	0.99
PA5465_at	4.83	5.75	4.88	4.98	0.36	0.99
PA0615_at	3.04	3.45	3.00	3.08	0.21	0.99
PA0870_phhC_at	5.72	5.42	5.86	5.67	-0.19	0.99
PA1543_apt_at	3.03	2.79	2.87	2.54	0.21	0.99
PA2403_at	3.33	3.10	3.30	3.48	-0.18	0.99
PA2798_at	4.24	4.77	4.20	4.34	0.24	0.99
PA2846_at	2.55	2.60	2.78	2.68	-0.15	0.99
PA2880_at	2.68	2.60	2.92	2.69	-0.17	0.99
PA3129_at	4.97	5.24	5.17	4.42	0.32	0.99
PA3158_wbpB_at	5.04	5.36	5.08	4.94	0.19	0.99
PA3294_s_at	5.18	5.16	5.43	5.23	-0.16	0.99
PA3441_at	2.50	2.46	2.74	2.55	-0.16	0.99
PA3828_at	4.24	4.91	4.12	4.43	0.30	0.99
PA4199_at	5.00	4.99	5.10	5.19	-0.15	0.99
PA4681_at	2.99	2.77	2.82	2.55	0.19	0.99
PA5043_pilN_at	5.43	6.03	6.00	5.98	-0.26	0.99
PA4424_at	4.83	5.17	4.62	4.95	0.22	0.99
PA2119_at	5.29	5.10	5.31	5.41	-0.16	0.99
PA4257_rpsC_at	6.16	7.55	7.44	7.29	-0.51	0.99
PA5547_at	3.56	3.45	3.84	3.53	-0.19	0.99
PA5239_rho_at	4.09	4.76	4.09	4.21	0.28	0.99
PA0066_at	3.68	4.05	3.73	3.61	0.20	0.99
PA3183_zwf_at	4.53	4.97	4.57	4.51	0.21	0.99
PA3466_at	4.32	3.98	4.02	3.90	0.19	0.99
PA0429_at	3.21	3.14	3.24	3.42	-0.16	1.00
PA1852_at	6.07	6.73	6.00	6.23	0.28	1.00
PA2148_at	4.01	3.71	3.72	3.63	0.18	1.00
PA2703_at	3.01	2.87	2.80	2.78	0.15	1.00
PA4285_recC_at	2.82	2.88	3.01	2.98	-0.15	1.00
ig_53521_56546_at	3.06	2.86	2.81	2.79	0.16	1.00
PA0404_i_at	4.96	5.81	5.08	5.04	0.32	1.00

PA2609_at	3.50	3.61	3.59	3.89	-0.18	1.00
PA2800_at	5.16	5.19	5.14	5.67	-0.23	1.00
PA3054_at	4.16	4.49	4.75	4.35	-0.23	1.00
PA3227_ppiA_at	5.24	5.33	5.45	5.41	-0.15	1.00
PA3845_at	4.48	4.69	4.31	4.51	0.18	1.00
Pae_tRNA_Cys_i_at	3.57	3.71	3.26	3.62	0.20	1.00
ig_2117030_2116265_at	3.57	3.48	3.67	3.67	-0.15	1.00
ig_3677719_3677080_at	3.85	3.62	3.66	3.45	0.18	1.00
ig_4321433_4320819_at	4.20	3.80	4.20	4.20	-0.20	1.00
PA0089_at	3.96	3.94	3.82	3.79	0.14	1.00
PA0132_at	4.62	5.35	4.65	4.75	0.28	1.00
PA0174_at	3.37	3.15	3.11	3.10	0.16	1.00
PA0224_at	2.69	2.56	2.96	2.65	-0.18	1.00
PA0285_at	2.74	2.82	2.63	2.64	0.14	1.00
PA0312_at	4.42	4.90	4.88	4.86	-0.21	1.00
PA0313_at	3.83	3.89	3.97	4.04	-0.14	1.00
PA0394_at	4.43	5.08	4.32	4.64	0.28	1.00
PA0405_at	4.40	4.73	4.66	4.85	-0.19	1.00
PA0733_at	5.19	4.70	4.72	4.74	0.22	1.00
PA0774_at	3.00	2.93	2.90	2.72	0.15	1.00
PA0782_putA_at	6.66	7.24	7.08	7.33	-0.25	1.00
PA0785_at	3.91	3.91	4.20	3.95	-0.17	1.00
PA0786_at	2.56	2.45	2.57	2.77	-0.16	1.00
PA0952_at	4.75	5.09	4.76	4.70	0.19	1.00
PA1159_at	4.31	4.77	4.87	4.64	-0.22	1.00
PA1228_at	3.73	4.18	3.71	3.79	0.20	1.00
PA1248_aprF_at	3.53	2.83	3.02	2.76	0.29	1.00
PA1287_at	4.34	3.73	4.20	4.39	-0.26	1.00
PA1288_at	5.76	6.09	6.04	6.19	-0.19	1.00
PA1305_at	5.44	5.80	5.24	5.56	0.22	1.00
PA1326_ilvA2_at	3.33	3.28	3.11	3.21	0.14	1.00
PA1336_at	4.04	3.80	4.14	4.02	-0.16	1.00
PA1345_at	2.75	2.80	2.89	2.95	-0.14	1.00
PA1442_at	5.86	6.55	6.04	5.82	0.28	1.00
PA1460_at	4.82	4.84	4.91	5.06	-0.15	1.00
PA1462_at	4.22	4.93	4.18	4.39	0.29	1.00
PA1472_at	2.60	2.74	2.58	2.45	0.16	1.00
PA1481_ccmG_at	4.29	4.04	4.05	3.93	0.17	1.00
PA1483_cycH_at	3.76	4.12	4.05	4.23	-0.20	1.00
PA1503_at	2.92	2.93	2.88	2.66	0.16	1.00
PA1575_at	3.62	3.41	3.42	3.29	0.16	1.00

PA1606_at	4.16	3.92	3.93	3.82	0.16	1.00
PA1714_at	4.41	6.11	5.75	5.95	-0.59	1.00
PA1736_at	3.10	2.75	3.11	3.10	-0.19	1.00
PA1989_pqqE_at	2.62	3.01	3.01	3.01	-0.20	1.00
PA2013_at	3.94	3.93	3.79	3.80	0.14	1.00
PA2184_at	3.79	3.34	3.50	3.17	0.23	1.00
PA2198_at	3.45	3.91	3.79	4.03	-0.23	1.00
PA2208_at	3.17	2.61	2.56	2.75	0.24	1.00
PA2225_at	2.85	2.76	2.89	3.01	-0.15	1.00
PA2298_at	4.58	4.59	4.47	4.43	0.14	1.00
PA2391_at	2.93	2.75	2.92	3.09	-0.16	1.00
PA2505_at	2.60	2.56	2.52	2.35	0.15	1.00
PA2571_at	5.21	4.59	5.25	5.06	-0.25	1.00
PA2588_at	4.51	4.17	4.56	4.49	-0.18	1.00
PA2598_at	2.68	2.44	2.73	2.70	-0.16	1.00
PA2739_pheT_at	4.51	4.80	4.81	4.86	-0.17	1.00
PA2768_at	3.66	3.01	3.52	3.67	-0.26	1.00
PA2835_at	2.82	2.33	2.81	2.78	-0.22	1.00
PA2926_hisP_at	4.14	3.52	3.62	3.53	0.26	1.00
PA2929_at	2.90	3.01	2.78	2.84	0.14	1.00
PA2945_at	3.66	3.75	3.61	3.50	0.15	1.00
PA3004_at	2.88	2.91	3.03	3.03	-0.14	1.00
PA3005_at	3.55	3.59	3.41	3.44	0.14	1.00
PA3044_at	4.03	3.45	3.48	3.52	0.24	1.00
PA3106_at	3.60	3.63	3.43	3.50	0.15	1.00
PA3147_wbpJ_at	3.57	3.88	3.58	3.51	0.18	1.00
PA3279_oprP_at	2.68	2.34	2.67	2.71	-0.18	1.00
PA3321_at	3.29	3.20	3.13	3.07	0.14	1.00
PA3341_at	4.86	5.05	5.24	5.02	-0.17	1.00
PA3373_at	3.75	3.45	3.96	3.65	-0.20	1.00
PA3420_at	3.01	2.76	3.15	2.97	-0.17	1.00
PA3460_at	2.91	3.24	3.26	3.24	-0.18	1.00
PA3501_at	2.57	2.58	2.72	2.72	-0.14	1.00
PA3547_algL_at	2.69	2.37	2.46	2.20	0.20	1.00
PA3567_at	3.42	3.82	3.78	3.86	-0.20	1.00
PA3595_at	2.95	2.70	3.02	2.95	-0.16	1.00
PA3599_at	2.88	2.92	2.83	2.65	0.16	1.00
PA3620_mutS_at	5.31	4.83	4.85	4.86	0.21	1.00
PA3717_at	2.95	2.64	2.89	3.08	-0.19	1.00
PA3772_at	3.53	3.31	3.60	3.56	-0.16	1.00
PA3914_moeA1_at	3.55	3.57	3.34	3.48	0.15	1.00
PA3952_at	4.71	4.79	4.60	4.62	0.14	1.00
PA3995_at	2.79	2.83	2.96	2.96	-0.14	1.00
PA4013_at	4.55	4.21	4.53	4.60	-0.18	1.00

PA4185_at	3.31	3.29	3.72	3.28	-0.20	1.00
PA4234_uvrA_at	4.52	4.88	4.63	4.36	0.21	1.00
PA4254_rpsQ_at	7.32	8.30	8.00	8.38	-0.38	1.00
PA4465_at	4.84	5.40	4.89	4.88	0.24	1.00
PA4475_at	5.74	6.13	5.75	5.74	0.19	1.00
PA4514_at	2.59	2.54	2.92	2.58	-0.18	1.00
PA4537_at	6.09	6.53	6.07	6.13	0.21	1.00
PA4580_at	4.28	4.12	4.18	3.86	0.19	1.00
PA4599_mexC_at	3.07	2.64	2.69	2.61	0.20	1.00
PA4618_at	2.78	2.84	2.75	2.55	0.16	1.00
PA4621_at	4.37	4.65	4.41	4.25	0.18	1.00
PA4629_at	3.59	3.85	3.67	3.37	0.20	1.00
PA4733_acsB_at	3.88	3.58	3.98	3.84	-0.18	1.00
PA4743_rbfA_at	2.91	4.08	2.91	3.22	0.43	1.00
PA4774_at	3.17	2.75	2.75	2.77	0.20	1.00
PA4777_at	3.05	2.75	2.72	2.72	0.18	1.00
PA4841_at	3.69	3.74	3.84	3.87	-0.14	1.00
PA4895_at	2.56	2.54	2.47	2.34	0.15	1.00
PA4909_at	2.98	2.62	2.67	2.55	0.19	1.00
PA4925_at	3.13	3.16	3.48	3.16	-0.18	1.00
PA4942_hflK_at	4.51	5.42	4.48	4.74	0.35	1.00
PA5055_at	5.13	5.41	4.92	5.23	0.20	1.00
PA5080_at	4.32	4.74	4.57	4.95	-0.23	1.00
PA5148_at	5.86	6.24	6.18	6.31	-0.19	1.00
PA5180_at	3.01	3.17	3.21	3.28	-0.16	1.00
PA5189_at	3.15	3.03	2.97	2.93	0.15	1.00
PA5365_phoU_at	3.08	3.13	2.95	2.97	0.14	1.00
PA5367_pstA_at	4.24	3.95	3.99	3.85	0.18	1.00
PA5406_at	5.42	5.48	5.30	5.32	0.15	1.00
PA5470_at	5.31	6.08	5.46	5.34	0.30	1.00
PA5483_algB_at	3.68	3.61	3.48	3.53	0.14	1.00
PA5536_at	2.55	2.49	2.47	2.24	0.16	1.00
Pae_AF241171cds24_at	2.56	2.50	2.73	2.63	-0.15	1.00
Pae_AF241171cds44_r_at	4.47	3.43	4.40	4.24	-0.37	1.00
Pae_orfH_at	2.62	2.43	2.29	2.44	0.16	1.00
PA0481_at	4.56	3.98	4.14	3.91	0.25	1.00
PA1021_at	2.70	2.50	2.64	2.92	-0.18	1.00
PA1031_at	4.30	4.52	4.14	4.35	0.17	1.00
PA1217_at	2.56	2.48	2.63	2.69	-0.14	1.00
PA3090_at	3.71	3.10	3.51	3.84	-0.27	1.00
PA3458_at	5.58	5.88	5.88	5.92	-0.17	1.00
PA4256_rplP_at	8.47	9.06	9.03	8.98	-0.24	1.00
PA4851_at	4.80	5.29	5.23	5.28	-0.21	1.00

ig_5513731_5513111_at	3.43	3.14	3.46	3.45	-0.17	1.00
PA1707_pcrH_at	5.71	6.62	6.39	6.62	-0.34	1.00
PA5256_dsbH_at	3.49	3.50	3.13	3.49	0.18	1.00
PA1153_at	3.79	3.53	3.41	3.58	0.17	1.00
PA2282_at	3.44	3.37	3.53	3.56	-0.14	1.00
PA2653_at	5.07	4.79	4.73	4.80	0.17	1.00
AFFX-Athal_GAPDH_at	2.23	1.89	2.12	2.08	-0.04	1.00
AFFX-Athal_ubq_at	2.13	1.98	2.06	2.17	-0.06	1.00
AFFX-Bsubtilis_pheB_at	3.96	4.15	4.28	3.99	-0.08	1.00
AFFX-Bsubtilis_trpD_at	2.07	1.94	2.07	2.10	-0.08	1.00
AFFX-YEL002C_WPB1_at	2.04	2.03	2.11	2.04	-0.04	1.00
AFFX-YEL024W_RIP1_at	2.24	2.13	2.26	2.16	-0.03	1.00
AFFX-YER022W_SRB4_at	2.18	1.98	2.10	2.15	-0.05	1.00
ig_1046911_1047549_at	3.51	2.81	3.20	2.89	0.11	1.00
ig_1047549_1046911_at	2.97	2.84	2.80	2.73	0.14	1.00
ig_1087095_1087843_at	4.52	4.17	4.33	4.23	0.06	1.00
ig_1204781_1205771_at	4.59	4.29	4.31	4.31	0.13	1.00
ig_1205771_1204781_at	3.72	4.08	3.80	3.75	0.12	1.00
ig_1246788_1247932_at	2.61	2.36	2.60	2.32	0.02	1.00
ig_1247932_1246788_at	2.13	2.02	2.12	2.10	-0.04	1.00
ig_1255042_1254309_at	4.87	4.65	4.73	4.70	0.05	1.00
ig_1324109_1324793_at	2.70	2.71	2.75	2.72	-0.03	1.00
ig_1348401_1349416_at	2.77	2.70	2.90	2.75	-0.09	1.00
ig_1428080_1427453_at	3.19	3.00	3.16	2.95	0.04	1.00
ig_1489095_1489815_at	2.79	2.62	2.57	2.62	0.11	1.00
ig_1489815_1489095_at	3.46	3.43	3.45	3.57	-0.07	1.00

ig_1553675_1554415_at	2.95	2.85	2.82	2.86	0.06	1.00
ig_1554415_1553675_at	3.46	3.40	3.47	3.32	0.04	1.00
ig_1637696_1638379_at	3.29	3.01	2.99	3.01	0.15	1.00
ig_1638379_1637696_at	2.53	2.60	2.62	2.60	-0.05	1.00
ig_1802453_1803626_at	2.57	2.49	2.69	2.41	-0.02	1.00
ig_1803626_1802453_at	2.95	2.99	3.11	3.07	-0.12	1.00
ig_188448_189120_at	2.94	3.06	3.26	3.04	-0.15	1.00
ig_189120_188448_at	3.66	3.39	3.60	3.57	-0.06	1.00
ig_1997509_1996806_at	2.68	2.61	2.59	2.77	-0.04	1.00
ig_2069490_2068728_at	3.02	2.53	2.98	2.73	-0.08	1.00
ig_2116265_2117030_at	3.24	2.98	3.19	2.98	0.03	1.00
ig_2165213_2164548_at	3.13	2.94	3.11	2.91	0.03	1.00
ig_2226144_2226879_at	2.83	2.48	2.76	2.65	-0.05	1.00
ig_2226879_2226144_at	2.75	2.63	2.83	2.69	-0.07	1.00
ig_2239267_2240302_at	3.13	2.72	3.01	3.01	-0.09	1.00
ig_224101_223454_at	2.49	2.36	2.48	2.59	-0.11	1.00
ig_2243603_2244492_at	2.99	2.81	3.08	3.01	-0.14	1.00
ig_2250595_2251275_at	2.64	2.43	2.45	2.46	0.07	1.00
ig_2251275_2250595_at	3.08	2.56	2.87	2.93	-0.08	1.00
ig_2281578_2282480_at	2.77	2.71	2.77	2.89	-0.09	1.00
ig_2282480_2281578_at	3.31	3.01	3.30	3.33	-0.16	1.00
ig_2450765_2451707_at	2.20	2.16	2.43	2.22	-0.14	1.00
ig_2451707_2450765_at	2.21	2.09	2.19	2.26	-0.07	1.00
ig_2557964_2558918_s_at	4.59	4.86	4.68	4.67	0.05	1.00
ig_2568284_2568929_at	2.36	2.01	2.20	2.25	-0.04	1.00

ig_2568929_2568284_at	3.82	3.01	3.76	3.53	-0.23	1.00
ig_2721530_2722174_at	2.38	2.21	2.28	2.21	0.05	1.00
ig_2722174_2721530_at	2.75	2.50	2.79	2.59	-0.07	1.00
ig_2737193_2737881_at	2.58	2.43	2.46	2.40	0.07	1.00
ig_2737881_2737193_at	3.30	3.06	3.29	3.13	-0.03	1.00
ig_2893827_2894451_at	4.23	5.15	4.44	4.26	0.33	1.00
ig_2894451_2893827_at	3.64	3.43	3.41	3.40	0.13	1.00
ig_2901558_2902217_at	3.34	3.32	3.34	3.28	0.02	1.00
ig_2906165_2905549_at	2.81	2.92	2.91	3.03	-0.11	1.00
ig_2918211_2918966_at	3.59	3.53	3.48	3.70	-0.03	1.00
ig_2922568_2923366_at	2.86	2.47	2.77	2.65	-0.05	1.00
ig_2927920_2928538_at	2.70	2.51	2.78	2.66	-0.11	1.00
ig_2928538_2927920_at	3.68	2.77	3.38	3.17	-0.05	1.00
ig_299081_297561_at	2.77	2.43	2.58	2.67	-0.03	1.00
ig_3051348_3051956_at	3.41	3.31	3.34	3.49	-0.05	1.00
ig_3051956_3051348_at	2.82	2.53	2.89	2.66	-0.10	1.00
ig_3088659_3087490_at	2.84	2.80	2.91	2.67	0.03	1.00
ig_3112150_3112877_at	5.19	4.81	4.91	4.96	0.06	1.00
ig_3115632_3116653_at	2.80	3.02	2.99	2.99	-0.08	1.00
ig_3116653_3115632_at	5.45	4.96	5.16	5.43	-0.09	1.00
ig_3122585_3123598_at	3.13	2.72	3.08	2.86	-0.04	1.00
ig_3123598_3122585_at	3.03	2.73	2.70	2.93	0.06	1.00
ig_3129070_3129728_at	2.73	2.63	2.76	2.68	-0.04	1.00
ig_3129728_3129070_at	3.88	4.26	3.89	4.01	0.12	1.00
ig_3206252_3206914_at	2.89	2.96	2.85	3.05	-0.02	1.00

ig_3206914_3206252_at	3.17	2.67	2.88	2.84	0.06	1.00
ig_3265210_3265847_at	2.50	2.10	2.29	2.47	-0.08	1.00
ig_3265847_3265210_at	2.83	2.67	2.79	2.88	-0.09	1.00
ig_326671_327284_at	4.06	3.97	4.07	4.00	-0.02	1.00
ig_3475169_3475955_at	2.45	2.22	2.49	2.37	-0.10	1.00
ig_3475955_3475169_at	2.71	2.93	2.64	2.72	0.14	1.00
ig_3514564_3515415_at	2.59	2.12	2.26	2.25	0.10	1.00
ig_3515415_3514564_at	2.63	2.44	2.45	2.44	0.09	1.00
ig_3545073_3545880_at	2.43	2.22	2.14	2.20	0.15	1.00
ig_3546926_3547688_at	2.40	2.58	2.32	2.52	0.07	1.00
ig_3547688_3546926_at	5.75	6.03	5.86	5.77	0.08	1.00
ig_3648915_3649704_at	3.47	3.20	3.46	3.28	-0.03	1.00
ig_3649704_3648915_at	4.16	4.60	4.15	4.29	0.15	1.00
ig_3677080_3677719_at	3.23	2.83	3.02	2.72	0.16	1.00
ig_3705160_3705889_at	4.14	3.62	4.23	3.97	-0.22	1.00
ig_3705889_3705160_at	3.43	3.66	3.44	3.53	0.06	1.00
ig_3874653_3873835_r_at	7.68	6.87	7.47	7.50	-0.21	1.00
ig_3961922_3962824_at	2.24	2.04	2.35	2.23	-0.15	1.00
ig_3962824_3961922_at	2.47	2.13	2.16	2.18	0.13	1.00
ig_4240578_4241341_at	3.11	3.15	3.30	3.10	-0.07	1.00
ig_4320819_4321433_at	3.43	3.50	3.20	3.52	0.10	1.00
ig_4327696_4326394_at	2.90	2.84	2.84	2.62	0.14	1.00
ig_4448217_4448892_at	3.49	3.17	3.41	3.31	-0.03	1.00
ig_4448892_4448217_at	4.92	4.30	4.59	4.32	0.16	1.00
ig_4509996_4510970_at	2.43	2.31	2.34	2.17	0.12	1.00

ig_4510970_4509996_at	2.46	2.50	2.64	2.48	-0.08	1.00
ig_4559649_4560294_at	3.62	3.47	3.47	3.76	-0.07	1.00
ig_4560294_4559649_at	2.05	2.12	2.10	2.18	-0.06	1.00
ig_4584530_4585148_at	2.54	2.52	2.47	2.65	-0.03	1.00
ig_4585148_4584530_at	3.31	2.99	3.24	3.17	-0.05	1.00
ig_4629184_4629943_at	2.41	2.20	2.23	2.23	0.07	1.00
ig_4629943_4629184_at	3.18	2.85	3.02	2.90	0.06	1.00
ig_4705304_4705955_at	3.00	2.81	2.92	2.84	0.02	1.00
ig_4705955_4705304_at	4.22	3.49	4.14	4.05	-0.24	1.00
ig_4713098_4713795_at	2.42	2.16	2.37	2.12	0.05	1.00
ig_4713795_4713098_at	2.69	2.27	2.36	2.53	0.03	1.00
ig_4956733_4956028_at	11.06	11.25	11.15	11.28	-0.06	1.00
ig_5086695_5087407_at	3.02	2.79	2.73	2.85	0.12	1.00
ig_518083_517462_at	3.86	3.70	3.99	3.73	-0.08	1.00
ig_5207621_5208463_at	3.27	3.53	3.41	3.62	-0.11	1.00
ig_5208463_5207621_at	3.18	2.88	3.18	3.03	-0.07	1.00
ig_5242558_5243177_at	2.42	2.27	2.32	2.19	0.09	1.00
ig_5243177_5242558_at	5.57	5.67	5.49	5.50	0.12	1.00
ig_5308424_5309325_at	6.14	6.11	6.26	6.25	-0.13	1.00
ig_545644_546334_at	3.40	3.48	3.70	3.41	-0.12	1.00
ig_5457716_5458499_i_at	2.40	2.24	2.35	2.42	-0.07	1.00
ig_546334_545644_at	2.67	2.66	2.61	2.53	0.09	1.00
ig_5513111_5513731_at	2.22	1.98	2.33	2.15	-0.14	1.00
ig_5542072_5541409_at	4.05	4.38	4.19	4.18	0.03	1.00
ig_5563286_5563964_at	5.37	5.31	5.20	5.63	-0.08	1.00

ig_5563964_5563286_at	3.35	3.12	3.55	3.25	-0.16	1.00
ig_56546_53521_at	2.20	2.11	2.22	2.14	-0.02	1.00
ig_5774806_5775619_at	2.48	2.31	2.55	2.46	-0.11	1.00
ig_5820113_5820909_at	2.55	2.39	2.45	2.31	0.09	1.00
ig_5820909_5820113_at	4.22	3.83	4.03	3.88	0.07	1.00
ig_5991130_5992382_at	4.60	4.89	4.67	4.88	-0.03	1.00
ig_5992382_5991130_at	4.35	3.85	4.18	4.07	-0.03	1.00
ig_6090705_6092045_at	2.39	2.12	2.48	2.30	-0.14	1.00
ig_6092045_6090705_at	2.60	2.59	2.66	2.69	-0.08	1.00
ig_6125079_6125795_at	3.30	3.07	3.17	3.58	-0.19	1.00
ig_6125795_6125079_at	3.43	3.17	3.46	3.33	-0.10	1.00
ig_629884_630527_at	4.09	3.72	3.95	4.09	-0.11	1.00
ig_630527_629884_at	3.05	2.71	2.69	2.85	0.11	1.00
ig_65339_64729_at	3.16	2.89	3.02	2.93	0.05	1.00
ig_66303_65479_at	2.71	2.75	2.71	2.70	0.03	1.00
ig_69272_68616_at	3.64	3.43	3.52	3.39	0.08	1.00
ig_721556_727608_s_at	7.73	8.17	7.88	7.87	0.08	1.00
ig_727608_721556_s_at	2.36	2.29	2.30	2.44	-0.05	1.00
ig_785174_785969_at	2.95	2.99	2.88	2.97	0.05	1.00
ig_789144_788253_at	2.46	2.73	2.51	2.53	0.08	1.00
ig_863300_864095_at	3.49	3.71	3.60	3.71	-0.06	1.00
ig_864095_863300_at	3.49	3.28	3.42	3.54	-0.09	1.00
ig_884172_884799_at	4.83	4.25	4.36	4.51	0.10	1.00
ig_901046_901934_at	2.42	2.42	2.28	2.40	0.09	1.00
ig_991198_991830_at	5.41	5.59	5.36	5.47	0.08	1.00

ig_991830_991198_at	2.33	2.16	2.29	2.33	-0.06	1.00
PA0001_dnaA_at	4.23	4.78	4.41	4.35	0.12	1.00
PA0002_dnaN_at	5.05	5.43	5.41	5.24	-0.09	1.00
PA0003_recF_at	2.67	2.90	2.70	2.66	0.11	1.00
PA0004_gyrB_at	4.84	4.73	4.92	4.84	-0.09	1.00
PA0006_at	3.77	3.16	3.26	3.45	0.11	1.00
PA0007_at	4.64	4.13	4.48	4.60	-0.15	1.00
PA0008_glyS_at	4.14	4.27	4.20	4.12	0.04	1.00
PA0009_glyQ_at	3.76	4.05	4.04	4.11	-0.17	1.00
PA0010_tag_at	3.15	2.73	2.91	2.88	0.04	1.00
PA0011_at	4.50	4.19	4.38	4.54	-0.12	1.00
PA0012_at	4.63	4.20	4.52	4.41	-0.06	1.00
PA0013_at	2.93	2.94	2.96	3.04	-0.07	1.00
PA0014_at	2.46	2.36	2.43	2.24	0.08	1.00
PA0015_at	2.86	2.81	2.76	2.82	0.05	1.00
PA0016_trkA_at	2.75	2.78	2.86	2.89	-0.11	1.00
PA0017_at	4.08	4.07	4.02	4.18	-0.03	1.00
PA0018_fmt_at	3.13	3.55	3.10	3.36	0.11	1.00
PA0019_def_at	3.81	4.11	3.82	3.96	0.07	1.00
PA0020_at	6.02	6.19	6.15	6.30	-0.11	1.00
PA0022_at	2.67	2.69	2.93	2.68	-0.13	1.00
PA0023_qor_at	4.23	3.93	4.02	4.23	-0.04	1.00
PA0028_at	3.24	2.96	3.07	2.85	0.14	1.00
PA0029_at	5.04	4.61	4.86	4.89	-0.05	1.00
PA0030_at	3.47	2.96	2.98	3.03	0.21	1.00
PA0031_betC_at	3.03	2.90	3.04	3.01	-0.06	1.00
PA0032_at	4.26	3.75	3.93	3.97	0.06	1.00
PA0033_at	3.42	3.02	3.26	3.09	0.04	1.00
PA0035_trpA_at	4.82	4.85	4.77	4.65	0.12	1.00
PA0042_at	2.49	2.67	2.37	2.52	0.14	1.00
PA0043_at	2.86	2.82	2.80	2.76	0.06	1.00
PA0045_at	5.38	6.08	5.93	5.85	-0.16	1.00
PA0046_at	4.61	5.13	4.81	5.03	-0.05	1.00
PA0047_at	3.12	3.27	3.30	3.31	-0.11	1.00
PA0048_at	3.04	2.86	2.94	3.09	-0.06	1.00
PA0056_at	2.44	2.36	2.28	2.40	0.06	1.00
PA0057_at	3.01	2.82	2.90	2.67	0.13	1.00
PA0058_at	3.42	3.43	3.66	3.33	-0.07	1.00
PA0059_osmC_at	4.12	3.79	3.83	3.80	0.14	1.00
PA0060_at	4.17	4.81	4.22	4.32	0.22	1.00
PA0062_at	2.55	2.89	2.80	2.59	0.03	1.00
PA0063_at	2.81	3.26	3.08	3.38	-0.20	1.00
PA0064_at	3.42	2.99	3.10	3.00	0.15	1.00
PA0065_at	3.38	3.22	3.36	3.34	-0.05	1.00

PA0068_at	5.06	5.18	5.15	5.26	-0.08	1.00
PA0073_at	5.66	5.55	5.67	5.68	-0.07	1.00
PA0075_at	4.23	3.96	4.02	4.25	-0.04	1.00
PA0076_at	3.75	4.00	3.85	3.98	-0.04	1.00
PA0078_at	4.62	4.69	4.71	4.64	-0.02	1.00
PA0080_at	4.55	4.26	4.21	4.39	0.11	1.00
PA0081_at	4.79	5.37	5.06	5.30	-0.10	1.00
PA0082_at	4.97	4.83	4.86	4.76	0.09	1.00
PA0083_at	4.93	5.03	5.21	4.91	-0.08	1.00
PA0084_at	6.33	6.54	6.41	6.59	-0.07	1.00
PA0085_at	5.97	6.21	6.16	6.10	-0.04	1.00
PA0086_at	4.86	5.10	5.00	5.24	-0.14	1.00
PA0088_at	2.89	2.66	3.00	2.78	-0.12	1.00
PA0091_at	2.94	3.12	2.84	3.10	0.06	1.00
PA0093_at	4.52	4.42	4.14	4.50	0.15	1.00
PA0094_at	4.08	4.37	4.21	4.31	-0.04	1.00
PA0096_at	3.85	3.48	3.61	3.53	0.09	1.00
PA0097_at	3.95	3.70	3.53	3.79	0.17	1.00
PA0098_at	3.24	3.06	3.05	3.40	-0.08	1.00
PA0099_at	3.31	3.20	3.19	3.25	0.03	1.00
PA0101_at	3.52	3.65	3.80	3.54	-0.08	1.00
PA0103_at	3.86	3.86	3.93	3.74	0.02	1.00
PA0105_coxB_at	2.72	2.83	2.81	2.87	-0.06	1.00
PA0106_coxA_at	2.56	2.57	2.64	2.63	-0.07	1.00
PA0108_coIII_at	2.81	2.66	2.76	2.78	-0.03	1.00
PA0109_at	3.86	3.94	3.76	4.09	-0.02	1.00
PA0111_at	2.55	2.57	2.67	2.52	-0.03	1.00
PA0116_at	4.12	3.83	4.06	3.60	0.14	1.00
PA0117_at	3.37	3.24	3.22	3.21	0.09	1.00
PA0119_at	2.70	2.66	2.38	2.70	0.14	1.00
PA0120_at	3.14	2.94	3.12	2.60	0.18	1.00
PA0124_at	3.49	3.98	3.51	3.56	0.20	1.00
PA0126_at	4.12	4.07	3.99	4.02	0.09	1.00
PA0127_at	4.53	3.82	4.46	4.24	-0.18	1.00
PA0133_at	3.58	3.70	3.87	3.56	-0.08	1.00
PA0137_at	3.75	3.35	3.88	3.53	-0.16	1.00
PA0138_at	4.20	4.06	3.98	4.16	0.06	1.00
PA0139_ahpC_at	6.24	6.51	6.46	6.58	-0.15	1.00
PA0140_ahpF_at	4.16	3.69	3.90	3.68	0.14	1.00
PA0141_at	5.45	5.91	5.68	6.09	-0.20	1.00
PA0142_at	3.32	3.01	3.16	3.02	0.08	1.00
PA0143_at	3.46	3.03	3.41	3.36	-0.14	1.00
PA0144_at	3.14	3.11	2.99	3.16	0.05	1.00
PA0145_i_at	3.22	2.60	2.81	2.87	0.07	1.00

PA0146_at	4.59	3.80	4.34	3.91	0.07	1.00
PA0147_at	3.75	3.84	3.82	3.61	0.08	1.00
PA0148_at	4.56	4.29	4.44	4.75	-0.17	1.00
PA0150_at	2.74	2.74	2.79	2.87	-0.08	1.00
PA0151_at	3.31	2.98	3.31	3.09	-0.06	1.00
PA0152_pcaQ_at	2.89	2.79	3.00	2.89	-0.11	1.00
PA0153_pcaH_at	4.48	4.29	4.30	4.29	0.09	1.00
PA0154_pcaG_at	3.86	3.52	3.90	3.69	-0.11	1.00
PA0156_at	5.06	4.86	4.83	4.98	0.06	1.00
PA0157_at	3.36	3.48	3.28	3.37	0.09	1.00
PA0158_at	3.20	2.96	2.83	3.15	0.09	1.00
PA0160_at	2.95	3.61	3.27	3.48	-0.10	1.00
PA0161_at	2.99	3.16	3.00	3.02	0.07	1.00
PA0162_at	4.05	3.87	4.02	3.71	0.10	1.00
PA0163_at	3.73	3.58	3.85	3.55	-0.05	1.00
PA0164_at	3.97	3.47	3.54	3.62	0.14	1.00
PA0165_at	3.77	4.18	4.01	4.26	-0.16	1.00
PA0166_at	2.84	2.69	2.61	2.72	0.10	1.00
PA0169_at	2.66	2.62	2.65	2.94	-0.15	1.00
PA0170_at	2.93	2.72	2.87	2.97	-0.10	1.00
PA0171_at	4.54	4.21	4.65	4.31	-0.10	1.00
PA0173_at	2.93	2.78	2.74	2.78	0.10	1.00
PA0178_at	3.67	3.49	3.57	3.67	-0.04	1.00
PA0180_at	3.53	3.74	3.49	3.54	0.12	1.00
PA0181_at	3.47	3.30	3.55	3.41	-0.09	1.00
PA0182_at	3.92	3.89	4.02	3.58	0.10	1.00
PA0183_atsA_at	3.47	3.15	3.30	3.43	-0.05	1.00
PA0184_at	2.77	2.53	2.69	2.55	0.03	1.00
PA0186_at	2.68	2.44	2.75	2.67	-0.15	1.00
PA0187_at	2.76	2.55	2.82	2.75	-0.13	1.00
PA0189_at	3.13	3.04	3.28	3.13	-0.12	1.00
PA0190_at	3.51	3.21	3.39	3.38	-0.02	1.00
PA0191_at	2.82	2.55	2.54	2.57	0.13	1.00
PA0192_at	3.38	3.06	3.15	3.50	-0.11	1.00
PA0193_at	2.50	2.48	2.53	2.58	-0.07	1.00
PA0194_at	4.46	3.73	3.86	4.11	0.11	1.00
PA0195_pntA_at	4.86	5.05	4.86	5.14	-0.05	1.00
PA0196_pntB_at	2.69	2.62	2.83	2.72	-0.12	1.00
PA0197_at	4.15	3.64	3.93	3.67	0.10	1.00
PA0198_exbB1_at	2.62	2.32	2.63	2.43	-0.06	1.00
PA0202_at	2.98	2.71	2.73	2.62	0.17	1.00
PA0204_at	2.34	2.16	2.29	2.46	-0.12	1.00
PA0205_at	4.03	3.48	3.90	3.67	-0.03	1.00
PA0206_at	3.82	3.62	3.82	3.75	-0.07	1.00

PA0207_at	5.58	5.59	5.44	5.54	0.09	1.00
PA0208_mdcA_at	3.21	2.85	2.94	3.21	-0.04	1.00
PA0209_at	3.03	2.90	2.92	2.81	0.10	1.00
PA0210_mdcC_at	3.54	3.46	3.44	3.38	0.09	1.00
PA0211_mdcD_at	3.18	2.93	3.22	3.09	-0.10	1.00
PA0215_at	2.88	2.67	2.90	2.77	-0.06	1.00
PA0217_at	2.89	2.79	2.75	2.88	0.02	1.00
PA0219_at	3.24	3.02	3.18	3.28	-0.10	1.00
PA0220_at	2.68	2.49	2.62	2.69	-0.07	1.00
PA0221_at	3.48	3.51	3.54	3.50	-0.02	1.00
PA0227_at	3.18	3.04	3.23	3.28	-0.14	1.00
PA0228_pcaF_at	2.51	2.47	2.56	2.63	-0.11	1.00
PA0229_pcaT_at	3.24	2.93	3.13	3.11	-0.03	1.00
PA0230_pcaB_at	4.54	4.48	4.46	4.40	0.08	1.00
PA0232_pcaC_at	2.45	2.41	2.63	2.44	-0.11	1.00
PA0233_at	3.58	3.21	3.46	3.52	-0.10	1.00
PA0235_pcaK_at	4.97	4.28	4.49	4.27	0.25	1.00
PA0237_at	3.59	3.28	3.68	3.43	-0.12	1.00
PA0239_at	3.18	3.52	3.35	3.15	0.10	1.00
PA0240_at	3.70	3.21	3.14	3.63	0.07	1.00
PA0242_at	3.71	3.31	3.37	3.45	0.10	1.00
PA0243_at	4.18	3.35	3.80	3.46	0.13	1.00
PA0244_at	3.55	3.22	3.40	3.73	-0.18	1.00
PA0245_aroQ2_at	3.72	3.81	3.72	3.66	0.07	1.00
PA0246_at	3.75	3.71	3.88	3.70	-0.06	1.00
PA0248_at	2.94	2.94	2.88	2.83	0.09	1.00
PA0249_at	3.06	3.10	3.10	3.18	-0.06	1.00
PA0250_at	4.04	3.86	3.77	3.96	0.09	1.00
PA0251_at	2.49	2.20	2.47	2.39	-0.09	1.00
PA0253_at	4.12	3.72	3.91	3.81	0.06	1.00
PA0254_at	3.82	3.61	3.72	3.80	-0.04	1.00
PA0255_at	4.20	3.79	4.00	3.91	0.04	1.00
PA0256_at	3.20	2.87	3.03	2.98	0.03	1.00
PA0257_at	2.95	2.72	2.68	2.73	0.13	1.00
PA0258_at	2.64	2.61	2.53	2.61	0.05	1.00
PA0259_at	4.73	4.59	4.61	4.50	0.10	1.00
PA0260_at	3.88	4.21	4.45	4.06	-0.21	1.00
PA0262_at	2.80	2.62	2.70	2.61	0.06	1.00
PA0264_at	2.90	2.81	3.00	2.98	-0.13	1.00
PA0265_gabD_at	4.29	5.25	4.38	4.65	0.25	1.00
PA0266_gabT_at	5.26	5.69	5.41	5.20	0.17	1.00
PA0267_at	3.60	3.44	3.57	3.57	-0.05	1.00
PA0268_at	3.31	3.49	3.35	3.35	0.05	1.00
PA0269_at	3.21	3.26	3.35	3.32	-0.10	1.00

PA0270_at	4.67	4.33	4.76	4.53	-0.15	1.00
PA0272_at	3.01	2.68	2.68	2.75	0.13	1.00
PA0273_at	3.70	3.38	3.75	3.64	-0.16	1.00
PA0275_at	3.32	3.31	3.45	3.32	-0.07	1.00
PA0277_at	4.22	5.16	4.23	4.73	0.21	1.00
PA0278_at	3.14	2.83	3.17	3.11	-0.15	1.00
PA0279_at	4.54	4.37	4.47	4.55	-0.06	1.00
PA0280_cysA_at	3.44	2.67	3.28	3.04	-0.11	1.00
PA0282_cysT_at	5.07	4.49	4.94	4.99	-0.18	1.00
PA0286_at	2.77	3.14	2.92	2.94	0.02	1.00
PA0290_at	3.35	3.23	3.20	3.18	0.11	1.00
PA0292_at	4.16	3.86	4.16	4.07	-0.11	1.00
PA0295_at	4.44	4.76	4.64	4.67	-0.05	1.00
PA0297_at	4.29	4.42	4.45	4.42	-0.08	1.00
PA0298_at	3.32	3.50	3.43	3.35	0.03	1.00
PA0299_at	6.47	6.75	6.79	6.60	-0.09	1.00
PA0300_potF2_at	5.06	5.44	5.31	5.50	-0.16	1.00
PA0301_potF3_at	2.57	2.75	2.74	2.79	-0.11	1.00
PA0302_potG_at	2.78	2.76	2.70	2.68	0.08	1.00
PA0303_potH_at	2.76	2.45	2.64	2.63	-0.03	1.00
PA0305_at	3.79	3.70	3.81	3.84	-0.08	1.00
PA0307_at	3.34	2.77	2.89	3.00	0.11	1.00
PA0309_at	2.85	3.40	2.88	2.99	0.19	1.00
PA0311_at	2.68	2.66	2.57	2.72	0.03	1.00
PA0314_at	5.45	5.19	5.52	5.06	0.03	1.00
PA0315_at	3.04	3.35	3.09	3.02	0.14	1.00
PA0316_serA_at	4.46	4.57	4.45	4.71	-0.06	1.00
PA0318_at	4.67	4.73	4.88	4.47	0.03	1.00
PA0319_at	4.09	3.96	4.23	4.07	-0.12	1.00
PA0320_at	3.17	2.68	2.81	2.69	0.18	1.00
PA0321_at	3.76	3.28	3.74	3.43	-0.06	1.00
PA0323_at	2.32	2.14	2.29	2.31	-0.07	1.00
PA0324_at	2.88	2.66	2.86	2.76	-0.04	1.00
PA0325_at	3.46	3.01	3.54	3.10	-0.08	1.00
PA0327_at	3.10	2.59	3.15	2.80	-0.13	1.00
PA0328_at	3.65	3.58	3.57	3.54	0.06	1.00
PA0330_rpiA_at	4.81	5.35	4.80	5.01	0.17	1.00
PA0332_at	4.12	4.30	4.23	4.03	0.08	1.00
PA0333_at	3.96	4.05	3.86	4.02	0.07	1.00
PA0335_at	4.30	4.22	4.23	4.48	-0.09	1.00
PA0338_at	3.14	2.84	3.01	2.75	0.11	1.00
PA0339_at	3.65	3.84	3.57	3.73	0.10	1.00
PA0340_at	4.08	4.05	3.98	4.03	0.06	1.00
PA0343_at	3.20	2.94	2.82	3.24	0.04	1.00

PA0344_at	3.82	3.60	3.51	3.63	0.14	1.00
PA0346_at	3.16	3.58	3.34	3.01	0.19	1.00
PA0348_at	3.54	3.39	3.48	3.34	0.05	1.00
PA0349_at	3.16	3.04	3.11	3.03	0.03	1.00
PA0350_folA_at	2.51	2.80	2.65	2.73	-0.03	1.00
PA0353_ilvD_at	5.06	4.91	4.76	5.05	0.08	1.00
PA0354_at	3.14	3.55	2.98	3.34	0.19	1.00
PA0356_at	3.81	3.62	3.60	3.64	0.10	1.00
PA0357_mutM_at	3.04	2.61	2.76	2.75	0.06	1.00
PA0360_at	3.70	3.63	3.65	3.84	-0.08	1.00
PA0363_coaD_at	3.98	4.18	4.13	3.97	0.03	1.00
PA0364_at	2.54	2.46	2.52	2.41	0.04	1.00
PA0371_at	3.05	3.00	3.32	2.85	-0.06	1.00
PA0372_at	3.41	3.55	3.47	3.36	0.06	1.00
PA0373_ftsY_at	5.18	5.09	5.07	5.02	0.09	1.00
PA0378_at	3.45	3.12	3.44	3.44	-0.15	1.00
PA0379_at	4.24	4.30	4.15	4.11	0.14	1.00
PA0380_i_at	2.69	3.21	3.12	2.94	-0.08	1.00
PA0381_thiG_at	3.29	3.09	3.24	3.07	0.03	1.00
PA0384_at	2.73	2.49	2.59	2.56	0.03	1.00
PA0385_at	3.35	3.39	3.04	3.37	0.16	1.00
PA0387_at	5.91	6.48	6.18	6.36	-0.07	1.00
PA0390_metX_at	3.97	3.88	3.77	3.83	0.13	1.00
PA0392_at	3.10	3.22	3.15	3.21	-0.02	1.00
PA0395_pilT_at	5.34	5.35	5.39	5.52	-0.11	1.00
PA0396_pilU_at	4.81	4.95	4.89	4.80	0.04	1.00
PA0398_at	3.04	3.03	3.02	2.90	0.08	1.00
PA0401_at	2.89	3.23	2.97	3.31	-0.08	1.00
PA0403_pyrR_at	6.72	6.79	6.87	6.70	-0.03	1.00
PA0406_at	4.27	3.92	3.94	4.33	-0.04	1.00
PA0409_pilH_at	7.91	8.91	8.47	8.73	-0.19	1.00
PA0410_pilI_at	4.27	4.98	4.47	4.36	0.21	1.00
PA0411_pilJ_at	4.04	4.52	4.44	4.42	-0.15	1.00
PA0412_pilK_at	4.33	4.27	4.28	4.36	-0.02	1.00
PA0414_at	3.29	3.54	3.43	3.58	-0.09	1.00
PA0415_at	5.33	5.54	5.34	5.47	0.03	1.00
PA0416_at	2.86	2.59	2.91	2.81	-0.13	1.00
PA0418_at	3.60	3.45	3.37	3.47	0.11	1.00
PA0420_bioA_at	3.15	3.19	2.86	3.33	0.08	1.00
PA0425_mexA_at	5.36	5.69	5.51	5.50	0.02	1.00
PA0426_mexB_at	5.01	5.12	5.14	4.89	0.05	1.00
PA0427_oprM_at	6.10	6.18	6.20	6.27	-0.10	1.00
PA0428_at	6.29	6.57	6.44	6.61	-0.09	1.00
PA0430_metF_at	2.96	3.40	3.59	3.12	-0.18	1.00

PA0431_at	4.86	5.38	5.18	4.87	0.09	1.00
PA0432_sahH_at	5.43	6.14	5.88	5.89	-0.10	1.00
PA0434_at	3.08	3.08	3.26	3.16	-0.13	1.00
PA0435_at	4.76	4.44	4.50	4.49	0.10	1.00
PA0437_codA_at	4.94	4.70	4.89	4.85	-0.05	1.00
PA0438_codB_at	2.95	3.04	3.08	3.10	-0.09	1.00
PA0439_at	2.69	2.69	2.57	2.72	0.04	1.00
PA0440_at	2.96	2.90	2.82	2.80	0.12	1.00
PA0441_at	3.23	3.06	2.75	3.22	0.16	1.00
PA0442_r_at	2.02	1.96	2.01	1.89	0.04	1.00
PA0443_at	3.47	2.92	3.14	3.50	-0.12	1.00
PA0446_at	3.21	3.22	3.41	3.26	-0.12	1.00
PA0447_gcdH_at	3.15	3.53	3.05	3.39	0.12	1.00
PA0448_at	2.60	2.39	2.45	2.42	0.06	1.00
PA0449_at	5.06	5.13	5.06	4.90	0.11	1.00
PA0453_at	3.20	3.04	3.24	3.15	-0.08	1.00
PA0454_at	3.56	3.59	3.53	3.56	0.03	1.00
PA0456_at	5.01	5.92	5.42	5.72	-0.10	1.00
PA0460_at	4.31	4.68	4.44	4.47	0.04	1.00
PA0464_creC_at	5.43	5.14	5.03	5.26	0.14	1.00
PA0465_creD_at	2.77	2.52	2.43	2.59	0.14	1.00
PA0466_at	4.37	4.10	4.19	4.43	-0.07	1.00
PA0467_at	5.44	5.79	5.67	5.78	-0.11	1.00
PA0468_at	4.12	4.38	4.19	4.16	0.08	1.00
PA0469_at	4.77	5.35	4.94	5.02	0.08	1.00
PA0470_at	3.13	3.02	3.12	3.08	-0.02	1.00
PA0471_at	2.92	2.78	2.68	2.98	0.02	1.00
PA0472_at	2.95	2.39	2.41	2.73	0.10	1.00
PA0473_at	2.93	2.83	2.96	2.98	-0.09	1.00
PA0474_at	2.84	2.85	2.97	2.80	-0.04	1.00
PA0475_at	3.22	2.92	3.26	3.11	-0.11	1.00
PA0476_at	3.83	3.87	3.87	3.72	0.06	1.00
PA0482_glcB_at	2.95	3.72	3.51	3.56	-0.20	1.00
PA0483_at	2.71	2.53	2.65	2.69	-0.04	1.00
PA0485_at	3.10	2.98	2.85	3.28	-0.03	1.00
PA0486_at	4.16	3.93	4.14	4.08	-0.07	1.00
PA0487_at	4.45	3.96	3.87	4.26	0.14	1.00
PA0488_at	4.57	4.27	4.51	4.49	-0.08	1.00
PA0490_at	3.73	3.82	3.85	3.50	0.10	1.00
PA0492_at	2.94	2.91	2.89	2.92	0.02	1.00
PA0493_at	2.21	2.28	2.26	2.30	-0.04	1.00
PA0494_at	3.13	2.79	2.97	2.90	0.02	1.00
PA0495_at	3.16	2.89	3.19	2.97	-0.05	1.00
PA0496_at	2.64	2.59	2.92	2.55	-0.11	1.00

PA0498_at	2.21	2.15	2.16	2.15	0.02	1.00
PA0499_at	3.38	3.04	3.22	2.87	0.16	1.00
PA0501_bioF_at	3.35	2.87	3.11	3.05	0.03	1.00
PA0504_bioD_at	3.17	3.04	3.19	2.92	0.05	1.00
PA0507_at	3.02	2.85	2.95	3.26	-0.17	1.00
PA0508_at	2.26	2.51	2.48	2.41	-0.06	1.00
PA0509_nirN_at	5.20	5.44	5.38	5.44	-0.09	1.00
PA0513_at	4.28	4.53	4.44	4.71	-0.17	1.00
PA0514_nirL_at	6.00	6.20	6.14	6.39	-0.17	1.00
PA0516_nirF_at	5.92	6.23	6.25	6.19	-0.15	1.00
PA0517_nirC_at	6.71	7.77	7.41	7.43	-0.18	1.00
PA0521_at	2.69	2.64	2.66	2.56	0.06	1.00
PA0527_dnr_at	2.98	2.94	2.94	3.16	-0.09	1.00
PA0531_at	3.55	3.83	3.58	3.53	0.13	1.00
PA0533_at	2.61	2.53	2.62	2.36	0.08	1.00
PA0534_at	5.87	6.26	6.18	6.22	-0.14	1.00
PA0536_at	5.34	5.80	5.62	5.33	0.10	1.00
PA0538_dsbB_at	4.15	4.72	4.54	4.63	-0.15	1.00
PA0539_at	3.10	2.64	2.90	2.35	0.24	1.00
PA0542_at	5.30	5.79	5.63	5.59	-0.07	1.00
PA0543_at	3.62	3.85	3.88	3.66	-0.03	1.00
PA0545_at	6.75	6.91	6.72	6.80	0.07	1.00
PA0547_at	4.15	4.77	4.33	4.44	0.08	1.00
PA0548_tktA_at	3.26	3.36	3.37	3.46	-0.10	1.00
PA0550_at	2.97	2.50	2.89	2.79	-0.10	1.00
PA0551_epd_at	4.05	4.33	4.24	4.32	-0.09	1.00
PA0552_pgk_at	3.80	3.88	3.74	4.10	-0.08	1.00
PA0553_at	6.04	6.59	6.40	6.40	-0.09	1.00
PA0555_fda_at	5.58	6.44	5.85	6.06	0.05	1.00
PA0556_at	2.24	2.42	2.36	2.20	0.05	1.00
PA0558_at	2.96	2.85	2.99	3.11	-0.15	1.00
PA0560_at	3.19	3.26	3.27	3.38	-0.10	1.00
PA0561_at	3.34	2.93	3.34	3.00	-0.04	1.00
PA0562_at	3.61	3.44	3.62	3.49	-0.03	1.00
PA0566_at	3.29	3.49	3.47	3.40	-0.05	1.00
PA0570_at	4.48	4.45	4.44	4.57	-0.04	1.00
PA0571_at	3.63	3.46	3.67	3.60	-0.09	1.00
PA0575_at	2.95	2.55	2.89	2.79	-0.09	1.00
PA0579_rpsU_at	6.31	7.43	6.42	7.05	0.13	1.00
PA0581_i_at	3.52	3.93	3.30	3.87	0.14	1.00
PA0582_folB_at	3.00	2.80	3.00	2.91	-0.05	1.00
PA0583_at	3.35	3.41	3.33	3.22	0.10	1.00
PA0584_cca_at	2.54	2.49	2.30	2.67	0.03	1.00
PA0586_at	6.05	6.05	6.07	6.24	-0.10	1.00

PA0589_at	2.76	3.01	2.85	2.72	0.10	1.00
PA0591_at	4.16	4.40	4.06	4.59	-0.04	1.00
PA0592_ksgA_at	3.41	3.40	3.31	3.43	0.04	1.00
PA0593_pdxA_at	3.52	3.59	3.71	3.68	-0.14	1.00
PA0594_surA_at	4.07	4.41	4.05	4.19	0.12	1.00
PA0597_at	3.41	3.49	3.23	3.53	0.07	1.00
PA0598_at	4.71	4.43	4.34	4.64	0.08	1.00
PA0599_at	3.30	2.99	3.04	3.04	0.11	1.00
PA0600_at	3.06	3.06	3.09	2.87	0.08	1.00
PA0601_at	2.73	2.77	2.76	2.84	-0.05	1.00
PA0605_at	2.82	2.70	2.75	2.91	-0.07	1.00
PA0609_trpE_at	4.16	3.97	4.08	4.23	-0.09	1.00
PA0644_at	2.55	2.91	2.64	2.53	0.14	1.00
PA0648_at	4.18	3.84	3.91	3.94	0.09	1.00
PA0650_trpD_at	3.50	3.86	3.84	3.80	-0.14	1.00
PA0651_trpC_at	2.73	2.92	2.82	2.89	-0.02	1.00
PA0652_vfr_at	7.47	7.87	7.84	7.59	-0.05	1.00
PA0654_speD_at	4.10	4.16	4.16	4.34	-0.12	1.00
PA0655_at	3.17	3.07	3.12	3.17	-0.03	1.00
PA0656_at	4.62	4.74	4.75	4.88	-0.13	1.00
PA0660_at	3.51	3.46	3.54	3.52	-0.04	1.00
PA0661_at	5.12	5.32	5.14	5.17	0.07	1.00
PA0662_argC_at	4.50	4.40	4.33	4.46	0.06	1.00
PA0663_at	4.93	4.59	4.74	5.03	-0.13	1.00
PA0664_at	5.53	5.41	5.47	5.40	0.03	1.00
PA0665_at	5.46	5.85	5.77	5.74	-0.10	1.00
PA0669_at	4.29	3.83	3.88	4.09	0.07	1.00
PA0670_at	3.62	3.32	3.53	3.26	0.07	1.00
PA0673_at	2.94	2.66	2.97	2.92	-0.15	1.00
PA0676_at	3.10	2.82	3.33	2.77	-0.09	1.00
PA0678_i_at	3.14	2.81	3.14	2.75	0.03	1.00
PA0680_at	2.67	2.56	2.72	2.65	-0.07	1.00
PA0681_at	2.71	2.66	2.75	2.75	-0.07	1.00
PA0682_at	4.10	3.54	3.87	4.08	-0.16	1.00
PA0683_at	2.77	2.68	2.89	2.76	-0.10	1.00
PA0684_at	3.56	3.21	3.06	3.39	0.16	1.00
PA0685_at	2.49	2.41	2.55	2.30	0.03	1.00
PA0686_at	3.17	2.81	2.92	2.92	0.07	1.00
PA0687_at	4.02	3.86	3.96	3.87	0.02	1.00
PA0688_at	2.69	2.43	2.45	2.47	0.10	1.00
PA0689_at	3.83	3.68	3.86	3.75	-0.05	1.00
PA0690_at	2.77	2.49	2.71	2.69	-0.07	1.00
PA0691_at	2.49	2.44	2.48	2.50	-0.03	1.00
PA0692_at	3.05	2.55	2.62	2.63	0.18	1.00

PA0693_exbB2_at	3.20	2.82	2.98	3.34	-0.15	1.00
PA0694_exbD2_at	5.25	5.11	5.20	5.30	-0.07	1.00
PA0695_at	4.66	4.24	4.58	4.60	-0.14	1.00
PA0698_at	2.50	2.28	2.50	2.51	-0.11	1.00
PA0706_cat_at	3.46	3.30	3.48	3.46	-0.09	1.00
PA0707_toxR_at	3.68	3.60	3.56	3.45	0.13	1.00
PA0708_at	3.74	3.56	3.47	3.62	0.10	1.00
PA0710_gloA2_at	3.17	2.76	3.09	2.67	0.09	1.00
PA0711_at	3.21	3.12	3.19	3.20	-0.03	1.00
PA0712_at	3.90	3.48	3.53	3.56	0.14	1.00
PA0715_at	2.40	2.89	2.71	2.64	-0.03	1.00
PA0717_at	11.22	10.18	10.93	11.14	-0.33	1.00
PA0718_at	12.83	12.27	12.65	12.65	-0.11	1.00
PA0729_at	3.49	7.18	6.59	6.43	-1.17	1.00
PA0731_at	4.55	4.68	4.52	4.47	0.13	1.00
PA0732_at	3.18	3.34	3.21	3.20	0.06	1.00
PA0734_i_at	3.20	3.07	3.01	3.08	0.09	1.00
PA0735_at	2.47	2.40	2.41	2.35	0.06	1.00
PA0737_at	4.77	4.95	4.90	4.71	0.06	1.00
PA0739_at	2.97	2.88	3.00	2.93	-0.04	1.00
PA0741_at	3.51	2.79	3.19	2.90	0.11	1.00
PA0742_at	3.19	3.04	3.21	3.25	-0.12	1.00
PA0746_at	3.69	3.57	3.86	3.63	-0.11	1.00
PA0747_at	4.74	4.76	4.83	4.92	-0.13	1.00
PA0748_at	3.36	3.23	2.93	3.38	0.14	1.00
PA0749_at	3.21	2.71	3.05	2.78	0.05	1.00
PA0750_ung_at	3.07	3.24	3.10	3.44	-0.12	1.00
PA0751_at	3.15	3.06	3.21	2.84	0.08	1.00
PA0753_at	2.82	2.59	2.94	2.74	-0.14	1.00
PA0755_at	2.94	2.75	2.72	2.88	0.04	1.00
PA0756_at	2.95	2.88	3.18	2.91	-0.13	1.00
PA0760_at	2.79	2.92	2.81	2.66	0.12	1.00
PA0761_nadB_at	3.53	3.93	4.09	3.67	-0.15	1.00
PA0762_algU_at	8.34	9.41	8.56	8.77	0.21	1.00
PA0765_mucC_at	6.54	7.26	6.72	6.72	0.18	1.00
PA0767_lepA_at	3.14	3.01	2.91	3.13	0.05	1.00
PA0768_lepB_at	4.60	5.39	4.92	4.71	0.18	1.00
PA0769_at	4.56	4.95	4.69	4.96	-0.07	1.00
PA0770_rnc_at	4.37	4.63	4.65	4.45	-0.04	1.00
PA0771_era_at	4.36	4.66	4.55	4.58	-0.06	1.00
PA0773_pdxJ_at	3.31	3.39	3.49	3.43	-0.11	1.00
PA0775_at	2.45	2.52	2.41	2.45	0.06	1.00
PA0776_at	3.22	3.17	3.57	3.07	-0.13	1.00

PA0778_at	4.42	4.49	4.31	4.45	0.08	1.00
PA0779_at	5.07	5.22	5.18	5.28	-0.08	1.00
PA0781_at	6.02	5.64	6.00	5.97	-0.15	1.00
PA0787_at	3.98	3.73	3.91	3.70	0.05	1.00
PA0789_at	4.22	4.30	4.20	4.24	0.04	1.00
PA0790_at	2.60	2.42	2.57	2.36	0.05	1.00
PA0792_prpD_at	4.44	4.40	4.40	4.38	0.03	1.00
PA0793_at	4.09	3.73	3.82	4.08	-0.04	1.00
PA0794_at	4.84	4.48	4.76	4.40	0.08	1.00
PA0795_prpC_at	2.99	3.12	3.01	3.38	-0.14	1.00
PA0796_prpB_at	2.56	2.59	2.59	2.71	-0.07	1.00
PA0797_at	2.76	3.05	2.72	2.79	0.15	1.00
PA0798_at	2.79	2.71	2.81	2.73	-0.03	1.00
PA0799_at	2.51	2.79	2.48	2.56	0.13	1.00
PA0801_at	5.80	5.52	5.66	5.44	0.11	1.00
PA0802_i_at	2.48	2.49	2.43	2.65	-0.06	1.00
PA0803_at	3.57	3.30	3.49	3.25	0.06	1.00
PA0806_i_at	3.99	4.08	4.07	4.09	-0.05	1.00
PA0808_at	2.84	2.50	2.49	2.75	0.05	1.00
PA0809_at	3.17	2.72	2.78	2.81	0.15	1.00
PA0811_at	3.63	3.26	3.36	3.30	0.12	1.00
PA0812_at	3.60	3.06	3.18	3.14	0.17	1.00
PA0813_at	3.56	3.34	3.55	3.23	0.06	1.00
PA0814_i_at	3.40	3.24	3.58	3.30	-0.12	1.00
PA0815_at	3.16	3.51	3.60	3.00	0.04	1.00
PA0816_at	3.43	3.25	3.34	3.50	-0.08	1.00
PA0817_at	3.28	3.45	3.39	3.21	0.07	1.00
PA0818_at	3.40	3.16	3.23	3.39	-0.03	1.00
PA0819_f_at	3.65	3.20	3.76	3.33	-0.12	1.00
PA0820_at	5.62	5.53	5.65	5.68	-0.09	1.00
PA0822_at	3.55	3.43	3.35	3.41	0.11	1.00
PA0824_at	2.83	2.32	2.77	2.74	-0.18	1.00
PA0825_at	2.66	2.49	2.52	2.72	-0.05	1.00
PA0826_at	3.42	3.34	3.36	3.34	0.03	1.00
PA0827_at	2.80	2.91	3.03	2.94	-0.12	1.00
PA0828_at	3.26	3.10	3.23	3.43	-0.15	1.00
PA0830_at	4.81	4.66	4.84	4.74	-0.06	1.00
PA0832_at	4.32	4.35	4.26	4.46	-0.03	1.00
PA0834_at	2.89	3.12	3.10	2.99	-0.04	1.00
PA0835_pta_at	5.51	6.05	5.85	6.00	-0.15	1.00
PA0836_at	7.39	7.89	7.90	7.76	-0.19	1.00
PA0839_at	2.30	2.40	2.32	2.28	0.05	1.00
PA0840_at	4.15	4.09	4.29	4.17	-0.11	1.00
PA0841_at	3.65	3.34	3.44	3.31	0.12	1.00

PA0842_at	3.30	2.94	3.33	3.13	-0.11	1.00
PA0843_plcR_at	2.87	2.67	2.70	2.73	0.05	1.00
PA0844_plcH_at	2.30	2.25	2.24	2.16	0.08	1.00
PA0846_at	2.53	2.53	2.53	2.27	0.13	1.00
PA0849_trxB2_at	3.14	2.92	2.88	3.03	0.07	1.00
PA0850_at	2.92	2.81	2.90	2.90	-0.04	1.00
PA0851_at	4.01	3.47	3.83	3.81	-0.08	1.00
PA0853_at	3.98	4.09	3.90	3.91	0.13	1.00
PA0855_at	3.35	3.17	3.13	3.28	0.05	1.00
PA0856_at	3.92	4.12	3.91	3.94	0.10	1.00
PA0858_at	3.60	3.57	3.46	3.49	0.11	1.00
PA0860_at	3.29	3.35	3.24	3.33	0.04	1.00
PA0861_at	4.49	4.59	4.72	4.53	-0.08	1.00
PA0863_at	2.96	2.73	3.03	2.80	-0.07	1.00
PA0864_at	4.10	3.44	3.67	3.42	0.23	1.00
PA0865_hpd_at	4.16	3.38	3.86	4.00	-0.16	1.00
PA0866_aroP2_at	3.67	3.45	3.42	3.44	0.13	1.00
PA0867_at	2.45	2.70	2.67	2.76	-0.14	1.00
PA0875_at	3.07	3.07	2.99	3.00	0.07	1.00
PA0877_at	3.85	3.78	3.80	3.69	0.07	1.00
PA0878_at	2.97	3.21	2.88	3.22	0.04	1.00
PA0880_at	3.07	2.73	2.80	2.76	0.11	1.00
PA0881_at	2.14	2.29	2.19	2.48	-0.12	1.00
PA0882_at	3.12	3.03	3.15	2.88	0.06	1.00
PA0884_at	3.33	3.09	3.16	3.11	0.07	1.00
PA0885_at	2.87	2.72	2.87	2.78	-0.03	1.00
PA0886_at	2.82	2.59	2.86	2.78	-0.11	1.00
PA0892_aotP_at	5.00	4.92	4.83	5.23	-0.07	1.00
PA0893_argR_at	3.59	4.03	4.01	3.91	-0.15	1.00
PA0896_aruF_at	3.02	2.74	2.57	2.84	0.17	1.00
PA0898_aruD_at	3.73	3.73	3.83	3.88	-0.13	1.00
PA0899_aruB_at	4.29	4.05	4.10	3.97	0.14	1.00
PA0901_aruE_at	4.89	5.11	5.08	4.99	-0.04	1.00
PA0903_alaS_at	4.36	4.50	4.41	4.51	-0.03	1.00
PA0904_lysC_at	4.99	5.56	5.43	5.48	-0.18	1.00
PA0905_csrA_at	6.28	6.46	6.16	6.37	0.10	1.00
PA0906_at	3.52	3.50	3.41	3.40	0.10	1.00
PA0908_at	3.42	3.19	3.31	3.58	-0.14	1.00
PA0910_at	2.76	2.95	2.61	3.01	0.04	1.00
PA0912_at	5.38	4.80	5.00	4.94	0.12	1.00
PA0914_at	3.72	3.12	3.37	3.57	-0.05	1.00
PA0916_at	3.80	3.65	3.63	3.56	0.12	1.00
PA0919_at	3.02	2.97	2.89	2.87	0.12	1.00
PA0921_at	2.97	2.99	3.08	2.96	-0.04	1.00

PA0924_at	3.44	3.34	3.46	3.21	0.06	1.00
PA0925_at	5.36	5.37	5.43	5.25	0.03	1.00
PA0926_at	2.79	3.05	2.98	2.97	-0.05	1.00
PA0928_at	3.91	4.14	3.92	4.06	0.03	1.00
PA0929_at	3.01	2.85	2.89	2.67	0.15	1.00
PA0930_at	3.54	3.23	3.36	3.31	0.05	1.00
PA0931_at	2.54	2.46	2.63	2.52	-0.08	1.00
PA0934_relA_at	6.49	6.42	6.36	6.44	0.06	1.00
PA0935_at	3.74	3.56	3.51	3.51	0.14	1.00
PA0936_at	4.61	4.75	4.56	4.59	0.11	1.00
PA0940_at	3.68	3.54	3.63	3.51	0.04	1.00
PA0941_at	3.99	3.87	3.91	3.79	0.08	1.00
PA0944_purN_at	3.30	4.05	3.29	3.62	0.22	1.00
PA0946_at	3.29	2.97	3.10	3.27	-0.05	1.00
PA0947_at	5.01	5.04	5.21	5.08	-0.12	1.00
PA0948_at	3.32	3.51	3.42	3.09	0.16	1.00
PA0950_at	5.58	6.04	5.59	5.63	0.20	1.00
PA0951_at	3.41	3.02	3.29	3.26	-0.06	1.00
PA0954_at	3.43	3.16	3.60	3.27	-0.14	1.00
PA0955_at	3.17	3.12	2.94	3.30	0.03	1.00
PA0956_proS_at	4.80	5.07	4.85	4.75	0.13	1.00
PA0957_at	3.54	3.30	3.48	3.44	-0.04	1.00
PA0960_at	2.59	2.78	2.65	2.82	-0.05	1.00
PA0963_aspS_at	3.03	3.64	3.26	3.18	0.12	1.00
PA0964_at	3.75	3.71	3.75	3.83	-0.06	1.00
PA0966_ruvA_at	4.79	5.34	4.87	4.89	0.18	1.00
PA0967_ruvB_at	4.39	4.44	4.37	4.29	0.09	1.00
PA0968_at	5.80	5.80	5.66	5.75	0.09	1.00
PA0969_tolQ_at	4.01	4.95	4.25	4.44	0.14	1.00
PA0973_oprL_at	6.52	6.81	6.66	7.06	-0.19	1.00
PA0974_at	5.12	5.36	5.10	5.15	0.12	1.00
PA0975_at	4.14	4.25	3.87	4.19	0.17	1.00
PA0976_at	4.58	4.50	4.47	4.56	0.02	1.00
PA0977_at	2.60	2.15	2.48	2.41	-0.07	1.00
PA0978_s_at	4.04	3.57	3.73	3.60	0.14	1.00
PA0979_s_at	2.51	2.62	2.63	2.60	-0.05	1.00
PA0981_at	2.96	2.85	2.71	2.84	0.13	1.00
PA0982_at	4.18	3.49	3.77	3.69	0.11	1.00
PA0983_at	5.07	4.79	5.34	4.65	-0.06	1.00
PA0984_at	3.17	2.73	2.57	2.92	0.21	1.00
PA0987_at	2.51	2.08	2.15	2.25	0.09	1.00
PA0988_at	4.46	4.22	4.42	4.34	-0.04	1.00
PA0990_at	3.13	3.18	2.93	3.23	0.07	1.00
PA0991_at	2.89	2.30	2.61	2.73	-0.07	1.00

PA0993_at	3.17	2.60	3.05	3.13	-0.20	1.00
PA0995_ogt_at	4.42	4.23	4.38	4.41	-0.07	1.00
PA0996_at	5.25	5.12	5.15	5.29	-0.04	1.00
PA1000_at	5.28	5.17	5.42	5.14	-0.05	1.00
PA1002_phnB_at	4.04	4.16	4.16	4.12	-0.04	1.00
PA1003_at	2.67	2.98	2.95	2.90	-0.10	1.00
PA1004_nadA_at	5.57	5.81	5.57	5.54	0.13	1.00
PA1005_at	3.28	3.15	3.13	3.14	0.08	1.00
PA1006_at	3.37	3.78	3.40	3.43	0.16	1.00
PA1010_dapA_at	5.76	6.16	5.90	5.71	0.16	1.00
PA1011_at	3.81	4.17	4.09	3.83	0.03	1.00
PA1012_at	3.94	3.66	3.97	3.89	-0.13	1.00
PA1013_purC_at	3.89	4.33	4.21	4.19	-0.09	1.00
PA1014_at	2.83	2.93	2.90	2.65	0.11	1.00
PA1016_at	3.13	2.84	3.04	3.01	-0.04	1.00
PA1017_pauA_at	3.30	2.70	3.16	3.07	-0.11	1.00
PA1018_at	2.63	2.84	2.69	2.44	0.17	1.00
PA1019_mucK_at	2.73	2.58	2.81	2.75	-0.12	1.00
PA1022_at	3.50	3.63	3.42	3.43	0.14	1.00
PA1023_at	2.56	2.47	2.65	2.57	-0.10	1.00
PA1024_at	3.15	3.36	3.32	3.44	-0.12	1.00
PA1028_at	4.56	4.29	4.37	4.62	-0.07	1.00
PA1030_at	5.25	5.61	5.25	5.38	0.11	1.00
PA1035_at	3.28	3.57	3.49	3.28	0.04	1.00
PA1037_at	2.90	2.87	2.87	2.94	-0.02	1.00
PA1038_at	3.45	3.57	3.51	3.58	-0.03	1.00
PA1039_at	4.33	4.17	4.28	4.31	-0.04	1.00
PA1043_at	4.84	4.76	5.04	4.73	-0.09	1.00
PA1044_at	4.83	5.05	4.92	4.65	0.16	1.00
PA1045_at	2.96	3.18	2.83	3.06	0.13	1.00
PA1051_at	2.97	2.95	2.92	2.87	0.07	1.00
PA1054_at	3.06	3.05	3.37	2.89	-0.08	1.00
PA1055_at	5.07	4.69	4.74	4.79	0.11	1.00
PA1057_at	3.19	3.08	3.21	3.16	-0.05	1.00
PA1058_at	3.92	3.70	3.73	3.65	0.12	1.00
PA1059_at	5.17	4.99	4.92	4.97	0.13	1.00
PA1061_at	3.20	3.16	3.04	3.17	0.08	1.00
PA1062_at	3.62	3.40	3.43	3.51	0.04	1.00
PA1063_at	2.92	2.76	2.88	2.74	0.03	1.00
PA1065_at	3.67	3.69	3.73	3.55	0.04	1.00
PA1070_braG_at	2.46	2.19	2.32	2.14	0.10	1.00
PA1071_braF_at	4.83	4.57	4.64	4.86	-0.04	1.00
PA1073_braD_at	3.03	2.78	3.16	2.81	-0.08	1.00
PA1075_at	5.23	5.40	5.25	5.71	-0.17	1.00

PA1076_at	4.27	4.34	4.15	4.38	0.04	1.00
PA1078_flgC_at	3.18	3.55	3.42	3.21	0.05	1.00
PA1079_flgD_at	3.26	3.04	3.42	3.20	-0.16	1.00
PA1080_flgE_at	4.46	4.87	4.84	4.62	-0.06	1.00
PA1085_flgJ_at	4.22	4.73	4.44	4.76	-0.12	1.00
PA1087_flgL_at	5.92	6.19	5.93	6.00	0.09	1.00
PA1089_at	3.59	3.06	3.17	3.20	0.14	1.00
PA1090_at	2.81	2.68	2.69	2.54	0.13	1.00
PA1092_fliC_at	8.35	8.78	8.38	8.63	0.06	1.00
PA1094_fliD_at	4.48	5.01	4.66	4.73	0.05	1.00
PA1097_fleQ_at	5.09	5.12	5.01	5.09	0.06	1.00
PA1099_fleR_at	4.34	4.98	4.57	4.92	-0.09	1.00
PA1104_fliI_at	4.88	4.77	5.02	4.86	-0.11	1.00
PA1106_at	2.57	2.73	2.47	2.71	0.06	1.00
PA1108_at	3.93	3.25	3.83	3.61	-0.13	1.00
PA1109_at	3.79	3.71	3.73	3.80	-0.02	1.00
PA1110_at	2.68	2.58	2.70	2.69	-0.06	1.00
PA1116_at	3.02	2.94	2.75	2.99	0.12	1.00
PA1119_at	3.66	3.83	3.86	3.80	-0.08	1.00
PA1120_at	2.47	2.67	2.65	2.72	-0.12	1.00
PA1121_at	2.77	2.57	2.67	2.58	0.05	1.00
PA1122_at	4.66	4.63	4.50	4.53	0.13	1.00
PA1124_dgt_at	2.57	2.58	2.80	2.63	-0.14	1.00
PA1126_at	3.95	3.68	3.84	3.52	0.14	1.00
PA1127_at	3.31	3.64	3.67	3.51	-0.12	1.00
PA1128_at	3.63	3.36	3.35	3.35	0.15	1.00
PA1129_at	2.95	2.74	2.87	2.66	0.08	1.00
PA1130_at	3.73	3.91	3.85	3.92	-0.06	1.00
PA1131_at	4.92	4.99	5.03	5.00	-0.06	1.00
PA1132_at	3.25	3.26	3.19	3.08	0.12	1.00
PA1134_at	3.74	3.23	3.38	3.47	0.06	1.00
PA1136_at	3.21	3.09	3.00	3.16	0.07	1.00
PA1138_at	3.60	3.13	3.27	3.91	-0.22	1.00
PA1139_at	3.07	3.28	3.22	3.16	-0.02	1.00
PA1140_at	2.85	2.64	2.92	2.80	-0.11	1.00
PA1141_at	3.95	3.46	3.83	3.49	0.04	1.00
PA1142_at	3.31	3.12	3.32	3.16	-0.02	1.00
PA1144_at	2.63	2.41	2.53	2.72	-0.11	1.00
PA1145_at	3.03	3.29	3.33	3.05	-0.03	1.00
PA1146_at	3.05	2.88	3.38	2.93	-0.19	1.00
PA1156_nrdA_at	3.43	3.89	3.60	3.61	0.06	1.00
PA1157_at	3.12	2.94	3.10	3.11	-0.07	1.00
PA1161_rrmA_at	3.28	3.00	3.09	2.90	0.14	1.00
PA1162_dapE_at	3.59	3.52	3.36	3.54	0.11	1.00

PA1163_at	3.02	3.01	3.03	2.87	0.06	1.00
PA1164_at	3.18	3.49	3.40	3.14	0.07	1.00
PA1169_at	3.92	3.49	3.90	3.63	-0.06	1.00
PA1171_at	2.62	2.48	2.64	2.49	-0.02	1.00
PA1179_phoP_at	2.81	2.89	2.86	2.70	0.07	1.00
PA1180_phoQ_at	3.43	3.10	3.29	3.31	-0.03	1.00
PA1181_at	5.13	5.10	5.11	4.94	0.09	1.00
PA1182_at	3.03	2.97	3.13	2.99	-0.06	1.00
PA1184_at	3.26	2.89	2.84	3.00	0.15	1.00
PA1185_at	4.36	3.82	4.41	4.23	-0.23	1.00
PA1186_at	4.20	3.59	4.21	3.71	-0.07	1.00
PA1187_at	4.09	3.42	3.63	3.36	0.26	1.00
PA1188_at	2.87	2.69	2.89	2.84	-0.09	1.00
PA1189_at	3.18	3.13	3.06	3.07	0.09	1.00
PA1190_at	3.74	3.63	3.73	3.75	-0.06	1.00
PA1191_at	4.12	4.17	4.09	4.09	0.05	1.00
PA1193_at	4.27	3.92	4.01	3.98	0.10	1.00
PA1197_at	2.77	2.93	2.77	2.71	0.11	1.00
PA1198_at	6.74	7.86	7.08	7.41	0.05	1.00
PA1205_at	4.36	4.09	4.44	4.13	-0.06	1.00
PA1207_kefB_at	3.18	3.02	2.81	3.23	0.08	1.00
PA1208_at	2.40	2.52	2.50	2.47	-0.02	1.00
PA1210_at	4.17	4.07	4.23	4.17	-0.08	1.00
PA1211_at	3.14	3.04	3.16	3.15	-0.07	1.00
PA1212_at	3.25	2.95	3.07	3.05	0.04	1.00
PA1213_at	3.09	2.61	2.88	3.05	-0.11	1.00
PA1214_at	3.02	3.08	3.08	3.16	-0.07	1.00
PA1215_at	3.10	2.74	2.92	3.06	-0.07	1.00
PA1219_at	3.68	3.41	3.77	3.52	-0.10	1.00
PA1220_at	2.91	2.75	3.06	2.73	-0.07	1.00
PA1221_at	3.72	3.25	3.20	3.40	0.18	1.00
PA1224_at	4.60	4.68	4.52	4.93	-0.09	1.00
PA1230_at	2.66	2.49	2.55	2.53	0.04	1.00
PA1232_at	2.68	2.77	2.67	2.59	0.10	1.00
PA1234_at	2.91	2.83	3.02	2.84	-0.06	1.00
PA1235_at	3.95	3.97	4.01	3.87	0.02	1.00
PA1236_at	4.75	4.32	4.50	4.47	0.05	1.00
PA1238_at	3.06	2.89	2.97	3.03	-0.03	1.00
PA1240_at	3.14	2.95	2.95	2.96	0.09	1.00
PA1242_at	3.34	3.28	3.17	3.20	0.12	1.00
PA1243_at	3.44	2.77	2.87	2.97	0.19	1.00
PA1247_aprE_at	3.87	3.59	3.70	4.02	-0.13	1.00
PA1250_aprI_at	3.60	3.58	3.68	3.62	-0.06	1.00
PA1251_at	2.38	2.45	2.11	2.41	0.16	1.00

PA1255_at	3.04	2.73	2.70	2.85	0.11	1.00
PA1256_at	2.96	2.73	2.84	2.89	-0.02	1.00
PA1257_at	2.85	2.58	2.82	2.69	-0.04	1.00
PA1258_at	3.03	2.77	2.99	2.92	-0.06	1.00
PA1260_at	4.83	4.80	4.73	5.02	-0.06	1.00
PA1261_at	2.61	2.48	2.55	2.39	0.07	1.00
PA1263_at	3.06	3.14	2.98	3.08	0.07	1.00
PA1264_at	2.89	2.75	2.77	2.93	-0.03	1.00
PA1265_at	3.52	3.44	3.36	3.53	0.03	1.00
PA1266_at	2.54	2.38	2.46	2.50	-0.02	1.00
PA1267_at	2.61	2.68	2.70	2.86	-0.14	1.00
PA1269_at	3.00	3.14	2.93	3.09	0.05	1.00
PA1270_at	3.81	3.65	3.62	3.60	0.12	1.00
PA1271_at	2.90	2.80	2.83	2.73	0.07	1.00
PA1273_cobB_at	3.72	3.57	3.54	3.60	0.08	1.00
PA1274_at	3.32	3.33	3.04	3.32	0.14	1.00
PA1275_cobD_at	4.18	4.01	4.28	3.82	0.05	1.00
PA1276_cobC_at	3.11	3.10	3.32	3.12	-0.11	1.00
PA1278_cobP_at	4.77	4.28	4.65	4.48	-0.04	1.00
PA1279_cobU_at	5.52	5.04	5.42	5.55	-0.20	1.00
PA1281_cobV_at	3.11	3.34	3.11	3.24	0.05	1.00
PA1283_at	4.34	4.53	4.47	4.72	-0.16	1.00
PA1284_at	3.03	3.09	2.81	3.19	0.06	1.00
PA1285_at	4.12	4.35	4.16	4.25	0.03	1.00
PA1286_at	2.93	2.75	2.80	2.74	0.07	1.00
PA1289_at	3.19	3.44	3.27	3.65	-0.15	1.00
PA1290_at	4.72	4.73	4.75	4.85	-0.07	1.00
PA1291_at	3.80	3.81	3.88	3.53	0.10	1.00
PA1294_rnd_at	5.00	5.03	4.94	4.95	0.07	1.00
PA1296_at	4.83	5.59	4.81	5.09	0.26	1.00
PA1302_at	3.88	3.68	3.80	3.80	-0.02	1.00
PA1303_at	2.46	2.32	2.52	2.35	-0.05	1.00
PA1308_at	4.44	4.96	4.56	4.65	0.10	1.00
PA1309_at	4.01	3.43	3.56	3.50	0.19	1.00
PA1310_phnW_at	2.75	2.61	2.79	2.54	0.02	1.00
PA1311_phnX_at	3.62	3.40	3.49	3.39	0.07	1.00
PA1313_at	3.20	3.12	2.97	3.15	0.10	1.00
PA1317_cyoA_at	2.68	3.00	2.97	2.89	-0.09	1.00
PA1318_cyoB_at	2.90	2.99	3.02	3.13	-0.13	1.00
PA1319_cyoC_at	2.91	2.84	2.78	2.89	0.04	1.00
PA1320_cyoD_at	2.57	2.08	2.26	2.57	-0.09	1.00
PA1321_cyoE_at	3.86	3.67	3.69	3.65	0.10	1.00
PA1322_at	2.78	2.55	2.77	2.64	-0.04	1.00
PA1324_at	4.95	5.36	5.26	5.13	-0.03	1.00

PA1328_at	3.91	4.05	4.06	4.10	-0.10	1.00
PA1332_at	3.29	3.14	3.27	3.01	0.08	1.00
PA1334_at	3.60	3.11	3.68	3.19	-0.07	1.00
PA1347_at	2.68	2.69	2.70	2.64	0.02	1.00
PA1349_at	2.93	2.87	3.01	2.89	-0.05	1.00
PA1350_at	3.91	4.08	3.88	4.05	0.03	1.00
PA1352_at	2.60	2.29	2.60	2.55	-0.13	1.00
PA1353_at	2.36	2.24	2.46	2.37	-0.11	1.00
PA1354_at	3.48	3.54	3.47	3.29	0.13	1.00
PA1356_at	2.99	2.97	3.13	3.09	-0.13	1.00
PA1357_at	2.91	2.91	3.05	2.99	-0.11	1.00
PA1358_at	3.41	3.75	3.43	3.51	0.11	1.00
PA1359_at	5.43	5.22	5.13	5.22	0.15	1.00
PA1361_at	2.68	2.78	2.82	2.72	-0.04	1.00
PA1364_at	3.37	3.23	3.15	3.27	0.09	1.00
PA1366_at	3.67	3.72	3.54	3.72	0.07	1.00
PA1367_at	4.14	3.63	4.05	3.98	-0.13	1.00
PA1370_at	3.03	2.68	2.70	2.69	0.16	1.00
PA1371_at	2.18	2.19	2.30	2.24	-0.09	1.00
PA1373_fabF2_at	3.98	4.18	4.24	4.04	-0.06	1.00
PA1375_pdxB_at	3.23	3.20	3.01	3.13	0.14	1.00
PA1376_aceK_at	3.52	3.74	3.77	3.78	-0.15	1.00
PA1379_at	2.78	2.82	2.83	2.73	0.02	1.00
PA1380_at	4.12	4.05	4.19	4.10	-0.06	1.00
PA1381_at	3.49	2.95	3.53	3.30	-0.20	1.00
PA1382_at	3.22	3.07	3.10	3.14	0.03	1.00
PA1384_galE_at	2.98	2.75	2.73	2.77	0.11	1.00
PA1385_at	3.72	2.81	3.37	3.44	-0.14	1.00
PA1386_at	3.56	3.41	3.71	3.46	-0.10	1.00
PA1387_at	2.93	2.75	3.04	2.85	-0.11	1.00
PA1388_at	3.55	3.31	3.30	3.46	0.05	1.00
PA1390_at	2.75	2.56	2.51	2.61	0.10	1.00
PA1391_at	2.83	2.83	3.02	2.80	-0.08	1.00
PA1395_at	3.11	3.17	3.15	3.29	-0.08	1.00
PA1396_at	3.16	2.54	2.82	3.03	-0.07	1.00
PA1397_at	2.86	3.04	2.80	3.01	0.05	1.00
PA1398_at	2.68	2.52	2.75	2.69	-0.12	1.00
PA1399_at	3.58	3.31	3.25	3.34	0.15	1.00
PA1400_at	2.89	2.61	2.66	2.77	0.04	1.00
PA1402_at	3.05	2.97	3.04	3.02	-0.02	1.00
PA1406_at	3.15	3.03	3.28	3.08	-0.09	1.00
PA1407_at	4.05	3.93	3.88	3.95	0.08	1.00
PA1408_at	3.21	3.14	2.96	3.18	0.10	1.00
PA1409_aphA_at	2.93	2.45	2.54	2.49	0.17	1.00

PA1410_at	3.32	3.17	3.40	3.14	-0.02	1.00
PA1412_at	2.71	2.47	2.87	2.63	-0.16	1.00
PA1413_at	3.43	3.59	3.39	3.34	0.15	1.00
PA1415_at	4.17	3.59	4.15	3.96	-0.17	1.00
PA1416_at	3.12	2.84	3.06	2.97	-0.03	1.00
PA1417_at	3.15	2.70	2.95	2.83	0.04	1.00
PA1419_at	3.40	3.53	3.75	3.03	0.08	1.00
PA1421_speB2_at	3.38	3.07	3.41	3.12	-0.04	1.00
PA1422_at	3.38	3.29	3.06	3.30	0.16	1.00
PA1423_at	4.89	4.97	4.56	4.99	0.16	1.00
PA1424_at	3.81	3.48	3.78	3.71	-0.10	1.00
PA1425_at	3.35	3.57	3.71	3.53	-0.16	1.00
PA1427_at	3.75	3.23	3.35	3.32	0.15	1.00
PA1428_at	2.60	2.38	2.65	2.58	-0.12	1.00
PA1431_rsaL_at	5.71	6.30	5.83	6.10	0.03	1.00
PA1435_at	3.78	3.95	3.77	3.83	0.07	1.00
PA1436_at	4.32	3.88	4.05	4.03	0.06	1.00
PA1438_at	3.42	3.22	3.53	3.42	-0.15	1.00
PA1439_at	4.21	4.58	4.51	4.19	0.04	1.00
PA1441_at	4.90	5.27	5.29	5.08	-0.10	1.00
PA1443_fliM_at	4.86	5.25	4.83	5.13	0.08	1.00
PA1446_fliP_at	3.52	3.66	3.57	3.69	-0.04	1.00
PA1448_fliR_at	3.68	3.23	3.37	3.33	0.11	1.00
PA1449_flhB_at	3.54	3.51	3.45	3.42	0.09	1.00
PA1450_at	3.56	3.51	3.57	3.45	0.02	1.00
PA1451_at	3.64	3.59	3.56	3.74	-0.04	1.00
PA1454_at	4.90	5.34	5.03	5.16	0.03	1.00
PA1455_fliA_at	6.17	6.72	6.24	6.25	0.20	1.00
PA1456_cheY_at	3.63	4.25	3.90	4.16	-0.09	1.00
PA1457_cheZ_at	4.28	5.31	4.93	4.88	-0.11	1.00
PA1459_at	5.36	5.94	5.63	5.56	0.06	1.00
PA1467_at	5.65	5.59	5.47	5.63	0.07	1.00
PA1468_at	3.39	4.05	3.69	3.67	0.05	1.00
PA1470_at	3.14	3.30	3.21	3.40	-0.08	1.00
PA1473_at	3.35	3.30	3.28	3.22	0.08	1.00
PA1478_at	2.85	3.48	2.99	3.45	-0.06	1.00
PA1480_ccmF_at	3.27	3.47	3.62	3.31	-0.10	1.00
PA1482_ccmH_at	3.73	4.22	3.99	4.05	-0.04	1.00
PA1484_at	3.69	3.84	3.73	3.68	0.06	1.00
PA1485_at	3.18	3.28	3.38	3.27	-0.10	1.00
PA1486_at	3.28	3.05	3.13	3.54	-0.17	1.00
PA1487_at	3.16	2.94	2.98	3.17	-0.03	1.00
PA1488_at	3.28	3.19	3.54	3.08	-0.08	1.00
PA1490_at	2.48	2.20	2.41	2.43	-0.08	1.00

PA1491_at	3.44	3.14	3.43	3.45	-0.15	1.00
PA1494_at	3.85	4.57	3.92	4.01	0.24	1.00
PA1496_at	2.82	2.71	2.76	2.67	0.05	1.00
PA1497_at	2.66	2.52	2.70	2.64	-0.08	1.00
PA1498_pykF_at	3.68	3.46	3.60	3.71	-0.09	1.00
PA1499_at	3.03	2.74	3.13	2.96	-0.16	1.00
PA1500_at	3.27	2.98	3.11	3.08	0.03	1.00
PA1502_gcl_at	2.84	2.51	2.66	2.40	0.15	1.00
PA1506_at	2.60	2.74	2.69	2.93	-0.14	1.00
PA1507_at	3.00	2.98	2.92	2.98	0.04	1.00
PA1508_at	5.00	5.02	5.14	5.01	-0.06	1.00
PA1510_at	2.41	2.40	2.51	2.36	-0.03	1.00
PA1511_at	3.39	2.80	2.84	2.96	0.19	1.00
PA1513_at	3.91	3.69	3.84	3.81	-0.03	1.00
PA1517_at	5.82	6.13	5.87	5.79	0.15	1.00
PA1518_at	3.15	3.44	3.25	3.14	0.10	1.00
PA1519_at	3.11	2.67	2.80	3.22	-0.11	1.00
PA1520_at	3.85	4.00	3.96	3.73	0.08	1.00
PA1521_at	3.95	3.78	3.92	3.65	0.08	1.00
PA1522_at	3.26	3.31	3.23	3.15	0.10	1.00
PA1523_xdhB_at	2.76	2.81	2.68	2.71	0.09	1.00
PA1524_xdhA_at	3.21	3.08	3.09	3.35	-0.07	1.00
PA1525_at	3.00	3.01	3.31	2.95	-0.13	1.00
PA1528_zipA_at	5.58	6.28	5.71	5.80	0.17	1.00
PA1530_at	4.43	4.28	4.64	4.31	-0.12	1.00
PA1533_at	5.70	6.04	5.79	5.85	0.05	1.00
PA1534_recR_at	3.67	3.71	3.58	3.54	0.13	1.00
PA1536_at	2.79	2.42	2.42	2.61	0.09	1.00
PA1541_at	3.48	3.43	3.42	3.30	0.09	1.00
PA1542_at	2.78	2.64	2.69	2.84	-0.05	1.00
PA1544_anr_at	5.75	6.88	6.01	6.29	0.16	1.00
PA1548_at	3.11	3.04	3.01	3.20	-0.03	1.00
PA1550_at	4.96	5.83	5.46	5.59	-0.13	1.00
PA1552_at	3.32	3.68	3.93	3.52	-0.22	1.00
PA1558_at	2.88	2.92	2.76	2.89	0.08	1.00
PA1560_at	3.30	2.99	2.99	3.01	0.14	1.00
PA1561_aer_at	5.23	5.78	5.55	5.14	0.16	1.00
PA1562_acnA_at	3.81	3.72	3.57	3.75	0.10	1.00
PA1563_at	4.48	4.13	4.35	3.92	0.17	1.00
PA1567_at	3.74	3.68	3.65	3.65	0.06	1.00
PA1568_at	3.12	2.88	2.94	2.73	0.16	1.00
PA1569_at	2.78	2.39	2.53	2.69	-0.03	1.00
PA1570_at	3.01	2.59	2.94	2.99	-0.17	1.00
PA1572_at	3.24	3.13	3.19	3.31	-0.07	1.00

PA1574_at	3.00	2.82	3.02	2.76	0.02	1.00
PA1576_at	3.28	3.06	3.24	3.16	-0.03	1.00
PA1577_at	3.03	2.83	3.09	2.91	-0.07	1.00
PA1578_at	5.11	4.88	5.11	4.77	0.06	1.00
PA1579_at	3.32	3.20	3.14	3.25	0.07	1.00
PA1581_sdhC_at	5.41	6.23	6.02	5.73	-0.05	1.00
PA1582_sdhD_at	4.44	5.41	5.29	5.22	-0.33	1.00
PA1591_at	4.49	4.63	4.52	4.45	0.07	1.00
PA1593_at	2.90	3.18	2.85	2.92	0.16	1.00
PA1594_at	2.64	2.63	2.71	2.66	-0.05	1.00
PA1595_at	3.50	3.34	3.26	3.38	0.10	1.00
PA1596_htpG_at	6.65	7.27	6.88	7.23	-0.10	1.00
PA1597_at	3.42	3.60	3.49	3.65	-0.06	1.00
PA1598_at	2.94	2.83	2.66	3.02	0.04	1.00
PA1599_at	3.22	2.76	2.81	2.84	0.17	1.00
PA1602_at	5.83	6.12	5.98	6.04	-0.04	1.00
PA1603_at	4.62	4.73	4.65	4.59	0.06	1.00
PA1604_at	5.91	5.87	5.95	6.03	-0.10	1.00
PA1609_fabB_at	4.26	4.58	4.43	4.69	-0.14	1.00
PA1610_fabA_at	5.28	5.67	5.49	5.64	-0.09	1.00
PA1612_at	2.87	2.69	2.62	2.65	0.14	1.00
PA1614_gpsA_at	4.00	4.42	4.08	4.08	0.13	1.00
PA1615_at	3.47	3.79	3.67	3.91	-0.16	1.00
PA1617_at	4.72	4.61	4.49	4.63	0.11	1.00
PA1618_at	3.49	3.53	3.48	3.43	0.05	1.00
PA1621_at	5.84	5.72	5.97	5.74	-0.08	1.00
PA1623_at	2.94	2.88	2.74	2.92	0.08	1.00
PA1625_at	4.03	4.15	4.09	3.93	0.08	1.00
PA1626_at	4.68	4.12	4.36	4.25	0.10	1.00
PA1627_at	2.92	2.90	2.91	2.84	0.03	1.00
PA1628_at	3.26	3.22	3.26	3.10	0.06	1.00
PA1629_at	2.97	3.19	3.29	3.11	-0.12	1.00
PA1630_at	4.35	4.02	4.04	4.14	0.10	1.00
PA1631_at	3.52	3.23	3.45	3.22	0.04	1.00
PA1632_kdpF_at	4.17	3.25	4.11	3.76	-0.22	1.00
PA1633_kdpA_at	3.85	3.53	4.01	3.63	-0.12	1.00
PA1637_kdpE_at	3.57	3.21	3.34	3.30	0.07	1.00
PA1638_at	3.40	3.25	3.36	3.37	-0.04	1.00
PA1639_at	3.29	3.21	3.35	3.31	-0.08	1.00
PA1640_at	2.95	3.09	3.17	3.08	-0.10	1.00
PA1641_at	4.82	4.91	4.92	4.71	0.05	1.00
PA1642_selD_at	4.53	4.30	4.39	4.29	0.08	1.00
PA1643_at	3.52	3.76	3.50	3.66	0.06	1.00
PA1644_at	3.48	3.37	3.36	3.40	0.04	1.00

PA1645_at	2.78	2.91	2.81	2.69	0.09	1.00
PA1648_at	3.26	3.21	3.54	3.16	-0.12	1.00
PA1651_at	3.00	2.86	2.87	2.94	0.02	1.00
PA1652_at	2.79	2.49	2.52	2.61	0.07	1.00
PA1653_at	4.21	4.19	4.31	4.23	-0.07	1.00
PA1655_at	3.38	3.38	3.48	3.40	-0.06	1.00
PA1656_at	3.72	4.49	4.01	4.80	-0.29	1.00
PA1660_at	3.42	3.22	3.42	3.38	-0.08	1.00
PA1661_at	4.48	4.32	4.36	4.14	0.14	1.00
PA1663_at	4.79	4.80	4.75	4.69	0.07	1.00
PA1664_at	6.01	6.45	6.63	6.11	-0.14	1.00
PA1670_stp1_at	5.28	4.69	5.22	5.01	-0.13	1.00
PA1673_at	5.67	5.88	5.93	5.67	-0.03	1.00
PA1674_folE2_at	3.52	3.72	3.67	3.41	0.08	1.00
PA1677_at	6.12	6.51	6.35	6.47	-0.09	1.00
PA1678_at	4.32	4.06	4.18	4.11	0.05	1.00
PA1679_at	4.75	4.81	4.77	4.58	0.11	1.00
PA1681_aroC_at	5.64	5.70	5.60	5.60	0.07	1.00
PA1682_at	4.04	3.94	3.90	4.02	0.03	1.00
PA1684_at	4.28	4.28	4.26	4.24	0.03	1.00
PA1686_alkA_at	4.13	4.19	4.12	4.15	0.03	1.00
PA1687_speE_at	2.57	2.46	2.62	2.69	-0.14	1.00
PA1689_at	3.45	3.27	3.42	3.56	-0.13	1.00
PA1690_pscU_at	3.09	3.18	3.20	3.01	0.03	1.00
PA1691_pscT_at	2.34	2.64	2.53	2.31	0.07	1.00
PA1693_pscR_at	2.88	3.06	2.92	2.84	0.09	1.00
PA1695_pscP_at	4.75	5.66	5.42	5.59	-0.30	1.00
PA1696_pscO_at	4.14	4.76	4.62	4.49	-0.11	1.00
PA1697_at	6.31	7.25	6.66	7.19	-0.15	1.00
PA1700_at	5.43	6.46	6.38	6.15	-0.32	1.00
PA1701_at	5.60	7.06	6.73	6.89	-0.48	1.00
PA1702_i_at	5.24	5.96	5.72	5.63	-0.07	1.00
PA1703_pcrD_at	5.04	5.85	5.49	5.49	-0.05	1.00
PA1704_pcrR_at	3.91	4.57	4.28	4.27	-0.03	1.00
PA1705_pcrG_i_at	4.56	5.39	5.42	5.10	-0.29	1.00
PA1710_exsC_at	4.91	6.04	5.66	6.01	-0.36	1.00
PA1711_at	6.33	7.84	7.41	7.17	-0.20	1.00
PA1712_exsB_at	6.10	7.09	6.76	6.99	-0.28	1.00
PA1715_pscB_at	4.14	6.12	5.48	5.78	-0.50	1.00
PA1716_pscC_at	6.25	7.40	7.12	6.76	-0.12	1.00
PA1718_pscE_at	5.63	6.92	6.45	6.80	-0.35	1.00
PA1721_pscH_at	4.41	5.06	4.98	4.91	-0.21	1.00
PA1722_pscI_at	5.80	6.44	5.81	6.06	0.18	1.00
PA1723_pscJ_at	4.16	5.01	4.56	4.79	-0.09	1.00

PA1725_pscL_at	4.96	5.38	5.09	4.99	0.13	1.00
PA1726_bglX_at	5.23	5.17	5.24	5.31	-0.08	1.00
PA1727_at	3.75	3.39	3.57	3.36	0.11	1.00
PA1729_at	6.71	6.83	6.63	6.86	0.02	1.00
PA1730_at	3.66	3.84	3.73	3.70	0.04	1.00
PA1731_at	3.37	3.63	3.11	3.79	0.05	1.00
PA1732_at	3.84	3.96	3.97	3.92	-0.04	1.00
PA1733_at	3.52	3.53	3.70	3.57	-0.11	1.00
PA1734_at	3.06	2.95	2.94	2.96	0.05	1.00
PA1735_at	2.99	2.89	2.86	2.79	0.11	1.00
PA1739_at	4.41	3.76	4.09	3.99	0.04	1.00
PA1740_at	4.11	3.77	3.80	4.15	-0.04	1.00
PA1741_at	4.31	4.10	4.25	4.27	-0.05	1.00
PA1742_at	5.45	5.71	5.50	5.75	-0.05	1.00
PA1747_at	2.68	2.70	2.73	2.83	-0.09	1.00
PA1748_at	2.98	3.22	3.32	2.75	0.06	1.00
PA1749_at	3.38	3.44	3.37	3.50	-0.03	1.00
PA1751_at	3.67	3.66	3.58	3.63	0.06	1.00
PA1752_at	5.18	5.18	5.21	5.02	0.07	1.00
PA1753_at	5.67	6.11	5.88	6.25	-0.18	1.00
PA1754_cysB_at	5.33	6.15	5.91	5.93	-0.18	1.00
PA1755_at	3.36	3.12	3.25	3.29	-0.03	1.00
PA1756_cysH_at	3.62	3.56	3.72	3.63	-0.09	1.00
PA1757_thrH_at	3.53	3.40	3.46	3.18	0.15	1.00
PA1758_pabB_at	4.10	3.57	3.71	4.06	-0.05	1.00
PA1760_at	3.60	3.65	3.51	3.81	-0.04	1.00
PA1761_at	3.32	3.37	3.40	3.44	-0.07	1.00
PA1762_at	3.32	2.95	3.28	3.19	-0.09	1.00
PA1763_at	2.62	2.61	2.78	2.70	-0.13	1.00
PA1765_at	2.24	2.41	2.45	2.47	-0.14	1.00
PA1767_at	3.68	3.88	3.82	3.93	-0.09	1.00
PA1768_at	5.37	5.96	5.42	5.51	0.20	1.00
PA1771_at	3.03	2.83	2.69	2.89	0.14	1.00
PA1772_at	3.78	4.44	3.90	4.17	0.08	1.00
PA1774_at	5.35	5.68	5.32	5.37	0.17	1.00
PA1776_at	5.21	6.49	5.69	5.27	0.37	1.00
PA1777_oprF_at	7.03	8.08	7.73	7.95	-0.29	1.00
PA1779_at	2.21	2.34	2.27	2.20	0.05	1.00
PA1780_nirD_at	2.63	2.52	2.85	2.38	-0.04	1.00
PA1781_nirB_at	3.32	3.10	3.09	3.17	0.08	1.00
PA1783_nasA_at	3.01	3.01	3.05	3.12	-0.08	1.00
PA1785_at	3.56	3.19	3.45	3.43	-0.06	1.00
PA1788_at	2.74	2.74	2.82	2.75	-0.04	1.00
PA1790_at	4.31	4.17	4.13	4.07	0.14	1.00

PA1791_at	3.29	3.28	3.20	3.54	-0.08	1.00
PA1792_at	4.38	4.20	4.15	4.16	0.13	1.00
PA1795_cysS_at	5.81	5.94	5.82	5.71	0.11	1.00
PA1796_folD_at	3.36	3.67	3.35	3.60	0.05	1.00
PA1797_at	3.80	3.07	3.23	3.13	0.25	1.00
PA1799_at	3.46	3.50	3.39	3.30	0.14	1.00
PA1800_tig_at	5.81	6.68	6.11	6.04	0.17	1.00
PA1801_clpP_at	4.11	4.95	4.79	4.89	-0.31	1.00
PA1802_clpX_at	5.26	5.33	5.18	5.15	0.13	1.00
PA1803_lon_at	3.47	3.93	3.59	3.67	0.07	1.00
PA1804_hupB_at	7.03	7.23	6.95	7.19	0.06	1.00
PA1807_at	3.12	3.12	3.06	3.31	-0.07	1.00
PA1808_at	3.33	3.32	3.40	3.40	-0.07	1.00
PA1809_at	3.42	3.27	3.22	3.52	-0.02	1.00
PA1810_at	3.82	3.52	3.59	3.68	0.03	1.00
PA1814_at	5.58	5.76	5.75	5.66	-0.04	1.00
PA1815_rnhA_at	3.58	3.59	3.58	3.32	0.13	1.00
PA1816_dnaQ_at	2.84	3.16	2.81	2.88	0.15	1.00
PA1817_at	2.91	2.77	2.98	2.89	-0.10	1.00
PA1819_at	3.13	2.80	3.02	3.03	-0.06	1.00
PA1820_nhaB_at	3.35	3.21	3.37	2.96	0.12	1.00
PA1821_at	4.66	4.85	5.08	4.76	-0.16	1.00
PA1823_at	4.20	4.16	4.33	3.96	0.03	1.00
PA1824_at	2.81	2.56	2.69	2.53	0.08	1.00
PA1826_at	3.66	3.34	3.48	3.41	0.05	1.00
PA1827_at	3.42	2.95	3.43	3.29	-0.17	1.00
PA1828_at	2.44	2.34	2.37	2.44	-0.02	1.00
PA1833_at	5.19	5.14	5.17	5.31	-0.08	1.00
PA1834_at	3.43	3.22	3.17	3.30	0.09	1.00
PA1835_at	3.00	3.01	2.86	3.10	0.02	1.00
PA1836_at	3.10	3.35	2.97	3.19	0.15	1.00
PA1839_at	6.00	5.55	5.67	5.79	0.04	1.00
PA1840_at	3.59	3.60	3.62	3.53	0.02	1.00
PA1841_at	2.42	2.51	2.49	2.48	-0.02	1.00
PA1842_at	2.59	2.61	2.61	2.39	0.10	1.00
PA1844_at	3.60	3.17	3.41	3.55	-0.09	1.00
PA1845_at	3.29	3.21	3.54	3.23	-0.14	1.00
PA1846_cti_at	2.85	2.89	3.30	2.81	-0.18	1.00
PA1847_at	5.77	6.62	5.81	6.04	0.27	1.00
PA1851_at	2.53	2.61	2.58	2.70	-0.07	1.00
PA1856_at	3.23	2.98	3.16	3.11	-0.03	1.00
PA1857_at	3.30	3.64	3.40	3.61	-0.04	1.00
PA1860_at	5.06	4.44	4.87	4.97	-0.17	1.00
PA1861_modC_at	3.35	3.61	3.26	3.41	0.15	1.00

PA1862_modB_at	3.21	3.66	3.27	3.42	0.09	1.00
PA1864_at	3.94	4.18	3.97	4.06	0.04	1.00
PA1866_at	4.95	4.25	4.28	4.62	0.15	1.00
PA1868_xqhA_at	3.57	3.34	3.42	3.60	-0.05	1.00
PA1870_at	3.15	2.99	3.00	3.08	0.03	1.00
PA1873_at	3.88	3.65	4.05	3.59	-0.05	1.00
PA1874_at	3.62	3.15	3.36	3.77	-0.18	1.00
PA1877_at	3.16	3.02	2.97	3.27	-0.03	1.00
PA1878_at	3.72	3.05	3.65	3.39	-0.13	1.00
PA1879_at	4.31	4.16	4.15	4.06	0.13	1.00
PA1880_at	3.70	3.67	3.83	3.75	-0.10	1.00
PA1881_at	3.24	3.04	3.19	3.19	-0.05	1.00
PA1882_at	3.21	2.68	2.93	2.81	0.08	1.00
PA1884_at	2.96	2.54	2.63	2.68	0.10	1.00
PA1886_polB_at	2.61	2.36	2.67	2.44	-0.07	1.00
PA1889_at	3.72	3.57	3.50	3.59	0.10	1.00
PA1892_at	2.96	2.59	2.78	2.57	0.10	1.00
PA1894_at	2.70	2.66	2.68	2.81	-0.07	1.00
PA1895_at	3.06	2.90	3.05	3.17	-0.13	1.00
PA1896_at	2.88	2.59	2.73	2.57	0.08	1.00
PA1897_at	2.89	2.92	3.01	2.92	-0.06	1.00
PA1898_at	3.16	3.38	3.34	3.14	0.03	1.00
PA1907_at	2.55	2.24	2.35	2.30	0.07	1.00
PA1909_at	2.63	2.54	2.72	2.55	-0.05	1.00
PA1910_at	4.12	3.31	3.94	3.40	0.05	1.00
PA1911_at	3.12	2.83	3.05	2.99	-0.05	1.00
PA1912_at	3.10	3.02	3.10	3.22	-0.10	1.00
PA1914_at	2.76	2.73	2.96	2.64	-0.05	1.00
PA1916_at	3.48	2.96	3.36	3.49	-0.20	1.00
PA1918_at	3.48	3.11	3.37	3.57	-0.18	1.00
PA1920_at	3.66	3.77	3.43	3.76	0.12	1.00
PA1921_at	3.11	2.58	2.66	2.69	0.17	1.00
PA1922_at	2.89	2.72	2.69	2.78	0.07	1.00
PA1923_at	2.90	2.72	2.91	2.96	-0.13	1.00
PA1925_at	2.68	2.20	2.48	2.52	-0.06	1.00
PA1927_metE_at	2.60	2.49	2.63	2.61	-0.07	1.00
PA1928_rimJ_at	5.71	5.46	5.56	5.55	0.03	1.00
PA1929_i_at	3.49	3.03	3.05	3.55	-0.04	1.00
PA1931_at	4.52	4.24	4.68	4.43	-0.17	1.00
PA1932_at	2.91	2.74	2.95	2.93	-0.12	1.00
PA1934_at	3.54	3.64	3.52	3.48	0.09	1.00
PA1939_at	3.11	3.01	2.83	3.03	0.13	1.00
PA1941_at	3.98	3.61	3.83	3.84	-0.04	1.00
PA1944_at	2.69	2.95	2.81	2.71	0.06	1.00

PA1945_at	3.72	3.60	3.61	3.54	0.08	1.00
PA1948_rbsC_at	4.94	4.53	4.91	4.71	-0.07	1.00
PA1949_rbsR_at	5.25	5.06	5.21	5.00	0.05	1.00
PA1950_rbsK_at	3.69	3.79	3.79	3.77	-0.04	1.00
PA1951_at	2.60	2.49	2.59	2.44	0.03	1.00
PA1952_at	3.92	3.18	3.67	3.51	-0.04	1.00
PA1953_at	2.78	2.55	2.58	2.44	0.15	1.00
PA1954_at	4.04	3.39	3.74	3.86	-0.09	1.00
PA1955_at	2.33	2.14	2.48	2.30	-0.15	1.00
PA1956_at	2.60	2.33	2.46	2.55	-0.03	1.00
PA1960_at	3.27	3.15	3.11	3.24	0.04	1.00
PA1961_at	2.62	2.25	2.55	2.60	-0.14	1.00
PA1962_at	2.41	2.44	2.54	2.48	-0.09	1.00
PA1963_at	3.03	2.74	2.90	2.71	0.08	1.00
PA1966_at	3.78	3.64	3.81	3.80	-0.09	1.00
PA1967_at	2.72	2.70	2.80	2.76	-0.07	1.00
PA1968_at	3.23	3.43	3.28	3.48	-0.05	1.00
PA1969_at	3.35	3.60	3.48	3.63	-0.08	1.00
PA1970_at	2.26	2.25	2.28	2.31	-0.04	1.00
PA1972_at	2.47	2.40	2.57	2.51	-0.10	1.00
PA1973_pqqF_at	3.01	2.90	2.97	2.88	0.03	1.00
PA1974_at	2.60	2.40	2.48	2.61	-0.04	1.00
PA1975_at	4.06	3.60	3.91	3.88	-0.07	1.00
PA1978_at	3.62	2.81	3.04	3.16	0.12	1.00
PA1979_at	2.42	2.28	2.28	2.26	0.08	1.00
PA1980_at	2.99	2.47	2.70	2.67	0.04	1.00
PA1981_at	2.54	2.56	2.79	2.60	-0.15	1.00
PA1982_exaA_at	3.37	3.34	3.42	3.24	0.03	1.00
PA1983_exaB_at	3.19	2.63	2.82	2.93	0.03	1.00
PA1985_pqqA_at	2.47	2.38	2.42	2.47	-0.02	1.00
PA1987_pqqC_at	3.38	3.29	3.40	3.07	0.10	1.00
PA1988_pqqD_at	3.03	3.04	3.03	3.36	-0.16	1.00
PA1990_at	3.68	3.07	3.29	3.22	0.13	1.00
PA1992_at	2.73	2.84	3.03	2.69	-0.07	1.00
PA1993_at	2.74	2.43	2.59	2.50	0.04	1.00
PA1994_at	2.90	2.58	2.43	2.86	0.10	1.00
PA1999_at	3.22	3.02	3.11	3.27	-0.07	1.00
PA2002_at	3.26	2.96	3.01	2.99	0.11	1.00
PA2003_bdhA_at	5.03	4.46	4.94	4.81	-0.13	1.00
PA2004_at	2.95	2.55	2.65	2.75	0.05	1.00
PA2005_at	2.58	2.45	2.65	2.32	0.03	1.00
PA2009_hmgA_at	3.48	3.48	3.47	3.72	-0.11	1.00
PA2011_at	3.74	3.48	3.68	3.44	0.05	1.00
PA2012_at	3.36	3.37	3.32	3.56	-0.08	1.00

PA2014_at	3.74	3.79	3.68	3.68	0.08	1.00
PA2015_at	6.27	6.69	6.39	6.68	-0.06	1.00
PA2017_at	3.96	3.99	4.21	3.96	-0.11	1.00
PA2018_at	4.23	4.17	4.40	4.25	-0.12	1.00
PA2021_at	3.72	4.10	3.84	3.72	0.13	1.00
PA2022_at	3.25	3.15	3.27	2.97	0.08	1.00
PA2025_gor_at	5.26	5.34	5.39	5.14	0.03	1.00
PA2026_at	3.07	2.92	2.83	3.04	0.06	1.00
PA2030_at	2.75	2.89	3.12	2.76	-0.12	1.00
PA2033_at	2.89	2.43	2.59	2.79	-0.03	1.00
PA2034_at	2.98	2.84	2.78	2.87	0.09	1.00
PA2035_at	2.76	2.77	2.75	2.73	0.02	1.00
PA2037_at	2.61	2.51	2.75	2.62	-0.12	1.00
PA2038_at	2.90	2.85	2.84	2.97	-0.03	1.00
PA2041_at	3.38	3.06	3.27	3.12	0.02	1.00
PA2042_at	4.37	4.53	4.49	4.51	-0.05	1.00
PA2043_at	3.09	2.76	3.04	2.68	0.07	1.00
PA2044_at	3.95	4.02	3.68	3.98	0.16	1.00
PA2046_at	4.33	3.78	4.16	4.20	-0.12	1.00
PA2047_at	2.79	2.74	2.90	2.73	-0.05	1.00
PA2049_at	3.17	3.33	3.35	3.21	-0.04	1.00
PA2050_at	2.98	3.01	3.24	2.99	-0.12	1.00
PA2051_at	3.42	3.09	3.30	3.10	0.06	1.00
PA2052_cynS_at	2.37	2.14	2.38	2.45	-0.16	1.00
PA2054_cynR_at	3.68	3.75	3.61	3.65	0.08	1.00
PA2056_at	3.57	3.33	3.38	3.28	0.12	1.00
PA2057_at	3.83	3.34	3.44	3.65	0.04	1.00
PA2058_at	3.01	2.36	2.91	2.85	-0.20	1.00
PA2059_at	2.79	2.66	2.99	2.66	-0.10	1.00
PA2060_at	3.21	2.93	3.10	3.21	-0.08	1.00
PA2062_at	4.37	4.08	4.26	4.24	-0.02	1.00
PA2064_pcoB_at	4.05	3.58	3.96	4.03	-0.18	1.00
PA2065_pcoA_at	2.99	2.58	2.77	2.60	0.10	1.00
PA2067_at	3.74	3.50	3.56	4.06	-0.19	1.00
PA2068_at	2.78	2.27	2.73	2.56	-0.12	1.00
PA2069_at	3.89	3.41	3.93	3.74	-0.18	1.00
PA2071_fusA2_at	4.05	4.01	4.17	4.08	-0.10	1.00
PA2072_at	3.49	3.12	3.36	3.33	-0.04	1.00
PA2074_at	3.88	3.19	3.79	3.53	-0.13	1.00
PA2077_at	3.73	3.41	3.63	3.44	0.04	1.00
PA2078_at	3.00	3.02	3.11	3.08	-0.09	1.00
PA2079_at	2.55	2.36	2.45	2.51	-0.02	1.00
PA2080_at	4.09	4.09	4.14	4.32	-0.14	1.00
PA2081_at	3.96	3.81	3.99	3.93	-0.07	1.00

PA2083_at	2.82	2.46	2.55	2.55	0.10	1.00
PA2086_at	3.32	3.26	3.31	3.36	-0.04	1.00
PA2087_at	2.68	2.49	2.57	2.49	0.05	1.00
PA2088_at	2.68	2.53	2.73	2.65	-0.09	1.00
PA2090_at	2.54	2.43	2.46	2.69	-0.09	1.00
PA2093_at	2.33	2.19	2.32	2.26	-0.02	1.00
PA2097_at	2.76	2.61	2.81	2.63	-0.03	1.00
PA2099_at	3.03	2.47	2.95	2.98	-0.22	1.00
PA2100_at	3.27	3.49	3.23	3.35	0.09	1.00
PA2102_at	2.91	3.00	2.97	3.05	-0.06	1.00
PA2103_at	4.90	5.03	4.88	4.82	0.11	1.00
PA2105_at	3.69	3.86	3.67	3.71	0.09	1.00
PA2108_at	3.24	2.99	3.22	3.06	-0.03	1.00
PA2110_at	3.01	2.71	3.08	2.87	-0.11	1.00
PA2111_at	2.46	2.37	2.56	2.54	-0.13	1.00
PA2112_at	2.29	2.54	2.57	2.47	-0.11	1.00
PA2118_ada_at	3.07	2.76	2.82	2.87	0.07	1.00
PA2121_at	2.61	3.03	2.70	2.87	0.03	1.00
PA2122_at	3.08	2.95	3.06	3.27	-0.15	1.00
PA2123_at	2.41	2.51	2.55	2.59	-0.11	1.00
PA2125_at	4.10	3.63	3.88	3.75	0.05	1.00
PA2126_at	4.62	4.95	4.73	4.89	-0.03	1.00
PA2127_at	4.38	4.86	4.61	4.76	-0.07	1.00
PA2129_at	2.87	2.96	2.97	3.03	-0.09	1.00
PA2130_at	2.78	2.84	2.81	2.71	0.05	1.00
PA2131_at	3.67	3.75	3.83	3.44	0.07	1.00
PA2133_at	3.53	3.39	3.67	3.46	-0.10	1.00
PA2134_at	4.02	4.04	4.08	4.24	-0.13	1.00
PA2135_at	2.75	2.73	2.82	2.79	-0.06	1.00
PA2137_at	3.26	3.16	3.08	3.20	0.07	1.00
PA2138_at	3.03	3.03	3.13	3.02	-0.04	1.00
PA2139_r_at	2.17	2.20	2.18	2.05	0.07	1.00
PA2141_at	3.11	2.67	2.69	2.99	0.05	1.00
PA2144_glgP_at	2.98	2.58	2.87	2.76	-0.04	1.00
PA2145_at	4.54	4.55	4.66	4.59	-0.08	1.00
PA2147_katE_at	3.07	2.58	2.86	2.91	-0.06	1.00
PA2150_at	3.00	2.66	2.90	2.87	-0.05	1.00
PA2152_at	2.74	2.56	2.42	2.68	0.09	1.00
PA2153_glgB_at	4.76	4.19	4.26	4.46	0.11	1.00
PA2154_at	3.01	3.02	3.21	2.99	-0.09	1.00
PA2157_at	2.82	2.76	2.71	2.62	0.13	1.00
PA2158_at	2.93	2.92	2.85	2.81	0.10	1.00
PA2159_at	2.75	2.48	2.74	2.70	-0.11	1.00
PA2160_at	2.96	2.38	2.57	2.61	0.08	1.00

PA2162_at	3.11	2.70	3.14	2.95	-0.14	1.00
PA2164_at	3.62	3.10	3.33	3.14	0.12	1.00
PA2165_at	2.75	2.51	2.48	2.56	0.11	1.00
PA2167_at	2.90	2.58	2.57	2.69	0.10	1.00
PA2168_at	3.57	3.36	3.07	3.50	0.18	1.00
PA2169_at	5.36	4.75	5.18	5.42	-0.24	1.00
PA2170_at	3.24	2.77	3.29	3.09	-0.19	1.00
PA2174_at	3.87	3.60	3.89	3.83	-0.13	1.00
PA2176_at	3.14	3.63	3.15	3.41	0.11	1.00
PA2177_at	3.02	3.10	2.99	2.88	0.13	1.00
PA2178_at	3.77	3.37	3.50	3.70	-0.03	1.00
PA2180_at	3.27	3.02	3.02	3.16	0.06	1.00
PA2181_at	4.37	3.58	3.81	3.85	0.15	1.00
PA2182_at	2.58	2.47	2.71	2.39	-0.02	1.00
PA2183_at	2.56	2.40	2.37	2.35	0.12	1.00
PA2185_at	2.57	2.54	2.75	2.42	-0.03	1.00
PA2186_r_at	4.20	3.84	3.79	4.01	0.12	1.00
PA2187_at	2.57	2.49	2.35	2.49	0.11	1.00
PA2188_at	3.51	3.53	3.36	3.62	0.03	1.00
PA2189_at	3.49	3.14	3.53	3.29	-0.09	1.00
PA2190_at	4.22	3.70	3.58	3.98	0.19	1.00
PA2192_at	3.51	3.14	3.33	3.10	0.12	1.00
PA2199_at	6.52	6.44	6.64	6.49	-0.09	1.00
PA2201_at	2.55	2.48	2.33	2.60	0.05	1.00
PA2203_at	2.58	2.55	2.48	2.56	0.05	1.00
PA2206_at	2.99	2.80	2.85	2.91	0.02	1.00
PA2207_at	3.29	3.06	3.32	3.34	-0.15	1.00
PA2210_at	3.33	3.14	3.28	2.98	0.11	1.00
PA2211_at	2.48	2.21	2.17	2.39	0.07	1.00
PA2212_at	3.38	3.42	3.35	3.19	0.13	1.00
PA2213_at	2.35	2.40	2.41	2.42	-0.04	1.00
PA2214_at	2.78	2.69	2.78	2.77	-0.04	1.00
PA2215_at	2.35	2.47	2.57	2.36	-0.06	1.00
PA2217_at	2.71	2.44	2.62	2.44	0.05	1.00
PA2218_at	2.63	2.65	2.68	2.56	0.02	1.00
PA2219_opdE_at	3.56	3.50	3.78	3.47	-0.09	1.00
PA2220_at	3.52	3.59	3.45	3.52	0.07	1.00
PA2221_at	2.60	2.80	2.42	2.66	0.16	1.00
PA2222_at	2.63	2.48	2.76	2.58	-0.11	1.00
PA2223_at	2.44	2.58	2.40	2.40	0.11	1.00
PA2227_at	3.01	2.88	3.03	2.92	-0.03	1.00
PA2228_at	2.54	2.59	2.50	2.48	0.07	1.00
PA2229_at	4.38	4.16	4.17	4.10	0.14	1.00
PA2230_at	3.14	3.37	3.33	3.09	0.05	1.00

PA2231_at	4.38	4.33	4.38	4.49	-0.08	1.00
PA2233_at	2.25	2.22	2.26	2.37	-0.08	1.00
PA2235_at	4.16	4.22	4.43	4.18	-0.12	1.00
PA2236_at	3.20	3.31	3.53	3.18	-0.10	1.00
PA2237_at	3.11	2.84	3.03	3.26	-0.17	1.00
PA2238_at	4.11	4.06	4.22	4.00	-0.03	1.00
PA2239_at	2.78	2.65	2.74	2.60	0.05	1.00
PA2240_at	3.52	3.14	3.27	3.23	0.08	1.00
PA2243_at	3.07	2.93	3.04	2.66	0.15	1.00
PA2244_at	3.24	3.14	3.35	3.16	-0.06	1.00
PA2245_at	3.04	2.76	2.97	3.03	-0.10	1.00
PA2246_bkdR_at	3.98	3.69	3.77	3.63	0.14	1.00
PA2250_lpdV_at	5.35	5.42	5.33	5.58	-0.07	1.00
PA2251_at	2.69	2.72	2.73	2.79	-0.05	1.00
PA2255_pvcB_at	2.52	2.38	2.46	2.54	-0.05	1.00
PA2256_pvcC_at	2.95	2.48	2.70	2.45	0.14	1.00
PA2257_pvcD_at	3.15	2.66	2.87	2.83	0.06	1.00
PA2259_ptxS_at	4.48	4.52	4.28	4.52	0.10	1.00
PA2260_at	4.34	4.57	4.63	4.39	-0.05	1.00
PA2261_at	3.63	3.18	3.49	3.73	-0.21	1.00
PA2263_at	3.82	3.80	3.89	3.81	-0.04	1.00
PA2264_at	3.36	3.86	3.70	3.36	0.08	1.00
PA2266_at	4.75	4.57	4.53	4.91	-0.06	1.00
PA2267_at	4.17	4.33	3.98	4.34	0.09	1.00
PA2269_at	4.98	4.67	5.02	4.96	-0.16	1.00
PA2271_i_at	3.95	3.64	3.78	3.96	-0.07	1.00
PA2272_pbpC_at	3.63	3.78	3.74	3.89	-0.11	1.00
PA2273_at	4.84	4.91	4.75	4.83	0.09	1.00
PA2274_at	4.51	5.31	4.69	4.97	0.08	1.00
PA2276_at	2.89	2.92	3.17	2.89	-0.13	1.00
PA2280_at	4.34	3.62	4.03	3.74	0.09	1.00
PA2281_at	3.74	4.07	4.13	3.91	-0.11	1.00
PA2283_at	3.10	2.65	3.08	2.86	-0.10	1.00
PA2284_at	2.43	2.15	2.36	2.50	-0.14	1.00
PA2285_at	2.35	2.18	2.34	2.43	-0.12	1.00
PA2289_at	3.27	2.75	2.92	2.93	0.09	1.00
PA2292_at	3.23	2.95	3.12	3.26	-0.10	1.00
PA2293_at	3.02	2.82	2.88	2.75	0.10	1.00
PA2294_at	3.48	2.95	3.33	3.27	-0.09	1.00
PA2296_at	4.18	4.02	4.12	4.16	-0.04	1.00
PA2297_at	2.98	3.35	2.98	3.07	0.14	1.00
PA2301_at	4.13	3.76	4.10	3.85	-0.03	1.00
PA2302_at	5.00	4.38	4.88	4.97	-0.24	1.00
PA2303_at	2.94	2.92	2.85	2.93	0.04	1.00

PA2304_at	5.41	5.32	5.41	5.51	-0.10	1.00
PA2306_at	3.04	3.00	3.24	2.91	-0.05	1.00
PA2307_at	3.77	3.20	3.72	3.57	-0.16	1.00
PA2309_at	4.29	4.10	4.34	4.26	-0.10	1.00
PA2310_at	3.01	2.74	2.75	2.92	0.05	1.00
PA2312_at	3.35	2.90	3.07	2.86	0.16	1.00
PA2313_at	3.03	2.62	3.11	2.72	-0.08	1.00
PA2315_at	3.29	2.83	3.29	3.16	-0.17	1.00
PA2316_at	2.99	2.94	3.01	2.97	-0.03	1.00
PA2318_at	2.63	2.50	2.71	2.51	-0.05	1.00
PA2321_at	3.04	3.27	3.04	3.09	0.09	1.00
PA2324_at	2.40	2.25	2.35	2.36	-0.03	1.00
PA2325_at	2.82	2.73	2.82	2.93	-0.10	1.00
PA2327_at	4.86	4.36	4.74	4.35	0.07	1.00
PA2328_at	2.90	2.72	2.72	2.77	0.06	1.00
PA2332_at	4.84	4.61	4.69	4.59	0.09	1.00
PA2333_at	3.52	3.38	3.48	3.55	-0.06	1.00
PA2334_at	2.65	2.59	2.70	2.62	-0.04	1.00
PA2335_at	2.70	2.61	2.71	2.77	-0.08	1.00
PA2338_at	3.48	3.13	3.38	3.28	-0.02	1.00
PA2339_at	3.63	3.27	3.61	3.41	-0.06	1.00
PA2340_at	2.36	2.16	2.27	2.17	0.04	1.00
PA2341_at	2.81	2.60	2.72	2.76	-0.04	1.00
PA2343_mtlY_at	2.78	2.52	2.67	2.69	-0.03	1.00
PA2344_mtlZ_at	3.45	3.11	3.31	3.31	-0.03	1.00
PA2345_at	3.06	2.87	2.86	3.00	0.04	1.00
PA2346_at	2.82	2.55	2.57	2.57	0.11	1.00
PA2347_at	3.02	2.87	2.83	2.94	0.06	1.00
PA2348_at	2.62	2.76	2.68	2.89	-0.09	1.00
PA2349_at	2.90	2.66	2.85	2.91	-0.10	1.00
PA2350_at	3.52	3.11	3.50	3.27	-0.07	1.00
PA2351_at	2.65	2.35	2.34	2.58	0.04	1.00
PA2354_at	3.51	2.84	3.28	3.20	-0.07	1.00
PA2355_at	3.42	3.55	3.45	3.40	0.06	1.00
PA2357_msuE_at	2.35	2.17	2.24	2.31	-0.02	1.00
PA2358_at	2.46	2.29	2.17	2.35	0.12	1.00
PA2359_at	2.75	2.62	2.70	2.58	0.05	1.00
PA2360_at	4.16	4.15	4.24	4.25	-0.09	1.00
PA2361_at	3.14	2.63	2.69	2.76	0.16	1.00
PA2362_at	2.62	2.70	2.87	2.67	-0.11	1.00
PA2367_at	3.35	3.11	3.45	3.31	-0.15	1.00
PA2368_i_at	5.93	5.49	6.03	5.77	-0.19	1.00
PA2369_at	4.51	4.05	4.14	4.79	-0.18	1.00
PA2371_at	3.70	3.68	3.86	3.80	-0.14	1.00

PA2372_at	2.50	2.32	2.39	2.25	0.09	1.00
PA2373_at	3.96	3.85	3.92	4.01	-0.06	1.00
PA2375_i_at	3.28	3.13	3.08	3.16	0.08	1.00
PA2376_at	2.54	2.48	2.58	2.57	-0.06	1.00
PA2377_at	2.65	2.71	2.87	2.60	-0.06	1.00
PA2378_at	3.94	3.90	3.93	3.87	0.02	1.00
PA2380_at	3.17	3.16	3.25	2.88	0.10	1.00
PA2382_lldA_at	2.89	2.86	2.90	2.97	-0.06	1.00
PA2384_at	2.68	2.66	2.45	2.61	0.14	1.00
PA2385_at	3.24	3.01	2.95	3.16	0.07	1.00
PA2386_pvdA_at	2.97	2.44	2.93	2.59	-0.05	1.00
PA2387_at	2.15	2.22	2.18	2.15	0.02	1.00
PA2388_at	4.63	5.24	4.98	5.10	-0.11	1.00
PA2389_at	3.39	2.89	3.24	3.19	-0.08	1.00
PA2390_at	4.12	3.72	4.07	3.91	-0.07	1.00
PA2392_at	3.20	2.74	2.84	2.71	0.19	1.00
PA2393_at	2.68	2.22	2.73	2.50	-0.16	1.00
PA2394_at	2.59	2.50	2.59	2.72	-0.11	1.00
PA2397_pvdE_at	2.45	2.35	2.37	2.50	-0.04	1.00
PA2399_pvdD_at	4.43	4.20	4.21	4.21	0.10	1.00
PA2400_at	3.47	3.36	3.36	3.40	0.04	1.00
PA2401_at	2.90	2.68	2.69	2.72	0.08	1.00
PA2402_at	3.00	2.83	2.96	2.81	0.03	1.00
PA2404_at	3.11	2.64	3.15	3.01	-0.21	1.00
PA2405_at	3.18	2.67	3.02	3.02	-0.09	1.00
PA2406_at	2.54	2.48	2.59	2.51	-0.04	1.00
PA2407_at	3.28	3.33	3.15	3.36	0.04	1.00
PA2408_at	2.79	2.60	2.82	2.67	-0.05	1.00
PA2410_at	3.31	2.96	3.14	3.27	-0.07	1.00
PA2411_at	3.06	2.66	2.76	2.90	0.03	1.00
PA2412_at	2.34	2.46	2.54	2.32	-0.03	1.00
PA2414_at	4.02	4.05	4.30	3.97	-0.10	1.00
PA2415_at	4.32	4.24	4.32	4.43	-0.10	1.00
PA2416_treA_at	3.15	3.01	3.17	3.04	-0.03	1.00
PA2419_at	2.39	2.28	2.36	2.35	-0.02	1.00
PA2426_pvdS_at	2.46	2.37	2.33	2.35	0.07	1.00
PA2427_at	2.75	2.55	2.79	2.44	0.04	1.00
PA2429_at	2.66	2.69	2.69	2.49	0.09	1.00
PA2430_at	3.45	3.18	3.53	3.37	-0.13	1.00
PA2431_at	3.20	2.87	3.19	2.64	0.12	1.00
PA2433_at	4.76	4.71	4.75	4.89	-0.09	1.00
PA2438_at	3.47	3.31	3.25	3.33	0.10	1.00
PA2439_at	2.77	2.52	2.57	2.54	0.09	1.00
PA2440_at	3.80	3.66	3.86	3.81	-0.11	1.00

PA2442_gcvT2_at	3.32	3.32	3.49	3.42	-0.13	1.00
PA2443_sdaA_at	3.11	3.37	3.36	3.16	-0.02	1.00
PA2444_glyA2_at	2.27	2.07	1.96	2.30	0.04	1.00
PA2445_gcvP2_at	3.24	3.44	3.31	3.50	-0.06	1.00
PA2446_gcvH2_at	3.68	4.56	3.64	4.13	0.24	1.00
PA2448_at	2.77	2.76	2.84	2.82	-0.07	1.00
PA2449_at	2.99	2.94	2.84	2.93	0.08	1.00
PA2450_at	3.47	3.49	3.45	3.31	0.10	1.00
PA2452_at	3.68	3.17	3.37	3.15	0.17	1.00
PA2453_at	4.40	5.52	5.25	5.09	-0.21	1.00
PA2454_at	3.69	3.59	3.68	3.70	-0.05	1.00
PA2455_at	3.95	3.44	3.92	3.66	-0.10	1.00
PA2457_at	3.83	3.59	3.25	3.85	0.16	1.00
PA2458_at	2.85	2.72	2.80	2.70	0.03	1.00
PA2459_at	2.72	2.83	2.70	2.66	0.10	1.00
PA2460_i_at	4.79	5.43	4.90	4.95	0.18	1.00
PA2462_at	3.16	3.03	3.02	3.11	0.02	1.00
PA2464_at	3.51	3.36	3.27	3.31	0.14	1.00
PA2465_at	2.84	2.80	2.92	2.76	-0.02	1.00
PA2466_at	2.92	2.65	2.99	2.80	-0.11	1.00
PA2468_at	3.59	3.04	3.42	3.36	-0.08	1.00
PA2469_at	3.72	3.70	3.68	3.49	0.12	1.00
PA2470_gtdA_at	4.18	3.97	4.23	4.00	-0.04	1.00
PA2474_at	3.27	2.95	3.12	3.19	-0.05	1.00
PA2475_at	3.03	2.89	3.22	2.98	-0.14	1.00
PA2478_at	2.92	2.74	2.82	2.69	0.07	1.00
PA2479_at	3.16	3.15	3.02	3.12	0.09	1.00
PA2480_at	2.97	2.73	2.78	2.84	0.04	1.00
PA2481_at	3.19	3.26	3.19	3.12	0.07	1.00
PA2484_at	3.15	3.31	3.27	3.29	-0.05	1.00
PA2485_at	4.95	6.08	5.32	5.54	0.09	1.00
PA2490_at	3.35	3.25	3.24	3.25	0.05	1.00
PA2491_at	2.80	2.92	2.83	3.22	-0.16	1.00
PA2492_mexT_at	3.91	4.08	4.02	3.87	0.05	1.00
PA2493_mexE_at	3.17	2.73	2.82	2.89	0.10	1.00
PA2494_mexF_at	2.67	2.68	2.70	2.88	-0.12	1.00
PA2495_oprN_at	2.92	2.95	2.89	2.92	0.03	1.00
PA2496_at	2.59	2.44	2.45	2.39	0.09	1.00
PA2497_at	4.25	4.40	4.50	4.31	-0.08	1.00
PA2498_at	2.53	2.25	2.31	2.35	0.06	1.00
PA2499_at	5.07	4.27	5.08	4.44	-0.09	1.00
PA2500_at	2.91	2.78	2.95	3.01	-0.13	1.00
PA2503_at	3.40	3.45	3.50	3.49	-0.07	1.00
PA2508_catC_at	3.48	3.05	3.32	3.30	-0.05	1.00

PA2510_catR_at	4.77	4.19	4.28	4.44	0.12	1.00
PA2511_at	3.84	3.68	3.63	3.61	0.14	1.00
PA2512_antA_at	4.59	4.14	4.41	4.38	-0.03	1.00
PA2513_antB_at	2.71	2.47	2.60	2.68	-0.05	1.00
PA2514_antC_at	2.48	2.19	2.19	2.22	0.13	1.00
PA2515_xylL_at	3.32	2.79	3.27	3.02	-0.09	1.00
PA2516_xylZ_at	3.39	2.98	3.24	3.56	-0.21	1.00
PA2518_xylX_at	2.82	2.70	2.67	2.72	0.06	1.00
PA2522_czcC_at	3.05	3.03	3.10	2.83	0.07	1.00
PA2524_at	4.17	3.90	4.02	3.90	0.07	1.00
PA2526_at	2.82	2.86	2.79	2.82	0.04	1.00
PA2528_at	3.85	4.00	3.89	3.70	0.12	1.00
PA2530_at	3.24	3.32	3.29	3.50	-0.12	1.00
PA2531_at	3.44	3.30	3.46	3.41	-0.07	1.00
PA2532_tpx_at	3.80	4.11	3.92	3.78	0.11	1.00
PA2533_at	3.96	3.67	3.82	4.07	-0.13	1.00
PA2534_at	3.01	2.77	2.90	3.08	-0.10	1.00
PA2535_at	3.09	3.14	3.03	3.26	-0.03	1.00
PA2536_at	4.02	4.00	3.90	3.92	0.10	1.00
PA2537_at	3.04	3.03	3.05	2.97	0.02	1.00
PA2539_at	2.71	2.74	2.72	2.68	0.03	1.00
PA2541_at	4.58	4.65	4.62	4.76	-0.08	1.00
PA2543_at	2.77	2.62	2.76	2.76	-0.07	1.00
PA2544_at	3.52	3.16	3.38	3.40	-0.05	1.00
PA2545_xthA_at	4.55	4.37	4.36	4.37	0.10	1.00
PA2546_at	4.00	3.78	3.94	3.71	0.07	1.00
PA2547_at	2.52	2.38	2.47	2.31	0.06	1.00
PA2548_at	4.11	3.71	4.10	3.83	-0.05	1.00
PA2549_at	4.43	4.15	4.16	4.15	0.13	1.00
PA2551_at	4.77	5.02	4.88	4.95	-0.02	1.00
PA2554_at	4.70	4.34	4.82	4.58	-0.18	1.00
PA2555_at	3.59	3.81	3.81	3.79	-0.10	1.00
PA2557_at	2.42	2.41	2.43	2.25	0.07	1.00
PA2558_at	3.92	3.62	3.86	3.80	-0.06	1.00
PA2559_at	5.07	5.07	5.02	5.02	0.05	1.00
PA2567_at	4.00	4.26	4.08	4.35	-0.09	1.00
PA2574_at	3.98	3.83	3.83	3.83	0.08	1.00
PA2575_at	4.86	5.17	5.18	5.10	-0.12	1.00
PA2576_at	4.61	4.42	4.51	4.46	0.03	1.00
PA2577_at	5.07	5.02	4.89	5.10	0.05	1.00
PA2578_at	2.68	2.42	2.38	2.50	0.11	1.00
PA2579_at	2.76	2.80	2.80	2.82	-0.03	1.00
PA2581_at	3.18	3.02	3.08	3.00	0.06	1.00
PA2585_uvrC_at	3.05	3.01	3.21	3.07	-0.11	1.00

PA2586_gacA_at	5.89	6.06	5.97	6.13	-0.07	1.00
PA2587_at	3.01	2.81	2.87	3.16	-0.10	1.00
PA2589_at	3.35	3.14	3.15	3.47	-0.07	1.00
PA2591_at	3.61	3.49	3.57	3.61	-0.04	1.00
PA2592_at	2.94	3.32	3.26	3.23	-0.12	1.00
PA2593_at	3.66	3.51	3.40	3.63	0.07	1.00
PA2595_at	3.22	2.85	3.24	3.13	-0.15	1.00
PA2596_at	2.79	2.73	2.85	2.83	-0.08	1.00
PA2599_at	4.50	4.23	4.23	4.25	0.13	1.00
PA2602_at	3.34	3.02	3.09	3.09	0.09	1.00
PA2604_at	6.04	6.91	6.30	6.39	0.13	1.00
PA2605_at	3.94	4.25	3.95	4.07	0.09	1.00
PA2606_at	4.52	4.75	4.56	4.80	-0.04	1.00
PA2607_at	2.34	2.50	2.47	2.31	0.03	1.00
PA2608_at	3.42	3.27	3.40	3.54	-0.12	1.00
PA2610_at	3.23	3.07	2.99	3.16	0.07	1.00
PA2611_cysG_at	3.49	3.64	3.67	3.67	-0.10	1.00
PA2612_serS_at	5.24	6.02	5.61	5.78	-0.06	1.00
PA2613_at	5.08	5.07	5.07	4.91	0.08	1.00
PA2614_lolA_at	4.14	4.57	4.27	4.11	0.17	1.00
PA2615_ftsK_at	4.98	5.31	5.02	4.94	0.16	1.00
PA2616_trxB1_at	2.73	2.92	2.89	2.65	0.06	1.00
PA2617_aat_at	2.87	2.88	2.98	2.87	-0.05	1.00
PA2620_clpA_at	6.41	6.81	6.57	6.83	-0.09	1.00
PA2622_cspD_at	8.17	8.40	8.33	8.47	-0.11	1.00
PA2626_trmU_at	5.36	5.72	5.23	5.52	0.17	1.00
PA2627_at	3.21	3.66	3.48	3.33	0.03	1.00
PA2629_purB_at	4.45	5.12	4.87	4.83	-0.07	1.00
PA2630_at	2.84	3.15	2.97	2.94	0.04	1.00
PA2631_at	4.40	5.24	5.12	5.05	-0.27	1.00
PA2633_at	3.32	3.35	3.29	3.42	-0.02	1.00
PA2634_at	3.58	4.26	3.69	3.74	0.20	1.00
PA2635_at	3.50	3.14	3.43	3.36	-0.08	1.00
PA2636_at	2.38	2.26	2.41	2.43	-0.10	1.00
PA2637_nuoA_at	5.71	6.06	6.12	6.00	-0.18	1.00
PA2638_nuoB_at	5.76	6.31	6.11	6.37	-0.20	1.00
PA2639_nuoD_at	2.89	2.97	2.95	3.02	-0.06	1.00
PA2652_at	3.35	3.59	3.45	3.39	0.05	1.00
PA2655_i_at	4.83	4.92	5.13	4.86	-0.12	1.00
PA2658_at	5.88	5.59	5.70	5.45	0.16	1.00
PA2661_at	3.71	3.69	3.61	3.72	0.04	1.00
PA2662_at	4.73	5.35	4.89	4.82	0.19	1.00
PA2663_at	4.95	5.39	5.19	5.07	0.04	1.00
PA2668_at	2.74	2.55	2.73	2.61	-0.03	1.00

PA2669_at	2.87	2.50	2.83	2.77	-0.12	1.00
PA2670_at	2.91	2.46	2.56	2.73	0.04	1.00
PA2672_at	3.81	2.93	3.38	3.63	-0.14	1.00
PA2673_at	3.29	3.32	3.40	3.05	0.09	1.00
PA2674_at	3.50	2.74	3.05	2.97	0.11	1.00
PA2675_at	4.19	3.29	3.73	3.64	0.06	1.00
PA2676_at	4.04	3.54	3.97	3.50	0.06	1.00
PA2679_at	3.19	3.08	3.10	3.14	0.02	1.00
PA2680_at	2.84	2.64	2.77	2.83	-0.06	1.00
PA2681_at	3.34	2.96	3.17	2.93	0.10	1.00
PA2683_at	2.95	3.01	2.83	3.18	-0.02	1.00
PA2685_at	3.11	2.93	3.01	2.89	0.07	1.00
PA2688_pfeA_at	2.50	2.31	2.57	2.44	-0.10	1.00
PA2691_at	4.21	3.52	4.17	3.82	-0.13	1.00
PA2692_at	2.92	3.38	3.10	3.05	0.08	1.00
PA2693_at	2.94	2.65	2.76	2.65	0.09	1.00
PA2694_at	4.63	3.98	4.06	4.08	0.24	1.00
PA2696_at	3.40	2.98	3.28	3.42	-0.16	1.00
PA2699_at	2.39	2.22	2.45	2.30	-0.07	1.00
PA2700_at	3.15	3.06	3.07	2.84	0.15	1.00
PA2701_at	3.22	2.88	3.06	2.85	0.10	1.00
PA2704_at	3.35	3.08	2.91	3.24	0.14	1.00
PA2705_at	3.63	3.59	3.50	3.53	0.10	1.00
PA2707_at	3.29	3.50	3.40	3.04	0.17	1.00
PA2708_at	2.74	2.80	2.89	2.69	-0.02	1.00
PA2709_cysK_at	4.16	4.53	4.30	4.60	-0.10	1.00
PA2710_at	3.37	3.66	3.45	3.68	-0.05	1.00
PA2711_at	5.03	4.41	4.49	4.66	0.15	1.00
PA2712_at	4.67	4.37	4.72	4.38	-0.03	1.00
PA2713_at	2.94	2.66	2.86	2.80	-0.03	1.00
PA2716_at	4.02	4.17	4.16	4.07	-0.02	1.00
PA2717_cpo_at	3.51	3.44	3.48	3.54	-0.03	1.00
PA2718_at	2.93	2.97	2.97	3.03	-0.05	1.00
PA2720_at	3.14	2.75	3.23	2.97	-0.16	1.00
PA2721_at	2.36	2.10	2.41	2.17	-0.06	1.00
PA2722_at	2.94	2.76	2.72	3.07	-0.04	1.00
PA2724_r_at	4.31	3.99	4.07	3.97	0.13	1.00
PA2725_at	3.73	3.53	3.69	3.75	-0.09	1.00
PA2726_at	3.29	3.22	3.28	3.06	0.08	1.00
PA2727_at	4.32	4.22	4.14	4.16	0.12	1.00
PA2728_at	4.44	3.86	4.05	4.11	0.07	1.00
PA2730_at	3.66	3.85	3.74	3.67	0.05	1.00
PA2731_at	3.28	3.29	3.13	3.25	0.10	1.00
PA2734_at	3.26	3.14	3.12	3.14	0.07	1.00

PA2736_at	4.76	5.35	5.15	5.32	-0.18	1.00
PA2737_at	6.73	7.03	6.80	6.90	0.03	1.00
PA2741_rplT_at	5.21	6.05	5.52	5.67	0.04	1.00
PA2743_infC_at	6.84	7.99	7.29	7.29	0.13	1.00
PA2745_at	4.38	4.55	4.57	4.43	-0.04	1.00
PA2746_at	2.28	2.54	2.39	2.56	-0.07	1.00
PA2749_endA_at	4.19	3.89	4.06	3.69	0.17	1.00
PA2750_at	3.33	3.15	3.25	3.18	0.02	1.00
PA2751_at	3.01	2.84	2.99	2.93	-0.03	1.00
PA2752_at	3.58	3.15	3.32	3.05	0.18	1.00
PA2754_at	5.85	6.60	6.30	6.45	-0.15	1.00
PA2758_at	3.02	3.24	3.43	3.14	-0.15	1.00
PA2761_at	2.90	3.10	2.92	3.05	0.02	1.00
PA2764_at	3.11	2.88	2.93	3.17	-0.05	1.00
PA2765_at	5.62	5.85	5.83	5.82	-0.09	1.00
PA2766_at	4.65	4.21	4.22	4.37	0.14	1.00
PA2767_at	3.21	2.71	2.89	2.82	0.10	1.00
PA2769_at	3.10	3.17	3.18	3.15	-0.03	1.00
PA2770_at	4.24	4.69	3.99	4.84	0.05	1.00
PA2771_at	2.66	2.71	2.85	2.78	-0.13	1.00
PA2772_at	2.49	2.78	2.79	2.58	-0.05	1.00
PA2773_at	2.86	2.59	2.83	2.90	-0.14	1.00
PA2777_at	4.20	4.63	4.37	4.30	0.08	1.00
PA2779_at	4.66	4.95	4.66	5.22	-0.14	1.00
PA2780_at	5.21	5.83	5.53	5.62	-0.06	1.00
PA2785_at	3.38	3.06	3.46	3.29	-0.15	1.00
PA2786_at	3.24	2.84	2.83	3.08	0.08	1.00
PA2787_cpg2_at	2.89	2.98	3.09	2.92	-0.07	1.00
PA2788_at	4.25	4.17	4.42	4.25	-0.12	1.00
PA2790_at	3.77	3.33	3.53	3.82	-0.13	1.00
PA2791_at	3.17	3.19	3.40	3.14	-0.09	1.00
PA2794_at	4.21	3.68	3.99	4.01	-0.05	1.00
PA2796_tal_at	3.58	3.53	3.62	3.45	0.02	1.00
PA2801_at	4.50	4.46	4.32	4.46	0.09	1.00
PA2802_at	3.07	3.14	3.12	2.98	0.05	1.00
PA2803_at	3.58	3.25	3.31	3.39	0.06	1.00
PA2804_at	3.44	3.28	3.24	3.26	0.11	1.00
PA2805_at	5.47	5.75	5.26	5.75	0.11	1.00
PA2806_at	2.92	2.72	2.83	2.64	0.09	1.00
PA2809_at	3.32	3.00	3.12	3.06	0.07	1.00
PA2810_at	3.03	2.84	3.02	2.97	-0.06	1.00
PA2811_at	2.65	2.78	2.93	2.79	-0.15	1.00
PA2812_at	4.39	4.44	4.20	4.37	0.13	1.00
PA2813_at	4.27	4.17	4.20	4.07	0.09	1.00

PA2814_at	3.29	3.16	3.17	3.06	0.11	1.00
PA2815_at	4.05	3.93	3.73	3.96	0.15	1.00
PA2817_at	3.99	3.83	3.48	3.97	0.18	1.00
PA2818_at	3.90	3.64	3.73	3.67	0.07	1.00
PA2819_at	3.51	2.82	3.19	3.25	-0.06	1.00
PA2821_at	2.95	2.85	2.81	2.93	0.03	1.00
PA2823_at	4.64	4.59	4.66	4.41	0.08	1.00
PA2824_at	3.82	3.74	3.72	3.73	0.06	1.00
PA2825_at	4.97	5.22	5.14	5.21	-0.08	1.00
PA2826_at	8.04	8.36	8.10	8.10	0.10	1.00
PA2827_at	5.47	5.63	5.31	5.74	0.03	1.00
PA2828_at	4.77	4.43	4.47	4.43	0.15	1.00
PA2829_at	2.40	2.29	2.43	2.45	-0.10	1.00
PA2831_at	3.55	3.35	3.37	3.44	0.04	1.00
PA2832_tpm_at	2.79	2.56	2.73	2.75	-0.07	1.00
PA2833_i_at	2.57	2.52	2.77	2.61	-0.15	1.00
PA2834_at	3.07	3.00	2.91	3.08	0.05	1.00
PA2836_at	2.73	2.53	2.57	2.64	0.03	1.00
PA2839_at	2.44	2.46	2.54	2.52	-0.08	1.00
PA2841_at	5.05	5.71	5.78	5.50	-0.25	1.00
PA2842_at	3.80	3.25	3.42	3.90	-0.14	1.00
PA2843_at	5.22	4.91	5.10	5.08	-0.03	1.00
PA2845_at	2.46	2.25	2.29	2.51	-0.04	1.00
PA2849_at	3.85	4.01	3.75	3.82	0.15	1.00
PA2852_at	2.98	2.91	2.87	2.89	0.07	1.00
PA2854_at	4.88	5.12	4.94	5.11	-0.02	1.00
PA2860_at	3.53	3.86	3.60	3.75	0.02	1.00
PA2861_ligT_at	3.31	2.96	3.24	3.30	-0.14	1.00
PA2863_lipH_at	2.77	2.37	2.49	2.34	0.16	1.00
PA2864_at	2.74	2.62	2.91	2.69	-0.12	1.00
PA2865_at	2.99	3.20	3.10	2.82	0.13	1.00
PA2869_at	3.45	3.50	3.29	3.45	0.11	1.00
PA2870_at	4.76	4.91	4.72	4.81	0.07	1.00
PA2871_at	3.35	3.31	3.10	3.46	0.05	1.00
PA2872_at	4.79	4.46	4.86	4.72	-0.17	1.00
PA2874_at	3.47	3.75	3.87	3.56	-0.10	1.00
PA2876_pyrF_at	4.56	4.12	4.21	4.28	0.09	1.00
PA2877_at	3.50	3.48	3.57	3.57	-0.08	1.00
PA2879_at	4.89	5.05	4.94	4.79	0.10	1.00
PA2881_at	3.24	3.03	3.19	3.22	-0.07	1.00
PA2882_at	3.13	2.82	2.93	2.81	0.10	1.00
PA2888_at	2.53	2.42	2.62	2.52	-0.09	1.00
PA2889_at	2.47	2.43	2.35	2.43	0.06	1.00
PA2891_at	3.82	3.15	3.65	3.55	-0.12	1.00

PA2892_at	4.45	4.12	4.09	4.22	0.13	1.00
PA2893_at	2.99	2.92	2.98	3.06	-0.07	1.00
PA2894_at	4.93	5.12	5.19	5.12	-0.13	1.00
PA2897_at	5.97	5.72	5.56	5.87	0.13	1.00
PA2898_at	3.68	3.71	3.89	3.58	-0.04	1.00
PA2899_at	3.89	4.12	3.59	4.33	0.04	1.00
PA2900_at	2.74	2.81	2.93	2.70	-0.04	1.00
PA2901_at	5.48	5.85	5.43	5.56	0.17	1.00
PA2902_at	2.96	2.70	2.66	2.70	0.15	1.00
PA2903_cobJ_at	3.45	3.11	3.29	3.18	0.05	1.00
PA2904_cobI_at	3.54	3.42	3.52	3.48	-0.02	1.00
PA2905_cobH_at	3.69	3.29	3.59	3.55	-0.08	1.00
PA2906_at	3.62	4.26	3.82	4.14	-0.04	1.00
PA2907_cobL_at	3.69	3.56	3.77	3.65	-0.08	1.00
PA2908_cbiD_at	2.92	3.08	2.97	2.85	0.09	1.00
PA2910_at	3.95	3.35	3.50	3.56	0.12	1.00
PA2912_at	3.70	3.12	3.48	3.41	-0.04	1.00
PA2913_at	2.62	2.41	2.64	2.58	-0.09	1.00
PA2914_at	3.32	3.00	3.23	2.95	0.07	1.00
PA2917_at	2.99	2.99	2.87	2.85	0.13	1.00
PA2918_at	2.70	2.63	2.74	2.77	-0.09	1.00
PA2921_at	2.91	3.15	3.22	2.67	0.08	1.00
PA2922_at	2.64	2.59	2.59	2.72	-0.04	1.00
PA2923_hisJ_at	2.95	2.89	3.05	3.02	-0.11	1.00
PA2924_hisQ_at	2.43	2.40	2.22	2.35	0.14	1.00
PA2927_at	2.81	2.62	2.57	2.60	0.13	1.00
PA2930_at	3.32	3.42	3.34	3.28	0.06	1.00
PA2932_morB_at	2.98	4.98	3.66	3.59	0.36	1.00
PA2933_at	4.30	4.05	4.09	4.07	0.10	1.00
PA2934_at	3.10	2.83	2.92	2.66	0.18	1.00
PA2936_at	2.93	2.54	2.91	2.80	-0.13	1.00
PA2938_at	4.08	3.71	4.00	3.70	0.04	1.00
PA2940_at	3.63	3.48	3.50	3.55	0.04	1.00
PA2941_at	3.20	3.11	3.02	3.09	0.10	1.00
PA2942_at	4.64	4.71	4.52	4.58	0.12	1.00
PA2943_at	3.25	3.16	2.93	3.23	0.12	1.00
PA2944_cobN_at	4.33	4.20	4.35	4.29	-0.05	1.00
PA2946_at	5.55	5.71	5.69	5.51	0.03	1.00
PA2947_i_at	3.64	3.33	3.57	3.28	0.06	1.00
PA2948_cobM_at	2.67	2.51	2.64	2.60	-0.03	1.00
PA2949_at	2.70	2.51	2.65	2.50	0.03	1.00
PA2950_at	4.12	4.28	3.84	4.45	0.05	1.00
PA2951_etfA_at	6.21	6.95	6.69	6.68	-0.11	1.00
PA2952_etfB_at	7.80	8.49	8.01	8.19	0.05	1.00

PA2954_at	3.19	2.98	2.78	3.12	0.14	1.00
PA2956_at	3.24	3.35	3.22	3.27	0.05	1.00
PA2960_pilZ_at	4.41	4.43	4.32	4.35	0.09	1.00
PA2961_holB_at	3.91	4.24	3.99	3.86	0.15	1.00
PA2962_tmk_at	6.18	6.48	6.07	6.39	0.10	1.00
PA2963_at	5.68	5.22	5.41	5.31	0.09	1.00
PA2964_pabC_at	4.36	4.42	4.36	4.34	0.04	1.00
PA2965_fabF1_at	5.08	4.95	5.03	5.15	-0.08	1.00
PA2967_fabG_at	5.74	6.55	6.49	6.34	-0.27	1.00
PA2969_plsX_at	2.84	2.94	2.83	2.68	0.14	1.00
PA2970_rpmF_at	3.76	5.24	4.68	4.93	-0.30	1.00
PA2971_at	5.93	6.59	5.92	6.18	0.21	1.00
PA2973_at	5.63	5.86	5.65	5.55	0.14	1.00
PA2976_rne_at	6.85	6.90	6.63	6.88	0.12	1.00
PA2978_ptpA_at	3.57	3.40	3.54	3.50	-0.04	1.00
PA2980_at	3.40	4.02	3.60	3.75	0.03	1.00
PA2982_at	4.44	4.37	4.47	4.58	-0.12	1.00
PA2984_at	2.96	2.79	2.92	2.70	0.06	1.00
PA2987_at	4.76	5.17	4.64	5.14	0.08	1.00
PA2988_at	2.81	3.13	3.04	3.06	-0.08	1.00
PA2991_sth_at	3.20	3.42	3.43	3.45	-0.13	1.00
PA2992_at	5.60	5.42	5.62	5.52	-0.06	1.00
PA2994_nqrF_at	3.59	3.30	3.62	3.15	0.06	1.00
PA2995_nqrE_at	7.48	7.10	7.31	7.33	-0.03	1.00
PA2998_nqrB_at	5.30	4.99	5.09	5.45	-0.12	1.00
PA3000_aroP1_at	2.40	2.24	2.40	2.33	-0.04	1.00
PA3001_at	4.96	5.52	5.26	5.45	-0.12	1.00
PA3002_mfd_at	3.99	4.03	3.95	3.99	0.04	1.00
PA3003_at	2.48	2.56	2.57	2.66	-0.09	1.00
PA3009_at	4.26	4.84	4.69	4.64	-0.12	1.00
PA3011_topA_at	4.73	4.67	4.51	4.67	0.12	1.00
PA3012_at	3.14	3.09	3.07	3.28	-0.06	1.00
PA3013_foaB_at	4.07	4.22	3.83	4.28	0.08	1.00
PA3017_at	5.42	5.73	5.58	5.86	-0.15	1.00
PA3018_at	3.76	4.00	4.20	3.90	-0.17	1.00
PA3019_at	2.97	3.19	3.12	3.09	-0.02	1.00
PA3020_at	4.05	3.74	3.65	3.85	0.14	1.00
PA3022_at	5.47	5.81	5.71	5.67	-0.05	1.00
PA3024_at	2.73	2.94	2.87	2.91	-0.05	1.00
PA3029_moaB2_at	2.91	2.97	2.98	2.66	0.11	1.00
PA3030_at	3.25	3.28	3.24	3.14	0.08	1.00
PA3032_at	3.04	2.72	2.95	2.92	-0.06	1.00
PA3033_at	3.97	4.35	4.25	4.15	-0.04	1.00
PA3034_at	2.64	2.86	2.69	2.56	0.13	1.00

PA3037_at	3.06	2.66	3.05	2.74	-0.04	1.00
PA3039_at	2.88	2.58	2.86	2.68	-0.04	1.00
PA3041_at	5.38	5.49	5.28	5.42	0.09	1.00
PA3042_at	5.36	5.95	5.45	5.41	0.22	1.00
PA3045_at	4.53	4.52	4.55	4.18	0.16	1.00
PA3046_at	5.17	5.03	5.07	5.03	0.05	1.00
PA3047_at	3.83	4.26	3.84	3.99	0.13	1.00
PA3048_at	3.66	3.69	3.57	3.58	0.10	1.00
PA3049_rmf_at	6.32	7.27	7.00	7.25	-0.33	1.00
PA3051_at	3.53	3.50	3.55	3.44	0.02	1.00
PA3052_at	4.55	4.43	4.40	4.52	0.03	1.00
PA3057_at	2.45	2.23	2.36	2.28	0.02	1.00
PA3058_at	4.22	3.92	4.17	4.09	-0.06	1.00
PA3059_at	3.55	3.36	3.56	3.27	0.04	1.00
PA3060_at	4.54	4.19	4.82	4.30	-0.19	1.00
PA3061_at	2.75	2.73	2.95	2.80	-0.14	1.00
PA3062_at	3.46	3.31	3.22	3.30	0.12	1.00
PA3063_at	2.97	2.81	2.99	2.86	-0.04	1.00
PA3064_at	3.22	2.95	3.04	3.21	-0.04	1.00
PA3065_at	2.97	2.82	3.08	2.97	-0.13	1.00
PA3066_at	3.19	2.80	3.16	3.13	-0.15	1.00
PA3067_at	5.07	5.06	4.90	5.07	0.08	1.00
PA3068_at	5.70	5.28	5.69	5.45	-0.08	1.00
PA3070_at	4.30	4.95	4.41	4.69	0.07	1.00
PA3071_at	3.21	3.42	3.25	3.14	0.12	1.00
PA3074_at	2.70	2.71	2.84	2.71	-0.07	1.00
PA3075_at	3.49	3.25	3.43	3.44	-0.07	1.00
PA3078_at	2.87	2.57	2.52	2.68	0.12	1.00
PA3079_at	2.55	2.59	2.65	2.75	-0.13	1.00
PA3080_at	3.48	3.61	3.64	3.53	-0.04	1.00
PA3082_at	3.57	3.54	3.63	3.17	0.16	1.00
PA3083_pepN_at	2.20	2.36	2.32	2.55	-0.16	1.00
PA3084_at	3.57	3.71	3.66	3.77	-0.08	1.00
PA3085_at	4.50	4.44	4.38	4.36	0.10	1.00
PA3086_at	3.50	3.14	3.35	3.08	0.10	1.00
PA3087_at	2.80	2.95	3.05	2.82	-0.06	1.00
PA3088_at	3.44	3.37	3.50	3.42	-0.06	1.00
PA3089_at	3.29	3.21	3.38	3.27	-0.08	1.00
PA3091_at	4.71	4.65	4.49	5.00	-0.07	1.00
PA3092_fadH1_at	3.15	2.92	3.03	2.82	0.11	1.00
PA3093_at	4.58	4.19	4.21	4.36	0.10	1.00
PA3094_at	4.03	3.61	3.61	3.67	0.18	1.00
PA3095_xcpZ_at	3.56	3.23	3.40	3.54	-0.08	1.00
PA3096_xcpY_at	4.53	4.03	4.04	4.22	0.15	1.00

PA3097_xcpX_at	3.09	3.06	3.16	3.10	-0.06	1.00
PA3098_xcpW_at	3.32	3.33	3.42	3.29	-0.03	1.00
PA3099_xcpV_at	4.10	3.95	4.17	4.05	-0.09	1.00
PA3102_xcpS_at	3.81	3.73	3.83	3.83	-0.06	1.00
PA3104_xcpP_at	3.33	3.59	3.38	3.46	0.04	1.00
PA3107_metZ_at	4.10	4.85	4.18	4.38	0.20	1.00
PA3108_purF_at	3.52	4.14	3.63	3.65	0.19	1.00
PA3111_folC_at	4.40	4.89	4.40	4.49	0.20	1.00
PA3112_accD_at	4.78	4.88	4.81	4.77	0.04	1.00
PA3113_trpF_at	6.24	6.73	6.43	6.45	0.05	1.00
PA3114_truA_at	3.78	3.92	4.00	3.76	-0.03	1.00
PA3115_at	6.28	6.68	6.64	6.66	-0.17	1.00
PA3116_at	3.97	4.06	3.88	3.87	0.14	1.00
PA3119_at	4.70	3.96	4.54	4.62	-0.25	1.00
PA3121_leuC_at	3.37	3.02	3.12	3.42	-0.08	1.00
PA3122_at	3.21	3.07	3.15	2.98	0.07	1.00
PA3123_at	3.36	3.74	3.33	3.57	0.11	1.00
PA3124_at	4.66	4.87	4.67	4.60	0.13	1.00
PA3125_at	3.39	3.11	3.28	3.43	-0.11	1.00
PA3127_at	3.80	3.43	3.60	3.46	0.08	1.00
PA3128_at	3.50	3.65	3.69	3.51	-0.02	1.00
PA3130_at	3.43	3.34	3.31	3.35	0.06	1.00
PA3131_at	5.31	5.24	5.40	5.22	-0.04	1.00
PA3132_at	4.29	4.35	4.20	4.27	0.09	1.00
PA3133_at	5.03	4.86	5.26	4.93	-0.15	1.00
PA3134_gltX_at	5.24	5.17	5.22	5.15	0.02	1.00
PA3135_at	2.68	2.94	2.73	2.79	0.05	1.00
PA3136_at	2.62	2.39	2.43	2.37	0.11	1.00
PA3137_at	3.51	3.05	3.15	3.02	0.19	1.00
PA3138_uvrB_at	4.36	4.55	4.36	4.46	0.04	1.00
PA3139_at	5.86	6.37	6.15	6.21	-0.06	1.00
PA3140_at	2.73	2.66	2.69	2.52	0.09	1.00
PA3142_i_at	4.03	4.43	4.12	4.73	-0.20	1.00
PA3145_wbpL_at	3.60	3.47	3.46	3.42	0.09	1.00
PA3148_wbpI_at	5.36	5.38	5.32	5.31	0.05	1.00
PA3150_wbpG_at	2.46	2.57	2.54	2.34	0.08	1.00
PA3151_hisF2_at	4.42	4.50	4.40	4.22	0.15	1.00
PA3153_wzx_at	2.49	2.62	2.61	2.66	-0.08	1.00
PA3155_wbpE_at	3.16	3.44	3.16	3.16	0.14	1.00
PA3156_wbpD_at	3.58	3.75	3.77	3.49	0.04	1.00
PA3157_at	3.19	3.04	3.25	3.08	-0.05	1.00
PA3161_himD_at	5.93	6.48	6.16	6.39	-0.07	1.00
PA3162_rpsA_at	6.09	6.34	6.14	6.23	0.03	1.00
PA3163_cmk_at	4.08	4.06	4.26	4.06	-0.09	1.00

PA3165_hisC2_at	3.10	3.27	2.96	3.15	0.13	1.00
PA3167_serC_at	5.08	5.10	4.92	5.06	0.10	1.00
PA3168_gyrA_at	4.87	4.92	4.94	5.00	-0.07	1.00
PA3170_at	3.31	3.25	3.14	3.21	0.11	1.00
PA3171_ubiG_at	5.11	5.80	5.55	5.58	-0.11	1.00
PA3172_at	3.12	3.47	3.01	3.50	0.04	1.00
PA3173_at	3.29	3.36	3.50	3.29	-0.07	1.00
PA3174_at	3.07	3.12	3.22	3.08	-0.06	1.00
PA3175_at	2.70	2.62	2.71	2.70	-0.04	1.00
PA3176_gltS_at	3.14	2.98	3.02	2.99	0.06	1.00
PA3177_at	5.68	6.66	5.88	5.93	0.26	1.00
PA3178_at	4.09	4.09	3.89	4.12	0.09	1.00
PA3179_at	4.75	4.71	4.64	4.95	-0.07	1.00
PA3180_at	3.18	2.83	3.16	2.78	0.04	1.00
PA3181_at	4.03	4.28	4.10	4.06	0.08	1.00
PA3182_at	6.15	6.72	6.32	6.20	0.17	1.00
PA3185_at	3.15	3.04	3.04	3.00	0.08	1.00
PA3189_at	2.81	2.77	2.93	2.79	-0.07	1.00
PA3192_gltR_at	3.08	3.21	3.19	3.18	-0.04	1.00
PA3194_edd_at	6.35	6.05	6.23	6.40	-0.12	1.00
PA3196_at	3.77	3.49	3.63	3.55	0.04	1.00
PA3197_at	2.81	2.99	2.83	2.80	0.09	1.00
PA3198_at	4.11	4.23	4.09	4.19	0.03	1.00
PA3199_at	3.37	3.65	3.60	3.50	-0.04	1.00
PA3202_at	4.20	4.63	4.08	4.35	0.21	1.00
PA3210_trkH_at	2.68	2.68	2.67	2.65	0.02	1.00
PA3212_at	5.13	5.30	5.37	4.78	0.14	1.00
PA3213_at	3.08	3.11	3.04	2.92	0.11	1.00
PA3214_at	4.20	4.02	4.09	4.06	0.04	1.00
PA3215_at	2.99	2.85	2.84	2.82	0.09	1.00
PA3216_at	3.72	3.67	3.89	3.67	-0.08	1.00
PA3219_at	4.25	3.61	3.90	4.04	-0.04	1.00
PA3220_at	2.45	2.45	2.50	2.45	-0.03	1.00
PA3223_acpD_at	3.04	3.06	3.16	3.07	-0.06	1.00
PA3224_at	3.38	3.19	3.26	3.25	0.03	1.00
PA3225_at	5.64	5.82	5.83	5.90	-0.13	1.00
PA3226_at	4.11	4.37	4.18	4.20	0.05	1.00
PA3229_at	3.01	2.97	2.67	3.23	0.04	1.00
PA3230_at	4.36	3.97	4.15	4.06	0.06	1.00
PA3231_at	2.10	2.08	2.19	2.11	-0.06	1.00
PA3232_at	2.49	2.32	2.48	2.55	-0.11	1.00
PA3233_at	2.59	2.37	2.70	2.52	-0.13	1.00
PA3235_at	3.44	2.74	3.31	3.40	-0.26	1.00
PA3236_at	3.45	3.19	3.30	3.14	0.10	1.00

PA3240_at	4.01	3.82	4.00	3.91	-0.04	1.00
PA3242_at	3.55	3.77	3.46	3.74	0.06	1.00
PA3245_minE_at	2.80	3.08	2.93	2.72	0.11	1.00
PA3246_rluA_at	4.71	5.00	5.02	4.79	-0.05	1.00
PA3249_at	3.26	3.00	2.94	3.09	0.11	1.00
PA3250_at	3.10	3.03	3.15	3.14	-0.08	1.00
PA3252_at	2.28	2.26	2.17	2.20	0.08	1.00
PA3253_at	2.87	3.05	3.21	2.97	-0.13	1.00
PA3254_at	3.03	2.79	2.95	2.98	-0.05	1.00
PA3256_at	2.99	2.86	2.85	2.76	0.12	1.00
PA3258_at	2.95	2.76	2.69	2.97	0.02	1.00
PA3260_at	5.35	6.28	5.99	5.96	-0.16	1.00
PA3261_at	3.58	3.33	3.61	3.54	-0.12	1.00
PA3262_at	4.18	4.66	4.33	4.35	0.08	1.00
PA3263_at	4.14	4.29	4.28	4.31	-0.08	1.00
PA3264_at	4.04	3.40	3.48	3.72	0.12	1.00
PA3265_at	3.43	2.92	3.22	3.21	-0.04	1.00
PA3266_capB_at	2.87	2.73	2.83	2.65	0.06	1.00
PA3267_at	4.29	4.08	4.31	4.27	-0.11	1.00
PA3268_at	3.38	3.15	3.26	3.22	0.02	1.00
PA3270_at	5.15	5.13	5.17	5.24	-0.07	1.00
PA3271_at	2.32	2.22	2.26	2.40	-0.06	1.00
PA3272_at	3.49	3.72	3.61	3.80	-0.10	1.00
PA3273_at	3.79	3.79	3.90	3.94	-0.13	1.00
PA3277_at	4.27	4.23	4.23	4.16	0.05	1.00
PA3278_at	6.53	6.79	6.82	6.78	-0.14	1.00
PA3280_oprO_at	2.65	2.56	2.50	2.61	0.05	1.00
PA3281_at	3.08	3.10	2.83	3.23	0.06	1.00
PA3282_at	3.06	3.16	3.00	2.96	0.13	1.00
PA3286_at	4.61	4.56	4.32	4.61	0.12	1.00
PA3289_at	3.01	2.66	2.98	2.94	-0.13	1.00
PA3291_at	2.59	2.69	2.79	2.65	-0.08	1.00
PA3295_at	2.70	2.92	3.00	2.87	-0.13	1.00
PA3296_phoA_at	3.09	2.99	3.12	3.11	-0.07	1.00
PA3299_fadD1_at	4.85	5.23	4.83	5.08	0.09	1.00
PA3301_at	3.65	3.72	3.70	3.74	-0.03	1.00
PA3302_at	3.24	3.16	3.26	3.20	-0.03	1.00
PA3304_at	2.40	2.50	2.51	2.48	-0.05	1.00
PA3305_at	4.51	4.24	4.34	4.58	-0.08	1.00
PA3307_r_at	7.33	7.44	7.28	7.24	0.13	1.00
PA3308_hepA_at	2.95	3.10	3.10	3.12	-0.09	1.00
PA3312_at	3.72	3.49	3.40	3.58	0.11	1.00
PA3313_at	3.67	3.97	3.72	3.82	0.05	1.00
PA3315_at	3.64	3.21	3.66	3.35	-0.08	1.00

PA3317_at	3.21	2.98	3.11	3.00	0.05	1.00
PA3318_at	3.49	3.16	3.51	3.47	-0.17	1.00
PA3319_plcN_at	3.08	2.85	2.98	2.88	0.04	1.00
PA3320_at	2.32	2.53	2.51	2.39	-0.03	1.00
PA3322_at	3.52	3.59	3.47	3.86	-0.11	1.00
PA3323_at	2.73	2.55	2.78	2.64	-0.07	1.00
PA3324_at	3.21	2.88	3.06	3.11	-0.04	1.00
PA3338_at	2.93	2.86	2.99	2.89	-0.05	1.00
PA3339_at	4.26	3.76	3.93	3.97	0.06	1.00
PA3344_recQ_at	5.23	5.30	5.09	5.23	0.11	1.00
PA3345_at	3.87	4.12	3.89	3.87	0.12	1.00
PA3346_at	4.15	4.11	4.35	4.17	-0.13	1.00
PA3349_at	4.09	4.88	4.38	4.81	-0.11	1.00
PA3350_at	3.98	4.11	4.13	4.16	-0.10	1.00
PA3351_at	7.72	8.61	8.07	8.16	0.05	1.00
PA3352_at	6.14	6.53	6.27	6.52	-0.07	1.00
PA3354_at	4.24	4.39	4.52	4.31	-0.10	1.00
PA3355_at	3.37	3.37	3.30	3.25	0.09	1.00
PA3356_at	6.13	5.97	5.99	5.99	0.06	1.00
PA3360_at	3.01	2.87	3.02	3.12	-0.13	1.00
PA3363_amiR_at	2.84	2.58	2.63	2.66	0.07	1.00
PA3364_amiC_at	2.41	2.50	2.59	2.56	-0.12	1.00
PA3365_at	2.50	2.46	2.55	2.51	-0.05	1.00
PA3367_at	2.50	2.64	2.39	2.67	0.04	1.00
PA3370_at	5.53	5.34	5.44	5.30	0.06	1.00
PA3371_at	4.21	4.58	4.32	4.59	-0.06	1.00
PA3374_at	4.65	4.17	4.65	4.33	-0.08	1.00
PA3375_at	3.07	2.55	2.98	2.85	-0.11	1.00
PA3376_at	3.31	2.86	3.21	3.17	-0.10	1.00
PA3377_at	2.62	2.43	2.68	2.62	-0.12	1.00
PA3378_at	3.84	2.80	3.23	3.11	0.15	1.00
PA3379_at	2.99	2.65	2.85	3.01	-0.11	1.00
PA3381_at	3.47	3.08	3.39	3.36	-0.11	1.00
PA3388_at	4.52	4.53	4.53	4.44	0.04	1.00
PA3390_at	2.65	2.50	2.39	2.53	0.12	1.00
PA3392_nosZ_at	4.88	5.16	5.15	5.01	-0.06	1.00
PA3393_nosD_at	4.09	4.01	4.05	4.12	-0.04	1.00
PA3394_nosF_at	4.34	4.54	4.20	4.54	0.07	1.00
PA3395_nosY_at	3.26	3.30	3.39	3.08	0.04	1.00
PA3396_nosL_at	3.11	2.85	2.81	3.02	0.07	1.00
PA3398_at	3.88	3.88	3.79	3.83	0.08	1.00
PA3400_at	2.62	2.56	2.67	2.41	0.05	1.00
PA3401_at	3.08	3.18	2.98	3.08	0.10	1.00
PA3402_at	2.60	2.54	2.57	2.76	-0.10	1.00

PA3403_at	4.35	4.16	4.29	4.12	0.05	1.00
PA3404_at	2.45	2.20	2.33	2.41	-0.04	1.00
PA3405_hasE_at	2.86	2.52	2.67	2.79	-0.04	1.00
PA3407_hasAp_at	2.34	2.31	2.34	2.25	0.03	1.00
PA3411_r_at	3.17	3.23	3.22	3.02	0.08	1.00
PA3412_at	2.65	2.50	2.80	2.59	-0.12	1.00
PA3416_at	4.50	4.64	4.80	4.64	-0.15	1.00
PA3419_at	5.53	5.22	5.13	5.33	0.15	1.00
PA3421_at	2.36	2.26	2.25	2.25	0.06	1.00
PA3422_at	4.48	3.97	4.25	4.46	-0.12	1.00
PA3423_at	2.93	2.85	2.88	2.83	0.04	1.00
PA3424_at	4.01	3.88	3.95	3.79	0.07	1.00
PA3425_at	3.34	3.15	3.37	3.02	0.06	1.00
PA3428_at	3.85	3.36	3.60	3.92	-0.15	1.00
PA3429_at	3.08	2.96	3.06	2.90	0.04	1.00
PA3430_at	3.87	3.71	3.49	3.86	0.11	1.00
PA3431_at	4.96	5.13	4.88	5.10	0.06	1.00
PA3432_i_at	3.20	3.05	3.00	3.01	0.12	1.00
PA3435_at	3.00	3.60	2.98	3.36	0.13	1.00
PA3438_folE1_at	2.27	2.12	2.39	2.21	-0.11	1.00
PA3445_at	2.67	2.64	2.71	2.70	-0.05	1.00
PA3446_at	3.16	3.00	2.91	2.96	0.14	1.00
PA3447_at	3.19	3.08	3.09	2.98	0.10	1.00
PA3448_at	2.40	2.53	2.54	2.44	-0.03	1.00
PA3449_at	3.62	2.99	3.12	3.16	0.16	1.00
PA3452_mqoA_at	2.82	3.05	2.81	2.94	0.06	1.00
PA3454_at	4.18	3.54	3.80	3.81	0.06	1.00
PA3456_at	3.51	3.68	3.62	3.28	0.15	1.00
PA3457_at	3.50	3.31	3.35	3.40	0.03	1.00
PA3461_at	4.42	4.13	4.25	4.12	0.09	1.00
PA3464_at	4.22	4.06	4.41	4.07	-0.10	1.00
PA3465_at	5.07	5.07	4.78	5.04	0.16	1.00
PA3467_at	3.58	3.23	3.32	3.24	0.13	1.00
PA3468_at	2.92	2.98	2.86	2.94	0.05	1.00
PA3469_at	2.71	3.16	3.10	3.03	-0.14	1.00
PA3470_i_at	2.15	2.25	2.45	2.23	-0.14	1.00
PA3471_at	3.44	3.39	3.51	3.57	-0.12	1.00
PA3473_at	2.98	2.95	2.93	2.92	0.04	1.00
PA3474_at	2.77	2.52	2.56	2.49	0.12	1.00
PA3475_pheC_at	3.17	2.77	2.68	2.97	0.14	1.00
PA3476_rhlI_at	3.89	4.11	3.92	4.01	0.04	1.00
PA3477_rhlR_at	5.47	5.35	5.55	5.53	-0.13	1.00
PA3480_at	3.46	3.76	3.54	3.46	0.11	1.00

PA3481_at	4.58	4.65	4.71	4.58	-0.03	1.00
PA3482_metG_at	4.04	4.56	4.59	4.28	-0.14	1.00
PA3483_at	3.52	3.27	3.38	3.18	0.12	1.00
PA3484_at	3.38	3.32	3.25	3.62	-0.09	1.00
PA3486_at	3.24	2.88	3.35	3.10	-0.17	1.00
PA3487_at	3.29	2.96	3.14	3.18	-0.03	1.00
PA3489_at	3.56	3.79	3.65	3.89	-0.10	1.00
PA3490_at	3.13	3.14	3.03	3.12	0.06	1.00
PA3492_at	3.57	3.31	3.54	3.64	-0.15	1.00
PA3493_at	4.03	3.83	4.08	3.94	-0.08	1.00
PA3494_at	4.22	4.00	4.38	4.08	-0.12	1.00
PA3495_nth_at	4.25	3.99	4.21	4.17	-0.07	1.00
PA3497_at	3.76	3.14	3.76	3.28	-0.07	1.00
PA3498_at	3.08	2.67	2.88	2.65	0.11	1.00
PA3500_at	3.86	3.63	3.96	3.63	-0.05	1.00
PA3502_at	2.56	2.49	2.49	2.72	-0.08	1.00
PA3504_at	3.20	3.05	3.17	3.26	-0.09	1.00
PA3505_at	3.88	3.37	3.76	3.28	0.10	1.00
PA3506_at	3.46	3.11	3.22	3.66	-0.16	1.00
PA3507_at	2.97	2.71	3.11	2.87	-0.15	1.00
PA3508_at	3.78	3.31	3.37	3.38	0.17	1.00
PA3509_at	3.51	3.29	3.49	3.35	-0.02	1.00
PA3510_at	2.74	2.35	2.62	2.65	-0.09	1.00
PA3511_at	3.03	3.03	3.06	3.17	-0.08	1.00
PA3513_at	2.84	2.79	2.88	2.83	-0.04	1.00
PA3514_at	2.53	2.69	2.73	2.59	-0.05	1.00
PA3515_at	2.97	2.99	3.27	2.99	-0.15	1.00
PA3516_at	3.86	3.43	3.31	3.58	0.20	1.00
PA3518_at	2.80	2.68	2.63	2.68	0.09	1.00
PA3519_at	2.33	2.26	2.30	2.33	-0.02	1.00
PA3520_at	3.14	3.06	3.24	3.14	-0.09	1.00
PA3521_at	3.24	3.07	3.17	3.05	0.05	1.00
PA3523_at	3.05	3.10	2.85	3.11	0.10	1.00
PA3524_gloA1_at	4.03	4.51	3.93	4.21	0.20	1.00
PA3525_argG_at	6.20	6.36	6.29	6.45	-0.09	1.00
PA3528_rnt_at	3.63	3.61	3.60	3.53	0.06	1.00
PA3530_at	3.00	2.51	2.92	2.70	-0.06	1.00
PA3535_at	2.94	2.59	2.54	2.71	0.14	1.00
PA3536_at	5.50	5.16	5.24	5.21	0.10	1.00
PA3537_argF_at	3.95	4.09	3.86	4.14	0.03	1.00
PA3538_at	3.95	4.18	3.94	4.04	0.07	1.00
PA3542_at	2.72	2.45	2.75	2.49	-0.03	1.00
PA3545_algG_at	2.68	2.56	2.60	2.57	0.04	1.00
PA3548_algI_at	2.51	2.28	2.44	2.40	-0.02	1.00

PA3549_algJ_at	3.27	3.17	3.14	3.18	0.06	1.00
PA3550_algF_at	4.07	4.10	4.14	4.08	-0.02	1.00
PA3551_algA_at	3.01	2.73	3.11	2.81	-0.09	1.00
PA3555_at	3.47	3.04	3.36	3.00	0.07	1.00
PA3556_at	3.75	3.54	3.62	3.73	-0.03	1.00
PA3559_at	3.09	2.59	2.61	2.67	0.20	1.00
PA3560_fruA_at	3.96	4.13	3.86	3.97	0.13	1.00
PA3563_fruR_at	4.94	5.31	5.03	4.96	0.13	1.00
PA3564_at	2.99	2.62	2.87	2.85	-0.06	1.00
PA3571_mmsR_at	3.73	3.34	3.50	3.50	0.03	1.00
PA3574_at	3.25	3.15	3.16	3.29	-0.03	1.00
PA3576_at	4.23	4.60	4.10	4.38	0.18	1.00
PA3577_i_at	2.46	2.65	2.59	2.38	0.07	1.00
PA3578_at	3.62	3.93	3.65	3.71	0.10	1.00
PA3580_at	3.23	3.34	3.34	3.29	-0.03	1.00
PA3585_glpM_at	2.45	2.48	2.55	2.33	0.02	1.00
PA3586_at	2.94	2.70	2.93	2.81	-0.05	1.00
PA3587_metR_at	5.83	5.62	5.62	5.70	0.07	1.00
PA3588_at	3.81	3.34	3.67	3.92	-0.22	1.00
PA3589_at	2.65	2.50	2.58	2.78	-0.11	1.00
PA3590_at	2.61	2.56	2.54	2.70	-0.03	1.00
PA3591_at	2.70	2.59	2.70	2.78	-0.09	1.00
PA3592_at	3.42	3.26	3.33	3.26	0.05	1.00
PA3593_at	3.26	2.93	2.93	3.16	0.05	1.00
PA3594_at	4.46	3.91	4.36	4.37	-0.17	1.00
PA3596_at	3.62	3.11	3.39	3.48	-0.07	1.00
PA3597_at	3.17	2.73	2.92	2.71	0.13	1.00
PA3598_at	3.41	3.56	3.42	3.42	0.07	1.00
PA3600_at	2.71	2.70	2.65	2.65	0.05	1.00
PA3603_dgkA_at	4.07	3.87	3.89	3.76	0.14	1.00
PA3606_at	3.14	3.19	3.06	3.05	0.11	1.00
PA3609_potC_at	4.66	4.28	4.59	4.46	-0.06	1.00
PA3610_potD_at	2.66	2.64	2.77	2.44	0.04	1.00
PA3618_at	5.54	5.44	5.67	5.47	-0.08	1.00
PA3619_at	3.05	2.95	3.38	2.94	-0.16	1.00
PA3624_pcm_at	4.27	4.15	4.18	4.36	-0.06	1.00
PA3626_at	3.32	3.15	3.24	3.01	0.11	1.00
PA3628_at	5.06	4.94	4.94	4.92	0.07	1.00
PA3629_adhC_at	3.11	3.14	3.48	3.05	-0.14	1.00
PA3630_at	4.47	4.43	4.38	4.43	0.04	1.00
PA3631_at	4.88	4.80	4.83	4.64	0.11	1.00
PA3634_at	7.24	7.25	7.28	7.29	-0.04	1.00
PA3638_at	3.11	3.11	3.20	3.14	-0.05	1.00
PA3640_dnaE_at	3.39	3.28	3.17	3.28	0.11	1.00

PA3641_at	3.24	3.05	2.96	3.14	0.09	1.00
PA3643_lpxB_at	5.07	5.04	4.95	5.11	0.03	1.00
PA3645_fabZ_at	3.90	4.65	4.05	4.21	0.14	1.00
PA3646_lpxD_at	3.95	4.59	4.28	4.35	-0.05	1.00
PA3647_at	5.79	5.80	5.74	5.71	0.07	1.00
PA3648_at	5.23	5.41	5.26	5.55	-0.09	1.00
PA3650_dxr_at	3.54	3.83	3.48	3.76	0.06	1.00
PA3651_cdsA_at	3.85	4.06	3.83	3.99	0.04	1.00
PA3652_uppS_at	4.30	4.73	4.59	4.35	0.05	1.00
PA3653_frr_at	6.58	7.11	6.92	6.91	-0.07	1.00
PA3655_tsf_at	6.69	7.01	6.69	6.89	0.06	1.00
PA3656_rpsB_at	5.87	6.63	6.42	6.61	-0.26	1.00
PA3658_glnD_at	4.65	4.31	4.33	4.36	0.13	1.00
PA3663_at	4.73	4.26	4.44	4.19	0.18	1.00
PA3665_at	5.38	6.20	5.63	5.51	0.22	1.00
PA3666_dapD_at	5.59	5.91	5.78	5.89	-0.09	1.00
PA3668_at	3.45	3.21	3.33	3.23	0.04	1.00
PA3669_at	3.70	3.80	3.94	3.76	-0.10	1.00
PA3671_at	3.59	3.02	3.11	3.34	0.08	1.00
PA3673_plsB_at	3.33	3.06	3.01	3.22	0.08	1.00
PA3676_at	4.76	4.44	4.57	4.47	0.09	1.00
PA3677_at	3.69	3.58	3.53	3.85	-0.06	1.00
PA3678_at	4.80	5.36	5.04	5.28	-0.08	1.00
PA3679_at	3.35	3.19	3.31	3.14	0.04	1.00
PA3680_at	2.73	2.94	2.85	2.94	-0.06	1.00
PA3682_at	2.79	2.37	2.50	2.55	0.05	1.00
PA3683_at	3.55	3.67	3.49	3.63	0.05	1.00
PA3685_at	4.07	3.89	4.07	3.97	-0.04	1.00
PA3686_adk_at	3.35	3.68	3.51	3.61	-0.04	1.00
PA3687_ppc_at	4.85	5.18	5.01	4.91	0.05	1.00
PA3688_at	4.07	3.94	4.03	4.22	-0.13	1.00
PA3695_at	3.03	3.01	2.99	3.13	-0.04	1.00
PA3696_at	2.83	2.55	2.72	2.86	-0.10	1.00
PA3697_at	4.79	4.99	4.91	5.13	-0.13	1.00
PA3698_at	3.69	3.70	3.54	3.59	0.13	1.00
PA3699_at	3.39	3.36	3.31	3.17	0.13	1.00
PA3700_lysS_at	4.87	4.92	4.76	4.81	0.11	1.00
PA3701_prfB_at	3.31	3.56	3.45	3.71	-0.15	1.00
PA3702_at	4.62	4.65	4.74	4.62	-0.04	1.00
PA3703_at	4.50	4.49	4.23	4.52	0.12	1.00
PA3707_at	3.38	3.26	3.53	3.34	-0.12	1.00
PA3708_at	2.81	2.88	2.89	2.87	-0.04	1.00
PA3709_at	2.75	2.49	2.61	2.70	-0.04	1.00
PA3710_at	3.43	3.30	3.22	3.23	0.14	1.00

PA3713_at	2.61	2.55	2.68	2.55	-0.03	1.00
PA3714_at	2.98	2.84	2.95	2.70	0.08	1.00
PA3715_at	2.36	2.47	2.59	2.36	-0.06	1.00
PA3718_at	3.39	3.71	3.48	3.50	0.06	1.00
PA3721_at	4.21	4.42	4.18	4.27	0.09	1.00
PA3722_at	4.77	4.38	4.47	4.45	0.12	1.00
PA3725_recJ_at	4.55	4.54	4.58	4.58	-0.04	1.00
PA3726_at	3.76	3.94	3.69	3.73	0.14	1.00
PA3728_at	2.75	2.67	2.67	2.60	0.08	1.00
PA3729_at	4.72	4.60	4.62	4.45	0.12	1.00
PA3734_at	3.07	2.64	2.75	3.15	-0.10	1.00
PA3735_thrC_at	4.33	4.59	4.73	4.41	-0.11	1.00
PA3737_dsbC_at	3.43	3.33	3.25	3.34	0.09	1.00
PA3738_xerD_at	3.26	3.05	3.18	3.25	-0.06	1.00
PA3739_at	3.41	3.30	3.54	3.30	-0.07	1.00
PA3740_at	3.76	3.40	3.67	3.62	-0.06	1.00
PA3742_rplS_at	4.97	5.59	5.26	5.16	0.07	1.00
PA3743_trmD_at	6.81	7.63	7.05	6.86	0.26	1.00
PA3744_rimM_at	4.80	6.01	5.43	5.60	-0.11	1.00
PA3745_rpsP_at	5.23	6.03	5.36	5.49	0.21	1.00
PA3748_at	3.28	3.27	3.23	3.25	0.04	1.00
PA3749_at	2.95	2.58	2.73	2.89	-0.04	1.00
PA3751_purT_at	4.42	4.33	4.50	4.33	-0.04	1.00
PA3752_at	5.10	5.05	5.09	4.90	0.08	1.00
PA3754_at	3.11	3.19	3.06	3.15	0.04	1.00
PA3757_at	3.08	3.70	3.20	3.21	0.18	1.00
PA3759_at	3.44	3.28	3.34	3.62	-0.12	1.00
PA3760_at	4.12	3.97	3.99	3.79	0.16	1.00
PA3761_at	3.48	2.96	3.03	3.17	0.12	1.00
PA3765_at	2.92	2.91	3.15	2.97	-0.14	1.00
PA3767_at	3.03	2.98	2.99	2.98	0.02	1.00
PA3769_guaA_at	5.93	5.96	6.09	5.98	-0.09	1.00
PA3774_at	3.01	2.70	2.76	2.90	0.03	1.00
PA3775_at	2.56	2.47	2.55	2.42	0.03	1.00
PA3776_at	3.01	2.60	2.92	2.62	0.03	1.00
PA3778_at	3.82	3.69	3.87	3.56	0.04	1.00
PA3780_at	2.56	2.51	2.57	2.57	-0.03	1.00
PA3781_at	2.82	2.83	3.05	2.87	-0.14	1.00
PA3783_at	2.37	2.24	2.45	2.31	-0.08	1.00
PA3788_at	6.05	6.21	6.12	6.10	0.02	1.00
PA3789_at	2.98	2.85	2.85	2.94	0.02	1.00
PA3792_leuA_at	5.06	5.31	5.16	5.25	-0.02	1.00
PA3793_at	4.74	4.84	4.49	4.88	0.10	1.00
PA3796_at	4.99	5.90	5.39	5.41	0.05	1.00

PA3797_at	4.06	4.50	4.14	4.31	0.05	1.00
PA3800_at	5.06	5.16	5.26	5.08	-0.06	1.00
PA3802_hisS_at	3.47	3.68	3.50	3.49	0.08	1.00
PA3803_at	5.47	5.88	5.55	5.73	0.04	1.00
PA3804_at	4.16	4.37	4.33	4.33	-0.06	1.00
PA3805_pilF_at	4.09	3.98	3.83	3.98	0.13	1.00
PA3806_at	4.74	5.47	4.94	4.98	0.15	1.00
PA3807_ndk_at	6.13	7.17	6.90	6.87	-0.24	1.00
PA3808_at	4.81	4.45	4.65	4.48	0.07	1.00
PA3810_hscA_at	2.67	3.00	2.62	2.91	0.07	1.00
PA3813_iscU_at	4.70	5.75	4.99	4.86	0.29	1.00
PA3818_at	3.20	3.33	3.24	3.04	0.13	1.00
PA3820_secF_at	4.46	4.50	4.55	4.60	-0.10	1.00
PA3823_tgt_at	3.93	3.87	3.74	3.78	0.14	1.00
PA3824_queA_at	3.40	3.35	3.60	3.23	-0.04	1.00
PA3825_at	2.78	2.68	2.74	2.68	0.02	1.00
PA3826_at	2.72	2.37	2.36	2.41	0.16	1.00
PA3829_at	2.97	2.84	3.04	2.94	-0.08	1.00
PA3832_holC_at	3.43	3.56	3.53	3.51	-0.02	1.00
PA3833_at	5.24	4.98	4.86	5.17	0.09	1.00
PA3834_valS_at	3.49	3.63	3.44	3.50	0.09	1.00
PA3835_at	3.42	2.88	3.18	3.21	-0.04	1.00
PA3837_at	3.78	3.37	3.72	3.71	-0.14	1.00
PA3838_at	3.39	2.91	3.31	3.34	-0.17	1.00
PA3839_at	3.51	3.62	3.51	3.36	0.13	1.00
PA3840_at	4.02	3.47	3.42	3.67	0.20	1.00
PA3843_at	6.18	6.31	6.28	6.24	-0.02	1.00
PA3846_at	3.12	3.45	3.21	3.59	-0.12	1.00
PA3848_at	3.05	3.16	3.11	2.95	0.07	1.00
PA3849_at	3.01	2.76	3.08	2.99	-0.15	1.00
PA3850_at	4.90	5.01	4.82	4.94	0.07	1.00
PA3851_at	2.70	2.95	2.62	2.73	0.15	1.00
PA3852_at	4.16	4.27	4.11	4.14	0.09	1.00
PA3854_at	4.61	4.56	4.75	4.37	0.02	1.00
PA3855_at	3.29	3.19	3.14	3.17	0.08	1.00
PA3856_at	3.30	3.29	3.06	3.36	0.09	1.00
PA3857_at	5.27	5.96	5.29	5.80	0.07	1.00
PA3858_at	4.12	4.05	4.07	3.97	0.06	1.00
PA3861_rhlB_at	3.40	3.60	3.31	3.42	0.13	1.00
PA3863_at	3.34	2.94	2.95	2.97	0.18	1.00
PA3866_at	4.69	4.75	4.76	4.39	0.15	1.00
PA3867_at	3.20	2.70	2.73	2.82	0.18	1.00
PA3868_at	4.26	4.23	4.31	4.28	-0.05	1.00
PA3869_at	2.96	2.99	2.95	2.86	0.07	1.00

PA3870_moaA1_ at	3.39	3.40	3.19	3.44	0.08	1.00
PA3871_at	4.11	4.26	4.07	4.12	0.09	1.00
PA3873_narJ_at	4.79	5.53	5.04	4.68	0.30	1.00
PA3874_narH_at	5.11	5.45	5.18	5.29	0.04	1.00
PA3875_narG_at	4.61	4.67	4.58	4.64	0.04	1.00
PA3879_narL_at	4.57	5.15	5.03	5.02	-0.17	1.00
PA3882_at	3.11	3.01	3.13	2.76	0.11	1.00
PA3883_at	3.92	3.80	4.06	3.92	-0.13	1.00
PA3884_at	3.42	3.04	3.01	3.23	0.11	1.00
PA3885_at	3.44	3.00	3.25	3.13	0.03	1.00
PA3886_at	3.62	3.72	3.73	3.73	-0.06	1.00
PA3887_nhaP_at	3.13	3.20	3.33	2.96	0.02	1.00
PA3888_at	3.65	3.59	3.87	3.60	-0.11	1.00
PA3889_at	3.19	3.33	3.43	3.31	-0.10	1.00
PA3890_at	3.05	2.76	2.59	2.99	0.12	1.00
PA3891_at	3.09	3.08	2.95	3.10	0.06	1.00
PA3892_at	3.40	2.61	3.32	3.26	-0.29	1.00
PA3893_at	3.41	2.94	2.99	3.19	0.08	1.00
PA3894_at	2.59	2.56	2.44	2.60	0.06	1.00
PA3896_at	3.29	3.29	3.39	3.27	-0.04	1.00
PA3898_at	4.15	3.66	3.74	3.83	0.12	1.00
PA3899_at	2.20	2.43	2.44	2.37	-0.09	1.00
PA3900_at	3.79	3.30	3.70	3.67	-0.14	1.00
PA3901_fecA_at	2.94	3.01	3.20	2.96	-0.10	1.00
PA3903_prfC_at	3.77	3.95	3.91	3.69	0.06	1.00
PA3904_i_at	4.07	5.10	4.64	4.82	-0.14	1.00
PA3905_at	3.71	3.48	3.66	3.60	-0.03	1.00
PA3907_at	5.06	4.64	4.89	5.04	-0.12	1.00
PA3908_at	3.52	3.14	3.39	3.36	-0.05	1.00
PA3910_at	4.12	4.26	4.37	4.15	-0.07	1.00
PA3913_at	2.71	2.90	3.06	2.81	-0.13	1.00
PA3915_moaB1_ at	3.90	3.99	3.88	3.78	0.11	1.00
PA3916_moaE_at	3.25	3.99	3.60	3.93	-0.14	1.00
PA3917_moaD_at	4.45	4.35	4.33	4.42	0.03	1.00
PA3918_moaC_at	4.75	4.96	4.87	4.92	-0.05	1.00
PA3919_at	4.85	4.82	4.90	4.93	-0.08	1.00
PA3923_at	2.73	2.67	2.86	2.71	-0.09	1.00
PA3924_at	3.19	2.82	2.94	2.88	0.09	1.00
PA3925_at	4.49	4.42	4.37	4.47	0.03	1.00
PA3927_at	3.48	3.49	3.51	3.25	0.10	1.00
PA3929_cioB_at	3.75	3.69	3.72	3.81	-0.05	1.00
PA3930_cioA_at	3.20	3.35	2.99	3.38	0.09	1.00
PA3931_at	4.79	4.14	4.35	4.51	0.03	1.00

PA3932_at	2.96	2.78	2.88	2.77	0.04	1.00
PA3933_at	2.73	2.55	2.60	2.49	0.09	1.00
PA3934_at	3.76	3.29	3.38	3.50	0.08	1.00
PA3935_tauD_at	3.43	3.12	3.35	3.14	0.03	1.00
PA3936_at	4.61	4.35	4.45	4.34	0.09	1.00
PA3938_at	2.81	2.75	2.57	2.72	0.13	1.00
PA3939_at	5.05	4.68	5.10	4.81	-0.09	1.00
PA3941_at	2.74	2.77	2.79	2.57	0.08	1.00
PA3942_tesB_at	3.13	3.39	3.14	3.23	0.08	1.00
PA3944_at	3.26	3.80	3.43	3.70	-0.04	1.00
PA3946_at	3.66	3.19	3.41	3.26	0.09	1.00
PA3947_at	4.13	3.89	3.97	3.82	0.12	1.00
PA3948_at	2.75	3.05	2.79	2.89	0.06	1.00
PA3950_at	4.47	4.49	4.57	4.58	-0.10	1.00
PA3951_at	3.28	3.18	3.14	3.51	-0.09	1.00
PA3953_at	3.31	3.35	3.38	3.55	-0.13	1.00
PA3955_at	4.44	3.92	4.31	4.19	-0.08	1.00
PA3958_at	3.61	3.44	3.48	3.51	0.03	1.00
PA3959_at	2.74	2.83	3.01	2.74	-0.09	1.00
PA3960_at	2.96	2.76	2.85	2.77	0.05	1.00
PA3961_at	3.44	3.55	3.57	3.49	-0.04	1.00
PA3962_at	2.91	3.68	2.95	3.17	0.23	1.00
PA3965_at	3.32	3.03	2.84	3.20	0.15	1.00
PA3968_at	3.57	3.25	4.13	3.30	-0.31	1.00
PA3969_at	4.63	4.44	4.34	4.43	0.15	1.00
PA3970_amn_at	3.07	3.24	3.05	3.32	-0.03	1.00
PA3971_at	4.20	3.79	4.15	3.98	-0.07	1.00
PA3972_at	4.31	4.65	4.29	4.74	-0.04	1.00
PA3975_thiD_at	4.73	4.96	4.88	4.99	-0.09	1.00
PA3980_at	3.99	4.41	4.09	4.02	0.14	1.00
PA3981_at	2.58	3.01	2.82	2.65	0.06	1.00
PA3983_at	3.91	4.00	3.94	3.76	0.11	1.00
PA3984_lnt_at	3.24	3.27	3.38	3.35	-0.11	1.00
PA3986_at	3.38	3.69	3.73	3.67	-0.17	1.00
PA3987_leuS_at	3.27	3.61	3.26	3.33	0.15	1.00
PA3988_at	3.32	3.34	3.32	3.30	0.02	1.00
PA3989_holA_at	5.34	5.42	5.36	5.31	0.05	1.00
PA3991_at	2.92	2.43	2.44	2.57	0.17	1.00
PA3992_at	3.57	3.16	3.35	3.27	0.05	1.00
PA3994_at	4.72	4.37	4.70	4.51	-0.06	1.00
PA3997_lipB_at	2.95	3.30	2.96	3.20	0.04	1.00
PA3998_at	4.18	4.72	4.59	4.39	-0.04	1.00
PA3999_dacC_at	6.59	6.92	6.67	6.72	0.06	1.00
PA4000_at	4.13	4.19	4.17	4.05	0.05	1.00

PA4005_at	4.72	4.72	4.83	4.86	-0.12	1.00
PA4006_at	2.80	2.96	2.99	2.91	-0.07	1.00
PA4007_proA_at	3.83	3.97	3.86	3.76	0.09	1.00
PA4009_at	2.66	2.60	2.81	2.59	-0.07	1.00
PA4010_at	4.69	4.85	4.86	4.98	-0.15	1.00
PA4012_at	2.89	2.96	2.75	3.03	0.04	1.00
PA4014_at	4.85	5.20	4.90	4.91	0.12	1.00
PA4015_at	4.99	4.69	4.65	4.91	0.06	1.00
PA4016_at	3.05	3.11	3.26	3.08	-0.09	1.00
PA4018_r_at	5.24	4.88	5.10	5.12	-0.05	1.00
PA4021_at	4.63	4.50	4.64	4.53	-0.02	1.00
PA4024_eutB_at	2.93	2.75	3.16	2.82	-0.15	1.00
PA4026_at	4.08	4.21	4.13	4.23	-0.04	1.00
PA4028_i_at	3.36	2.77	3.15	3.12	-0.07	1.00
PA4030_at	3.99	4.40	4.02	4.08	0.15	1.00
PA4032_at	4.23	4.26	4.15	4.46	-0.06	1.00
PA4033_at	1.95	1.88	2.05	1.97	-0.09	1.00
PA4034_aqpZ_at	2.66	2.64	2.67	2.83	-0.10	1.00
PA4037_at	3.15	3.22	3.17	3.30	-0.05	1.00
PA4038_at	2.71	2.76	2.82	2.71	-0.03	1.00
PA4040_at	3.41	3.27	3.35	3.40	-0.03	1.00
PA4044_dxs_at	3.21	3.30	3.14	3.19	0.09	1.00
PA4045_at	3.38	3.48	3.29	3.35	0.11	1.00
PA4047_ribA_at	4.89	4.79	4.89	4.70	0.04	1.00
PA4048_at	3.06	2.93	2.89	3.16	-0.03	1.00
PA4050_pgpA_at	4.54	4.26	4.55	4.13	0.06	1.00
PA4051_thiL_at	4.28	4.73	4.80	4.41	-0.10	1.00
PA4054_ribB_at	4.97	4.76	4.88	4.69	0.08	1.00
PA4055_ribC_at	3.67	4.02	3.88	3.93	-0.06	1.00
PA4056_ribD_at	4.56	4.93	4.90	4.86	-0.14	1.00
PA4057_at	4.83	5.53	5.30	5.28	-0.11	1.00
PA4060_at	3.61	3.36	3.63	3.02	0.16	1.00
PA4063_at	2.57	2.39	2.52	2.34	0.05	1.00
PA4065_at	4.38	3.88	4.24	3.65	0.18	1.00
PA4068_at	4.79	4.40	4.65	4.69	-0.07	1.00
PA4071_at	3.14	2.69	2.86	3.03	-0.03	1.00
PA4072_at	3.70	3.22	3.28	3.29	0.18	1.00
PA4074_at	3.40	3.02	3.35	3.24	-0.08	1.00
PA4076_at	3.27	3.09	3.13	2.96	0.13	1.00
PA4081_at	2.81	2.59	2.74	2.62	0.02	1.00
PA4082_at	2.41	2.22	2.23	2.24	0.08	1.00
PA4084_at	2.87	2.67	2.73	2.67	0.07	1.00
PA4085_at	2.66	2.26	2.59	2.44	-0.06	1.00
PA4086_at	2.76	2.69	2.86	2.75	-0.08	1.00

PA4087_at	3.41	3.34	3.56	3.40	-0.10	1.00
PA4088_at	2.94	3.02	3.08	3.03	-0.07	1.00
PA4089_at	2.88	2.74	2.94	2.77	-0.04	1.00
PA4091_hpaA_at	2.54	2.43	2.44	2.63	-0.05	1.00
PA4093_at	2.62	2.43	2.38	2.55	0.06	1.00
PA4095_at	2.98	2.81	2.99	2.86	-0.03	1.00
PA4096_at	2.39	2.18	2.60	2.30	-0.17	1.00
PA4097_at	3.06	2.92	2.96	2.89	0.06	1.00
PA4098_at	4.16	3.98	4.31	4.01	-0.09	1.00
PA4099_at	2.45	2.39	2.51	2.49	-0.08	1.00
PA4100_at	3.01	2.34	2.71	3.00	-0.18	1.00
PA4102_at	3.02	3.09	3.19	3.13	-0.10	1.00
PA4105_at	3.63	3.11	3.60	3.50	-0.18	1.00
PA4106_at	3.24	3.12	3.18	3.14	0.02	1.00
PA4107_at	2.49	2.35	2.62	2.47	-0.12	1.00
PA4108_at	5.23	5.75	5.45	5.64	-0.05	1.00
PA4109_ampR_at	3.91	3.82	4.06	3.84	-0.08	1.00
PA4110_ampC_at	3.23	3.00	3.02	3.05	0.08	1.00
PA4111_i_at	4.24	4.69	4.50	4.54	-0.05	1.00
PA4114_at	3.52	3.47	3.46	3.47	0.03	1.00
PA4116_at	3.91	4.24	4.03	3.96	0.08	1.00
PA4117_at	3.21	3.28	3.15	3.29	0.02	1.00
PA4118_at	2.85	2.73	2.79	2.69	0.05	1.00
PA4121_at	2.83	2.77	2.77	2.68	0.08	1.00
PA4123_hpcC_at	3.29	3.26	3.27	3.40	-0.07	1.00
PA4124_hpcB_at	2.92	2.54	2.59	2.72	0.08	1.00
PA4125_hpcD_at	2.74	2.41	2.86	2.60	-0.15	1.00
PA4126_at	3.70	3.42	3.49	3.57	0.03	1.00
PA4127_hpcG_at	3.09	2.87	3.12	2.97	-0.07	1.00
PA4128_at	3.70	3.30	3.57	3.30	0.07	1.00
PA4135_at	4.13	4.20	4.20	4.18	-0.03	1.00
PA4136_at	3.16	3.03	3.23	3.09	-0.07	1.00
PA4137_at	2.42	2.31	2.42	2.17	0.07	1.00
PA4138_tyrS_at	3.50	3.51	3.43	3.35	0.12	1.00
PA4140_at	3.63	3.25	3.63	3.54	-0.15	1.00
PA4142_at	3.23	3.01	3.09	3.03	0.06	1.00
PA4143_at	4.81	4.52	4.68	4.71	-0.02	1.00
PA4145_at	2.47	2.56	2.79	2.55	-0.16	1.00
PA4146_at	4.19	3.47	3.79	3.51	0.18	1.00
PA4151_acoB_at	3.03	2.72	2.76	2.94	0.02	1.00
PA4152_at	3.82	3.40	3.44	3.43	0.17	1.00
PA4153_at	3.67	3.64	3.85	3.69	-0.12	1.00
PA4155_at	3.24	2.73	2.91	3.00	0.03	1.00
PA4157_at	2.91	2.57	2.69	2.74	0.02	1.00

PA4158_fepC_at	2.23	2.17	2.31	2.33	-0.11	1.00
PA4159_fepB_at	2.83	2.80	2.70	2.87	0.03	1.00
PA4161_fepG_at	3.06	2.75	2.79	2.85	0.09	1.00
PA4162_at	3.98	4.00	4.06	4.02	-0.05	1.00
PA4163_at	4.45	4.62	4.56	4.67	-0.08	1.00
PA4166_at	2.79	2.89	2.97	2.66	0.02	1.00
PA4167_at	2.93	2.64	2.75	2.92	-0.05	1.00
PA4168_at	2.98	2.93	3.15	2.87	-0.05	1.00
PA4169_at	3.16	2.91	2.98	3.04	0.02	1.00
PA4170_at	3.31	3.10	3.11	3.17	0.07	1.00
PA4172_at	2.97	2.59	2.63	2.86	0.03	1.00
PA4174_at	3.28	3.03	3.29	3.10	-0.04	1.00
PA4176_ppiC2_at	3.75	4.14	4.06	3.90	-0.04	1.00
PA4177_at	2.97	2.60	2.88	2.62	0.03	1.00
PA4178_at	3.11	2.84	2.83	2.89	0.12	1.00
PA4182_at	2.96	3.07	3.08	3.26	-0.15	1.00
PA4183_at	3.95	3.55	3.74	4.15	-0.19	1.00
PA4184_at	3.40	3.23	3.35	3.48	-0.10	1.00
PA4188_at	3.12	2.92	3.23	3.00	-0.10	1.00
PA4189_at	2.87	2.47	2.74	2.96	-0.19	1.00
PA4190_at	3.96	3.51	3.89	3.85	-0.14	1.00
PA4191_at	2.77	2.37	2.57	2.26	0.16	1.00
PA4192_at	3.33	2.86	3.29	3.03	-0.06	1.00
PA4193_at	3.40	3.13	3.34	3.12	0.03	1.00
PA4196_at	4.39	4.25	4.39	4.38	-0.06	1.00
PA4198_at	3.64	3.73	3.52	3.62	0.11	1.00
PA4200_at	3.33	3.05	3.16	3.09	0.06	1.00
PA4201_ddlA_at	2.95	3.04	2.92	3.15	-0.04	1.00
PA4203_at	3.32	4.01	3.64	3.48	0.10	1.00
PA4206_at	5.16	5.50	5.33	5.41	-0.04	1.00
PA4207_at	5.53	5.44	5.53	5.63	-0.09	1.00
PA4208_at	4.30	3.83	4.08	4.12	-0.03	1.00
PA4209_at	3.42	3.17	3.48	3.41	-0.15	1.00
PA4219_at	3.18	2.80	2.94	3.12	-0.04	1.00
PA4220_i_at	3.77	3.77	3.86	3.50	0.09	1.00
PA4221_fptA_at	2.90	2.95	2.93	2.76	0.08	1.00
PA4223_at	4.07	3.72	3.86	3.58	0.18	1.00
PA4224_at	3.68	3.25	3.32	3.44	0.08	1.00
PA4225_pchF_at	2.98	2.39	2.59	2.49	0.15	1.00
PA4226_pchE_at	3.02	2.67	2.77	2.81	0.06	1.00
PA4228_pchD_at	2.47	2.57	2.65	2.23	0.08	1.00
PA4229_pchC_at	2.81	2.54	2.70	2.58	0.04	1.00
PA4230_pchB_at	2.36	2.32	2.28	2.49	-0.05	1.00
PA4231_pchA_at	3.10	3.00	3.01	3.04	0.03	1.00

PA4232_ssb_at	4.59	5.19	5.11	5.15	-0.24	1.00
PA4233_at	3.60	3.39	3.58	3.47	-0.03	1.00
PA4235_bfrA_at	4.31	4.49	4.26	4.25	0.15	1.00
PA4239_rpsD_at	5.99	6.52	6.03	6.28	0.09	1.00
PA4240_rpsK_at	7.41	8.21	7.79	8.00	-0.08	1.00
PA4241_rpsM_at	5.62	6.72	6.31	6.26	-0.11	1.00
PA4242_rpmJ_at	5.63	6.46	6.14	5.82	0.07	1.00
PA4243_secY_at	3.98	4.80	4.46	4.50	-0.09	1.00
PA4244_rplO_at	6.76	7.38	7.15	7.15	-0.08	1.00
PA4246_rpsE_at	6.39	7.57	6.98	7.36	-0.19	1.00
PA4247_rplR_at	5.80	6.39	6.12	5.83	0.12	1.00
PA4248_rplF_at	7.14	8.44	7.98	8.20	-0.30	1.00
PA4249_rpsH_at	7.68	8.45	8.15	8.23	-0.12	1.00
PA4250_rpsN_at	8.48	9.08	9.00	8.94	-0.19	1.00
PA4251_rplE_at	9.17	9.75	9.54	9.67	-0.14	1.00
PA4253_rplN_at	7.54	8.24	8.02	8.11	-0.17	1.00
PA4255_rpmC_at	5.13	5.81	5.64	5.58	-0.14	1.00
PA4258_rplV_at	9.11	9.77	9.51	9.68	-0.15	1.00
PA4259_rpsS_at	6.83	7.55	7.24	7.30	-0.08	1.00
PA4260_rplB_at	9.40	10.04	9.76	9.78	-0.05	1.00
PA4261_rplW_at	7.62	8.11	8.04	7.91	-0.11	1.00
PA4262_rplD_at	7.24	8.40	8.00	8.05	-0.20	1.00
PA4263_rplC_at	6.66	8.05	7.62	7.89	-0.40	1.00
PA4264_rpsJ_at	6.67	7.85	7.35	7.29	-0.06	1.00
PA4265_tufA_s_at	8.13	8.42	8.33	8.33	-0.06	1.00
PA4269_rpoC_at	6.96	7.94	7.45	7.73	-0.14	1.00
PA4270_rpoB_at	6.88	7.30	7.02	7.01	0.08	1.00
PA4271_rplL_at	8.86	9.40	9.16	9.29	-0.09	1.00
PA4272_rplJ_at	7.72	8.93	8.62	8.68	-0.32	1.00
PA4273_rplA_at	7.52	8.61	8.26	8.39	-0.26	1.00
PA4274_rplK_at	8.55	9.42	9.17	9.38	-0.29	1.00
PA4275_nusG_at	5.80	6.50	6.08	5.86	0.18	1.00
PA4278_at	3.03	3.03	3.04	3.14	-0.06	1.00
PA4279_at	5.21	5.31	5.36	5.26	-0.05	1.00
PA4280_birA_at	3.78	3.93	4.14	3.77	-0.09	1.00
PA4284_recB_at	4.60	4.48	4.66	4.50	-0.05	1.00
PA4286_at	4.99	5.15	5.10	5.20	-0.08	1.00
PA4287_at	3.24	2.91	2.99	2.84	0.16	1.00
PA4291_at	4.98	5.29	5.11	5.31	-0.07	1.00
PA4292_at	2.99	3.14	3.21	3.21	-0.14	1.00
PA4293_at	2.57	2.53	2.75	2.57	-0.11	1.00
PA4294_at	4.55	4.09	4.58	4.37	-0.15	1.00
PA4295_at	2.34	2.22	2.30	2.20	0.03	1.00
PA4297_at	2.95	2.80	2.95	3.08	-0.14	1.00

PA4298_at	2.88	2.62	2.74	2.55	0.11	1.00
PA4301_at	4.20	4.14	4.01	4.14	0.10	1.00
PA4302_at	2.58	2.37	2.43	2.40	0.05	1.00
PA4303_at	3.47	3.09	3.40	3.48	-0.16	1.00
PA4304_at	2.61	2.52	2.50	2.34	0.15	1.00
PA4306_at	2.54	2.38	2.61	2.38	-0.03	1.00
PA4307_pctC_at	3.10	2.97	3.03	3.27	-0.11	1.00
PA4308_at	5.02	4.95	5.08	5.01	-0.06	1.00
PA4310_pctB_at	4.25	5.32	4.29	4.82	0.23	1.00
PA4312_at	3.08	3.29	3.52	3.20	-0.17	1.00
PA4313_at	3.78	3.61	3.80	3.42	0.09	1.00
PA4314_purU1_at	4.53	4.92	4.82	4.81	-0.09	1.00
PA4316_sbcB_at	2.85	3.51	2.98	3.15	0.12	1.00
PA4318_at	4.76	5.24	4.73	5.11	0.08	1.00
PA4319_at	2.91	2.98	2.83	2.82	0.12	1.00
PA4321_at	4.82	4.89	4.87	4.91	-0.04	1.00
PA4322_at	4.07	4.20	4.54	3.99	-0.13	1.00
PA4323_at	3.08	3.31	3.08	3.26	0.02	1.00
PA4324_at	4.49	4.65	4.48	4.40	0.13	1.00
PA4325_at	5.30	5.39	5.37	5.36	-0.02	1.00
PA4327_at	3.51	2.95	3.14	3.02	0.15	1.00
PA4331_at	3.85	3.45	3.65	3.53	0.06	1.00
PA4333_at	3.37	4.08	4.11	3.68	-0.17	1.00
PA4334_at	3.87	3.65	3.64	3.58	0.15	1.00
PA4335_at	2.16	2.30	2.37	2.34	-0.12	1.00
PA4339_at	2.93	2.80	2.92	2.58	0.12	1.00
PA4343_at	4.20	3.74	3.99	3.89	0.03	1.00
PA4346_at	3.23	3.22	3.34	3.25	-0.08	1.00
PA4347_at	4.13	4.26	3.98	4.17	0.12	1.00
PA4348_at	6.09	6.11	6.00	5.96	0.13	1.00
PA4350_at	3.14	2.97	2.88	3.03	0.10	1.00
PA4351_at	2.65	2.44	2.69	2.46	-0.03	1.00
PA4355_at	5.18	4.85	5.22	4.95	-0.07	1.00
PA4356_xenB_at	3.39	3.10	3.45	3.33	-0.15	1.00
PA4357_r_at	4.90	4.35	4.43	4.74	0.05	1.00
PA4358_at	3.54	3.71	3.67	3.51	0.04	1.00
PA4359_i_at	3.93	3.67	3.80	3.95	-0.08	1.00
PA4361_at	2.90	2.94	2.68	2.95	0.11	1.00
PA4363_iciA_at	2.94	2.76	2.84	2.71	0.08	1.00
PA4365_at	2.94	2.82	3.04	2.86	-0.07	1.00
PA4366_sodB_at	5.69	5.92	5.84	6.07	-0.15	1.00
PA4367_at	3.05	2.87	2.94	2.83	0.07	1.00
PA4368_at	3.19	3.22	3.21	3.26	-0.03	1.00
PA4369_at	3.47	3.28	3.28	3.35	0.06	1.00

PA4372_at	4.70	4.50	4.69	4.66	-0.07	1.00
PA4373_at	4.12	3.79	3.79	3.91	0.11	1.00
PA4374_at	3.60	3.62	3.58	3.60	0.02	1.00
PA4376_pncB2_at	3.55	3.60	3.53	3.50	0.06	1.00
PA4379_at	4.53	5.41	4.73	4.81	0.20	1.00
PA4380_at	3.43	3.40	3.43	3.60	-0.10	1.00
PA4381_at	4.04	4.08	4.08	4.08	-0.02	1.00
PA4382_at	2.74	2.75	2.57	2.82	0.05	1.00
PA4384_at	4.87	4.41	4.88	4.66	-0.13	1.00
PA4386_groES_at	9.36	10.08	9.99	9.97	-0.26	1.00
PA4388_at	4.17	4.15	4.15	4.13	0.02	1.00
PA4390_at	2.96	2.84	2.96	2.97	-0.07	1.00
PA4391_at	4.35	4.19	4.51	4.17	-0.07	1.00
PA4392_at	4.08	3.70	4.15	3.99	-0.18	1.00
PA4393_at	3.83	3.63	3.63	3.54	0.14	1.00
PA4396_at	3.12	3.14	3.03	3.01	0.11	1.00
PA4399_at	3.59	3.06	3.17	3.12	0.18	1.00
PA4401_at	3.31	3.24	3.32	3.38	-0.08	1.00
PA4402_argJ_at	4.93	4.56	4.86	4.98	-0.18	1.00
PA4403_secA_at	4.35	4.76	4.19	4.54	0.19	1.00
PA4404_at	3.94	3.92	4.25	3.92	-0.15	1.00
PA4406_lpxC_at	4.70	5.37	4.94	5.06	0.04	1.00
PA4407_ftsZ_at	6.23	6.74	6.67	6.61	-0.15	1.00
PA4408_ftsA_at	3.72	4.11	3.95	3.62	0.13	1.00
PA4409_ftsQ_at	5.45	5.80	5.76	5.42	0.03	1.00
PA4410_ddlB_at	4.67	4.45	4.42	4.58	0.06	1.00
PA4412_murG_at	4.98	5.17	4.95	5.29	-0.04	1.00
PA4413_ftsW_at	4.61	4.66	4.53	4.53	0.10	1.00
PA4414_murD_at	5.27	5.18	5.25	5.34	-0.07	1.00
PA4415_mraY_at	4.67	5.20	4.74	4.87	0.14	1.00
PA4417_murE_at	4.47	4.62	4.49	4.74	-0.07	1.00
PA4418_ftsI_at	5.50	5.21	5.12	5.27	0.16	1.00
PA4419_ftsL_at	3.20	3.48	3.11	3.32	0.12	1.00
PA4423_at	3.41	3.33	3.29	3.27	0.09	1.00
PA4425_at	4.95	5.46	4.88	5.30	0.11	1.00
PA4427_sspB_at	4.15	4.10	4.22	4.28	-0.13	1.00
PA4428_sspA_at	3.18	4.08	3.61	3.86	-0.11	1.00
PA4429_at	6.12	6.38	6.19	6.78	-0.24	1.00
PA4431_at	7.14	8.22	8.01	8.03	-0.34	1.00
PA4432_rpsI_at	6.24	7.03	6.40	6.61	0.13	1.00
PA4433_rplM_at	4.99	6.19	5.82	5.98	-0.31	1.00
PA4434_at	3.81	3.81	3.89	3.95	-0.11	1.00
PA4435_at	3.40	3.11	3.34	3.25	-0.04	1.00
PA4439_trpS_at	4.93	4.68	4.86	5.03	-0.14	1.00

PA4441_at	5.92	6.45	5.84	6.39	0.07	1.00
PA4442_cysN_at	4.21	3.73	4.02	4.12	-0.10	1.00
PA4445_at	2.78	3.09	2.94	2.88	0.02	1.00
PA4446_algW_at	2.78	2.63	2.63	2.69	0.04	1.00
PA4447_hisC1_at	3.99	3.99	4.08	3.97	-0.03	1.00
PA4449_hisG_at	3.76	3.88	3.76	3.76	0.06	1.00
PA4452_at	4.44	4.32	4.42	4.52	-0.09	1.00
PA4453_at	5.22	5.46	5.11	5.25	0.16	1.00
PA4454_at	4.94	4.80	4.87	4.57	0.15	1.00
PA4455_at	3.77	4.02	3.64	3.83	0.16	1.00
PA4458_at	5.01	5.57	4.98	5.22	0.19	1.00
PA4459_at	3.82	4.38	3.98	3.95	0.13	1.00
PA4460_at	5.52	5.84	5.60	5.49	0.14	1.00
PA4463_at	8.38	8.39	8.56	8.26	-0.03	1.00
PA4464_ptsN_at	7.54	8.10	7.62	7.66	0.18	1.00
PA4466_at	4.09	4.47	4.08	4.15	0.17	1.00
PA4469_at	2.74	2.74	2.84	2.57	0.03	1.00
PA4471_at	2.28	2.32	2.20	2.26	0.07	1.00
PA4472_pmbA_at	5.21	5.38	5.09	5.22	0.14	1.00
PA4473_at	3.99	4.45	4.15	3.91	0.19	1.00
PA4474_at	3.81	4.64	4.05	4.00	0.20	1.00
PA4482_gatC_at	4.89	5.50	5.16	5.09	0.07	1.00
PA4483_gatA_at	3.51	3.42	3.42	3.57	-0.03	1.00
PA4484_gatB_at	5.72	5.91	5.68	5.90	0.02	1.00
PA4486_at	4.09	3.84	3.77	3.85	0.16	1.00
PA4488_at	4.10	3.85	4.07	3.94	-0.03	1.00
PA4489_at	4.01	3.51	3.90	3.78	-0.08	1.00
PA4490_at	4.63	4.56	4.35	4.56	0.14	1.00
PA4492_at	4.37	4.36	4.39	4.19	0.07	1.00
PA4495_at	7.23	7.64	7.39	7.32	0.08	1.00
PA4496_at	5.31	4.78	4.90	4.77	0.21	1.00
PA4497_at	3.08	2.71	2.87	2.98	-0.03	1.00
PA4498_at	3.27	3.13	3.31	3.25	-0.08	1.00
PA4500_at	3.08	3.12	3.22	3.21	-0.11	1.00
PA4501_at	3.46	3.25	3.45	3.57	-0.15	1.00
PA4502_at	3.12	3.25	3.18	3.48	-0.14	1.00
PA4508_at	3.01	2.83	3.08	2.98	-0.11	1.00
PA4510_at	3.46	3.55	3.39	3.70	-0.04	1.00
PA4511_at	5.28	5.10	5.15	4.98	0.13	1.00
PA4512_at	3.22	3.16	3.22	3.28	-0.06	1.00
PA4515_at	2.69	2.70	2.60	2.52	0.14	1.00
PA4516_at	3.16	3.15	3.31	3.23	-0.12	1.00
PA4518_at	2.34	2.33	2.47	2.47	-0.14	1.00
PA4528_pilD_at	4.98	5.72	5.64	5.46	-0.20	1.00

PA4529_at	5.85	6.11	6.06	6.13	-0.11	1.00
PA4530_at	5.37	6.00	5.76	5.75	-0.07	1.00
PA4532_at	4.65	4.79	4.66	4.87	-0.05	1.00
PA4533_at	3.92	3.70	3.80	3.47	0.18	1.00
PA4536_at	4.83	4.92	4.75	4.88	0.06	1.00
PA4540_at	4.06	3.84	3.80	3.91	0.10	1.00
PA4541_at	3.97	3.94	4.16	3.67	0.04	1.00
PA4542_clpB_at	6.55	6.73	6.75	6.66	-0.06	1.00
PA4543_at	2.82	2.73	2.77	2.60	0.09	1.00
PA4544_rluD_at	3.70	3.75	3.72	3.52	0.10	1.00
PA4546_pilS_at	3.04	3.11	3.13	3.28	-0.13	1.00
PA4547_pilR_at	4.33	4.16	4.29	4.44	-0.13	1.00
PA4548_at	5.19	5.40	5.32	5.16	0.06	1.00
PA4549_fimT_at	3.45	3.33	3.56	3.47	-0.12	1.00
PA4550_fimU_at	2.79	3.04	2.87	3.09	-0.06	1.00
PA4557_lytB_at	3.67	3.64	3.64	3.75	-0.04	1.00
PA4558_at	3.82	4.27	4.05	4.29	-0.13	1.00
PA4559_lspA_at	3.78	4.04	3.92	3.79	0.06	1.00
PA4560_ileS_at	3.43	3.71	3.62	3.67	-0.08	1.00
PA4561_ribF_at	5.66	5.73	5.59	5.92	-0.06	1.00
PA4562_at	2.92	3.02	2.80	2.92	0.11	1.00
PA4565_proB_at	4.84	5.69	5.09	5.17	0.13	1.00
PA4566_obg_at	4.55	5.88	5.25	5.05	0.07	1.00
PA4567_rpmA_at	5.86	6.94	6.54	7.00	-0.37	1.00
PA4568_rplU_at	7.54	8.22	7.61	7.85	0.15	1.00
PA4570_at	3.36	3.14	3.29	3.28	-0.04	1.00
PA4572_fklB_at	5.29	5.53	5.48	5.45	-0.06	1.00
PA4573_at	4.06	3.53	3.63	3.66	0.15	1.00
PA4575_at	6.56	6.64	6.53	6.62	0.03	1.00
PA4579_at	3.58	3.59	3.58	3.53	0.03	1.00
PA4582_at	4.25	4.09	4.18	3.93	0.12	1.00
PA4585_rtcA_at	3.47	3.41	3.41	3.33	0.07	1.00
PA4586_at	3.56	3.23	3.43	3.55	-0.09	1.00
PA4588_gdhA_at	4.11	4.13	4.14	3.99	0.06	1.00
PA4589_at	3.18	2.83	3.15	3.14	-0.13	1.00
PA4590_pra_at	3.11	2.75	2.88	3.27	-0.14	1.00
PA4592_at	3.61	3.24	3.49	3.27	0.04	1.00
PA4593_at	3.72	3.37	3.55	3.33	0.10	1.00
PA4595_at	5.28	5.63	5.56	5.60	-0.12	1.00
PA4600_nfxB_at	2.98	2.74	2.91	2.96	-0.08	1.00
PA4601_at	3.30	3.39	3.56	3.30	-0.08	1.00
PA4603_at	3.54	3.15	3.57	3.23	-0.06	1.00
PA4604_at	2.77	2.84	2.81	2.71	0.05	1.00
PA4608_at	7.70	7.67	7.78	7.85	-0.13	1.00

PA4609_radA_at	3.31	3.35	3.39	3.45	-0.09	1.00
PA4610_at	6.32	6.78	6.63	6.80	-0.17	1.00
PA4612_at	2.52	2.55	2.40	2.47	0.10	1.00
PA4613_katB_at	3.04	3.29	3.11	3.08	0.07	1.00
PA4616_at	3.76	3.99	3.65	3.91	0.09	1.00
PA4617_at	3.35	3.05	3.15	3.37	-0.06	1.00
PA4619_at	2.55	2.60	2.70	2.69	-0.12	1.00
PA4623_r_at	4.86	4.55	4.63	4.48	0.15	1.00
PA4624_at	3.13	3.13	3.19	2.98	0.05	1.00
PA4625_at	3.06	3.08	3.24	2.96	-0.03	1.00
PA4626_hprA_at	4.26	4.32	4.44	3.93	0.10	1.00
PA4627_at	4.00	4.02	4.09	3.76	0.08	1.00
PA4628_lysP_at	2.37	2.34	2.23	2.40	0.04	1.00
PA4632_at	5.50	5.87	5.81	5.71	-0.07	1.00
PA4633_at	4.81	5.23	4.80	5.02	0.11	1.00
PA4635_at	2.78	3.09	2.80	2.77	0.15	1.00
PA4637_i_at	3.18	3.39	3.14	3.31	0.06	1.00
PA4638_at	3.16	2.69	2.91	3.05	-0.05	1.00
PA4639_at	5.99	5.96	5.99	6.10	-0.07	1.00
PA4640_mqoB_at	5.54	6.08	5.67	5.71	0.12	1.00
PA4643_at	4.10	3.92	3.79	4.11	0.06	1.00
PA4645_at	4.72	4.96	4.66	4.94	0.04	1.00
PA4646_upp_at	3.37	3.85	3.46	3.56	0.10	1.00
PA4647_uraA_at	2.76	2.62	2.70	2.48	0.10	1.00
PA4648_at	4.33	3.90	4.05	4.45	-0.14	1.00
PA4649_at	3.39	3.28	3.33	3.69	-0.17	1.00
PA4650_at	2.71	2.52	2.76	2.76	-0.14	1.00
PA4652_at	2.65	2.68	2.67	2.72	-0.03	1.00
PA4653_at	2.48	2.59	2.59	2.72	-0.12	1.00
PA4654_at	2.59	2.70	2.52	2.88	-0.06	1.00
PA4655_hemH_at	4.09	4.44	4.05	4.30	0.09	1.00
PA4656_at	3.13	3.56	3.40	3.24	0.03	1.00
PA4657_at	5.39	5.83	5.74	5.74	-0.13	1.00
PA4658_at	5.08	5.79	5.26	5.34	0.13	1.00
PA4659_at	3.08	2.90	3.21	2.96	-0.10	1.00
PA4660_phr_at	4.43	4.24	4.14	4.29	0.12	1.00
PA4661_at	5.53	6.03	5.91	6.06	-0.20	1.00
PA4662_murI_at	3.03	3.45	3.19	3.16	0.07	1.00
PA4663_moeB_at	3.12	3.12	3.05	3.02	0.09	1.00
PA4664_hemK_at	3.28	3.23	3.36	3.19	-0.02	1.00
PA4665_prfA_at	4.17	4.88	4.05	4.43	0.29	1.00
PA4666_hemA_at	3.59	3.76	3.59	3.62	0.07	1.00
PA4669_ipk_at	3.27	3.32	3.28	3.24	0.03	1.00
PA4670_prs_at	5.16	6.34	5.30	5.49	0.36	1.00

PA4671_at	6.58	7.53	6.98	7.25	-0.06	1.00
PA4673_at	5.70	6.23	5.79	5.74	0.20	1.00
PA4674_at	3.51	3.33	3.22	3.43	0.10	1.00
PA4676_at	2.77	3.24	3.06	3.03	-0.04	1.00
PA4677_at	4.35	4.19	4.13	4.27	0.07	1.00
PA4678_rimI_at	2.78	2.97	2.73	2.76	0.13	1.00
PA4679_at	4.94	5.16	5.03	5.17	-0.05	1.00
PA4680_at	2.61	2.56	2.72	2.51	-0.03	1.00
PA4683_at	3.60	3.56	3.87	3.46	-0.08	1.00
PA4684_at	2.84	2.78	2.75	3.09	-0.10	1.00
PA4685_at	2.64	2.28	2.47	2.21	0.12	1.00
PA4686_at	4.22	4.12	3.99	4.23	0.06	1.00
PA4687_hitA_at	5.03	5.00	5.19	4.66	0.09	1.00
PA4688_hitB_at	4.52	4.19	4.34	4.31	0.03	1.00
PA4689_at	3.42	3.61	3.42	3.70	-0.04	1.00
PA4690_at	3.23	3.15	3.13	3.11	0.07	1.00
PA4696_ilvI_at	5.01	5.52	5.06	5.30	0.09	1.00
PA4697_at	7.25	7.18	7.13	7.47	-0.08	1.00
PA4699_at	4.29	4.26	4.29	4.23	0.02	1.00
PA4705_at	2.93	2.95	2.84	3.15	-0.05	1.00
PA4706_at	3.22	3.15	3.19	3.31	-0.07	1.00
PA4708_at	4.23	4.36	4.23	4.40	-0.02	1.00
PA4709_at	3.32	3.50	3.35	3.35	0.06	1.00
PA4710_at	3.71	3.75	3.98	3.65	-0.09	1.00
PA4711_at	3.77	3.34	3.61	3.73	-0.11	1.00
PA4713_at	3.35	3.64	3.62	3.54	-0.08	1.00
PA4714_at	3.25	3.06	3.07	3.30	-0.04	1.00
PA4715_at	3.17	2.86	3.27	3.04	-0.14	1.00
PA4717_at	4.10	4.44	4.22	4.25	0.04	1.00
PA4719_at	2.88	2.84	3.09	2.81	-0.09	1.00
PA4720_trmA_at	3.82	3.70	3.71	3.57	0.12	1.00
PA4722_at	3.50	3.65	3.37	3.49	0.14	1.00
PA4724_at	3.95	3.97	4.04	4.12	-0.12	1.00
PA4725_at	3.74	3.52	3.64	3.74	-0.06	1.00
PA4729_panB_at	6.18	6.51	6.23	6.26	0.10	1.00
PA4730_panC_at	4.47	4.69	4.32	4.69	0.07	1.00
PA4732_pgi_at	4.76	5.11	4.72	5.02	0.06	1.00
PA4734_at	3.47	3.93	3.37	3.76	0.13	1.00
PA4735_at	3.47	3.76	3.44	3.52	0.13	1.00
PA4736_at	3.83	4.12	3.84	3.91	0.10	1.00
PA4737_at	3.18	3.78	3.56	3.51	-0.05	1.00
PA4741_rpsO_at	4.68	5.02	4.55	4.83	0.16	1.00
PA4744_infB_at	5.19	5.53	5.11	5.32	0.14	1.00
PA4745_nusA_at	5.85	6.49	6.11	6.07	0.08	1.00

PA4746_at	5.99	6.39	6.11	6.17	0.05	1.00
PA4748_tpiA_at	3.46	4.38	3.73	3.66	0.23	1.00
PA4752_ftsJ_at	2.96	3.00	2.89	2.93	0.07	1.00
PA4753_at	4.31	4.77	4.30	4.54	0.12	1.00
PA4757_at	3.49	4.01	3.86	3.91	-0.13	1.00
PA4759_dapB_at	3.04	3.14	3.00	2.95	0.12	1.00
PA4760_dnaJ_at	5.97	6.08	5.95	6.46	-0.18	1.00
PA4761_dnaK_at	7.64	7.96	7.92	7.94	-0.13	1.00
PA4762_grpE_at	7.49	8.52	7.62	7.74	0.32	1.00
PA4764_fur_at	5.27	5.01	5.40	5.21	-0.16	1.00
PA4766_at	3.01	2.66	2.87	2.60	0.10	1.00
PA4767_at	4.73	4.45	4.62	4.84	-0.14	1.00
PA4768_smpB_at	3.74	3.66	3.86	3.64	-0.06	1.00
PA4770_lldP_at	2.50	3.01	2.98	2.90	-0.19	1.00
PA4771_lldD_at	2.97	2.42	2.79	2.68	-0.04	1.00
PA4775_at	3.62	3.34	3.56	3.54	-0.07	1.00
PA4780_at	5.22	5.20	5.37	5.20	-0.08	1.00
PA4782_at	3.19	3.45	3.15	3.21	0.14	1.00
PA4786_at	4.52	4.44	4.68	4.47	-0.10	1.00
PA4788_at	2.91	3.04	2.75	3.00	0.10	1.00
PA4789_at	4.46	4.45	4.28	4.44	0.10	1.00
PA4790_at	3.48	3.41	3.53	3.54	-0.09	1.00
PA4792_at	5.34	5.25	5.23	5.31	0.02	1.00
PA4793_at	5.17	5.06	4.89	5.14	0.10	1.00
PA4794_at	3.09	3.16	3.11	3.09	0.03	1.00
PA4795_at	5.53	5.40	5.52	5.46	-0.02	1.00
PA4796_at	4.69	4.39	4.60	4.56	-0.04	1.00
PA4798_at	3.09	3.04	2.96	3.11	0.03	1.00
PA4799_at	2.92	2.62	2.97	2.74	-0.08	1.00
PA4800_at	2.37	2.39	2.44	2.43	-0.06	1.00
PA4803_at	4.41	4.04	4.12	4.00	0.16	1.00
PA4804_at	2.93	2.66	2.80	2.73	0.03	1.00
PA4805_at	2.81	2.86	2.97	2.90	-0.10	1.00
PA4808_selA_at	3.16	3.34	3.24	3.36	-0.05	1.00
PA4809_fdhE_at	4.59	4.27	4.15	4.59	0.06	1.00
PA4812_fdnG_at	3.57	4.14	3.74	3.58	0.20	1.00
PA4813_lipC_at	2.68	2.33	2.38	2.37	0.13	1.00
PA4814_fadH2_at	2.86	2.45	2.80	2.62	-0.05	1.00
PA4817_at	3.06	3.07	2.93	2.96	0.11	1.00
PA4819_at	3.48	3.35	3.47	3.16	0.10	1.00
PA4820_at	2.80	2.66	2.64	2.63	0.09	1.00
PA4824_at	2.96	2.90	3.05	3.01	-0.10	1.00
PA4825_mgtA_at	2.59	2.65	2.83	2.62	-0.11	1.00
PA4826_at	3.95	4.84	4.24	4.07	0.24	1.00

PA4827_at	3.44	3.48	3.56	3.61	-0.13	1.00
PA4828_at	3.24	3.28	3.52	3.27	-0.13	1.00
PA4829_lpd3_at	3.31	3.49	3.34	3.24	0.11	1.00
PA4830_at	3.79	3.81	3.81	3.83	-0.02	1.00
PA4832_at	3.10	2.70	2.78	2.68	0.17	1.00
PA4833_at	3.49	3.16	3.09	3.37	0.10	1.00
PA4834_at	2.70	2.45	2.34	2.63	0.09	1.00
PA4836_at	2.64	2.78	2.62	2.68	0.06	1.00
PA4837_at	4.86	4.95	4.57	4.95	0.14	1.00
PA4838_at	3.52	3.39	3.51	3.34	0.03	1.00
PA4839_speA_at	2.99	3.23	2.97	3.07	0.09	1.00
PA4843_at	4.52	4.70	4.51	4.66	0.02	1.00
PA4844_at	3.28	3.02	3.29	3.11	-0.05	1.00
PA4845_dipZ_at	3.64	4.24	3.61	3.81	0.23	1.00
PA4846_aroQ1_at	5.13	5.56	5.03	5.27	0.19	1.00
PA4847_accB_at	6.89	7.21	6.99	7.04	0.03	1.00
PA4848_accC_at	5.69	6.01	5.74	5.81	0.07	1.00
PA4849_at	3.46	3.39	3.38	3.22	0.12	1.00
PA4850_prmA_at	4.20	4.59	4.27	4.82	-0.15	1.00
PA4852_at	6.86	7.12	6.88	6.82	0.14	1.00
PA4854_purH_at	3.71	3.96	3.88	4.05	-0.13	1.00
PA4855_purD_at	3.94	4.29	4.07	4.39	-0.12	1.00
PA4856_at	5.23	5.33	5.09	5.38	0.04	1.00
PA4858_at	2.42	2.19	2.36	2.38	-0.06	1.00
PA4859_at	4.43	4.06	4.34	4.25	-0.05	1.00
PA4861_at	3.39	3.28	3.37	3.43	-0.07	1.00
PA4862_at	3.61	3.44	3.71	3.45	-0.05	1.00
PA4864_ureD_at	2.72	2.53	2.73	2.61	-0.04	1.00
PA4870_at	4.69	4.79	4.86	4.44	0.09	1.00
PA4873_at	3.55	3.70	3.64	3.88	-0.14	1.00
PA4874_at	4.80	5.55	5.53	5.21	-0.19	1.00
PA4875_at	2.72	2.26	2.53	2.51	-0.03	1.00
PA4876_osmE_at	7.11	7.20	7.09	7.03	0.09	1.00
PA4882_at	3.56	3.43	3.70	3.46	-0.09	1.00
PA4883_at	3.85	3.30	3.56	3.78	-0.09	1.00
PA4884_at	3.22	2.79	3.12	3.00	-0.05	1.00
PA4885_irlR_at	3.17	3.43	3.29	3.20	0.05	1.00
PA4886_at	2.42	2.48	2.38	2.40	0.06	1.00
PA4887_at	4.50	4.32	4.64	4.30	-0.06	1.00
PA4888_at	2.96	2.75	2.96	2.87	-0.06	1.00
PA4889_at	3.47	3.40	3.64	3.52	-0.14	1.00
PA4891_ureE_at	3.86	3.50	3.83	3.80	-0.13	1.00
PA4894_i_at	2.77	2.90	3.05	2.79	-0.09	1.00
PA4897_at	3.14	3.00	3.17	3.18	-0.11	1.00

PA4900_at	3.21	2.96	3.03	3.23	-0.05	1.00
PA4904_vanA_at	2.80	2.76	2.80	2.87	-0.06	1.00
PA4905_vanB_at	3.07	2.54	2.89	2.62	0.05	1.00
PA4907_at	4.34	3.81	4.27	4.13	-0.12	1.00
PA4910_at	2.56	2.48	2.64	2.55	-0.08	1.00
PA4911_at	4.08	3.59	3.87	4.21	-0.20	1.00
PA4914_at	3.90	3.73	3.76	3.99	-0.06	1.00
PA4923_at	3.34	3.46	3.57	3.37	-0.07	1.00
PA4929_at	2.81	2.81	2.96	2.94	-0.13	1.00
PA4931_dnaB_at	2.76	2.80	2.69	2.82	0.02	1.00
PA4932_rplI_at	7.28	8.42	7.88	8.26	-0.22	1.00
PA4933_at	6.62	7.52	7.25	7.13	-0.12	1.00
PA4934_rpsR_at	5.55	7.62	6.92	6.92	-0.33	1.00
PA4935_rpsF_at	5.90	7.17	6.61	6.68	-0.11	1.00
PA4937_rnr_at	5.78	6.25	5.96	5.98	0.04	1.00
PA4938_purA_at	4.79	5.50	5.29	5.40	-0.20	1.00
PA4939_at	3.74	3.91	3.86	3.58	0.11	1.00
PA4940_at	3.78	4.89	4.03	4.32	0.16	1.00
PA4944_at	5.49	7.04	5.64	6.26	0.31	1.00
PA4946_mutL_at	3.31	3.21	3.46	3.34	-0.14	1.00
PA4947_amiB_at	4.70	5.15	4.74	4.92	0.10	1.00
PA4949_at	2.72	2.91	2.76	2.72	0.08	1.00
PA4954_motA_at	5.48	5.59	5.48	5.48	0.06	1.00
PA4956_rhdA_at	3.46	3.67	3.57	3.64	-0.04	1.00
PA4957_psd_at	4.23	4.34	4.39	4.33	-0.08	1.00
PA4959_at	4.21	4.33	4.48	4.35	-0.15	1.00
PA4961_at	3.62	3.62	3.42	3.57	0.12	1.00
PA4962_at	2.81	2.60	2.87	2.84	-0.15	1.00
PA4963_at	2.92	2.87	2.81	2.73	0.12	1.00
PA4964_parC_at	4.56	4.39	4.45	4.73	-0.11	1.00
PA4966_at	3.69	3.82	3.80	3.78	-0.04	1.00
PA4967_parE_at	4.10	3.98	4.19	4.03	-0.07	1.00
PA4968_at	3.41	3.44	3.31	3.49	0.03	1.00
PA4970_at	4.20	4.56	4.34	4.18	0.12	1.00
PA4971_at	4.37	3.99	4.38	4.28	-0.15	1.00
PA4973_thiC_at	4.55	4.08	4.34	4.17	0.06	1.00
PA4977_at	3.16	2.64	2.75	2.71	0.17	1.00
PA4980_at	4.01	3.66	3.69	3.72	0.13	1.00
PA4981_at	3.73	3.65	3.69	3.45	0.12	1.00
PA4982_at	2.92	2.88	2.72	2.87	0.10	1.00
PA4983_at	3.74	3.45	3.86	3.64	-0.16	1.00
PA4985_at	2.67	2.58	2.74	2.57	-0.03	1.00
PA4986_at	3.47	3.15	3.26	3.53	-0.08	1.00
PA4987_at	2.53	2.58	2.71	2.56	-0.07	1.00

PA4989_at	3.38	3.50	3.21	3.52	0.08	1.00
PA4990_at	2.63	2.27	2.30	2.52	0.04	1.00
PA4993_at	5.54	5.13	5.49	5.34	-0.09	1.00
PA4994_at	4.87	4.63	4.68	4.64	0.09	1.00
PA4995_at	3.87	3.59	3.37	3.71	0.19	1.00
PA4997_msbA_at	2.98	3.93	3.22	3.27	0.21	1.00
PA4998_at	4.33	4.94	4.69	4.67	-0.04	1.00
PA4999_at	3.30	3.10	3.26	3.18	-0.02	1.00
PA5000_at	4.48	4.48	4.26	4.48	0.11	1.00
PA5001_at	3.97	4.22	4.01	3.86	0.16	1.00
PA5002_at	5.27	5.15	5.19	5.31	-0.04	1.00
PA5003_at	3.90	3.81	3.79	3.80	0.06	1.00
PA5004_at	2.84	3.08	2.83	2.83	0.13	1.00
PA5006_at	3.01	3.08	3.13	3.05	-0.05	1.00
PA5007_at	3.66	3.71	3.69	3.73	-0.03	1.00
PA5008_at	2.93	3.04	2.96	2.91	0.05	1.00
PA5009_waaP_at	4.05	3.98	3.93	3.88	0.11	1.00
PA5010_waaG_at	6.12	6.32	6.20	6.21	0.02	1.00
PA5011_waaC_at	4.91	4.94	4.84	4.87	0.06	1.00
PA5013_ilvE_at	4.75	5.30	5.12	5.09	-0.08	1.00
PA5014_glnE_at	4.39	4.45	4.18	4.59	0.04	1.00
PA5016_aceF_at	6.63	6.85	6.83	6.96	-0.15	1.00
PA5018_msrA_at	5.46	5.59	5.56	5.62	-0.07	1.00
PA5020_at	3.94	3.13	3.63	3.67	-0.12	1.00
PA5023_at	3.63	4.00	3.91	3.83	-0.06	1.00
PA5025_metY_at	4.63	4.66	4.62	4.74	-0.03	1.00
PA5026_at	3.83	3.47	3.63	3.75	-0.04	1.00
PA5029_at	2.92	2.77	2.62	2.77	0.15	1.00
PA5031_at	3.38	3.26	3.30	3.17	0.08	1.00
PA5032_at	2.62	2.66	2.50	2.70	0.04	1.00
PA5034_hemE_at	3.35	3.36	3.40	3.35	-0.02	1.00
PA5035_gltD_at	3.41	3.39	3.34	3.31	0.07	1.00
PA5036_gltB_at	4.66	4.23	4.48	4.49	-0.04	1.00
PA5037_at	4.54	5.14	4.42	4.87	0.20	1.00
PA5038_aroB_at	3.78	3.69	3.51	3.70	0.13	1.00
PA5039_aroK_at	4.70	5.22	4.90	5.14	-0.06	1.00
PA5044_pilM_at	8.48	9.15	8.97	9.04	-0.19	1.00
PA5045_ponA_at	2.37	3.02	2.61	2.67	0.05	1.00
PA5046_at	6.74	7.24	7.03	7.21	-0.13	1.00
PA5047_at	5.61	5.47	5.43	5.49	0.08	1.00
PA5048_at	4.38	4.18	4.26	4.57	-0.14	1.00
PA5049_rpmE_at	3.47	3.53	3.23	3.48	0.15	1.00
PA5051_argS_at	3.69	4.39	3.86	4.07	0.07	1.00
PA5052_at	4.78	5.09	4.78	5.16	-0.04	1.00

PA5056_phaC1_at	3.00	2.91	2.80	3.01	0.06	1.00
PA5058_phaC2_at	2.49	2.65	2.92	2.48	-0.13	1.00
PA5060_phaF_at	7.44	7.81	7.60	7.51	0.07	1.00
PA5061_at	4.91	4.90	5.01	4.99	-0.09	1.00
PA5062_at	3.93	3.82	3.98	3.82	-0.02	1.00
PA5063_ubiE_at	4.15	4.10	3.93	4.49	-0.08	1.00
PA5064_at	3.51	3.91	3.91	3.81	-0.15	1.00
PA5065_at	3.74	3.92	3.98	3.76	-0.04	1.00
PA5066_hisI_at	6.37	6.61	6.40	6.53	0.02	1.00
PA5070_tatC_at	2.82	2.99	3.03	2.92	-0.07	1.00
PA5071_at	5.02	4.87	5.23	4.86	-0.10	1.00
PA5072_at	3.61	3.80	3.37	3.69	0.18	1.00
PA5073_at	4.48	4.13	4.30	4.24	0.04	1.00
PA5074_at	3.08	2.86	2.99	3.26	-0.15	1.00
PA5076_at	2.98	3.12	2.98	2.86	0.13	1.00
PA5077_mdoH_at	4.33	4.43	4.48	4.23	0.02	1.00
PA5078_at	5.10	5.42	5.10	5.24	0.09	1.00
PA5079_at	3.85	3.99	3.97	4.02	-0.07	1.00
PA5081_at	4.46	4.18	4.07	4.26	0.15	1.00
PA5082_at	2.82	3.10	3.51	2.84	-0.22	1.00
PA5083_at	2.53	2.48	2.56	2.58	-0.06	1.00
PA5084_at	2.50	2.48	2.79	2.50	-0.16	1.00
PA5085_at	4.91	5.00	4.93	4.87	0.06	1.00
PA5086_at	6.01	6.22	6.30	6.05	-0.06	1.00
PA5087_at	2.98	2.74	2.76	2.81	0.08	1.00
PA5088_at	2.57	2.64	2.51	2.61	0.04	1.00
PA5090_at	3.54	2.97	3.38	3.40	-0.14	1.00
PA5091_hutG_at	4.83	4.77	5.16	4.80	-0.18	1.00
PA5092_hutI_at	2.67	2.57	2.54	2.64	0.03	1.00
PA5094_at	3.49	3.44	3.83	3.39	-0.15	1.00
PA5096_at	3.31	2.83	3.09	2.88	0.08	1.00
PA5098_hutH_at	3.00	2.93	2.98	3.27	-0.16	1.00
PA5099_at	2.47	2.19	2.38	2.43	-0.07	1.00
PA5102_at	4.24	3.43	3.85	3.35	0.24	1.00
PA5103_at	3.60	3.59	3.67	3.65	-0.06	1.00
PA5104_at	4.11	4.20	4.10	4.13	0.04	1.00
PA5109_at	3.28	3.56	3.40	3.32	0.06	1.00
PA5110_fbp_at	3.68	3.79	3.59	3.70	0.09	1.00
PA5111_gloA3_at	4.96	5.34	5.19	5.26	-0.07	1.00
PA5113_at	4.09	4.20	4.37	3.97	-0.03	1.00
PA5114_at	4.16	4.12	3.95	4.08	0.12	1.00
PA5115_at	3.32	3.14	3.40	3.31	-0.12	1.00

PA5117_typA_at	4.07	4.43	4.08	4.22	0.09	1.00
PA5118_thiI_at	4.58	4.94	4.68	4.60	0.12	1.00
PA5119_glnA_at	6.41	7.15	6.79	6.97	-0.10	1.00
PA5121_at	3.61	3.24	3.35	3.21	0.14	1.00
PA5123_at	5.11	4.87	5.03	4.80	0.07	1.00
PA5125_ntrC_at	2.77	2.94	2.64	2.95	0.06	1.00
PA5126_at	4.40	4.11	4.14	4.06	0.16	1.00
PA5128_secB_at	4.30	4.49	4.36	4.46	-0.02	1.00
PA5131_pgm_at	4.78	5.37	4.81	5.25	0.04	1.00
PA5132_at	4.76	4.62	4.77	4.88	-0.14	1.00
PA5134_at	4.16	4.44	4.44	3.97	0.09	1.00
PA5135_at	4.22	4.35	4.14	4.26	0.09	1.00
PA5136_at	4.87	4.39	4.49	4.68	0.05	1.00
PA5139_at	4.37	4.46	4.70	4.43	-0.15	1.00
PA5140_hisF1_at	3.41	3.18	3.38	3.15	0.03	1.00
PA5141_hisA_at	3.48	3.65	3.55	3.50	0.04	1.00
PA5143_hisB_at	2.99	3.34	2.99	3.25	0.05	1.00
PA5144_i_at	3.33	2.83	3.02	2.95	0.09	1.00
PA5147_mutY_at	4.31	4.37	4.20	4.65	-0.09	1.00
PA5149_at	3.38	3.36	3.23	3.36	0.07	1.00
PA5150_at	3.04	2.74	2.80	2.77	0.11	1.00
PA5151_at	3.84	3.67	3.73	3.87	-0.04	1.00
PA5153_at	3.19	3.35	3.23	3.40	-0.04	1.00
PA5155_at	2.12	2.10	2.20	2.12	-0.05	1.00
PA5158_at	2.79	2.80	2.72	2.60	0.13	1.00
PA5159_at	2.72	2.69	2.79	2.81	-0.10	1.00
PA5163_rmlA_at	4.64	4.81	4.72	4.65	0.04	1.00
PA5166_at	3.65	3.65	3.39	3.68	0.12	1.00
PA5174_at	3.85	4.22	3.78	3.97	0.16	1.00
PA5176_at	4.02	3.84	3.90	3.90	0.03	1.00
PA5177_at	3.51	3.95	3.70	3.58	0.09	1.00
PA5183_at	2.76	2.66	2.76	2.86	-0.10	1.00
PA5184_at	6.07	5.88	6.06	5.99	-0.05	1.00
PA5185_at	2.95	2.82	2.80	2.88	0.04	1.00
PA5186_at	3.30	3.16	3.34	3.40	-0.14	1.00
PA5188_at	2.92	2.68	3.00	2.70	-0.05	1.00
PA5190_at	4.52	4.50	4.47	4.72	-0.09	1.00
PA5191_at	3.63	3.46	3.63	3.60	-0.07	1.00
PA5192_pckA_at	5.15	5.68	5.42	5.55	-0.07	1.00
PA5193_yrfI_at	5.34	5.37	5.17	5.34	0.10	1.00
PA5194_at	5.39	5.08	5.16	5.11	0.10	1.00
PA5196_at	3.24	4.04	3.28	3.53	0.24	1.00
PA5197_rimK_at	3.47	3.17	3.20	3.18	0.13	1.00
PA5198_at	3.36	3.22	3.39	3.42	-0.12	1.00

PA5199_envZ_at	4.96	4.38	4.66	4.75	-0.04	1.00
PA5202_at	2.98	2.79	2.90	3.03	-0.09	1.00
PA5205_at	2.59	2.44	2.38	2.48	0.09	1.00
PA5207_at	2.84	2.93	2.81	2.73	0.11	1.00
PA5216_at	4.14	4.09	4.16	4.24	-0.09	1.00
PA5217_at	5.11	4.55	4.85	4.58	0.12	1.00
PA5219_at	2.77	3.00	2.75	2.78	0.12	1.00
PA5221_at	3.21	3.11	3.00	3.04	0.14	1.00
PA5222_at	3.80	4.07	3.95	4.01	-0.04	1.00
PA5223_ubiH_at	3.53	3.66	3.59	3.36	0.13	1.00
PA5227_at	4.35	4.98	4.30	4.65	0.19	1.00
PA5228_at	5.00	5.06	4.89	4.90	0.13	1.00
PA5230_at	3.66	3.64	3.65	3.68	-0.02	1.00
PA5232_at	7.04	7.16	7.18	7.06	-0.02	1.00
PA5233_at	3.24	3.30	3.43	3.30	-0.10	1.00
PA5234_at	2.72	2.87	2.98	2.67	-0.03	1.00
PA5236_at	5.07	4.77	4.92	4.57	0.17	1.00
PA5237_at	3.69	3.83	3.51	3.91	0.05	1.00
PA5238_at	2.78	2.58	2.69	2.58	0.05	1.00
PA5242_ppk_at	4.45	4.38	4.33	4.37	0.07	1.00
PA5243_hemB_at	5.12	5.57	5.05	5.24	0.20	1.00
PA5244_at	2.74	2.72	2.67	2.64	0.08	1.00
PA5245_at	4.00	4.31	4.20	4.39	-0.14	1.00
PA5246_at	3.70	3.32	3.53	3.59	-0.05	1.00
PA5248_at	4.15	4.13	4.12	4.08	0.04	1.00
PA5249_at	3.91	3.89	3.98	3.77	0.03	1.00
PA5252_at	4.03	4.03	3.87	4.13	0.03	1.00
PA5253_algP_at	7.87	7.79	7.74	7.76	0.08	1.00
PA5257_at	3.94	3.68	3.74	3.73	0.08	1.00
PA5258_at	4.28	4.60	4.26	4.39	0.11	1.00
PA5259_hemD_at	3.57	4.01	3.94	3.80	-0.08	1.00
PA5260_hemC_at	3.81	4.74	3.85	4.06	0.32	1.00
PA5261_algR_at	5.05	5.70	5.37	5.48	-0.06	1.00
PA5262_algZ_at	4.65	4.81	4.74	4.56	0.08	1.00
PA5263_argH_at	3.81	3.80	3.93	3.91	-0.12	1.00
PA5265_at	3.49	3.08	3.21	3.24	0.07	1.00
PA5269_at	3.51	3.27	3.34	3.14	0.15	1.00
PA5271_at	4.56	4.44	4.58	4.62	-0.10	1.00
PA5272_cyaA_at	2.70	3.13	2.75	2.84	0.12	1.00
PA5273_at	3.16	3.20	3.02	3.08	0.13	1.00
PA5275_at	4.91	5.20	5.16	5.25	-0.15	1.00
PA5278_dapF_at	3.19	3.13	3.10	2.95	0.14	1.00
PA5280_sss_at	3.37	3.27	3.47	3.39	-0.11	1.00
PA5283_at	3.34	3.66	3.49	3.40	0.06	1.00

PA5285_at	6.38	7.16	6.87	6.94	-0.13	1.00
PA5287_amtB_at	3.42	3.62	3.42	3.48	0.08	1.00
PA5293_at	4.30	4.30	4.52	4.37	-0.14	1.00
PA5295_at	3.12	3.48	3.38	2.99	0.12	1.00
PA5296_rep_at	3.05	3.05	3.27	2.88	-0.02	1.00
PA5297_poxB_at	4.26	4.29	4.41	4.39	-0.13	1.00
PA5298_at	2.58	3.03	2.57	2.73	0.15	1.00
PA5299_at	3.25	3.17	3.32	3.29	-0.09	1.00
PA5300_cycB_at	5.61	6.24	5.90	6.26	-0.15	1.00
PA5301_at	5.93	6.79	6.03	6.21	0.24	1.00
PA5302_dadX_at	5.19	5.07	5.42	5.06	-0.11	1.00
PA5305_at	5.57	5.83	5.90	5.73	-0.12	1.00
PA5306_at	5.19	5.82	5.44	5.43	0.07	1.00
PA5308_lrp_at	4.09	4.12	4.07	4.01	0.07	1.00
PA5310_at	3.71	3.81	3.96	3.74	-0.09	1.00
PA5311_at	2.87	2.47	2.87	2.56	-0.04	1.00
PA5312_at	6.51	6.88	6.57	6.69	0.07	1.00
PA5313_at	3.79	4.93	4.01	4.07	0.32	1.00
PA5315_rpmG_at	4.20	5.08	4.33	4.37	0.29	1.00
PA5318_at	2.69	2.50	2.62	2.52	0.03	1.00
PA5319_radC_at	3.73	3.74	3.81	3.84	-0.09	1.00
PA5320_dfp_at	4.47	4.68	4.53	4.36	0.13	1.00
PA5321_dut_at	4.03	3.67	3.81	4.02	-0.07	1.00
PA5322_algC_at	5.77	5.83	5.83	5.84	-0.04	1.00
PA5323_argB_at	2.88	3.41	3.17	3.24	-0.06	1.00
PA5324_at	2.85	3.08	3.03	3.19	-0.15	1.00
PA5326_at	2.77	2.68	2.70	2.60	0.08	1.00
PA5327_at	3.91	3.68	3.97	3.76	-0.07	1.00
PA5331_pyrE_at	4.44	4.90	4.68	4.95	-0.15	1.00
PA5332_crc_at	5.90	6.50	5.96	6.23	0.10	1.00
PA5334_rph_at	4.50	4.77	4.71	4.72	-0.08	1.00
PA5336_gmk_at	3.89	4.48	3.91	4.09	0.19	1.00
PA5338_spoT_at	5.10	5.37	5.13	5.14	0.10	1.00
PA5339_at	5.28	5.29	5.17	5.16	0.13	1.00
PA5340_at	5.74	5.83	5.89	5.60	0.04	1.00
PA5341_at	3.15	2.93	2.85	2.95	0.14	1.00
PA5342_at	3.46	3.55	3.56	3.63	-0.09	1.00
PA5343_at	3.53	3.31	3.45	3.13	0.13	1.00
PA5344_at	5.28	5.38	5.03	5.33	0.15	1.00
PA5346_at	5.72	6.28	5.84	6.04	0.06	1.00
PA5349_at	4.39	4.53	4.63	4.45	-0.08	1.00
PA5350_r_at	6.88	6.58	6.96	6.73	-0.11	1.00
PA5352_at	2.99	2.66	3.11	2.72	-0.09	1.00
PA5354_glcE_at	4.01	3.73	3.73	3.92	0.05	1.00

PA5356_glcC_at	5.05	5.36	4.97	5.20	0.12	1.00
PA5357_at	3.52	3.69	3.70	3.60	-0.04	1.00
PA5358_ubiA_at	3.51	3.27	3.30	3.26	0.11	1.00
PA5359_at	4.81	4.58	4.87	4.74	-0.11	1.00
PA5361_phoR_at	3.01	2.97	3.01	3.02	-0.02	1.00
PA5362_at	3.11	3.12	3.11	2.91	0.11	1.00
PA5363_at	5.24	4.96	5.16	4.83	0.10	1.00
PA5366_pstB_at	2.56	2.59	2.58	2.71	-0.08	1.00
PA5371_at	4.88	4.98	4.88	5.04	-0.02	1.00
PA5373_betB_at	6.66	6.82	6.55	6.72	0.10	1.00
PA5375_betT1_at	3.62	3.41	3.40	3.41	0.11	1.00
PA5377_at	5.82	5.62	5.58	5.79	0.04	1.00
PA5378_at	2.73	2.82	2.92	2.76	-0.07	1.00
PA5379_sdaB_at	4.17	3.87	4.03	3.89	0.05	1.00
PA5383_at	4.49	3.23	3.93	3.91	-0.06	1.00
PA5385_at	2.68	2.60	2.63	2.60	0.03	1.00
PA5386_at	3.02	2.86	3.01	2.90	-0.02	1.00
PA5387_at	3.05	2.78	3.01	2.57	0.12	1.00
PA5391_at	2.65	2.24	2.69	2.38	-0.09	1.00
PA5392_at	2.74	2.36	2.44	2.45	0.10	1.00
PA5394_cls_at	3.33	3.29	3.35	3.35	-0.03	1.00
PA5395_at	2.81	2.77	3.10	2.73	-0.12	1.00
PA5396_at	2.85	2.64	2.99	2.74	-0.12	1.00
PA5398_at	2.85	2.73	2.73	3.00	-0.08	1.00
PA5401_at	3.03	2.61	3.08	2.74	-0.09	1.00
PA5402_at	3.12	2.71	2.87	3.11	-0.07	1.00
PA5403_at	2.64	2.72	2.58	2.73	0.02	1.00
PA5404_at	3.25	3.14	3.11	3.11	0.08	1.00
PA5405_i_at	3.52	3.16	3.34	3.28	0.03	1.00
PA5407_at	4.21	3.93	3.93	4.01	0.10	1.00
PA5408_at	4.11	4.07	3.97	4.04	0.08	1.00
PA5409_at	3.17	3.24	3.19	2.96	0.13	1.00
PA5411_at	3.11	2.69	3.07	3.00	-0.13	1.00
PA5414_at	4.36	5.30	4.81	4.61	0.12	1.00
PA5417_soxD_at	2.83	2.47	2.54	2.69	0.04	1.00
PA5418_soxA_at	2.83	2.29	2.68	2.79	-0.17	1.00
PA5419_soxG_at	3.80	3.54	3.74	3.51	0.04	1.00
PA5422_at	4.17	4.39	4.11	4.32	0.07	1.00
PA5424_at	3.95	4.89	4.42	4.05	0.18	1.00
PA5425_purK_at	3.71	3.49	3.49	3.86	-0.07	1.00
PA5426_purE_at	3.95	4.15	4.22	4.13	-0.13	1.00
PA5430_at	3.82	3.76	3.63	3.76	0.10	1.00
PA5432_at	4.07	4.08	3.89	4.04	0.11	1.00
PA5434_mtr_at	2.49	2.40	2.35	2.41	0.07	1.00

PA5435_at	3.68	4.14	3.83	4.41	-0.21	1.00
PA5439_at	4.30	5.16	4.54	4.69	0.11	1.00
PA5440_at	3.89	3.97	3.92	4.08	-0.07	1.00
PA5441_at	3.65	4.08	3.88	3.73	0.06	1.00
PA5442_at	3.54	3.33	3.63	3.29	-0.03	1.00
PA5443_uvrD_at	4.12	4.12	4.21	4.22	-0.10	1.00
PA5444_at	3.85	3.81	3.85	3.91	-0.05	1.00
PA5447_wbpZ_at	3.89	3.78	3.88	3.85	-0.04	1.00
PA5448_wbpY_at	3.14	2.97	3.05	2.98	0.04	1.00
PA5450_wzt_at	3.35	3.43	3.36	3.53	-0.06	1.00
PA5451_wzm_at	4.37	3.87	4.22	4.32	-0.15	1.00
PA5452_wbpW_at	2.97	2.84	2.93	2.85	0.02	1.00
PA5453_gmd_at	4.86	4.84	4.80	4.72	0.09	1.00
PA5455_at	3.77	3.80	3.98	3.79	-0.10	1.00
PA5456_at	4.03	3.48	3.89	3.91	-0.14	1.00
PA5457_at	3.33	3.23	3.35	3.28	-0.04	1.00
PA5459_at	3.80	3.34	3.68	3.37	0.04	1.00
PA5461_at	3.68	3.86	3.84	3.85	-0.08	1.00
PA5462_at	3.50	3.52	3.54	3.55	-0.04	1.00
PA5466_at	3.57	3.34	3.31	3.38	0.11	1.00
PA5467_at	2.60	2.51	2.46	2.49	0.07	1.00
PA5468_at	2.59	2.43	2.66	2.56	-0.10	1.00
PA5469_at	3.22	2.92	3.13	2.86	0.08	1.00
PA5471_at	5.03	5.97	5.12	5.26	0.31	1.00
PA5472_at	2.92	3.16	3.01	3.20	-0.07	1.00
PA5474_at	4.17	4.12	4.24	4.32	-0.13	1.00
PA5477_at	4.77	4.76	4.78	4.71	0.02	1.00
PA5479_gltP_at	3.06	3.45	3.06	3.36	0.05	1.00
PA5480_at	2.61	2.31	2.33	2.41	0.09	1.00
PA5481_at	4.95	4.50	4.69	4.53	0.11	1.00
PA5486_at	3.24	3.33	3.16	3.20	0.11	1.00
PA5487_at	4.04	4.83	4.22	4.47	0.09	1.00
PA5488_at	4.83	5.47	4.91	4.90	0.25	1.00
PA5491_at	5.43	6.05	5.74	5.85	-0.05	1.00
PA5492_at	5.46	5.77	5.46	5.68	0.04	1.00
PA5493_polA_at	3.69	3.48	3.38	3.57	0.11	1.00
PA5494_at	5.32	5.46	5.52	5.44	-0.09	1.00
PA5495_thrB_at	4.38	5.18	4.53	4.70	0.16	1.00
PA5496_at	4.36	4.28	4.51	4.40	-0.14	1.00
PA5497_at	3.82	4.03	4.08	3.99	-0.11	1.00
PA5498_at	5.57	5.38	5.28	5.42	0.13	1.00
PA5501_znuB_at	4.04	4.42	4.12	4.10	0.12	1.00
PA5502_at	4.50	4.23	4.13	4.33	0.14	1.00
PA5506_at	3.09	3.57	3.36	3.51	-0.10	1.00

PA5510_at	3.09	3.07	3.26	3.15	-0.12	1.00
PA5511_at	2.83	3.05	3.17	2.93	-0.11	1.00
PA5513_at	5.12	5.08	5.08	5.03	0.05	1.00
PA5514_at	2.81	2.63	2.77	2.90	-0.12	1.00
PA5518_at	3.96	3.94	4.01	3.85	0.02	1.00
PA5520_at	2.87	2.70	2.68	2.62	0.14	1.00
PA5521_at	2.79	3.00	2.96	2.74	0.05	1.00
PA5524_at	3.22	3.45	3.25	3.38	0.02	1.00
PA5525_at	2.76	2.99	2.81	2.80	0.07	1.00
PA5526_at	3.37	3.78	3.48	3.33	0.17	1.00
PA5529_at	4.70	4.35	4.45	4.21	0.19	1.00
PA5530_at	2.35	2.31	2.38	2.41	-0.06	1.00
PA5531_tonB_at	6.27	5.88	6.16	5.91	0.04	1.00
PA5532_at	2.71	2.81	3.03	2.71	-0.11	1.00
PA5534_at	3.40	3.08	3.29	3.40	-0.10	1.00
PA5535_at	3.49	3.37	3.47	3.55	-0.08	1.00
PA5537_at	3.45	3.32	3.42	3.19	0.08	1.00
PA5538_amiA_at	2.43	2.46	2.38	2.36	0.08	1.00
PA5539_at	3.37	3.00	3.23	3.00	0.07	1.00
PA5540_at	3.31	2.78	3.22	2.97	-0.05	1.00
PA5542_at	3.57	3.24	3.28	3.36	0.09	1.00
PA5545_at	5.77	5.89	5.92	5.99	-0.12	1.00
PA5546_at	6.95	6.93	6.97	7.22	-0.15	1.00
PA5548_at	3.61	3.03	3.54	3.33	-0.11	1.00
PA5549_glmS_at	4.37	4.26	3.96	4.36	0.15	1.00
PA5552_glmU_at	4.00	4.64	4.22	4.22	0.09	1.00
PA5553_atpC_at	3.91	4.04	4.05	4.18	-0.14	1.00
PA5555_atpG_at	7.02	7.65	7.48	7.40	-0.11	1.00
PA5556_atpA_at	6.14	7.24	6.89	7.12	-0.31	1.00
PA5558_atpF_at	7.54	8.44	8.05	8.38	-0.22	1.00
PA5559_atpE_at	8.68	9.23	9.04	9.21	-0.17	1.00
PA5560_atpB_at	5.53	5.58	5.52	5.51	0.04	1.00
PA5561_atpI_at	4.82	4.91	4.62	4.93	0.09	1.00
PA5565_gidA_at	4.16	4.92	4.46	4.34	0.14	1.00
PA5566_at	3.16	2.89	3.04	2.68	0.17	1.00
PA5567_at	3.68	3.96	3.80	3.74	0.05	1.00
PA5569_rnpA_at	6.97	7.53	6.96	7.25	0.15	1.00
PA5570_rpmH_at	5.06	5.80	5.21	5.29	0.18	1.00
Pae_16SrRNA_s_at	14.48	14.51	14.57	14.48	-0.03	1.00
Pae_23SrRNA_s_at	14.56	14.59	14.59	14.62	-0.03	1.00
Pae_AF027291cds_at	3.65	2.58	3.31	3.13	-0.11	1.00
Pae_AF035937cd	2.38	2.06	2.22	2.27	-0.03	1.00

s10_at						
Pae_AF035937cds11_at	2.91	2.76	2.94	2.94	-0.10	1.00
Pae_AF035937cds12_at	3.43	3.09	3.42	3.38	-0.13	1.00
Pae_AF035937cds13_at	2.68	2.30	2.61	2.75	-0.19	1.00
Pae_AF035937cds6_at	3.42	2.66	3.05	3.24	-0.10	1.00
Pae_AF035937cds7_at	2.70	2.42	2.86	2.62	-0.18	1.00
Pae_AF035937cds8_at	3.18	2.91	3.10	2.88	0.05	1.00
Pae_AF035937cds9_at	2.57	2.33	2.50	2.46	-0.03	1.00
Pae_AF080442cds1_at	3.24	2.83	3.24	3.16	-0.17	1.00
Pae_AF080442cds2_at	2.55	2.38	2.52	2.49	-0.04	1.00
Pae_AF133697cds_at	2.06	2.06	2.16	2.09	-0.07	1.00
Pae_AF133699cds2_at	3.32	2.71	3.18	3.01	-0.08	1.00
Pae_AF133699cds3_at	2.18	2.00	1.91	2.05	0.11	1.00
Pae_AF133699cds4_at	3.03	2.91	2.95	2.90	0.04	1.00
Pae_AF140629cds1_at	2.58	2.21	2.27	2.25	0.13	1.00
Pae_AF140629cds2_at	2.55	2.24	2.22	2.30	0.13	1.00
Pae_AF147795cds10_at	2.41	2.01	2.09	2.13	0.10	1.00
Pae_AF147795cds11_at	2.56	2.29	2.51	2.54	-0.10	1.00
Pae_AF147795cds3_at	2.41	2.27	2.46	2.43	-0.11	1.00
Pae_AF147795cds4_at	2.54	2.38	2.54	2.48	-0.05	1.00
Pae_AF147795cds5_at	2.25	2.40	2.27	2.43	-0.02	1.00
Pae_AF147795cds6_at	2.37	2.34	2.72	2.30	-0.16	1.00
Pae_AF147795cds7_at	2.64	2.27	2.45	2.58	-0.06	1.00
Pae_AF147795cds9_at	3.05	2.61	2.76	2.74	0.08	1.00
Pae_AF191564cds1_at	2.85	2.27	2.51	2.47	0.07	1.00
Pae_AF241171cd	2.72	2.57	2.69	2.68	-0.04	1.00

s10_at						
Pae_AF241171cds12_at	2.75	2.58	2.87	2.69	-0.11	1.00
Pae_AF241171cds13_at	2.42	2.11	2.20	2.38	-0.03	1.00
Pae_AF241171cds14_at	3.03	2.57	2.77	3.13	-0.15	1.00
Pae_AF241171cds15_at	3.55	3.06	3.50	3.42	-0.15	1.00
Pae_AF241171cds16_at	3.87	3.22	3.43	3.83	-0.08	1.00
Pae_AF241171cds18_at	2.46	2.53	2.58	2.50	-0.04	1.00
Pae_AF241171cds21_at	2.75	2.47	2.75	2.66	-0.09	1.00
Pae_AF241171cds22_at	2.76	2.55	2.80	2.66	-0.08	1.00
Pae_AF241171cds23_at	2.44	2.23	2.51	2.32	-0.08	1.00
Pae_AF241171cds25_at	3.29	3.00	3.17	3.04	0.04	1.00
Pae_AF241171cds26_at	4.05	3.47	3.70	3.68	0.07	1.00
Pae_AF241171cds27_at	2.72	2.28	2.47	2.38	0.07	1.00
Pae_AF241171cds30_at	2.88	2.65	2.71	2.68	0.07	1.00
Pae_AF241171cds31_at	2.31	2.07	2.26	2.29	-0.08	1.00
Pae_AF241171cds34_at	2.42	2.21	2.49	2.32	-0.09	1.00
Pae_AF241171cds35_at	3.43	2.91	3.40	3.35	-0.21	1.00
Pae_AF241171cds36_at	2.16	2.20	2.12	2.18	0.02	1.00
Pae_AF241171cds39_at	3.78	3.19	3.65	3.50	-0.09	1.00
Pae_AF241171cds4_at	2.53	2.49	2.59	2.51	-0.04	1.00
Pae_AF241171cds40_at	2.81	2.36	2.64	2.66	-0.07	1.00
Pae_AF241171cds41_at	3.06	2.93	3.03	2.90	0.03	1.00
Pae_AF241171cds45_at	2.47	2.19	2.23	2.28	0.07	1.00
Pae_AF241171cds47_at	2.41	2.36	2.46	2.47	-0.08	1.00
Pae_AF241171cds48_at	2.73	2.34	2.40	2.36	0.16	1.00
Pae_AF241171cd	2.02	1.90	2.08	2.10	-0.12	1.00

s49_i_at						
Pae_AF241171cds5_at	2.35	2.13	2.29	2.30	-0.05	1.00
Pae_AF241171cds50_i_at	2.14	2.09	2.19	2.11	-0.04	1.00
Pae_AF241171cds51_at	2.30	2.19	2.32	2.36	-0.10	1.00
Pae_AF241171cds6_at	3.13	2.77	2.88	3.09	-0.03	1.00
Pae_AF241171cds7_at	2.96	2.42	2.51	2.53	0.17	1.00
Pae_AF241171cds8_at	3.61	3.40	3.56	3.49	-0.02	1.00
Pae_AF241171cds9_at	4.30	3.76	4.11	4.26	-0.16	1.00
Pae_flgK_at	2.21	2.11	2.15	2.10	0.03	1.00
Pae_flgL_at	3.20	2.66	2.80	2.90	0.08	1.00
Pae_J05162cds_at	2.21	2.06	2.22	2.00	0.02	1.00
Pae_L06160cds_at	2.81	2.44	2.85	2.62	-0.11	1.00
Pae_L06161cds_at	2.50	2.20	2.33	2.30	0.03	1.00
Pae_L07945cds_at	2.23	2.03	2.23	2.17	-0.07	1.00
Pae_L37109cds1_at	2.81	2.60	2.72	2.62	0.04	1.00
Pae_L37109cds2_at	2.52	2.16	2.40	2.34	-0.02	1.00
Pae_L81176cds5_at	4.38	3.70	4.05	3.96	0.04	1.00
Pae_L81176cds6_at	2.62	2.54	2.77	2.54	-0.07	1.00
Pae_M14849cds_at	2.54	2.25	2.53	2.18	0.04	1.00
Pae_M21652cds_at	2.91	2.52	2.86	2.70	-0.07	1.00
Pae_M98270cds2_at	3.50	3.16	3.60	3.41	-0.17	1.00
Pae_M98270cds3_at	2.29	2.03	2.19	2.31	-0.09	1.00
Pae_orfA_vioA_at	2.74	2.39	2.69	2.65	-0.10	1.00
Pae_orfC_at	2.81	2.60	2.58	2.69	0.07	1.00
Pae_orfD_at	4.46	3.63	4.04	4.23	-0.09	1.00
Pae_orfE_at	2.48	2.46	2.69	2.44	-0.09	1.00
Pae_orfG_at	3.19	2.71	3.03	3.15	-0.13	1.00
Pae_orfL_at	3.15	2.82	2.98	2.91	0.04	1.00
Pae_tRNA_Asn_s_at	2.64	2.80	2.76	2.93	-0.12	1.00

Pae_tRNA_Gly_s_at	3.44	4.17	3.77	3.61	0.12	1.00
Pae_tRNA_His_f_at	3.62	3.38	3.56	3.64	-0.10	1.00
Pae_tRNA_Ile_f_at	3.14	4.00	3.46	3.38	0.16	1.00
Pae_tRNA_Leu_s_at	4.88	5.17	4.90	4.89	0.13	1.00
Pae_tRNA_Met_s_at	2.17	2.19	2.13	2.21	0.02	1.00
Pae_tRNA_Phe_f_at	5.23	4.56	4.69	4.91	0.10	1.00
Pae_tRNA_Pro_f_at	2.65	2.51	2.79	2.64	-0.13	1.00
Pae_tRNA_Thr_s_at	3.27	2.97	3.02	3.04	0.09	1.00
Pae_tRNA_Trp_f_at	6.27	6.68	6.34	6.54	0.03	1.00
Pae_tRNA_Tyr_s_at	2.51	2.86	2.54	2.49	0.17	1.00
Pae_U85514cds_at	2.87	2.64	2.66	2.79	0.03	1.00
Pae_U97065cds3_at	3.45	2.86	3.13	3.03	0.07	1.00
AFFX-Athal_actin_at	2.05	1.95	2.01	2.01	-0.01	1.00
AFFX-YEL018W_at	2.11	1.93	2.01	2.02	0.00	1.00
AFFX-YER148W_SPT15_at	2.52	2.16	2.44	2.25	0.00	1.00
AFFX-YFL039C_ACT1_at	2.27	2.07	2.25	2.11	-0.01	1.00
ig_1118158_1117390_at	2.72	2.31	2.53	2.49	0.00	1.00
ig_1254309_1255042_at	2.91	2.80	2.78	2.89	0.02	1.00
ig_1324793_1324109_at	2.10	2.22	2.19	2.13	0.00	1.00
ig_1427453_1428080_at	2.43	2.39	2.45	2.35	0.01	1.00
ig_2164548_2165213_at	3.58	3.04	3.21	3.45	-0.02	1.00
ig_223454_224101_at	2.89	2.84	2.87	2.86	0.00	1.00
ig_2902217_2901558_at	2.60	2.58	2.57	2.60	0.01	1.00
ig_297561_299081_at	2.92	2.68	2.74	2.82	0.02	1.00
ig_3087490_3088659_at	2.56	2.67	2.73	2.53	-0.01	1.00

ig_4294604_4297249_at	2.16	2.08	2.09	2.12	0.02	1.00
ig_4326394_4327696_at	2.92	2.76	2.82	2.83	0.01	1.00
ig_5087407_5086695_at	3.15	2.94	3.00	3.08	0.01	1.00
ig_5131427_5130767_at	2.73	2.53	2.62	2.64	0.00	1.00
ig_5309325_5308424_at	2.79	2.60	2.67	2.70	0.01	1.00
ig_5775619_5774806_at	3.68	3.61	3.56	3.70	0.01	1.00
ig_64729_65339_at	4.23	3.94	4.12	4.07	-0.01	1.00
ig_68616_69272_at	2.84	2.85	2.85	2.83	0.00	1.00
ig_773696_774416_at	3.38	2.97	2.96	3.40	-0.01	1.00
ig_774416_773696_at	3.20	2.82	3.02	3.01	-0.01	1.00
ig_788253_789144_at	5.22	5.02	5.12	5.12	0.00	1.00
ig_901934_901046_at	2.97	2.88	2.92	2.93	0.00	1.00
PA0005_at	2.64	2.98	2.64	2.97	0.01	1.00
PA0021_at	3.04	2.69	3.03	2.75	-0.02	1.00
PA0026_at	3.98	4.21	4.01	4.20	-0.01	1.00
PA0053_at	3.20	3.17	3.15	3.20	0.01	1.00
PA0054_at	3.10	3.05	3.09	3.08	-0.01	1.00
PA0061_at	3.10	3.13	3.13	3.13	-0.01	1.00
PA0072_at	3.93	3.78	3.94	3.75	0.01	1.00
PA0092_at	3.34	3.26	3.29	3.34	-0.01	1.00
PA0095_at	4.85	4.65	4.74	4.73	0.02	1.00
PA0110_at	2.99	3.03	3.13	2.92	-0.02	1.00
PA0115_at	3.35	3.25	3.33	3.23	0.02	1.00
PA0118_at	3.58	3.48	3.53	3.54	-0.01	1.00
PA0130_at	3.88	4.04	3.89	4.01	0.01	1.00
PA0131_at	2.47	2.74	2.66	2.54	0.01	1.00
PA0149_at	3.19	3.28	3.37	3.12	-0.01	1.00
PA0155_pcaR_at	4.37	4.40	4.39	4.38	0.00	1.00
PA0185_at	3.93	3.72	3.72	3.96	-0.02	1.00
PA0188_at	3.86	3.25	3.64	3.51	-0.02	1.00
PA0199_exbD1_at	3.25	2.93	2.99	3.24	-0.02	1.00
PA0203_at	4.30	3.84	3.97	4.15	0.01	1.00
PA0212_mdcE_at	3.35	3.18	3.24	3.27	0.01	1.00
PA0216_at	3.33	3.16	3.32	3.18	-0.01	1.00
PA0218_at	3.11	3.08	3.07	3.15	-0.01	1.00

PA0223_at	4.59	4.42	4.52	4.48	0.00	1.00
PA0226_at	4.75	4.38	4.64	4.48	0.00	1.00
PA0231_pcaD_at	3.29	3.31	3.48	3.15	-0.02	1.00
PA0234_at	2.59	2.32	2.46	2.45	0.00	1.00
PA0241_at	3.90	3.36	3.50	3.73	0.02	1.00
PA0274_at	4.10	3.64	3.89	3.88	-0.02	1.00
PA0287_at	2.32	2.16	2.33	2.17	-0.01	1.00
PA0306_at	3.45	3.26	3.42	3.31	-0.01	1.00
PA0317_at	3.82	3.92	3.78	3.99	-0.02	1.00
PA0337_ptsP_at	4.10	4.46	4.24	4.32	0.00	1.00
PA0341_lgt_at	2.77	2.61	2.57	2.81	-0.01	1.00
PA0352_at	3.39	3.83	3.65	3.54	0.01	1.00
PA0369_at	3.12	3.01	3.13	3.00	0.00	1.00
PA0374_ftsE_at	4.12	4.44	4.23	4.34	0.00	1.00
PA0391_at	6.17	6.16	6.18	6.18	-0.01	1.00
PA0397_at	2.68	2.60	2.70	2.61	-0.01	1.00
PA0402_pyrB_at	5.55	5.77	5.69	5.66	-0.01	1.00
PA0407_gshB_at	4.33	4.27	4.27	4.35	-0.01	1.00
PA0408_pilG_at	5.54	6.34	6.08	5.75	0.02	1.00
PA0419_at	3.75	3.60	3.76	3.58	0.01	1.00
PA0421_at	3.45	3.82	3.60	3.68	-0.01	1.00
PA0433_at	2.65	2.51	2.72	2.40	0.02	1.00
PA0444_at	2.82	2.59	2.59	2.80	0.01	1.00
PA0445_s_at	3.18	3.32	3.33	3.19	-0.01	1.00
PA0452_at	3.52	3.23	3.53	3.19	0.02	1.00
PA0455_dbpA_at	3.35	3.38	3.50	3.23	0.00	1.00
PA0457_at	3.74	3.49	3.58	3.67	-0.01	1.00
PA0478_at	3.06	2.76	2.95	2.87	0.00	1.00
PA0479_at	4.12	3.67	3.87	3.94	-0.01	1.00
PA0484_at	4.62	4.55	4.55	4.62	0.00	1.00
PA0497_at	3.53	3.21	3.29	3.42	0.02	1.00
PA0532_at	2.61	2.58	2.70	2.49	0.00	1.00
PA0535_at	3.64	3.61	3.62	3.65	-0.01	1.00
PA0544_at	3.79	3.69	3.77	3.71	0.00	1.00
PA0557_at	2.62	2.45	2.47	2.58	0.01	1.00
PA0559_at	4.03	3.98	3.99	4.02	0.00	1.00
PA0563_at	4.27	4.35	4.23	4.42	-0.01	1.00
PA0565_at	3.27	2.97	3.19	3.05	0.00	1.00
PA0568_at	3.97	3.57	3.72	3.79	0.02	1.00
PA0569_at	3.54	3.17	3.30	3.45	-0.02	1.00
PA0573_at	2.62	2.34	2.47	2.51	-0.01	1.00
PA0580_gcp_at	4.00	3.99	4.06	3.93	0.00	1.00
PA0587_at	5.22	5.58	5.39	5.42	0.00	1.00
PA0653_at	2.87	2.79	2.81	2.86	0.00	1.00

PA0672_at	2.81	2.87	2.85	2.83	0.00	1.00
PA0674_at	3.20	2.93	3.11	3.05	-0.01	1.00
PA0675_at	3.10	2.92	3.20	2.83	0.00	1.00
PA0677_at	3.14	2.76	3.14	2.79	-0.02	1.00
PA0697_at	2.66	2.37	2.47	2.54	0.01	1.00
PA0699_at	3.13	2.68	2.81	2.99	0.01	1.00
PA0701_at	3.14	2.84	3.00	3.00	-0.01	1.00
PA0703_at	3.13	3.08	3.16	3.07	-0.01	1.00
PA0704_at	3.55	3.48	3.45	3.58	0.00	1.00
PA0714_at	3.82	3.79	3.79	3.83	-0.01	1.00
PA0719_at	12.05	11.84	11.89	12.00	0.00	1.00
PA0740_at	3.02	2.64	2.80	2.83	0.01	1.00
PA0752_at	3.07	2.93	3.02	2.99	0.00	1.00
PA0754_at	3.12	2.99	3.16	2.95	0.00	1.00
PA0757_at	3.89	4.30	3.92	4.23	0.02	1.00
PA0780_at	3.30	3.33	3.19	3.44	0.00	1.00
PA0784_at	3.36	3.31	3.36	3.29	0.01	1.00
PA0800_at	3.69	3.70	3.79	3.61	-0.01	1.00
PA0810_at	3.95	4.01	4.08	3.85	0.01	1.00
PA0821_at	2.47	2.59	2.68	2.40	-0.01	1.00
PA0837_slyD_at	4.89	5.17	5.19	4.90	-0.02	1.00
PA0845_at	2.42	2.46	2.52	2.37	-0.01	1.00
PA0847_at	3.38	3.07	3.25	3.19	0.01	1.00
PA0848_at	2.56	2.61	2.61	2.53	0.02	1.00
PA0859_at	3.84	4.30	4.15	4.03	-0.02	1.00
PA0868_at	2.47	2.54	2.62	2.37	0.01	1.00
PA0874_at	2.48	2.59	2.53	2.53	0.00	1.00
PA0883_at	2.97	2.64	2.82	2.82	-0.01	1.00
PA0920_at	4.10	4.11	4.34	3.89	-0.01	1.00
PA0939_at	2.79	2.53	2.89	2.48	-0.02	1.00
PA0961_at	3.95	3.95	3.94	3.94	0.01	1.00
PA0989_at	2.69	2.47	2.60	2.55	0.00	1.00
PA0992_at	4.10	3.86	4.22	3.78	-0.02	1.00
PA1027_at	2.96	2.63	2.79	2.81	-0.01	1.00
PA1029_at	6.10	6.59	6.25	6.47	-0.02	1.00
PA1040_at	3.66	3.88	3.86	3.70	-0.01	1.00
PA1042_at	2.51	2.44	2.54	2.44	-0.01	1.00
PA1046_at	2.73	2.63	2.57	2.81	-0.01	1.00
PA1048_at	4.51	4.75	4.69	4.57	0.00	1.00
PA1056_at	2.57	2.68	2.57	2.68	0.00	1.00
PA1066_at	2.91	2.70	2.65	2.98	-0.01	1.00
PA1069_at	3.44	3.44	3.26	3.61	0.00	1.00
PA1072_braE_at	2.93	2.83	2.86	2.88	0.01	1.00
PA1074_braC_at	5.73	6.19	5.94	6.01	-0.02	1.00

PA1086_flgK_at	4.63	4.58	4.48	4.75	-0.02	1.00
PA1107_at	3.41	2.96	3.11	3.31	-0.02	1.00
PA1111_at	3.47	3.61	3.47	3.59	0.01	1.00
PA1115_at	3.71	3.64	3.58	3.77	0.00	1.00
PA1117_at	2.67	3.37	3.07	2.99	-0.01	1.00
PA1125_at	4.93	4.95	5.39	4.52	-0.01	1.00
PA1135_at	4.44	4.22	4.43	4.23	0.00	1.00
PA1143_at	3.48	2.87	3.25	3.08	0.01	1.00
PA1147_at	4.12	4.06	3.97	4.18	0.01	1.00
PA1154_at	2.57	2.50	2.53	2.52	0.01	1.00
PA1165_at	5.48	4.87	5.22	5.12	0.00	1.00
PA1183_dctA_at	3.69	3.96	3.99	3.62	0.02	1.00
PA1196_at	3.81	4.10	3.92	3.99	0.00	1.00
PA1201_at	3.38	2.98	3.10	3.25	0.00	1.00
PA1203_at	4.21	3.94	4.06	4.10	-0.01	1.00
PA1204_at	2.54	2.54	2.50	2.61	-0.02	1.00
PA1206_at	4.58	4.91	4.84	4.69	-0.02	1.00
PA1209_at	5.31	5.02	5.09	5.22	0.01	1.00
PA1225_at	3.69	3.42	3.48	3.65	-0.01	1.00
PA1227_at	2.93	2.96	2.90	2.97	0.00	1.00
PA1233_at	2.79	2.72	2.77	2.73	0.01	1.00
PA1239_at	2.36	2.36	2.25	2.48	0.00	1.00
PA1253_at	3.08	3.09	3.08	3.07	0.01	1.00
PA1277_cobQ_at	3.54	3.49	3.66	3.37	0.00	1.00
PA1280_at	3.34	2.89	3.04	3.19	0.00	1.00
PA1292_at	5.06	5.32	5.27	5.08	0.01	1.00
PA1297_at	3.14	3.11	3.10	3.14	0.00	1.00
PA1304_at	2.94	3.16	2.91	3.23	-0.02	1.00
PA1307_at	6.12	5.99	6.03	6.07	0.01	1.00
PA1314_at	4.06	3.94	3.95	4.05	0.00	1.00
PA1315_at	4.82	4.56	4.73	4.65	0.00	1.00
PA1329_at	3.28	3.07	3.34	3.00	0.00	1.00
PA1335_at	4.32	3.92	4.24	3.98	0.01	1.00
PA1346_at	4.11	4.06	4.12	4.06	0.00	1.00
PA1348_at	5.52	5.33	5.39	5.46	0.00	1.00
PA1351_at	3.16	2.98	2.97	3.19	-0.01	1.00
PA1355_at	2.91	2.98	3.35	2.58	-0.02	1.00
PA1363_at	2.33	2.31	2.32	2.29	0.02	1.00
PA1369_at	2.24	2.39	2.30	2.31	0.01	1.00
PA1378_at	2.94	2.95	2.79	3.07	0.02	1.00
PA1383_at	2.98	2.74	2.73	3.00	0.00	1.00
PA1392_at	3.23	3.02	3.28	2.99	-0.01	1.00
PA1393_cysC_at	2.50	2.31	2.45	2.38	-0.01	1.00
PA1403_at	2.99	2.79	2.92	2.89	-0.01	1.00

PA1418_at	3.31	3.23	3.35	3.22	-0.02	1.00
PA1426_at	2.87	2.54	2.70	2.71	0.00	1.00
PA1433_at	3.47	2.95	3.27	3.15	-0.01	1.00
PA1444_fliN_at	2.55	2.49	2.48	2.57	-0.01	1.00
PA1447_fliQ_at	3.97	3.83	4.11	3.67	0.01	1.00
PA1452_flhA_at	3.22	3.68	3.54	3.39	-0.01	1.00
PA1458_at	5.63	6.45	6.08	6.04	-0.02	1.00
PA1463_at	5.00	5.65	5.13	5.55	-0.02	1.00
PA1492_at	4.03	3.44	3.68	3.73	0.03	1.00
PA1501_at	2.61	2.37	2.53	2.48	-0.02	1.00
PA1514_at	3.32	3.15	3.25	3.21	0.01	1.00
PA1515_alc_at	4.27	4.25	4.20	4.32	0.00	1.00
PA1538_at	2.98	2.86	2.82	3.05	-0.01	1.00
PA1551_at	4.64	5.29	4.96	4.99	0.00	1.00
PA1564_at	3.31	3.95	3.42	3.79	0.03	1.00
PA1592_i_at	9.37	10.23	9.57	9.99	0.02	1.00
PA1601_at	2.75	2.68	2.69	2.73	0.01	1.00
PA1611_at	3.62	3.58	3.69	3.53	-0.01	1.00
PA1613_at	2.94	2.93	2.88	2.98	0.00	1.00
PA1616_at	2.96	4.05	3.43	3.57	0.00	1.00
PA1624_at	3.76	3.58	3.73	3.61	0.00	1.00
PA1646_at	4.26	4.04	4.33	3.94	0.02	1.00
PA1647_at	3.38	3.27	3.33	3.34	-0.01	1.00
PA1671_stk1_at	3.51	3.44	3.45	3.51	0.00	1.00
PA1672_at	3.57	3.79	3.62	3.73	0.00	1.00
PA1683_at	3.41	3.06	3.25	3.19	0.01	1.00
PA1688_at	2.83	2.82	2.94	2.71	0.00	1.00
PA1692_at	5.07	5.51	5.30	5.30	-0.01	1.00
PA1717_pscD_at	5.38	6.58	6.11	5.92	-0.03	1.00
PA1724_pscK_at	3.54	4.62	4.09	4.04	0.02	1.00
PA1737_at	3.33	3.43	3.34	3.42	0.00	1.00
PA1738_at	2.73	2.48	2.53	2.65	0.02	1.00
PA1743_at	2.30	2.12	2.30	2.13	-0.01	1.00
PA1764_at	2.61	2.45	2.58	2.50	-0.01	1.00
PA1794_glnS_at	4.45	4.60	4.52	4.55	0.00	1.00
PA1798_at	4.31	4.16	4.27	4.18	0.01	1.00
PA1818_at	2.73	2.96	2.80	2.88	0.01	1.00
PA1822_at	3.51	3.71	3.45	3.75	0.01	1.00
PA1829_at	2.66	2.61	2.62	2.66	-0.01	1.00
PA1830_at	2.98	2.87	2.89	2.95	0.01	1.00
PA1832_at	3.00	2.90	2.98	2.93	0.00	1.00
PA1843_metH_at	4.10	4.23	4.02	4.31	0.00	1.00
PA1849_at	2.94	2.88	2.86	2.97	0.00	1.00
PA1853_at	3.62	3.43	3.55	3.50	0.01	1.00

PA1854_at	2.48	2.24	2.19	2.52	0.00	1.00
PA1855_r_at	4.09	4.20	4.03	4.22	0.02	1.00
PA1867_at	3.86	3.95	3.89	3.91	0.00	1.00
PA1872_at	3.33	3.53	3.60	3.27	0.00	1.00
PA1876_at	3.63	3.55	3.57	3.61	0.00	1.00
PA1883_at	4.04	3.71	3.92	3.80	0.01	1.00
PA1887_at	4.49	4.22	4.47	4.27	-0.01	1.00
PA1890_at	3.29	3.10	3.34	3.04	0.01	1.00
PA1891_at	3.70	3.36	3.51	3.57	-0.01	1.00
PA1893_at	3.45	3.35	3.35	3.48	-0.01	1.00
PA1908_at	3.24	2.65	2.89	2.99	0.01	1.00
PA1933_at	3.31	3.40	3.40	3.32	0.00	1.00
PA1958_at	3.46	3.23	3.40	3.29	0.00	1.00
PA1964_at	3.28	3.44	3.39	3.29	0.01	1.00
PA1976_at	3.81	3.30	3.60	3.47	0.02	1.00
PA1977_at	3.84	3.42	3.72	3.51	0.02	1.00
PA1991_at	3.45	3.24	3.36	3.33	0.00	1.00
PA2010_at	2.90	2.84	2.77	2.96	0.01	1.00
PA2032_at	4.02	3.44	3.66	3.76	0.02	1.00
PA2045_at	4.44	4.49	4.48	4.42	0.01	1.00
PA2061_at	2.45	2.45	2.45	2.45	0.00	1.00
PA2076_at	3.00	2.87	2.92	2.95	0.00	1.00
PA2082_at	3.59	3.72	3.72	3.62	-0.02	1.00
PA2085_at	4.19	3.58	3.92	3.81	0.02	1.00
PA2091_at	4.63	4.04	4.36	4.34	-0.01	1.00
PA2094_at	3.08	3.02	3.02	3.09	0.00	1.00
PA2095_at	2.44	2.33	2.40	2.37	0.00	1.00
PA2096_at	3.22	3.16	3.23	3.16	-0.01	1.00
PA2107_at	4.76	4.36	4.49	4.64	0.00	1.00
PA2116_at	3.70	3.38	3.49	3.61	-0.01	1.00
PA2155_at	2.61	2.53	2.54	2.59	0.01	1.00
PA2156_at	2.13	2.00	2.11	2.05	-0.01	1.00
PA2161_at	2.18	2.08	2.02	2.25	0.00	1.00
PA2166_at	3.11	2.84	2.90	3.04	0.00	1.00
PA2171_at	3.53	3.12	3.18	3.43	0.02	1.00
PA2175_at	3.04	3.26	3.14	3.13	0.02	1.00
PA2179_at	2.94	2.68	2.65	2.98	0.00	1.00
PA2205_at	4.00	3.36	3.86	3.55	-0.03	1.00
PA2209_at	2.53	2.28	2.52	2.32	-0.02	1.00
PA2216_at	4.33	3.55	4.02	3.84	0.01	1.00
PA2226_at	2.47	2.30	2.42	2.34	0.01	1.00
PA2241_at	4.65	4.35	4.59	4.38	0.02	1.00
PA2242_at	5.02	4.98	5.06	4.93	0.00	1.00
PA2252_at	2.90	2.88	2.74	3.03	0.00	1.00

PA2262_at	3.03	2.63	2.78	2.85	0.01	1.00
PA2268_at	2.46	2.26	2.40	2.35	-0.02	1.00
PA2279_arsC_at	2.47	2.62	2.58	2.54	-0.02	1.00
PA2287_at	2.79	2.54	2.77	2.54	0.01	1.00
PA2295_at	2.97	2.85	2.93	2.87	0.01	1.00
PA2314_at	3.49	3.46	3.43	3.54	-0.01	1.00
PA2317_at	2.78	2.73	2.84	2.65	0.01	1.00
PA2337_mtlR_at	3.08	2.71	2.84	2.95	0.00	1.00
PA2353_at	2.71	2.44	2.65	2.50	0.00	1.00
PA2363_at	3.65	3.84	3.65	3.86	-0.01	1.00
PA2364_at	4.56	4.64	4.80	4.43	-0.01	1.00
PA2383_at	2.71	2.82	2.81	2.72	0.00	1.00
PA2395_at	2.63	2.38	2.63	2.38	0.00	1.00
PA2398_fpvA_at	2.26	1.99	2.15	2.08	0.01	1.00
PA2409_at	3.06	2.81	2.98	2.88	0.01	1.00
PA2417_at	3.20	2.91	3.02	3.07	0.01	1.00
PA2418_at	3.28	3.15	3.26	3.20	-0.02	1.00
PA2420_at	2.51	2.43	2.48	2.43	0.02	1.00
PA2425_at	3.53	3.51	3.48	3.53	0.01	1.00
PA2428_at	3.01	2.73	2.80	2.95	-0.01	1.00
PA2435_at	2.91	2.78	2.68	2.98	0.01	1.00
PA2441_at	3.11	2.72	2.88	2.97	-0.01	1.00
PA2461_at	2.49	2.46	2.52	2.45	-0.01	1.00
PA2467_at	3.25	2.71	3.06	2.87	0.01	1.00
PA2472_at	2.83	2.61	2.67	2.73	0.02	1.00
PA2477_at	3.66	3.36	3.53	3.50	0.00	1.00
PA2509_catB_at	3.28	3.16	3.41	3.04	-0.01	1.00
PA2520_czcA_at	3.11	3.05	3.00	3.19	-0.01	1.00
PA2542_at	3.32	3.13	3.30	3.17	0.00	1.00
PA2563_at	3.37	3.18	3.27	3.32	-0.02	1.00
PA2564_at	3.79	3.46	3.57	3.65	0.02	1.00
PA2569_at	4.05	4.06	3.97	4.11	0.01	1.00
PA2580_at	3.60	3.62	3.51	3.70	0.01	1.00
PA2590_at	2.40	2.46	2.36	2.47	0.02	1.00
PA2597_at	3.80	3.20	3.52	3.51	-0.02	1.00
PA2603_at	3.14	2.94	3.11	2.95	0.01	1.00
PA2625_at	2.41	2.48	2.47	2.44	-0.01	1.00
PA2650_at	4.02	3.38	3.93	3.49	-0.01	1.00
PA2654_at	4.56	4.91	4.76	4.73	-0.01	1.00
PA2671_at	3.89	3.78	3.70	3.94	0.01	1.00
PA2678_at	2.90	2.76	2.83	2.80	0.01	1.00
PA2684_at	3.31	3.56	3.34	3.49	0.01	1.00
PA2687_pfeS_at	2.85	2.54	2.76	2.66	-0.02	1.00
PA2695_at	3.67	3.45	3.68	3.41	0.01	1.00

PA2698_at	3.54	3.25	3.42	3.32	0.02	1.00
PA2702_at	2.72	2.46	2.55	2.62	0.01	1.00
PA2714_at	2.72	2.48	2.57	2.66	-0.02	1.00
PA2729_at	3.61	3.42	3.35	3.71	-0.02	1.00
PA2732_at	4.58	4.08	4.36	4.26	0.02	1.00
PA2740_pheS_at	4.39	4.84	4.63	4.56	0.02	1.00
PA2742_rpmI_at	7.35	8.69	7.93	8.14	-0.02	1.00
PA2744_thrS_at	5.10	5.97	5.51	5.57	-0.01	1.00
PA2756_at	3.62	3.18	3.53	3.30	-0.01	1.00
PA2774_at	3.35	3.30	3.29	3.36	0.00	1.00
PA2775_at	4.35	3.95	4.17	4.16	-0.01	1.00
PA2789_at	2.44	2.43	2.47	2.42	-0.02	1.00
PA2822_at	3.50	3.23	3.50	3.27	-0.02	1.00
PA2837_at	4.55	3.75	4.21	4.08	0.01	1.00
PA2844_at	3.32	3.24	3.43	3.15	-0.01	1.00
PA2851_efp_at	5.31	5.87	5.57	5.66	-0.02	1.00
PA2857_at	3.12	3.19	3.16	3.14	0.00	1.00
PA2858_at	3.34	3.50	3.33	3.49	0.01	1.00
PA2866_mttC_at	3.42	3.38	3.10	3.69	0.01	1.00
PA2867_at	4.69	5.30	4.83	5.11	0.02	1.00
PA2878_at	2.97	2.85	2.96	2.84	0.01	1.00
PA2886_at	3.74	3.46	3.53	3.63	0.02	1.00
PA2887_at	2.83	2.83	2.81	2.84	0.00	1.00
PA2895_at	3.91	4.61	4.05	4.43	0.02	1.00
PA2928_at	4.35	4.00	4.38	3.99	-0.01	1.00
PA2935_at	2.47	2.31	2.41	2.35	0.01	1.00
PA2957_at	4.48	5.08	4.67	4.88	0.01	1.00
PA2959_at	4.05	4.86	4.35	4.51	0.02	1.00
PA2974_at	3.63	3.95	3.85	3.75	-0.01	1.00
PA2979_kdsB_at	3.59	3.57	3.50	3.62	0.01	1.00
PA2981_lpxK_at	3.71	3.43	3.79	3.33	0.01	1.00
PA2990_at	5.97	6.25	6.04	6.15	0.02	1.00
PA3025_at	3.92	3.58	3.96	3.55	0.00	1.00
PA3028_moeA2_at	3.51	3.46	3.45	3.49	0.01	1.00
PA3035_at	3.53	3.27	3.52	3.28	0.00	1.00
PA3036_at	3.22	2.93	3.13	2.98	0.02	1.00
PA3043_at	2.93	3.00	2.80	3.17	-0.02	1.00
PA3050_pyrD_at	5.25	5.29	5.30	5.21	0.02	1.00
PA3053_at	3.30	3.02	3.30	2.99	0.02	1.00
PA3072_at	4.51	4.31	4.56	4.30	-0.02	1.00
PA3073_at	4.51	3.89	4.14	4.22	0.02	1.00
PA3105_xcpQ_at	3.69	3.66	3.57	3.80	-0.01	1.00
PA3117_asd_at	3.79	3.80	3.82	3.77	0.00	1.00
PA3144_f_at	2.84	3.15	3.18	2.76	0.03	1.00

PA3154_wzy_at	2.80	2.88	2.95	2.73	0.00	1.00
PA3188_at	3.06	2.59	2.72	2.92	0.00	1.00
PA3191_at	3.09	2.93	2.92	3.09	0.00	1.00
PA3193_glk_at	3.48	3.65	3.62	3.54	-0.02	1.00
PA3222_at	3.71	3.39	3.42	3.64	0.02	1.00
PA3237_at	2.76	2.50	2.62	2.64	0.00	1.00
PA3241_at	3.52	3.02	3.01	3.56	-0.01	1.00
PA3248_at	3.84	3.59	3.70	3.74	0.00	1.00
PA3255_at	3.29	3.07	3.25	3.10	0.01	1.00
PA3274_at	2.66	2.73	2.72	2.67	0.00	1.00
PA3292_at	3.22	3.09	3.20	3.08	0.01	1.00
PA3303_at	3.20	2.86	3.20	2.88	-0.01	1.00
PA3316_at	4.32	3.97	4.31	3.99	0.00	1.00
PA3325_at	2.90	2.41	2.56	2.76	0.00	1.00
PA3336_at	3.40	3.22	3.35	3.26	0.01	1.00
PA3347_at	3.51	3.66	3.62	3.59	-0.01	1.00
PA3358_at	3.80	3.82	3.79	3.82	0.01	1.00
PA3359_at	2.81	2.62	2.64	2.77	0.01	1.00
PA3366_amiE_at	4.02	4.05	4.24	3.87	-0.01	1.00
PA3368_at	3.37	2.83	3.27	2.91	0.01	1.00
PA3399_at	3.58	3.23	3.39	3.38	0.02	1.00
PA3409_at	2.96	2.62	2.93	2.64	0.01	1.00
PA3410_at	2.98	2.76	2.90	2.85	0.00	1.00
PA3426_at	2.67	2.71	2.89	2.53	-0.02	1.00
PA3442_at	3.29	3.25	3.28	3.25	0.01	1.00
PA3453_at	3.67	3.67	3.71	3.61	0.01	1.00
PA3522_at	2.90	2.60	2.72	2.75	0.01	1.00
PA3526_at	4.88	5.69	5.27	5.25	0.03	1.00
PA3539_at	3.42	3.16	3.35	3.23	0.00	1.00
PA3543_algK_at	3.23	2.92	3.25	2.89	0.01	1.00
PA3546_algX_at	3.59	3.02	3.18	3.41	0.01	1.00
PA3557_at	3.62	3.23	3.39	3.45	0.00	1.00
PA3561_fruK_at	5.56	5.58	5.59	5.54	0.00	1.00
PA3562_at	3.69	3.99	3.82	3.88	0.00	1.00
PA3573_at	3.17	3.08	3.08	3.16	0.00	1.00
PA3579_at	4.05	4.40	4.34	4.14	-0.01	1.00
PA3584_glpD_at	8.89	8.87	8.78	9.00	-0.01	1.00
PA3601_at	2.99	3.39	3.21	3.16	0.00	1.00
PA3602_at	4.50	4.59	4.72	4.34	0.01	1.00
PA3612_at	3.79	3.81	3.77	3.81	0.01	1.00
PA3637_pyrG_at	3.66	4.29	3.80	4.15	0.00	1.00
PA3644_lpxA_at	3.75	4.38	4.01	4.12	0.00	1.00
PA3660_at	3.37	3.40	3.27	3.48	0.01	1.00
PA3670_at	2.75	2.75	2.86	2.66	-0.01	1.00

PA3681_at	3.54	3.01	3.31	3.27	-0.02	1.00
PA3705_at	3.66	3.42	3.53	3.55	0.00	1.00
PA3711_at	3.25	3.01	3.21	3.02	0.01	1.00
PA3730_at	3.36	3.14	3.24	3.23	0.02	1.00
PA3733_at	3.24	3.15	3.23	3.15	0.00	1.00
PA3741_at	2.87	2.88	2.97	2.81	-0.02	1.00
PA3753_at	3.72	4.08	4.02	3.78	0.00	1.00
PA3766_at	4.02	3.72	3.85	3.92	-0.01	1.00
PA3771_at	2.35	2.26	2.30	2.32	-0.01	1.00
PA3777_xseA_at	3.06	2.93	3.11	2.90	-0.01	1.00
PA3782_at	3.87	3.83	3.86	3.86	-0.01	1.00
PA3798_at	3.98	3.82	3.98	3.85	-0.01	1.00
PA3799_at	5.22	5.20	5.13	5.28	0.00	1.00
PA3809_fdx2_at	4.31	4.74	4.42	4.59	0.02	1.00
PA3830_at	3.70	3.50	3.61	3.56	0.02	1.00
PA3831_pepA_at	4.75	4.92	4.83	4.83	0.01	1.00
PA3836_at	4.22	3.95	4.12	4.03	0.01	1.00
PA3844_at	4.17	4.21	4.11	4.27	0.00	1.00
PA3853_at	4.28	4.57	4.50	4.36	0.00	1.00
PA3864_at	3.17	3.21	3.14	3.21	0.01	1.00
PA3865_at	4.32	4.39	4.33	4.37	0.00	1.00
PA3878_narX_at	3.51	3.70	3.50	3.68	0.01	1.00
PA3897_at	2.92	2.74	2.81	2.86	0.00	1.00
PA3906_at	4.94	4.66	4.55	5.03	0.01	1.00
PA3909_at	3.45	3.20	3.41	3.24	0.00	1.00
PA3921_at	2.85	2.69	2.92	2.60	0.01	1.00
PA3926_at	4.87	4.33	4.74	4.51	-0.02	1.00
PA3928_r_at	4.34	4.42	4.34	4.42	0.00	1.00
PA3949_at	4.41	3.91	4.21	4.14	-0.01	1.00
PA3954_at	2.71	2.46	2.61	2.55	0.01	1.00
PA3957_at	2.96	2.58	2.79	2.80	-0.02	1.00
PA3964_at	2.72	2.74	2.61	2.88	-0.01	1.00
PA3974_at	3.15	2.91	3.02	3.07	-0.01	1.00
PA3979_at	4.20	4.26	4.24	4.23	0.00	1.00
PA3985_at	3.16	3.01	3.19	3.00	-0.01	1.00
PA4004_at	5.37	5.72	5.44	5.63	0.01	1.00
PA4008_at	2.77	2.35	2.71	2.40	0.00	1.00
PA4017_at	5.07	5.66	5.22	5.51	0.00	1.00
PA4019_at	3.34	3.75	3.47	3.66	-0.02	1.00
PA4043_ispA_at	4.36	4.39	4.44	4.29	0.01	1.00
PA4046_at	3.83	3.45	3.59	3.68	0.01	1.00
PA4052_nusB_at	3.81	3.79	3.75	3.84	0.01	1.00
PA4066_at	3.83	3.72	3.93	3.64	0.00	1.00
PA4073_at	3.59	3.37	3.47	3.49	0.00	1.00

PA4075_at	3.64	3.22	3.36	3.54	-0.02	1.00
PA4077_at	3.28	2.87	3.04	3.09	0.01	1.00
PA4083_at	3.46	2.95	2.88	3.55	-0.01	1.00
PA4092_hpaC_at	2.52	2.37	2.48	2.42	-0.01	1.00
PA4094_at	3.37	3.10	3.30	3.16	0.01	1.00
PA4103_at	2.92	2.24	2.64	2.49	0.01	1.00
PA4119_aph_at	3.56	3.16	3.62	3.09	0.00	1.00
PA4144_at	3.23	2.87	3.03	3.05	0.01	1.00
PA4150_at	4.85	4.49	4.67	4.67	0.00	1.00
PA4160_fepD_at	3.29	3.10	3.14	3.25	0.00	1.00
PA4164_at	3.54	3.42	3.53	3.44	-0.01	1.00
PA4165_at	3.89	3.35	3.71	3.55	-0.01	1.00
PA4171_at	2.87	2.75	2.82	2.81	-0.01	1.00
PA4173_at	2.71	2.61	2.64	2.67	0.01	1.00
PA4202_at	4.09	4.43	4.48	4.06	-0.01	1.00
PA4204_at	3.57	3.95	3.75	3.74	0.01	1.00
PA4205_at	3.61	4.01	3.75	3.85	0.01	1.00
PA4218_at	3.81	3.18	3.36	3.57	0.02	1.00
PA4227_pchR_at	2.43	2.47	2.41	2.49	0.00	1.00
PA4245_rpmD_at	5.19	6.10	5.63	5.62	0.02	1.00
PA4267_rpsG_at	10.08	10.56	10.31	10.35	-0.01	1.00
PA4276_secE_at	6.11	7.45	6.82	6.81	-0.03	1.00
PA4282_at	2.34	2.69	2.45	2.58	0.00	1.00
PA4288_at	3.83	3.94	3.77	4.03	-0.02	1.00
PA4299_at	2.64	2.67	2.64	2.67	0.00	1.00
PA4305_at	3.56	3.65	3.49	3.72	0.00	1.00
PA4338_at	3.58	3.27	3.47	3.39	-0.01	1.00
PA4341_at	3.05	2.84	2.74	3.19	-0.02	1.00
PA4342_at	3.45	2.99	3.33	3.06	0.02	1.00
PA4353_at	5.48	5.61	5.49	5.56	0.01	1.00
PA4362_at	3.57	3.56	3.57	3.56	0.00	1.00
PA4371_at	3.51	3.26	3.43	3.32	0.01	1.00
PA4375_at	2.52	2.36	2.54	2.32	0.01	1.00
PA4395_at	3.90	4.09	4.08	3.89	0.01	1.00
PA4398_at	3.28	2.77	3.04	3.03	-0.01	1.00
PA4400_at	3.06	2.98	2.88	3.13	0.02	1.00
PA4411_murC_at	4.06	4.28	4.22	4.14	-0.01	1.00
PA4416_murF_at	4.40	4.88	4.60	4.72	-0.02	1.00
PA4436_at	3.24	3.03	3.04	3.24	-0.01	1.00
PA4437_at	3.80	3.56	3.79	3.59	-0.02	1.00
PA4440_at	2.97	3.31	3.11	3.20	-0.01	1.00
PA4450_murA_at	4.01	4.76	4.24	4.55	-0.01	1.00
PA4461_at	4.09	4.28	4.23	4.13	0.00	1.00
PA4467_at	3.69	3.69	3.74	3.67	-0.02	1.00

PA4468_sodM_at	3.97	3.36	3.74	3.64	-0.03	1.00
PA4470_fumC1_at	2.87	2.78	2.89	2.78	-0.01	1.00
PA4493_at	5.35	5.31	5.44	5.20	0.01	1.00
PA4494_at	3.39	3.72	3.36	3.75	0.00	1.00
PA4504_at	3.31	3.25	3.14	3.39	0.01	1.00
PA4506_at	3.90	4.33	4.12	4.10	0.01	1.00
PA4509_at	3.81	3.39	3.69	3.55	-0.02	1.00
PA4513_at	2.51	2.37	2.50	2.36	0.02	1.00
PA4517_at	3.06	2.82	3.04	2.84	0.00	1.00
PA4521_at	3.58	3.71	3.66	3.64	0.00	1.00
PA4531_at	2.48	2.52	2.48	2.52	0.00	1.00
PA4564_at	3.73	4.02	3.93	3.80	0.01	1.00
PA4583_at	3.28	3.31	3.37	3.24	-0.01	1.00
PA4594_at	3.66	3.27	3.46	3.50	-0.02	1.00
PA4598_mexD_at	3.82	3.35	3.65	3.52	0.00	1.00
PA4602_glyA3_at	4.20	4.33	4.21	4.29	0.01	1.00
PA4605_at	3.16	3.33	3.28	3.24	-0.02	1.00
PA4630_at	2.93	3.00	3.03	2.92	-0.01	1.00
PA4631_at	3.69	3.73	3.69	3.71	0.01	1.00
PA4634_at	3.27	2.97	2.97	3.24	0.02	1.00
PA4651_at	3.00	2.57	2.82	2.77	0.00	1.00
PA4667_at	4.70	5.22	4.86	5.06	0.00	1.00
PA4672_at	4.11	4.63	4.34	4.41	0.00	1.00
PA4675_at	3.23	2.97	3.07	3.15	-0.01	1.00
PA4692_at	4.17	4.25	4.02	4.36	0.02	1.00
PA4695_ilvH_at	4.89	4.82	4.85	4.85	0.01	1.00
PA4712_at	3.38	3.16	3.27	3.31	-0.02	1.00
PA4747_secG_at	2.70	3.14	2.89	2.96	-0.01	1.00
PA4750_folP_at	4.25	4.19	4.30	4.14	0.00	1.00
PA4754_at	5.37	4.98	5.25	5.14	-0.02	1.00
PA4756_carB_at	5.03	5.21	5.08	5.13	0.01	1.00
PA4758_carA_at	4.27	4.50	4.47	4.30	0.00	1.00
PA4769_at	3.65	3.67	3.63	3.68	0.01	1.00
PA4772_at	2.93	3.04	2.97	3.01	0.00	1.00
PA4783_at	2.98	2.63	2.91	2.73	-0.01	1.00
PA4806_at	2.72	2.64	2.70	2.65	0.00	1.00
PA4807_selB_at	3.22	3.35	3.35	3.21	0.00	1.00
PA4811_fdnH_at	4.85	4.60	4.64	4.81	0.01	1.00
PA4835_at	3.68	3.22	3.40	3.48	0.01	1.00
PA4842_at	6.06	6.68	6.29	6.49	-0.02	1.00
PA4860_at	3.45	3.11	3.37	3.16	0.02	1.00
PA4871_at	2.57	2.41	2.44	2.51	0.01	1.00
PA4893_ureG_at	3.20	3.08	3.28	2.98	0.00	1.00
PA4903_at	4.16	3.93	4.25	3.85	-0.01	1.00

PA4906_at	2.72	2.97	2.78	2.89	0.01	1.00
PA4912_at	2.48	2.59	2.52	2.53	0.01	1.00
PA4913_at	4.00	3.73	3.89	3.81	0.02	1.00
PA4924_at	3.10	3.10	3.20	2.97	0.02	1.00
PA4926_at	3.42	3.13	3.30	3.23	0.01	1.00
PA4930_alr_at	2.92	3.02	2.91	3.01	0.01	1.00
PA4936_at	5.23	5.46	5.40	5.30	-0.01	1.00
PA4950_at	3.09	2.88	3.03	2.90	0.02	1.00
PA4958_at	6.05	6.06	5.90	6.21	0.00	1.00
PA4965_at	3.75	3.82	3.80	3.78	0.00	1.00
PA4972_at	2.80	3.67	3.19	3.23	0.03	1.00
PA4975_at	2.85	2.50	2.72	2.62	0.01	1.00
PA4976_aspC_at	3.35	3.42	3.70	3.02	0.02	1.00
PA4979_at	3.03	2.76	2.83	2.99	-0.02	1.00
PA4984_at	3.60	3.30	3.43	3.48	-0.01	1.00
PA4991_at	4.05	3.85	3.94	3.96	0.00	1.00
PA4992_at	2.76	2.77	2.74	2.77	0.01	1.00
PA5005_at	3.33	3.27	3.25	3.34	0.01	1.00
PA5012_waaF_at	3.88	4.05	3.93	3.97	0.02	1.00
PA5017_at	2.66	2.71	2.86	2.55	-0.02	1.00
PA5059_at	2.83	2.85	2.83	2.87	-0.01	1.00
PA5075_at	4.93	4.63	4.69	4.85	0.00	1.00
PA5093_at	3.91	3.84	3.97	3.78	0.00	1.00
PA5097_at	2.55	2.60	2.57	2.56	0.01	1.00
PA5105_hutC_at	3.19	3.40	3.28	3.30	0.00	1.00
PA5112_estA_at	5.32	5.20	5.35	5.21	-0.02	1.00
PA5116_at	4.23	3.98	4.30	3.95	-0.02	1.00
PA5120_at	3.39	3.69	3.62	3.48	-0.02	1.00
PA5122_at	3.84	3.98	3.85	3.95	0.01	1.00
PA5142_hisH1_at	3.97	4.21	3.95	4.21	0.01	1.00
PA5162_rmlD_at	4.20	4.16	4.22	4.13	0.00	1.00
PA5169_at	2.77	2.71	2.72	2.79	-0.01	1.00
PA5175_cysQ_at	2.86	3.30	3.01	3.17	-0.01	1.00
PA5178_at	8.13	8.40	8.22	8.34	-0.01	1.00
PA5179_at	3.56	3.31	3.47	3.40	0.00	1.00
PA5187_at	3.35	3.32	3.34	3.32	0.01	1.00
PA5208_at	4.03	3.76	3.77	4.03	-0.01	1.00
PA5209_at	3.84	4.10	4.11	3.81	0.01	1.00
PA5212_i_at	7.69	8.74	8.03	8.45	-0.03	1.00
PA5225_at	4.49	4.89	4.64	4.70	0.02	1.00
PA5231_at	3.65	4.06	3.90	3.81	0.00	1.00
PA5240_trxA_at	5.70	5.90	5.71	5.87	0.01	1.00
PA5241_ppx_at	2.72	2.97	2.78	2.95	-0.02	1.00
PA5251_at	3.63	3.79	3.72	3.66	0.02	1.00

PA5264_at	4.35	4.39	4.37	4.36	0.01	1.00
PA5277_lysA_at	3.74	3.80	3.81	3.75	0.00	1.00
PA5284_at	2.73	2.63	2.58	2.75	0.01	1.00
PA5291_at	2.97	2.70	2.82	2.85	0.00	1.00
PA5292_at	2.55	2.70	2.66	2.58	0.00	1.00
PA5294_at	4.65	4.12	4.52	4.26	0.00	1.00
PA5304_dadA_at	6.95	7.61	7.23	7.29	0.02	1.00
PA5316_rpmB_at	6.01	7.70	6.80	6.90	0.00	1.00
PA5325_at	2.77	2.68	2.65	2.80	0.01	1.00
PA5328_at	2.47	2.38	2.44	2.39	0.01	1.00
PA5330_at	4.70	4.84	4.74	4.79	0.00	1.00
PA5333_at	3.60	3.63	3.70	3.52	0.01	1.00
PA5353_glcF_at	3.33	3.24	3.09	3.46	0.01	1.00
PA5360_phoB_at	3.15	2.93	2.89	3.16	0.02	1.00
PA5364_at	5.40	5.26	5.39	5.27	0.00	1.00
PA5369_at	4.54	4.07	4.24	4.31	0.03	1.00
PA5370_at	2.91	2.65	2.77	2.82	-0.01	1.00
PA5384_at	3.21	3.15	3.25	3.13	-0.01	1.00
PA5397_at	3.14	2.67	2.93	2.87	0.00	1.00
PA5400_at	2.70	2.40	2.53	2.53	0.02	1.00
PA5410_at	2.88	2.81	2.92	2.74	0.02	1.00
PA5416_soxB_at	2.73	2.44	2.61	2.59	-0.02	1.00
PA5420_purU2_at	3.89	3.56	3.58	3.88	0.00	1.00
PA5421_fdhA_at	4.07	3.27	3.85	3.43	0.03	1.00
PA5431_at	2.64	2.46	2.67	2.42	0.00	1.00
PA5433_at	2.97	2.84	2.84	2.96	0.01	1.00
PA5438_at	5.34	5.61	5.43	5.52	0.00	1.00
PA5449_wbpX_at	3.72	3.38	3.55	3.57	-0.01	1.00
PA5454_rmd_at	2.64	2.90	2.76	2.77	0.01	1.00
PA5478_at	3.21	3.45	3.30	3.36	0.00	1.00
PA5489_dsbA_at	5.52	6.58	5.99	6.05	0.03	1.00
PA5515_at	2.89	2.75	2.80	2.84	0.01	1.00
PA5533_at	2.15	2.38	2.13	2.40	0.00	1.00
PA5543_at	3.97	3.66	3.91	3.73	0.00	1.00
PA5557_atpH_at	7.94	8.33	8.11	8.16	0.00	1.00
Pae_AF035937cds3_at	3.40	2.98	3.14	3.26	-0.01	1.00
Pae_AF043558cds_at	2.38	2.17	2.51	2.05	0.00	1.00
Pae_AF241171cds1_r_at	3.19	2.71	2.71	3.20	0.00	1.00
Pae_AF241171cds17_at	2.58	2.23	2.37	2.43	0.00	1.00
Pae_AF241171cds20_at	3.48	3.00	3.27	3.24	-0.01	1.00

Pae_AF241171cds28_at	3.56	3.40	3.48	3.45	0.02	1.00
Pae_AF241171cds29_at	2.56	2.23	2.45	2.32	0.01	1.00
Pae_L06157cds2_at	2.65	2.17	2.42	2.42	-0.01	1.00
Pae_L81176cds3_at	3.30	2.83	3.16	2.97	-0.01	1.00
Pae_L81176cds4_at	2.46	2.30	2.41	2.37	-0.01	1.00
Pae_M21651cds_at	2.33	2.36	2.39	2.29	0.00	1.00
Pae_M57501cds_g_at	2.30	2.25	2.42	2.09	0.02	1.00
Pae_orfB_at	2.63	2.52	2.57	2.60	-0.01	1.00
Pae_orfI_at	3.11	2.72	3.04	2.78	0.00	1.00
